# Discovery of Boronic Acids-Based β-Lactamase Inhibitors Through In Situ Click Chemistry

**DOI:** 10.3390/ijms26094182

**Published:** 2025-04-28

**Authors:** Nicolò Santi, Alessandra Piccirilli, Federico Corsini, Magdalena A. Taracila, Mariagrazia Perilli, Robert A. Bonomo, Francesco Fini, Fabio Prati, Emilia Caselli

**Affiliations:** 1 Department of Life Sciences, Università degli Studi di Modena e Reggio Emilia (UNIMORE), via Campi 103, 41125 Modena, Italy; nicolo.santi@unimore.it (N.S.); federico.corsini@unimore.it (F.C.); francesco.fini@unimore.it (F.F.); fprati@unimore.it (F.P.); 2 Department of Biotechnological and Applied Clinical Sciences, Università degli Studi dell’Aquila, via Vetoio, 67100 L’Aquila, Italy; alessandra.piccirilli@univaq.it (A.P.); mariagrazia.perilli@univaq.it (M.P.); 3 Department of Medicine, Case Western Reserve University School of Medicine, Cleveland, OH 44106, USA; mat8@case.edu (M.A.T.); robert.bonomo@va.gov (R.A.B.); 4 Research Service, Louis Stokes Cleveland Department of Veterans Affairs Medical Center, Cleveland, OH 44106, USA; 5 Clinician Scientist Investigator, Louis Stokes Cleveland Department of Veterans Affairs Medical Center, Cleveland, OH 44106, USA; 6 Departments of Medicine, Pharmacology, Molecular Biology and Microbiology, Biochemistry, and Proteomics and Bioinformatics, Case Western Reserve University School of Medicine, Cleveland, OH 44106, USA; 7 Cleveland Veteran Affair Medical Center for Antimicrobial Resistance and Epidemiology (Case VA CARES), Case Western Reserve University, Cleveland, OH 44106, USA

**Keywords:** in situ click chemistry, boronic acid, beta-lactamase inhibitors, KTGS, antimicrobial resistance, BATSI

## Abstract

In this study, we evaluated in situ click chemistry as a platform for discovering boronic acid-based β-lactamase inhibitors (BLIs). Unlike conventional drug discovery approaches requiring multi-step synthesis, protection strategies, and extensive screening, the in situ method can allow for the generation and identification of potent β-lactamase inhibitors in a rapid, economic, and efficient way. Using KPC-2 (class A carbapenemase) and AmpC (class C cephalosporinase) as templates, we demonstrated their ability to catalyse azide-alkyne cycloaddition, facilitating the formation of triazole-based β-lactamase inhibitors. Initial screening of various β-lactamases and boronic warheads identified compound **3** (3-azidomethylphenyl boronic acid) as the most effective scaffold for kinetic target-guided synthesis (KTGS). KTGS experiments with AmpC and KPC-2 yielded triazole inhibitors with *K*_i_ values as low as 140 nM (compound **10a**, AmpC) and 730 nM (compound **5**, KPC-2). Competitive inhibition studies confirmed triazole formation within the active site, while an LC–MS analysis verified that the reversible covalent interaction of boronic acids did not affect detection of the in situ-synthesised product. While KTGS successfully identified potent inhibitors, limitations in amplification coefficients and spatial constraints highlight the need for optimised warhead designs. This study validates KTGS as a promising strategy for BLI discovery and provides insights for further refinement in fighting β-lactamase-mediated antibiotic resistance.

## 1. Introduction

Nearly a century after penicillin’s discovery, the development of new antibiotics continues to lag behind the rapid evolution of antimicrobial resistance (AMR), which has significantly impacted β-lactams, the most widely used bactericidal agents [1,2]. Among various resistance mechanisms in Gram-negative bacteria, β-lactamases play a dominant role by hydrolysing the β-lactam ring, rendering these antibiotics ineffective [3,4,5,6]. β-lactamases are classified into serine β-lactamases (SBLs; classes A, C, D) and metallo-β-lactamases (MBLs; class B), each with distinct hydrolytic mechanisms [7,8,9,10]. Contemporary and highly clinically relevant enzymes, such as KPCs, NDMs, and OXAs families, contribute to resistance against expanded-spectrum cephalosporins (ceftazidime, ceftolozane and cefiderocol), monobactams, and even carbapenems, threatening the efficacy of “last-resort” antibiotics [11,12,13,14,15].

**Figure 1:**
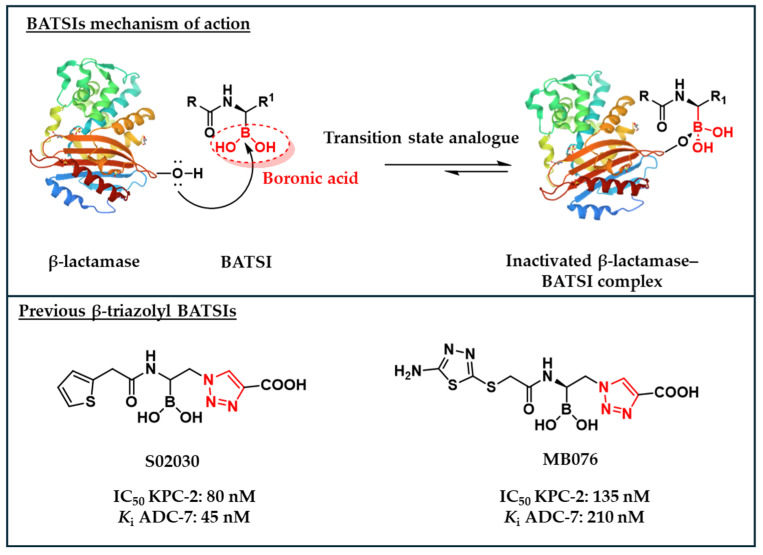
**Top panel**: BATSIs mechanism of action against β-lactamases. **Bottom panel**: structures and biochemical parameters of β-lactamase inhibition of S02030 and MB076.

Boronic acid transition state inhibitors (BATSIs) are covalent, reversible inhibitors mimicking the high-energy tetrahedral intermediate during β-lactam hydrolysis, offering a promising strategy against both SBLs and MBLs (Figure 1) [16,17,18]. The success of cyclic BATSIs, including commercially available vaborbactam and Phase III and I candidates taniborbactam and xeruborbactam, highlights their clinical relevance [4,19,20,21,22,23,24,25,26,27,28]. During the past decades, our group has developed a vast library of acyclic BATSIs [17,29,30,31,32,33,34]. The incorporation of a triazole group in the β-position of the boron atom led to the generation of potent BATSIs, including MB076 and S02030, both exhibiting strong activity against class A and C β-lactamases [17,19,35,36].

Both S02030 and MB076 feature a 1,4-disubstituted 1,2,3-triazole moiety, synthesised via copper-catalysed azide–alkyne cycloaddition (CuAAC) [17]. The triazole’s affinity for the β-lactamases active site, combined with the broad availability of azides and alkynes, opens up opportunities for creating novel BATSIs [37,38].

In this context, an attractive approach to obtain triazole-decorated molecules is represented by kinetic target-guided synthesis (KTGS), an appealing drug discovery method where the target protein catalyses the synthesis of its own inhibitors [39]. In KTGS, the protein-templated synthesis of bioactive compounds is achieved once the target facilitates the approaching of affine reagents with proper orientation, therefore lowering the energy of activation required for their irreversible binding [39]. KTGS allows for efficient screening of small molecules, particularly through in situ click chemistry, where the target enzyme guides the formation of disubstituted 1,2,3-triazoles from azides and alkynes [40,41,42,43,44,45,46,47,48]. In situ click chemistry efficiency is driven by the ability of the target enzyme to overcome the high energy of activation required for (3 + 2) cycloaddition reactions (>25–30 kcal/mol) [49,50,51]. Successful examples of this technique are mostly focused on the generation of non-covalent compounds, starting from previously reported inhibitors in a binary format (pairs of reagents) [39]. Although KTGS has been known for more than two decades, reports on its application remain relatively infrequent, with only a few unsuccessful examples being documented in the literature [39,52,53]. This limited adoption is probably due to the lack of robust and generalisable protocols. The difficulty in the detection of false negatives have also contributed to the slow progress of KTGS in drug discovery pipelines [50,54,55]. With fewer than 10% of KTGS studies focusing on covalent inhibitors, examples of this approach have not yet been reported for developing BATSIs or BLIs [39,46]. This highlights an untapped potential in using KTGS for covalent drug discovery, specifically in the design of novel inhibitors targeting β-lactamases.

**Figure 2:**
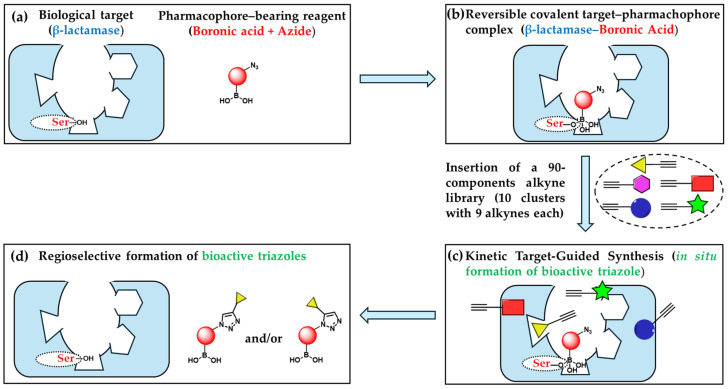
KTGS exemplification. (**a**) Selection of an appropriate β-lactamase and an azido-functionalised boronic acid warhead; (**b**) in situ experiment: once within the β-lactamase, the boronic acid forms a reversible covalent bond with the catalytic serine (in red); (**c**) upon insertion of an alkyne library, only alkynes with the proper orientation will be converted into triazoles by the enzyme. Alkynes attached to various coloured shapes indicate different functionalities linked to the alkynes; (**d**) the enzyme dictates a 1,4- or 1,5-regioisomeric preference for the few bioactive triazoles formed in situ.

Among the various KTGS formats available, the multicomponent format (involving a cluster of reagents) enables broader exploration compared with the binary format (pairing two reagents), facilitating faster and more efficient screening [39]. To accelerate BATSIs development and expand the KTGS scope, we applied a multicomponent in situ click chemistry approach using KPC-2 (class A) and chromosomal AmpC (class C) β-lactamases as templates (Figure 2). Our strategy involves synthesising azido-functionalised boronic acid “warheads”, leveraging the ability of boron to selectively target the β-lactamase catalytic serine located in the active site. The azido group enables further exploration within the active site through reactions with various alkynes, forming potentially bioactive triazoles. This work represents the first attempt to perform in situ click chemistry to develop triazole-based BATSIs using β-lactamases as scaffolds and a 90-component alkyne library to explore new chemical space.

## 2. Results

### 2.1. Design and Synthesis of the Warhead

**Figure 3:**
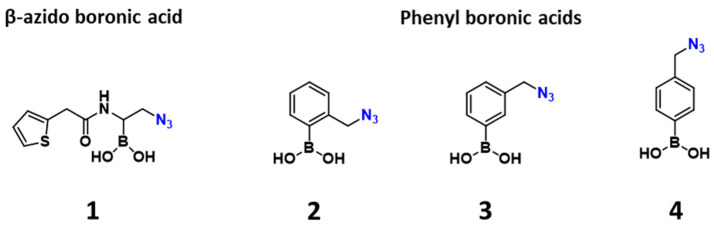
Structure for the designed warheads.

KTGS allows for the development of potent inhibitors without prior knowledge of their target affinities, provided that one of the two components demonstrates sufficient affinity to act as an anchor molecule [50]. Previously, even a low level of affinity was reported to be sufficient for the target-templated reaction [50]. Therefore, to enable in situ click chemistry within the β-lactamase’s binding pocket, a series of bifunctional “warheads” displaying two defined features were designed. Firstly, the presence of the boronic acid moiety serves as an anchor to the SBLs catalytic serine. Secondly, the azido functionalisation guarantees the clickable building block for generation of bioactive triazoles within the target active site. Thus, one 2-azido-1-acylamino-ethaneboronic acid and three azidomethyl-phenylboronic acids were synthesised and characterised (Figure 3). Inspired by one of the most potent BATSI, S02030 (Figure 2), [17,19,36] compound **1** was obtained through deprotection of the corresponding pinanediol ester, following a previously reported procedure [17]. Herein, the amide side chain from cephalothin is well known to interact with various β-lactamases through van der Waals and H-bonding interactions and can aid the process of recognition within the target [56]. Although being a promising scaffold for potential novel inhibitors, we observed by ^1^H NMR studies that compound **1** partially degrades in phosphate buffer (see Supplementary Information, Figures S1 and S2); therefore, this warhead was excluded from further studies. Other warheads inspired by previously developed acyclic boronic acids were not considered in this manuscript and will be explored in future works [20,33,34,35].

Phenyl boronic acids bearing 1,4-disubstituted 1,2,3-triazoles at the *m*- and *p*-positions were reported as potent inhibitors of KPC-2 by Zhou et al. [57,58]. For those compounds, docking studies revealed the potency of *m*-analogues deriving from the key hydrogen bond between the triazole nitrogen and T237 of KPC-2, while the *p*-substituted compounds were observed to take advantage of the π–π stacking interaction with W105. Interestingly, the azido starting materials, (3-(azidomethyl)phenyl)boronic acid (**3**) and (4-(azidomethyl)phenyl)boronic acid (**4**), were reported to exhibit sub-µM activity vs. KPC-2 [58]. Inspired by those scaffolds, compound **2** was also included. Whereas the role of phenyl boronic acids as BLIs have been extensively discussed in the past decades, KTGS might allow for further refining for this class of inhibitors. Chemically, azido-derivatives **2**, **3**, and **4** were synthesised via a single-step transformation starting from the respective bromo-derivatives, following previously reported procedures [57,58].

### 2.2. Inhibition (%) of the Warheads on Representative BLs

Selection of an appropriate warhead-bearing reagent is crucial for achieving a successful in situ click chemistry [39]. Thus, we assessed the inhibitory activity of the selected warheads against a pool of different β-lactamases to verify their binding specificity for the target. The affinity (inhibitory ability) of 2-4 against 12 representative β-lactamases from class A, B, C, and D was assessed as the percentage of inhibition at a fixed concentration of 200 µM (Table 1). Although boronic acids often yield potent nM inhibitors against SBLs, we reasoned that initiating the screening at higher inhibitor concentrations would enable a less stringent selection process, potentially identifying warheads with broader applicability, including activity against MBLs.

For both KPC-2 and chromosomal AmpC, this percentage was lowered to 100 µM due to high activity of some of the warheads at 200 µM.

Unsurprisingly, phenylboronic acids displayed better activity against class A and C, while they are less prone to inhibition vs. MBLs and class D. Among compounds **2**–**4**, the *m*-derivative **3** exhibited the best activity at 100 µM for KPC-2 and chromosomal AmpC, with 76% (Entry 1) and 100% (Entry 8) of inhibition, respectively. Thus, cpd **3** was chosen as the ideal warhead-bearing reagent and AmpC and KPC-2 as target proteins to start the in situ click chemistry screening.

### 2.3. Generation of a 90-Component Alkyne Library

**Figure 4:**
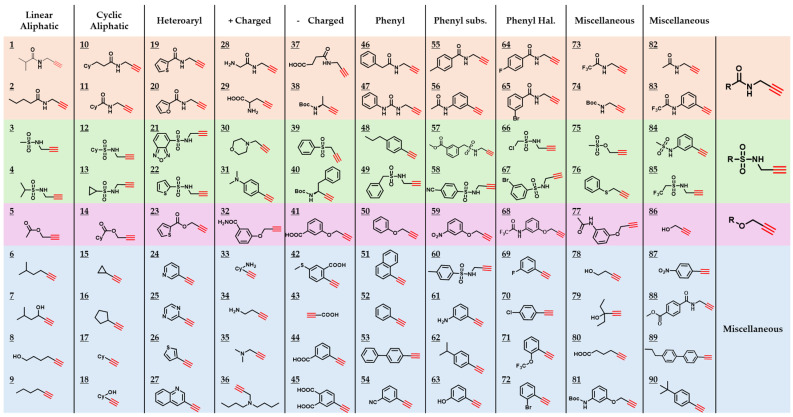
The 90-component alkyne library. Alkynes are separated into 10 clusters (columns) by their chemical and steric properties. Each cluster contains 9 alkynes, generally including an amido group (orange rows), sulphonamides (green rows), and ester or ether (magenta rows) scaffolds.

To facilitate KTGS in a multicomponent format with the azido-bearing warhead **3**, a diverse 90-member alkyne library was designed (Figure 4) [45,59]. Based on their availability, alkynes were either purchased from commercial sources or synthesised through a one- or two-step synthesis. Synthesis was carried out mostly using propargyl amine, propargyl alcohol, or propargyl bromide as sources of the alkyne functional group (see SI). To explore the chemical diversity in the potential bioactive triazoles formed, the library was divided into 10 clusters containing nine alkynes each. Clusters were created by including alkynes capable of interacting with the target via van der Waals force (columns 1 and 2), π-stacking and H-bonding (columns 3, 6, 7, and 8), and π-cation, π-anion, and H-bonding (columns 4 and 5). A miscellany of different relevant alkynes capable of interacting with the β-lactamases active site through some of the above interactions were included in the last two columns.

### 2.4. In Situ Click Chemistry with KPC-2

**Scheme 1:**
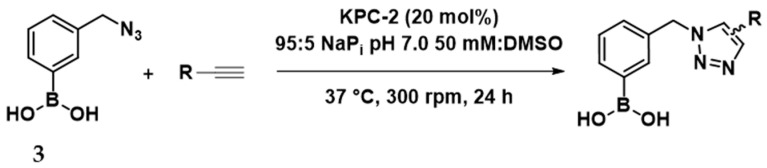
Multicomponent in situ click chemistry between **3** and an alkyne library in the presence of KPC-2.

Based upon the outcome of Table 1, initial efforts to perform the multicomponent in situ click chemistry were attempted using KPC-2 as template. Starting from the azido-functionalised boronic acid **3,** optimised conditions (see SI Table S2) were applied in combination with the 90-component alkyne library. The reactions were conducted in 0.2 mL microtubes in a 95:5 sodium phosphate 50 mM pH 7.0:DMSO mixture (final volume 100 µL), stirring at 300 rpm at 37 °C for 24 h (Scheme 1).

**Figure 5:**
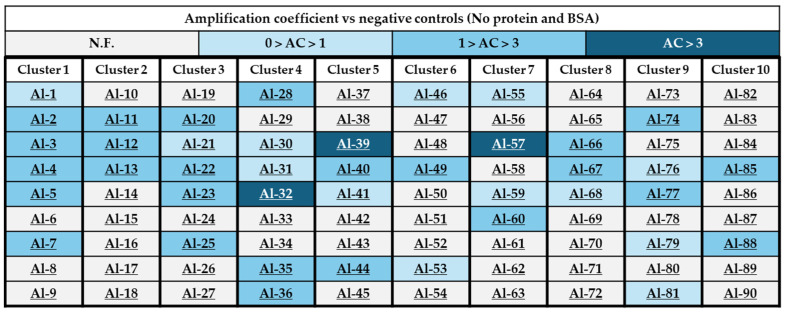
Cold map for the in situ click chemistry screening using KPC-2. Each cell corresponds to an alkyne. Dark blue denotes alkynes that generated triazoles with AC > 3 in the presence of the protein template.

The experiments were conducted in triplicate, using a reaction mixture containing one equivalent of warhead **3**, five equivalents of each alkyne cluster (9 alkynes), and 20 mol% KPC-2 as the catalyst. Product formation was monitored directly from the crude mixture using a hybrid quadrupole orbitrap LC–MS system in the ESI+ or ESI- mode after 24 h. Peak areas of the desired products were compared across three conditions: with KPC-2, without any enzyme, and with bovine serum albumin (BSA). Both no enzyme and BSA were used as controls; therefore, only a negligible amount of product was expected to be observed in their presence. The resulting ratio, named as the amplification coefficient (AC), was calculated from the peak area of the product obtained in the presence of KPC-2 vs. the one acquired with the controls [54,60]. CuAAC reactions employing CuSO_4_, sodium ascorbate, and tris(3-hydroxypropyltriazolylmethyl)amine (THPTA) served as the control, regioselectively yielding the 1,4-regioisomer. To evaluate the presence of the 1,5-regioisomer in the KTGS experiment, a second control was obtained using thermal reaction conditions (80 °C for 24 h), which resulted in a mixture of both 1,4- and 1,5-regioisomers. Out of 90 potential combinations, three triazole-based BATSIs (triazoles deriving from alkynes Al-32, Al-39, and Al-57) were identified with AC values exceeding three compared with the controls (Figure 5). Whereas the derivative from Al-39 was exclusively formed as a 1,4-regioisomer in the presence of KPC-2, both derivates from Al-32 and Al-57 were observed as mixture of regioisomers. Interestingly, the previously reported KPC-2 inhibitor 1,4-disubstituted 1,2,3-triazoles were not detected in this experiment, despite their known inhibition constants (*K_i_*) for KPC-2, ranging from 32 nM to 1 µM (alkyne derivative = cyclopropyl (Al-15), 3-pyridinyl (Al-24), 3-thiophenyl (Al-26), COOH (Al-43), and phenyl substituents (Al-52)) [57,58].

**Figure 6:**
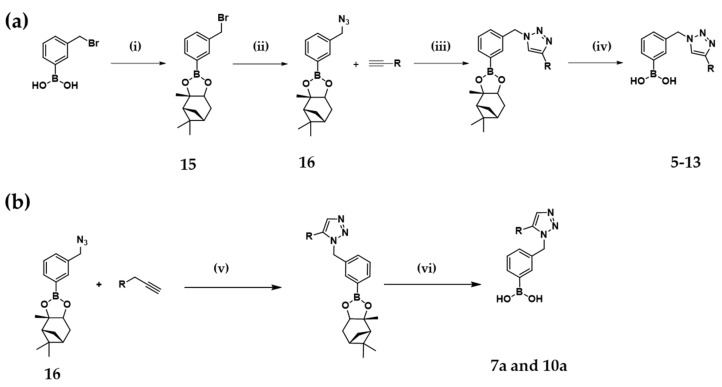
(**a**) General synthesis for 1,4-derivatives BATSIs. Reagents and conditions: (i) *m*-bromomethylphenyl boronic acid (1.0 eq.), (+)-pinanediol (1.0 eq.), THF, rt, on, quantitative. (ii) Compound **15** (1.0 eq.), NaN_3_ (10.0 eq.), MeOH, reflux, 5 h, 82%. (iii) Compound **16** (1.0 eq.), alkyne (1.0 eq.), CuSO_4_ (0.05 eq.), Na ascorbate (0.2 eq.), THPTA (0.05 eq.), *t-Bu*OH:H_2_O 1:1, 60 °C, 1–8 h, 80–99%. (iv) Protected triazoles (1.0 eq.), MeB(OH)_2_ (10.0 eq.), HCl 0.2 M (1.0 eq.), DCM, rt, on, 50–99%. (**b**) General synthesis for 1,5-derivatives BATSIs starting from compound **16**. (v) Compound **16** (1.0 eq.), alkyne (1.1 eq.), Cp*RuCl(PPh_3_)_2_ (0.025 eq.), THF, 70 °C, 8 h, 38–41%. (vi) Protected triazoles (1.0 eq.), MeB(OH)_2_ (10.0 eq.), HCl 0.2 M (1 eq.), DCM, rt, on, 45–50%.

To verify their biological activity, compounds **5** (triazole derivative from Al-32), **6** (derivative from Al-39), **7**, and **7a** (derivatives from Al-57) were synthesised, purified, and characterised at bench scale (Figure 6). While compound **5**, **6**, and **7** are 1,4-disubstituted 1,2,3-triazoles, compound **7a** referred to the 1,5-regioisomers of the triazole derived from Al-57, respectively. Compound **5a**, corresponding to the 1,5-regioisomer for the triazole derived from Al-32 could not be synthesised under the conditions reported in Figure 6b. Some control compounds, which either were not formed in the KTGS experiment or had AC values below three during the KPC-2 screenings, were also synthesised for comparative analysis; thus, derivatives **8** (from Al-2), **9** (Al-7), **10** (Al-12), and **11** (Al-31) were also prepared.

**Figure 7:**
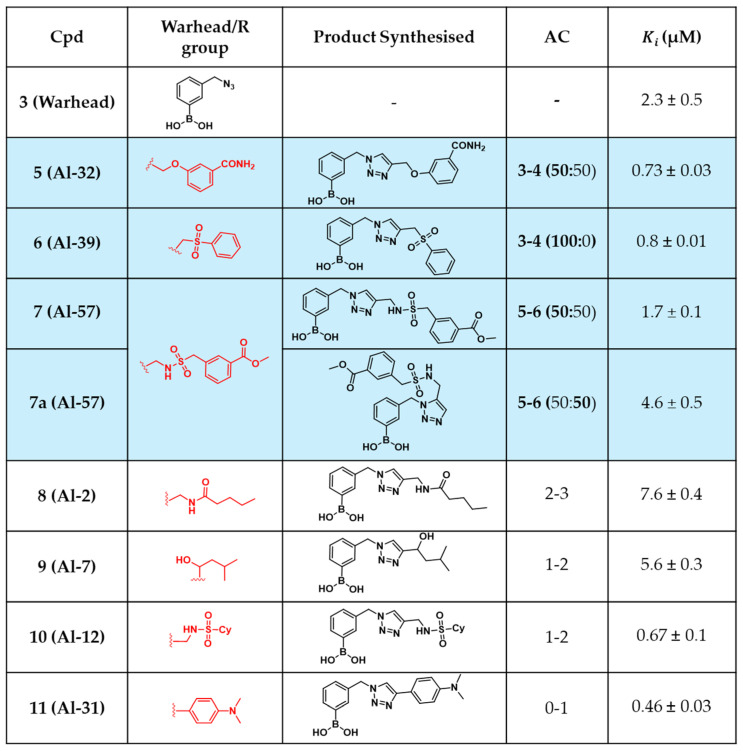
Compounds synthesised and their biological evaluation. AC = Amplification coefficient. In the AC column, between brackets reports the regioisomeric ratio observed (1,4- vs. 1,5-) and bold reports the synthesised regioisomer. Light blue indicates the compounds obtained from the KPC-2 screening. Substrate: nitrocefin (NCF). *K*_M_ (NCF) = 16 ± 1.2 µM; [NCF]: 100 µM; [KPC-2] = 67 nM.

The ability of the KTGS technique to identify good inhibitors of KPC-2 was evaluated by comparing the *K_i_* values of screened and control compounds against KPC-2. Using a four-step synthesis starting from commercially available *m*-bromomethylphenyl boronic acid, compounds **5**–**11** were prepared and tested biologically (Figure 7). Compound **7** (1,4-regioisomer), selected from the screening with an AC of 5-6, displayed a *K_i_* of 1.7 µM. The correspondent 1,5-regioisomer **7a** exhibited a *K_i_* of 4.6 µM. Compounds **5** and **6**, with ACs of 3-4, exhibited similar inhibitory activity (*K_i_* = 0.73 µM and 0.8 µM, respectively). For both cpds **5** and **6**, a 3-fold improvement compared with the warhead activity (*K_i_* = 2.3 µM) was observed. However, some control compounds, notably compound **11**, showed comparable or superior activity (*K_i_* = 0.46 µM), despite not being identified in the KTGS screening (false negative).

### 2.5. In Situ Click Chemistry with AmpC

**Figure 8:**
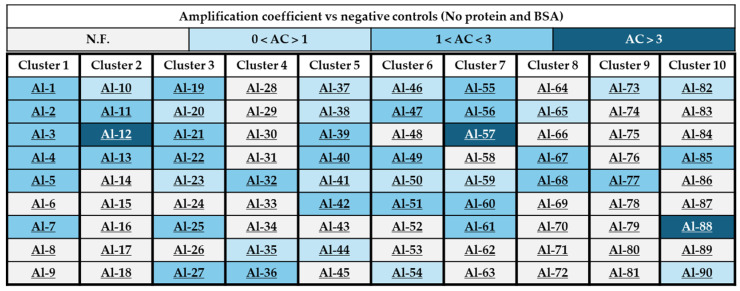
Cold map for the in situ click chemistry screening using AmpC. Each cell corresponds to an alkyne. Dark blue denotes alkynes that generated triazoles with AC > 3 in the presence of the protein template.

The results in Table 1 indicate that warhead **3** completely inhibits AmpC at a concentration of 100 µM, demonstrating a promising target–warhead combination for optimising and enhancing KTGS outcomes. Following the same conditions used for the multicomponent screening with KPC-2, the in situ click chemistry experiment was performed using AmpC as template. Similarly, out of 90-potential triazoles, only three products were formed with an amplification coefficient >3 (Figure 8). In the first case, cycloaddition of **3** with the alkyne Al-12 produced regioisomers **10** and **10a** (ratio 21:79) with an AC between 5 and 6. An acceptable AC was also obtained for Al-57, which was transformed into the triazole **7** and 7a in a 50:50 regioisomeric ratio. Eventually, the 1,5-product of the reaction between the warhead **3** and Al-88 was exclusively formed with AC 3-4 compared with controls.

Potentially bioactive BATSIs and controls were synthesised following the synthesis reported in Figure 6 and characterised. Despite several attempts, the 1,5-product **12a** (derivative from Al-88) could not be synthesised due to the low catalytic efficiency of the Cp*RuCl(PPh_3_)_2_ catalyst used, and only the 1,4-product **12** was successfully obtained. Similarly, for compound **5a** (derivative from Al-32), a potential inhibitor derived from the KPC-2 screening, the same outcome was observed. Notably, to the best of our knowledge, only one example of RuAAC involving a boronic acid has been reported in the literature, and not with the ruthenium catalyst available to us [61,62]. This highlights the added value of KTGS as it can reveal bioactive compounds that would otherwise be challenging or inefficient to access through conventional synthetic methods.

**Figure 9:**
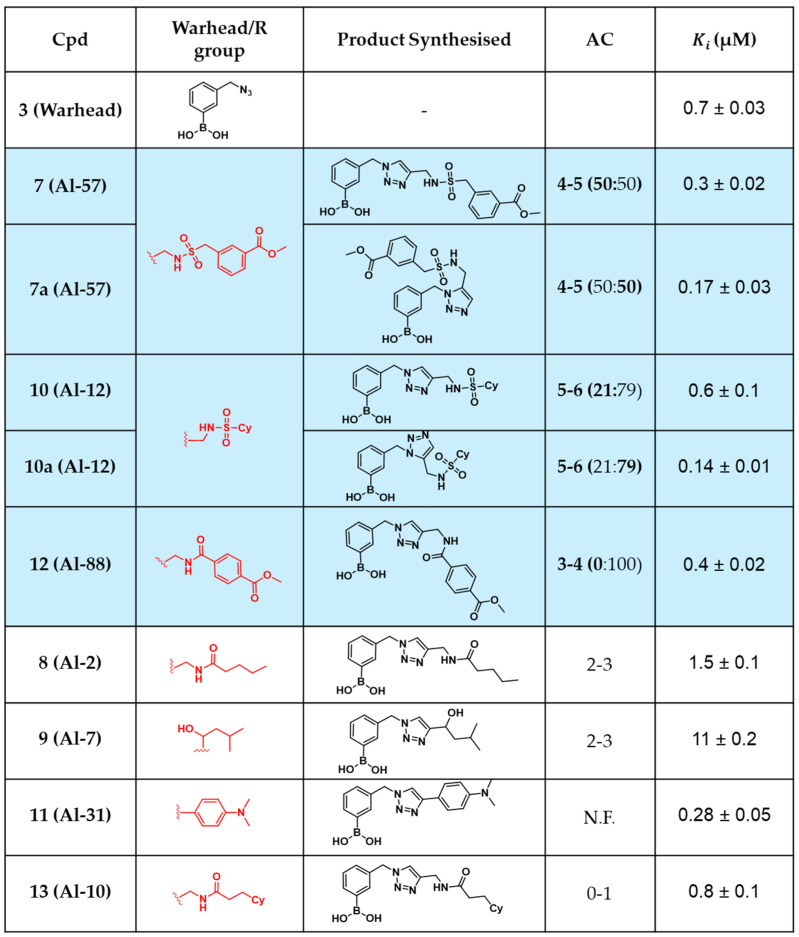
Compounds synthesised and their biological evaluation. AC = Amplification coefficient. In the AC column, between brackets reports the regioisomeric ratio observed (1,4- vs. 1,5-) and bold reports the regioisomers synthesised. Light blue indicates the compounds from the AmpC screening. Substrate: nitrocefin (NCF). *K*_M__NCF = 118 ± 2 µM; [NCF]: 100 µM; [AmpC] = 36 nM.

Microbiological assays with AmpC highlighted the 1,5-regioisomer **10a** (Figure 9, AC 5-6) as the most potent inhibitor, showing a *K_i_* value of 140 nM. The corresponding 1,4-regioisomer **10**, observed at 21% in the screening, displayed nanomolar potency (*K_i_* = 600 nM), yet it was four-fold weaker with respect to the 1,5-regioisomer. Compounds **7** and **12**, identified with ACs of 4-5 and 3-4, respectively, were also effective inhibitors (*K_i_* = 300 and 400 nM, respectively). The relative 1,5-regioisomer **7a** exhibited an excellent inhibitory activity with *K_i_* of 170 nM. Notably, warhead **3** itself exhibited a *K_i_* of 700 nM. Control compounds (cpds **8**, **9**, **11**, and **13**) displayed a various range of activity. While controls **8** and **9** exhibited worse inhibitory activity (*K_i_* = 1.5 and 11 µM, respectively) when compared with warhead **3**, cpds **11** and **13** demonstrated similar activity (*K_i_* = 280 and 800 nM, respectively) to the triazoles selected from the AmpC screening.

**Scheme 2:**
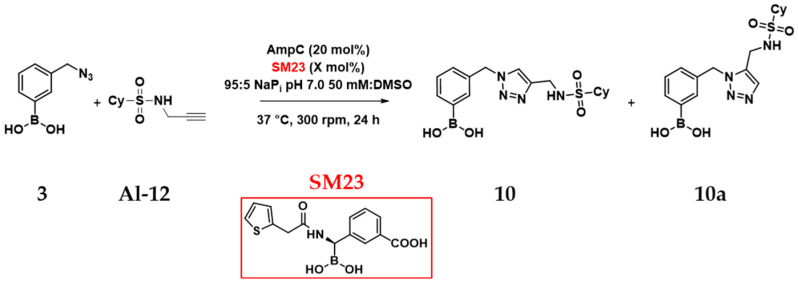
In situ click chemistry between 3 and Al-12 in the presence of AmpC and SM23 (red box).

To assess whether the in situ click chemistry takes place within the AmpC active site, a control experiment was performed. SM23, a known AmpC inhibitor (*K_i_* 1 nM) previously reported by our group [18,63], was added to the reaction aiming to assess if the catalysis was affected by a BATSI obstructing the target active site (Scheme 2). For this experiment, a binary combination between warhead **3** and a model alkyne (Al-12) was selected.

In the absence of SM23, 20 mol% AmpC catalyses the formation of both regioisomers **10** and **10a** (Table 2, Entry 3, regioisomeric ratio 21:79) compared with no enzyme (Entry 1) and BSA (Entry 2). Predictably, even 1 mol% SM23 in combination of 20 mol% AmpC affects the ratio of formation of triazoles **10**/**10a**. In this case, AmpC catalyses only the formation of **10a** with an AC of 1-2 (Entry 4). An even more dramatic drop in conversion is observed when the amount of SM23 was equal to the AmpC one, with neither AC > 1 nor regioselectivity observed (Entry 5). Therefore, those findings suggest that the in situ click chemistry between warhead **3** and alkyne Al-12 happens within the AmpC catalytic site.

**Figure 10:**
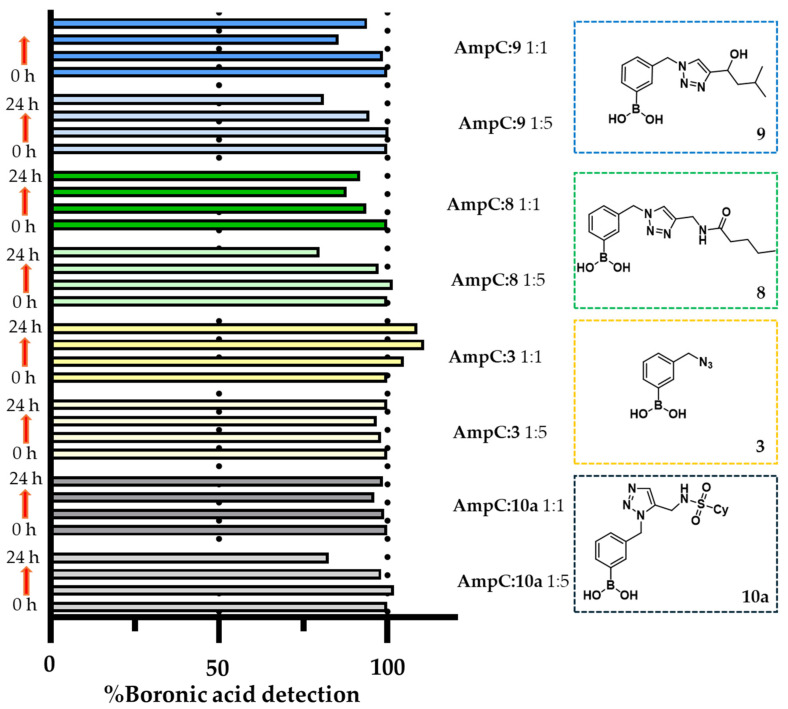
The % of detection of **10a** (grey box), **3** (yellow box), **8** (green box), and **9** (blue box) over the course of the 24 h using the t-SIM method. Each cpd was tested in presence of 20% mol of AmpC or stoichiometric amount of AmpC. Each box includes 4 columns corresponding to % boronic acid detected at a certain time point (0, 8, 16, and 24 h).

Given that boronic acids act as transition-state inhibitors by binding the catalytic serine of β-lactamases through a reversible covalent bond, we aimed to determine whether the in situ click chemistry reaction products were quantitatively detected during LC–MS analysis. Although boronic acids are reversible binders, their fast on–slow off behaviour raised concerns that triazole product formation might be underestimated due to covalent adducts forming with the protein target [64]. To investigate this, compound **10a**, warhead **3**, and compounds **8** and **9** were incubated with AmpC (1:1 or 1:5 protein:compound ratio), and their detection was monitored over 24 h (Figure 10). The selection of these compounds was designed to assess whether potent inhibitors (e.g., compound **10a**, *K_i_* = 140 nM) had a different impact compared with less effective scaffolds (e.g., compound **9**, *K_i_* = 11 µM). The percentage of boronic acids detected was estimated using peak area measurements in the LC–MS-targeted SIM mode, with quantification being performed using calibration curves. The experiments were conducted at 37 °C in a 95:5 sodium phosphate 50 mM pH 7.0:DMSO mixture.

For compound **10a**, the most potent AmpC inhibitor reported in this study, a slight decrease in detection was observed over 24 h, with 83% of **10a** remaining in the presence of 20 mol% of the target protein. However, when **10a** and AmpC were incubated at a stoichiometric ratio, **10a** levels remained stable throughout the experiment. A similar trend was observed for compounds **8** and **9**, indicating that the inhibitors affinity for the protein target did not influence its detection in the LC–MS analysis. Additionally, no differences were observed when warhead **3** was incubated with either a limiting or stoichiometric amount of AmpC. These findings suggest that the covalent nature of the warhead and the in situ click chemistry products did not interfere with the KTGS experiments or their outcomes.

### 2.6. Molecular Docking Studies

**Figure 11:**
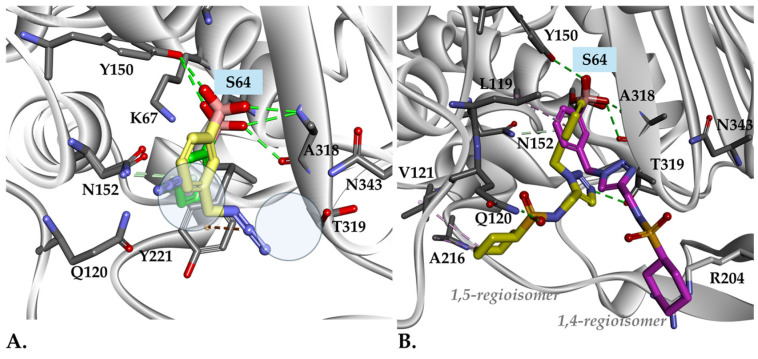
(**A**) The molecular docking of warhead **3** into the active site of *E. coli* AmpC with the tetrahedral conformation and known interaction with the active site catalytic (conserved) residues. (**B**) The molecular docking of compound **10**/**10a** shows both regioisomers making productive interactions within AmpC active site, with the boronic acid pharmacophore being located in the catalytic tetrahedral (H-bonds Y150 or K67 and A318). Light blue boxes denote catalytic serine(s).

To gain a better understanding of the mechanism of the AmpC-catalysed in situ click chemistry, molecular docking simulations were implemented. LibDock score was used as a relative metric for comparing ligand poses (warhead **3** and compounds **10** and **10a**) derived from the AmpC screening, prioritising complementarity and stability in the binding site (e.g., VDW vs. electrostatic) [65,66]. In this regard, the higher the score, the higher the probability of a compound with a high binding affinity. Based on this scoring, for AmpC, the conformations of warhead **3** have the lowest score (less than 80). 1,5-regioisomers **10a** have the highest score (120–123), followed by its relative 1,4-regioisomers **10** (112–115). Binding free energy was also estimated between each ligand and enzyme. Using CHARMm-based energies and implicit solvation methods, the overall binding free energy was determined from ligand energy, protein energy, and entropic energy [67]. The interaction energy protocol was used to calculate the non-bound interactions (i.e., van de Waals, electrostatic) between each compound and active site residues. As expected, warhead **3** had the worst interaction energy (−12.23 kcal/mol), while the best compound **10a** had the highest (−21.3 kcal/mol), therefore supporting the data obtained from the AmpC screening. Furthermore, the molecular docking of warhead **3** into the active site of *E. coli* AmpC revealed that hydroxy boronate maintains the tetrahedral conformation observed in prior X-ray structures, forming hydrogen bonds with conserved catalytic residues Y150 and A318 (Figure 11A). Moreover, the phenyl ring engages in π–π interactions with N152 or Y221. Warhead **3** allows for two different conformations with comparable interaction energies (~12.23 kcal/mol). The alkyne fragments may preferentially bind with any of the conformations (left or right), and the new formed triazole will bind with residues T319, N343, or Q120. The docking of the 1,4-regioisomer **10** and the 1,5-regioisomer **10a** into the active site of AmpC shows two distinct binding patterns (Figure 11B). The shared phenylboronate core maintains steric interactions with N152. The 1,5-regioisomer **10a**, which has the highest binding affinity (140 nM) and the most favourable interaction energy (−21.43 kcal/mol), forms interactions between its triazole and T319 and between SO_2_ and Q120. Its cyclohexane group engages in steric interactions with A216 and V121. In contrast, the 1,4-regioisomer **10a** has a less favourable interaction energy (−13.38 kcal/mol) and positions its cyclohexane group toward R204.

## 3. Discussion

In this work, we investigated whether in situ click chemistry can be readily employed as a platform to discover boronic acid-based β-lactamase inhibitors. Previously, triazole-containing boronic compounds were discovered following standard procedures (CuAAC) on the mgs scale, leading to the discovery of potent β-lactamase inhibitors [17]. In contrast, the synthesis of inhibitors within the catalytic site of an enzyme using a multicomponent assay offers different advantages. Firstly, this approach is timesaving, as 90 reactions can potentially be conducted simultaneously, allowing for a rapid identification of inhibitors. A second advantage is operability, as the boronic group contained in the warhead does not require tedious protection and deprotection steps, which are often required when a click reaction is conducted under conditions of chemical catalysis with Cu(I). Eventually, small-scale reactions represent an economically viable solution for avoiding an excessive consumption of reagents. In this study, two enzymes belonging to separate classes of β-lactamases were used as the template, the class A carbapenemase KPC-2 and the class C cephalosporinase AmpC. Despite sharing the same mechanism of action, those enzymes possess different specificity towards β-lactam antibiotics, reflecting the diversity of their catalytic sites. Analysis on KPC-2 and AmpC active sites with the DoG site scorer, an automated tool able for predicting and evaluating binding pockets for druggability assessment, reveals significant differences on the physicochemical properties of the two target pockets chemistry (see SI Table S4 and Figures S11–S14) [52]. While KPC-2 possess a narrow and enclosed active site, suggesting a lower pre-disposition to accommodate KTGS building blocks, AmpC, display larger volume, broader surface, and favourable physicochemical parameters. Microbiological screening of the azidomethylphenyl boronic warheads designed revealed boronic warhead **3**, bearing the substituent in the *meta* position, as the most active towards both KPC-2 and Amp-C. When the in situ click chemistry experiment was conducted in the presence of those enzymes, few alkynes were selected for the formation of the triazoles, indicating that both β-lactamases can catalyse the azido-alkyne cycloaddition reaction. Catalysis in the presence of KPC-2 reveals the formation of three out of 90 potential triazoles with an amplification coefficient > 3 relative to controls. When in situ click chemistry is repeated with AmpC, whose catalytic site exhibit more favourable druggability parameters than KPC-2, results are comparable in both amplification coefficient and number of hits. Interestingly, in some cases, AmpC dictates a specific preference for the formation of 1,5-regioisomer triazoles (i.e., with Al-12 and Al-88).

These results further demonstrate that overcoming the energy barrier typical of the cycloaddition reaction is possible only with appropriate spatial orientation in the catalytic site of both azide and alkyne. To prove that click chemistry takes place within the catalytic site, a binary experiment was attempted with warhead 3 and alkyne Al-12 in the presence of SM23, a potent AmpC inhibitor (*K_i_* = 1 nM). At a concentration of 1 mol% inhibitor, the AC drops from a value of 5−6 without inhibitor to a value of 1−2. With an equimolar concentration of SM23 and enzyme (20%), a negligible triazole peak is visible, suggesting that the high affinity of the inhibitor for the catalytic site prevents the appropriate spatial arrangement of azidoboronate and alkyne for cycloaddition. These combinations of results prove the role of β-lactamases in promoting cycloaddition reaction within their active site. Further control experiments confirmed that both the warhead and reaction products were efficiently detected during LC–MS analysis when incubated with AmpC. This suggests that the reversible covalent bond between the boronic acid and the β-lactamase catalytic serine does not interfere with the quantification of the in situ click chemistry products. For both KPC-2 and AmpC, the synthesised triazoles prove to be better inhibitors than warhead **3** (*K_i_* vs. KPC-2 = 2.3 µM; *K_i_* vs. AmpC = 700 nM), reaching values of *K_i_* = 730 nM for compound **5**, the best KPC-2 inhibitor, and *K_i_* = 140 nM for **10a**, the best AmpC inhibitor. In support of our findings, docking simulations performed reveal that compound **10a** has both the highest binding energy and the highest LibDock score compared with the regioisomer **10** and warhead **3**. These data further confirm the validity of KTGS as a platform for BLIs discovery.

Triazole-based BATSIs derived from the reaction of other alkynes that were not selected in situ were also synthesised and tested. Some of them proved to be comparable inhibitors as the hit compounds against both enzymes, suggesting how this technique can un-detect potential inhibitors. This problem of “false negative”, however, is inherent in the technique itself [49,50,51,68]. Furthermore, structural rigidity and steric constraints of the boronic acid scaffold likely restricted the conformational freedom required for effective alignment of reactants. While the reversible covalent interaction between the catalytic serine and the electrophilic boron anchors the warhead within the active site, it also limits the spatial and conformational flexibility needed for alkyne fragments. These limitations emphasise the necessity of designing more flexible and adaptable warheads, such as warhead **1**, that might balance inherent activity with catalytic efficiency for inhibitors formation through KTGS applications. Future work will focus on expanding the application of in situ click chemistry to a broader range of warheads and β-lactamases, with particular emphasis on optimising reaction conditions tailored to each specific target.

## 4. Materials and Methods

### 4.1. Chemistry

Methods. All reactions dealing with air- and moisture-sensitive compounds were carried out in dry reaction vessels under a nitrogen atmosphere. Flash column chromatography (CC) was performed using silica gels (particle size 35–70 µm). Solvents used in CC were commercially available and distilled before use. Thin-layer chromatography was used for product detection using silica gel-coated plates, with visualization effected via exposure to UV light at 254 nm or staining and heating with potassium permanganate (KMnO_4_) or Pancaldi solution (phosphomolybdic acid and Ce(IV)sulphate in 4% sulphuric acid).

Instrumentation. ^1^H NMR, ^13^C NMR, and ^11^B NMR spectra were obtained on a Bruker Ascend 400 instrument at 400 MHz (^1^H NMR at 400 MHz, ^13^C NMR at 101 MHz, ^11^B NMR at 128 MHz) and Bruker Ascend 600 instrument at 600 MHz (^1^H NMR at 600 MHz, ^13^C NMR at 151 MHz, ^11^B NMR at 193 MHz) at ambient temperature with CDCl_3_, CD_3_OD, D_2_O, or *d*_6_-DMSO as the deuterated solvent. All chemical shifts δ are reported in parts per million (ppm), and the residual solvent peak was used as an internal reference: proton (CDCl_3_ δ 7.26, MeOD δ 3.31, D_2_O δ 4.79, DMSO δ 2.50), carbon (CDCl_3_ δ 77.0, MeOD δ 49.0, DMSO δ 39.5), or tetramethylsilane (TMS δ 0.00) were used as a reference. Coupling constants (J) were reported in Hertz (Hz) and referred to apparent peak multiplets. Data for ^1^H NMR spectra were reported as follows: chemical shift (ppm), multiplicity (given as s (singlet), d (doublet), t (triplet), q (quartet), m (multiplet), br (broad) or a combination of them), coupling constants (Hz), and integration. ^13^C NMR and ^11^B NMR were only reported as chemical shifts. High-resolution mass spectra (HRMS) were recorded on an Ultimate 3000 UHPLC coupled to a Q-Exactive hybrid quadrupole-orbitrap mass spectrometer via an HESI-II heated electrospray ionisation source (Thermo Fisher Scientific, Waltham, MA, USA).

Materials. All reagents and solvents were of commercial quality from freshly opened containers. All substances that are not described in the following synthetic procedures were obtained from commercial suppliers, such as:BLD Pharmatech GmbH (Reinbek, Germany), Merck KGaA (Darmstadt, Germany), Thermo Fisher scientific, Santacruz biotechnology (Dallas, TX, USA) and Enamine (Kyiv, Ukraine). Anhydrous 1,4-dioxane, toluene, and other solvents were purchased and used under a N_2_ atmosphere.

Synthesis and characterisation data. Detailed synthetic procedures, compounds characterisation, and spectral data are available in the Supplementary Materials.

### 4.2. In Situ Click Chemistry

Materials. Reactions were incubated in a TS-100C (Biosan, Riga, Latvia) thermo-shaker equipped with an interchangeable heating block for microtubes and PCR plates.

Cluster of alkynes preparation. Mixtures of alkynes (cluster 1 to 10, see Figure 4) were prepared from a dimethylsulphoxide (DMSO) 50 mM stock solution of each alkyne; mixtures X (10 clusters of 9 alkynes): mixing 10 µL of DMSO to 10 µL of stock solutions of 9 alkynes to reach a 5 mM final concentration of each alkyne.

Azide preparation. Warhead **3** was dissolved in DMSO at a 50 mM final concentration. For reactions, warhead **3** was diluted to 1 mM in DMSO.

Binary and multicomponent KTGS. In a 0.2 mL microtube, 2.5 µL of azide **3** (stock concentration of 1 mM in DMSO), 2.5 µL of an alkyne mixture (stock 5 mM in DMSO), 6.9 µL of AmpC (stock 72 µM in NaP_i_ pH 7.0) and 88.1 µL of 50 mM sodium phosphate buffer pH 7.0 were mixed to reach a final volume of 100 µL. The final concentrations are the following: azide 25 µM (1 eq.), alkyne cluster 125 µM (5 eq. × 9), AmpC 5 µM (20 mol% or 0.02 eq.), DMSO 5%. For experiments in the presence of KPC-2, 3.27 µL of KPC-2 (stock 153 µM in NaP_i_ pH 7.0) was used to afford a final concentration of 5 µM (20 mol% or 0.02 eq.). The microtube was shaken at 300 rpm at 37 °C for 24 h. Reactions were transferred into a LC–MS vial and directly injected (10 µL) for liquid chromatography–mass spectrometry (LC–MS). Reactions were analysed with an Ultimate 3000 UHPLC coupled to a Q-exactive hybrid quadrupole-orbitrap mass spectrometer via an HESI-II heated electrospray ionisation source. The chromatographic separation was performed injecting a 10 µL sample volume on a Hypersil Gold C18 100 × 2.1 mm, 1.9 µm column (Thermo Fisher Scientific), kept at 30 °C eluting with 0.3 mL/min flow of ultrapure water (A) and methanol (B), both with 0.1% formic acid as mobile phase. A linear gradient profile was applied from 2% to 98% B over 10 min followed by a reconditioning step pending successive sample injection. A data-dependent mass spectrometric data acquisition strategy was used for full MS/DD-MS2. Full MS experiments were acquired by alternating positive and negative ionisation mode at 35,000 FWHM (at 200 *m*/*z*) resolving power with a 250 < *m*/*z* < 1000 scan range ,and AGC target and maximum injection time set at 3 × 10^6^ and 243 ms, respectively. The top 2 mono-charged ions were selected for MS2 acquisition at 17,500 FWHM using a 4.3 *m*/*z* (1.0 offset) wide isolation window, with AGC set at 2 × 10^5^ and 100 ms maximum injection time. An inclusion list was eventually used to target the MS2 spectra acquisition of preferred ion species. Detection was based on calculated [M+H]^+^ and [M−H]^−^ molecular ions with a 5 ppm accuracy tolerance for their respective ion chromatogram extraction. Peak retention time and area of detected target compounds was used for their detection and semi-quantitative evaluation in between reaction batches.

For controls (buffer and BSA), AmpC/KPC-2 is replaced with no enzyme (buffer volume 95 µL) and BSA (stock 100 µM, final concentration 5 µM, buffer volume 90 µL). Hits were identified in each cluster by mass and retention time and compared with both controls (buffer and BSA) and synthetically prepared triazoles obtained in mixtures. The peak area of each triazole obtained in the presence of the proteins (AmpC and KPC-2) was compared with the peaks observed with controls to calculate the amplification coefficient (AC, PA_KPC-2/AmpC_/PA_No protein/BSA_).

CuAAC positive controls. In a 0.2 mL microtube, 2.5 µL of azide **3** (stock concentration of 1 mM in DMSO), 2.5 µL of an alkyne mixture (stock 5 mM in DMSO), 2.5 µL of CuSO_4_ (stock 1 mM in water), 2.5 µL of sodium ascorbate (stock 5 mM in water) 2.5 µL of THPTA (stock 2 mM in water), 40 µL of water, and 47.5 µL of *t-Bu*OH were mixed to reach a final volume of 100 µL. The final concentrations are the following: azide 25 µM (1 eq.), alkyne cluster 125 µM (5 eq. × 9), CuSO_4_ 25 µM (1 eq.), sodium ascorbate 125 µM (5 eq.), THPTA 50 µM (2 eq.), DMSO 5%, *t-Bu*OH 47.5%, water 47.5%. The microtube was shaken at 300 rpm at 37 °C for 24 h. Reactions were transferred into an LC–MS vial and analysed in the same way as described in the binary and multicomponent KTGS section above.

Thermal reactions. In a 0.2 mL microtube, 2.5 µL of azide **3** (stock concentration of 1 mM in DMSO), 2.5 µL of an alkyne mixture (stock 5.1 mM in DMSO), and 95 µL of 50 mM sodium phosphate buffer pH 7.0 were mixed to reach a final volume of 100 µL. The final concentrations are the following: azide 25 µM (1 eq.), alkyne cluster 125 µM (5 eq. × 9). The microtube was shaken at 300 rpm at 80 °C for 24 h. Reactions were transferred into an LC–MS vial and analysed in the same way as described in the binary and multicomponent KTGS section above.

### 4.3. Microbiology and Determination of K_i_

The β-lactamases used in the present study were from a Clinical Biochemistry Laboratory collection (Department of Biotechnological and Applied Clinical Sciences, University of L’Aquila, L’Aquila, Italy). All enzymes show a purity degree of higher than 95%. The concentration of each enzyme was determined by a Bradford assay. The *K*_M_ values of each β-lactamase for nitrocefin were determined following the hydrolysis of the substrate under the initial rate and by linearisation of the Michaelis–Menten equation using the Hanes-Woolf method [69].

Competitive inhibition assays were monitored directly using, as a reporter substrate, 100 µM nitrocefin. *K*_i_ values were calculated using the following equation:

v_0_/v_i_ = 1 + (*K*_M_ × I)/(*K*_M_ + S) × *K*_i_ (1)

where v_i_ and v_0_ are the initial rates of hydrolysis of nitrocefin with or without an inhibitor, respectively; I is the concentration of the inhibitor, *K*_i_ is the inhibition constant, *K*_M_ is the Michaelis–Menten constant, and S is the concentration of reporter substrate. The plot v_0_/v_i_ versus [I] yielded a straight line of slope *K*_M_/(*K*_M_ + S) × *K*_i_ [70].

The IC_50_ value for each compound was graphically calculated, plotting residual activity (%) versus [I].

For the β-lactamases test at a fixed concentration previously reported in Table 1, the following conditions were applied (Table 3):

### 4.4. Docking Studies

The crystal structures of *E. coli* AmpC (PDB: 1KE4) were used for molecular docking. The structures were prepared for docking using DS2020 version 20.1 (Discovery Studio Client 2020, Dassault Systèmes BIOVIA, San Diego, CA, USA) modelling software. The crystallographic waters were removed, and the structures were further minimised using the conjugate gradient method, with an RMS gradient of 0.001 kcal/(mol × Å). Generalised born with a simple switching (GBSW) solvation model was used, and long-range electrostatics were treated using a particle mesh Ewald method with a periodic boundary condition. The SHAKE algorithm was applied. The azide warhead **3** and compounds were built and docked into the active site of AmpC using the *LibDock* protocol. In this high-throughput algorithm, ligand conformations are aligned to polar and apolar receptor interaction sites (hotspots), and the best scoring poses are reported. Because some of the output poses may have hydrogen atoms near the receptor, a CHARMm minimisation step was enabled to optimise the docked poses. Furthermore, the generated poses were analysed and the best poses were ranked based on the scoring functions (a higher score predict a more favourable binding affinity), and the minimum distance from boron atom to catalytic serine (S64 for AmpC) was used to create the enzyme–ligand complexes. The complexes were further minimised to assess the stability of the systems. From the initially generated conformations (several hundreds/compound), the first 50 conformations with the highest LibDock score were selected and analysed. Binding free energy was estimated between each ligand and enzyme. Using CHARMm-based energies and implicit solvation methods, the overall binding free energy was determined from ligand energy, protein energy, and entropic energy. The interaction energy protocol was used to calculate the non-bound interactions (i.e., van de Waals, electrostatic) between each compound and active site residues.

## Figures and Tables

**Table 1:** The % of inhibition at a fixed concentration of 200 µM for warheads 1–4 against 12 β-lactamases.

Entry	β-lactamase 1	Class	2	3	4
1	KPC-2 ^2^	A	35	76	72
2	CTX-M-15		0	42	22
3	KPC-53		54	65	55
4	SHV-12		14	38	48
5	NDM-1	B	23	20	24
6	VIM-1		23	24	33
7	IMP-1		12	11	22
8	AmpC ^2^	C	57	100	81
9	ADC-25		27	67	46
10	CMY-2		19	79	67
11	OXA-24	D	20	24	26
12	OXA-48		<1	2	<1

^1^ Substrates, proteins and substrates concentrations, and relative constants are reported in Section 4.3. ^2^ The % of inhibition at 100 µM.

**Table 2:** AmpC catalytic activity in the presence of an SM23 inhibitor. Acn = Amplification Coefficient.

Entry *	Enzyme	%SM23	Regioselectivity (10:10a)	AC 10/10a
1	None	-	50:50	-
2	BSA (20 mol%)	-	60:40	-
3	AmpC (20 mol%)	-	21:79	5–6
4	AmpC (20 mol%)	1 mol%	0:100	1–2
5	AmpC (20 mol%)	20 mol%	50:50	0–1

* Reactions were run in 0.2 mL microtubes with a total volume of 100 µL. Conditions: 3 (1 eq.), Al-12 (5 eq.), 95:5 sodium phosphate 50 mM pH 7.0: DMSO; 300 rpm; 37 °C; 24 h. For Entry 4 and 5, SM23 was added as a last component before starting the reaction.

**Table 3:** Conditions and kinetic values for the 12 β-lactamases tested.

β-lactamase	Buffer	Substrate	*K*_M substrate_	[Substrate]	[β-lactamase]
KPC-2	NaPi ^1^	NCF ^3^	10 ± 1 µM	50 µM	1 nM
CTX-M-15	NaPi	NCF	35 ± 1 µM	25 µM	2.5 nM
KPC-53	NaPi	NCF	106 ± 2 µM	100 µM	30 nM
SHV-12	NaPi	NCF	50 ± 3 µM	25 µM	7 nM
NDM-1	HEPES ^2^	MPM ^4^	80 ± 1 µM	100 µM	4.5 nM
VIM-1	HEPES	MPM	130 ± 4 µM	150 µM	22 nM
IMP-1	HEPES	MPM	30 ± 1 µM	80 µM	13 nM
AmpC	NaPi	NCF	118 ± 2 µM	142 µM	14 nM
ADC-25	NaPi	NCF	120 ± 3 µM	24 µM	3 nM
CMY-2	NaPi	NCF	8 ± 1 µM	24 µM	2.5 nM
OXA-24	NaPi	NCF	29 ± 1 µM	142 µM	4 nM
OXA-48	NaPi	IMI ^5^	13 ± 1 µM	50 µM	75 nM

^1^ NaPi 50 mM pH 7.0; ^2^ HEPES 20 mM pH 7.0+ 20 µM Zn. ^3^ NCF = Nitrocefin; ^4^ MPM = Meropenem; ^5^ IMI = Imipenem.

## Data Availability

The raw data supporting the conclusions of this article will be made available by the authors on Zenodo (https://zenodo.org/).

## Supplementary Materials

The following supporting information can be downloaded at: https://www.mdpi.com/article/10.3390/ijms26094182/s1.
