# Supplementary material for: Discovery of Boronic Acids-Based β-Lactamase Inhibitors Through In Situ Click Chemistry
Source: Int J Mol Sci. 2025 Apr 28;26(9):4182. doi: 10.3390/ijms26094182 (PMC12071365; doi:10.3390/ijms26094182)

Supporting Information

Discovery of boronic acids-based β-Lactamase Inhibitors through *In Situ* Click Chemistry

Nicolò Santi[1], Alessandra Piccirilli[2], Federico Corsini[1], Magdalena A. Taracila[3,4], Mariagrazia Perilli,[2] Robert A. Bonomo,[3,4,5,6,7] Francesco Fini,[1] Fabio Prati[1] and Emilia Caselli[1,*]

Contents

Preparation of starting materials and products……………………………………………..3

Degradation pathway for warhead-bearing azide ***1***……………………………………………………3

General procedure and characterization data for warhead-bearing azides…………………………………4

General procedure and characterization data for alkynes……………………………………………….5

General procedure and characterization data for protected and deprotected triazoles ……………………21

***In situ* click chemistry data…………………………………………………………………...31**

Multicomponent in situ click chemistry KPC-2…………………………………………………...31

Optimisation on AmpC……………………………………………………………………………34

Control experiment in presence of ***SM23***…………………………………………………………….35

Multicomponent in situ click chemistry AmpC………………………………………………………..36

DogSite Scorer analysis…………………………………………………………………….40

Summary for β-lactamases binding pockets……………………………………………………….40

Figures for Class A………………………………………………………………………………...41

Figures for Class B………………………………………………………………………………...41

Figures for Class C……………………………………………………………………………...…42

Figures for Class D………………………………………………………………………………...42

References…………………………………………………………………………………43

NMR Spectra……………………………………………………………………………...46

NMR spectra for azides ……………………………………………………………………………46

NMR spectra for alkynes ………………………………………………………………………........48

NMR spectra for triazoles and starting materials………………………………………………...72

Preparation of starting materials and products

*Degradation pathway for warhead-bearing azide **1***

Azido warhead bearing **1** showed a partial degradation to 2-(thiophen-2-yl)-N-vinylacetamide in presence of phosphate buffers (NaP_i, KP_i, PBS). Those observations were confirmed by ^{1}H, ^{13}C, ^{11}B NMR analysis using $PBS_{(D2O)}$:MeOD 95:5 over a course of 30 h (**Figure S1**). Replacement of phosphate buffers with water confirmed compound **1** do not undergo any chemical modification in the same period (**Figure S2**).

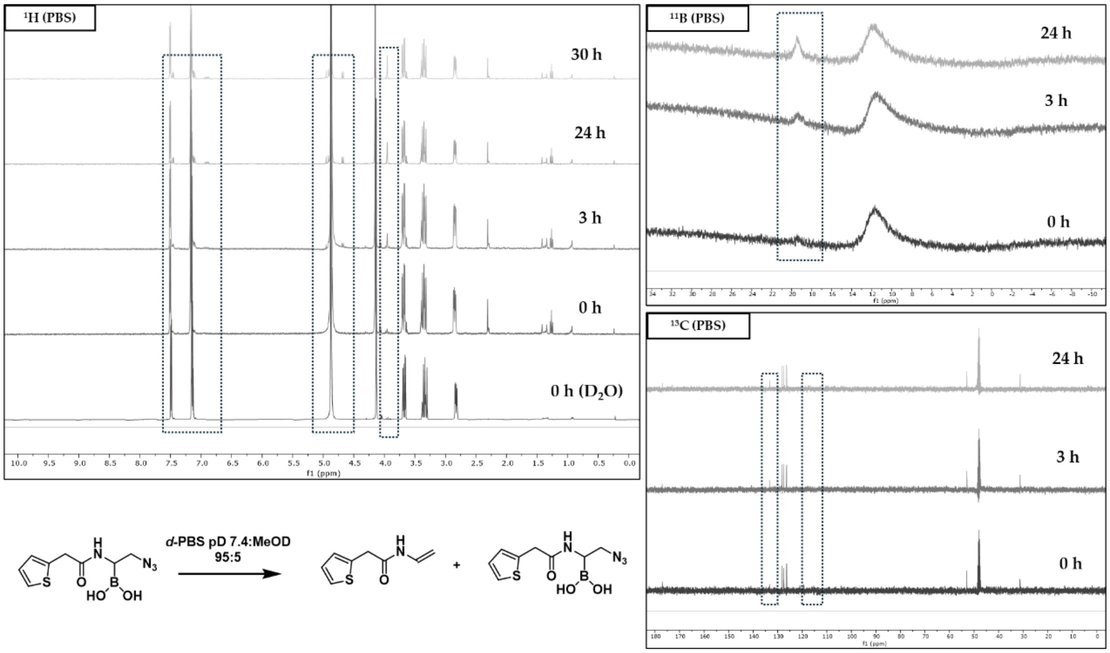

Figure S1. Timeline of degradation of **1** into 2-(thiophen-2-yl)-*N*-vinylacetamide in presence of phosphate buffers. **Top left**: ^{1}H NMR analysis of **1** over the course of 30 h in PBS_{D2O}:MeOH 95:5. **Top right**: ^{11}B NMR analysis of **1** over the course of 24 h in PBS_{D2O}:MeOH 95:5. **Bottom right**: ^{13}C NMR analysis of **1** over the course of 24 h in PBS_{D2O}:MeOH 95:5.

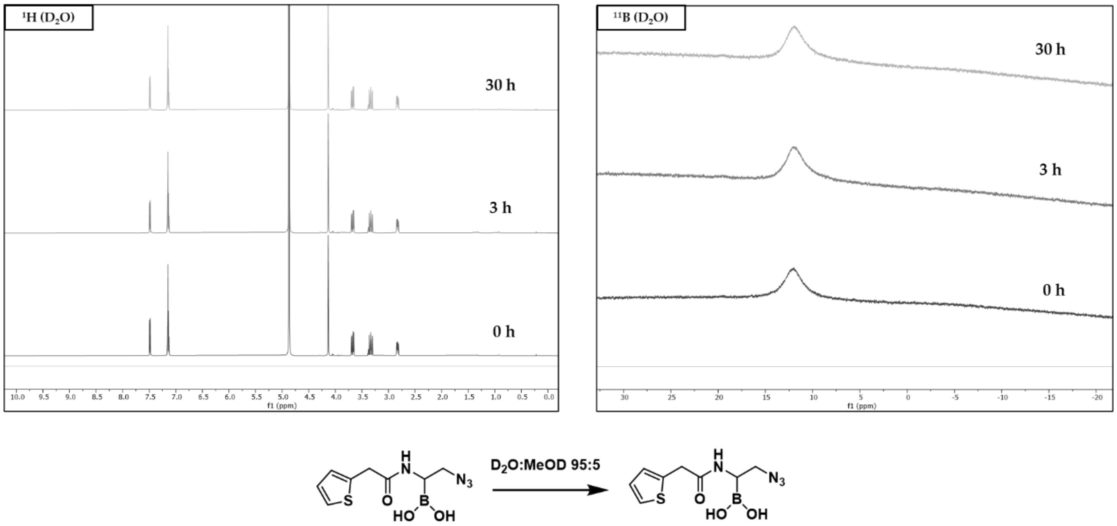

Figure S2. ^{1}H NMR (left) and ^{11}B NMR (right) analysis of **1** over the course of 30 h in D_2O:MeOH 95:5. No degradation observed.

General procedure and characterization data for warhead-bearing azides

Method A

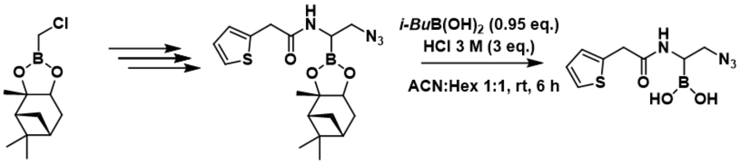

Method B

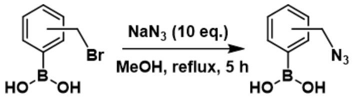

General procedure 1, Method A: Starting material *N*-((*R*)-2-azido-1-((3a*R*,4*S*,6*S*,7a*R*)-3a,5,5-trimethylhexahydro-4,6-methanobenzo[*d*][1,3,2]dioxaborol-2-yl)ethyl)-2-(thiophen-2-yl)acetamide was obtained following previously reported procedure.[1] The protected azide (1.0 eq.) was dissolved in acetonitrile (CH_3CN, 7 mL), followed by addition of isobutyl boronic acid (0.95 eq.) and HCl 3 M (3.0 eq.). After few minutes, hexane (Hex, 7 mL) was added and the mixture stirred vigorously for 20 mins. Successively, hexane was removed, followed by addition of new hexane. The mixture was stirred for another 30 mins. Removal and addition of hexane was repeated until disappearance of the isobutyl boronic acid pinanediol spot on TLC. Upon completion, the acetonitrile phase was dried under reduced pressure. The resulting product was washed with diethyl ether (Et_2O, 3 x 10 mL). Furthermore, trituration with petroleum ether (EtOPet, 3 x 10 mL) was performed to afford the product.

General procedure 1, Method B: In a round-bottom flask *o*-, *m*- or *p*-(bromomethyl)phenylboronic acid (1.0 eq.) was added to a stirred solution of methanol (MeOH, 10 mL). Sodium azide (10.0 eq.) was added portion-wise and the mixture stirred at 73°C for 5 h. The mixture was cooled down to room temperature and diluted with Et_2O (25 mL). The precipitate was filtered down and washed 3 times with Et_2O to remove sodium azide. The filtrate obtained was evaporated under reduced pressure and recrystallysed from Dichloromethane:Pentane (DCM:Pen) to afford the corresponding azide.

2-azido-1-(2-(thiophen-2-yl)acetamido)ethyl)boronic acid (**1**)

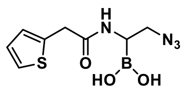

Synthesis according to the **general procedure 1, method A** to afford a brown solid (624 mg, 97% yield). **^{1}H NMR** (600 MHz, DMSO) δ: 9.52 (q, J = 5.2 Hz, 1H), 7.41 – 7.38 (m, 1H), 6.96 (d, J = 3.5 Hz, 2H), 4.72 (s, 2H), 3.87 (d, J = 2.1 Hz, 2H), 3.45 (dd, J = 12.9 and 3.9 Hz, 1H), 3.10 (dd, J = 12.9 and 9.5 Hz, 1H), 2.64 (dt, J = 9.4 and 3.5 Hz, 1H). **^{13}C NMR** (151 MHz, DMSO-*d*$_6$) δ: 173.74, 135.24, 126.95, 126.86, 125.51, 53.52, 32.38. **^{11}B NMR** (193 MHz, DMSO-*d*$_6$) δ: 11.95. **HRMS (ESI) m/z**: $[M-H]^-$ calcd. for 253.0573 found $[M-H]^-$: 253.0571.

(2-(azidomethyl)phenyl)boronic acid (**2**)

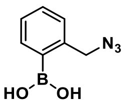

Synthesis according to the **general procedure 1, method B** to afford a white solid (130 mg, 63% yield). **^{1}H NMR** (400 MHz, D_2O) δ: 7.42-7.37 (m, 1H), 7.36-7.25 (m, 3H), 4.46 (s, 2H). **HRMS (ESI) m/z**: [M-H+HCOO]$^-$: calcd. for 222.0687 found [M-H+ HCOO]$^-$: 222.0692. Characterization data in agreement with literature.[2]

(3-(azidomethyl)phenyl)boronic acid (**3**)

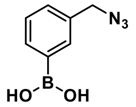

Synthesis according to the **general procedure 1, method B** to afford a white solid (173 mg, 84% yield). **^{1}H NMR** (400 MHz, MeOD) δ: 7.70-7.50 (m, 2H), 7.34-7.26 (m, 2H), 4.37 (s, 2H). **HRMS (ESI) m/z**: [M-H+HCOO]$^-$: calcd. for 222.0686 found [M-H+HCOO]$^-$: 222.0692. Characterization data in agreement with literature.[3]

(4-(azidomethyl)phenyl)boronic acid (**4**)

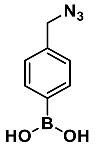

Synthesis according to the **general procedure 1, method B** to afford a white solid (120 mg, 68% yield). **^{1}H NMR** (400 MHz, D_2O) δ: 7.87 (d, J = 7.7 Hz, 2H), 7.53 (d, J = 7.7 Hz, 2H), 4.53 (s, 2H). **HRMS (ESI) m/z**: [M-H+HCOO]$^-$: calcd. for 222.0686 found [M-H+HCOO]$^-$: 222.0692. Characterization data in agreement with literature.[3]

General procedure and characterization data for alkynes

Alkynes **Al-5**, **Al-6**, **Al-7**, **Al-8**, **Al-9**, **Al-15**, **Al-16**, **Al-17**, **Al-18**, **Al-24**, **Al-25**, **Al-26**, **Al-27**, **Al-29**, **Al-31**, **Al-33**, **Al-34**, **Al-35**, **Al-36**, **Al-43**, **Al-44**, **Al-48**, **Al-50**, **Al-51**, **Al-52**, **Al-53**, **Al-54**, **Al-61**, **Al-62**, **Al-63**, **Al-69**, **Al-70**, **Al-71**, **Al-72**, **Al-75**, **Al-78**, **Al-79**, **Al-80**, **Al-86**, **Al-87**, **Al-89** and **Al-90** were purchased from commercial sources. The other alkynes employed in this work were prepared following the standard procedure described below unless otherwise indicated.

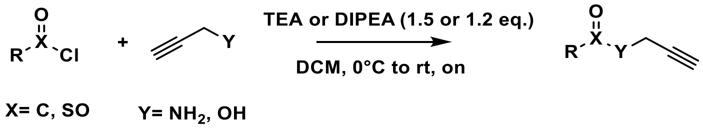

General procedure 2: In a round-bottom flask propargyl amine or propargyl alcohol (1.0 eq.) and triethylamine (TEA, 1.5 eq.) or *N,N*-Diisopropylethylamine (DIPEA, 1.2 eq.) were added to a stirred solution of dichloromethane (DCM, 5 mL) at 0 °C. After 15 mins, the acyl chloride or the sulfonyl chloride (1.20 eq.) was added dropwise or portion-wise. The reaction was stirred at 0 °C for 15 mins, then overnight at rt. Once disappearance of the starting materials was confirmed by TLC, the solution was further diluted with DCM (5 mL). The organic layer (10 mL) was washed with: 1) HCl 1 M (5 mL); 2) H_2O (5 mL); 3) Brine (5 mL). Successively, the organic layer was dried over a $MgSO_4$ bed and evaporated under reduced pressure to afford the respective alkyne.

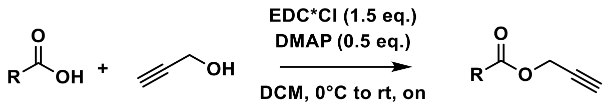

General procedure 3: In a round-bottom flask propargyl alcohol (1.5 eq.) was added to a stirred solution of DCM (5 mL) at 0 °C. After 15 mins, the carboxylic acid (1.0 eq.) and 4-Dimethylaminopyridine (DMAP, 0.5) were added dropwise or portion-wise. Successively, 1-Ethyl-3-(3-dimethylaminopropyl)carbodiimide chloridrate (EDC*Cl, 1.5 eq.) was added. The reaction was allowed to warm up to room temperature and stirred overnight. Once disappearance of the starting materials was confirmed by TLC, the solution was further diluted with DCM (5 mL) and HCl 1 M (5 mL). The organic layer (10 mL) was washed with: 1) 10% $NaHCO_3$ (5 mL); 2) H_2O (5 mL); 3) Brine (5 mL). Successively, the organic layer was dried over a $MgSO_4$ bed and evaporated under reduced pressure to afford the respective alkyne.

Step 1

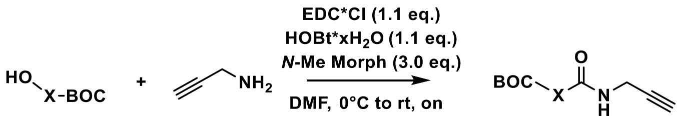

X= Val, Gly

Step 2

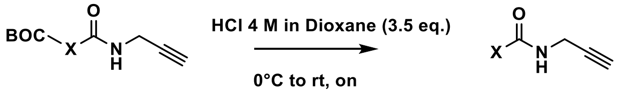

X= Val, Gly

General procedure 4:

Step 1. In a round-bottom flask the BOC-amino acid-OH derivative (Valine or Glycine, 1.0 eq.) was added to a stirred solution of *N*,*N*-Dimethylformamide (DMF, 3 mL) at 0 °C. Next, 1*H*-1,2,3-Benzotriazol-1-ol (HOBT, 1.1 eq.), EDC*Cl (1.1 eq.) and *N*-methyl morpholine (N-Me Morph, 3.0 eq.) were added sequentially. After 10 mins, propargylamine was added and the solution was stirred at 0 °C for 4 hours. Successively, the mixture was left stirring overnight at rt. After this time, the mixture was diluted with EtOAc (10 mL) and HCl 1 M (10 mL). The aqueous layer was extracted twice with EtOAc (10 mL). The combined organic layers (30 mL) were washed with: 1) 10% $NaHCO_3$ (10 mL); 2) H_2O (10 mL); 3) Brine (10 mL). Successively, the organic layer was dried over a $MgSO_4$ bed and evaporated under reduced pressure. The crude mixture was purified by recrystallisation using a DCM:Hex mixture to afford the protected alkyne.

Step 2. In a round-bottom flask containing the BOC-protected alkyne (1.0 eq.) was added HCl 4 M in dioxane (5 mL, 3.5 eq.) at 0 °C. Next, the solution was stirred at 0 °C for 4 hours. Successively, the mixture was left stirring overnight at rt. After this time, the mixture was evaporated under reduced pressure. The crude product was washed with Et_2O and purified by recrystallisation using *iso*-propanol to afford the desired alkyne.

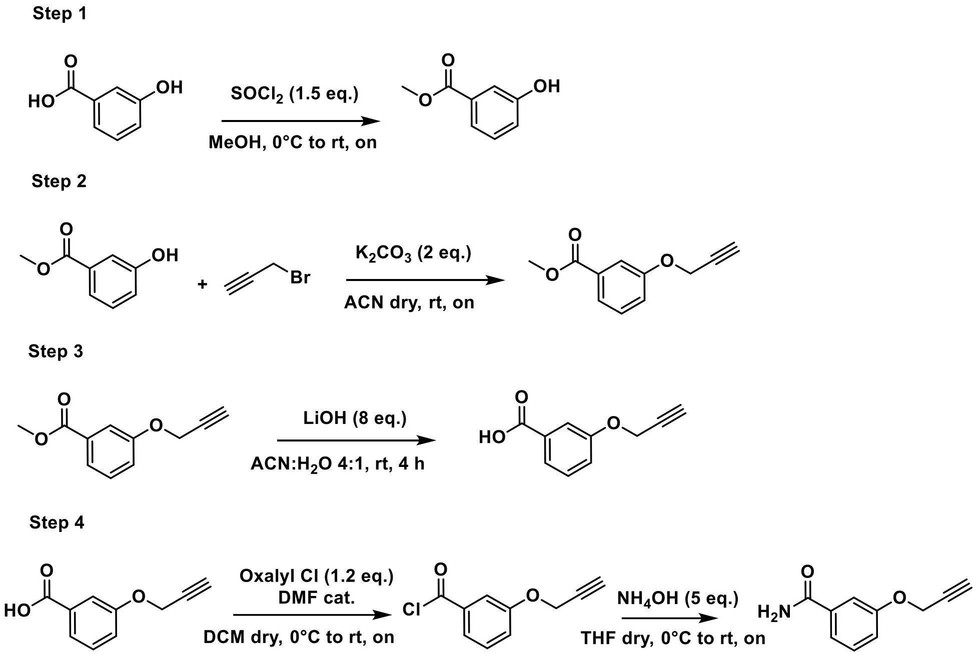

General procedure 5:

Step 1. In a round-bottom flask 3-hydroxy benzoic acid (1.0 eq.) was added to a stirred solution of MeOH (10 mL) at 0 °C. Next, thionyl chloride ($SOCl_2$, 1.5 eq.) was added. The mixture was left stirring overnight at rt. After this time, both methanol and thionyl chloride were evaporated under reduced pressure to afford methyl 3-hydroxybenzoate (1.10 g, 99%).

Step 2. In a round-bottom flask flushed with argon, methyl 3-hydroxybenzoate (1.0 eq.) was added to a stirred solution of dry ACN (10 mL) at 0 °C. This step was followed by addition of potassium carbonate (K_2CO_3, 2 eq.). Next, propargyl bromide (1.5 eq.) was added dropwise and the mixture was left stirring overnight at rt. After this time, H_2O was added to the mixture and the resulting solution extracted three times with EtOAc (3x10 mL). Successively, the combined organic layers were dried over a $MgSO_4$ bed and evaporated under reduced pressure to afford methyl 3-(prop-2-yn-1-yloxy)benzoate (600 mg, 96%).

Step 3. In a round-bottom flask methyl 3-(prop-2-yn-1-yloxy)benzoate (1.0 eq.) was added to a stirred solution of ACN:H_2O 4:1 (5 mL). Next, lithium hydroxide (LiOH, 8.0 eq.) was added and the mixture was left stirring for 4 hours at rt. After this time, the base was neutralised by addition of HCl 3 M and the mixture filtrated in a Buchner funnel. The residual solution was evaporated under reduced pressure to afford **Al- 41** as white powder (500 mg, 92%).

Step 4. In a round-bottom flask a solution of **Al-41** (1.0 eq.) in DCM dry (5 mL) was stirred at 0 °C for 10 minutes. Successively, few drops of DMF (catalytic amount) and oxalyl chloride (1.2 eq.) were added to the solution. The mixture was left stirring at rt overnight. Next, the solution was evaporated under reduced pressure and flushed with argon before addition of THF dry (5 mL). Successively, the solution was cooled to 0 °C and ammonium hydroxide (NH_4OH, 5.0 eq.) was added. The mixture was left stirring at rt overnight. The mixture was diluted with acetone (5 mL) and washed three times with H_2O (3x5 mL) and brine (5 mL). Successively, the organic layer was dried over a $MgSO_4$ bed and evaporated under reduced pressure to afford **Al-32** (300 mg, 99%).

N-(prop-2-yn-1-yl)isobutyramide (**Al-1**)

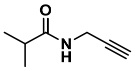

Synthesis according to the **general procedure 2**. Product was obtained without further purification as a beige powder (492 mg, 65%). **^{1}H NMR** (400 MHz, $CDCl_3$) δ**:** 5.61 (br, 1H), 4.05 (dd, J = 5.2 and 2.6 Hz, 2H), 2.37 (hept, J = 6.9 Hz, 1H), 2.23 (t, J = 2.6 Hz, 1H), 1.17 (d, J = 6.9 Hz, 6H). **HRMS (ESI) m/z**: $[M+H]^+$ calcd. 126.0913 found $[M+H]^+$: 126.0914. Characterization data in agreement with literature. [4]

N-2-Propyn-1-ylpentanamide (**Al-2**)

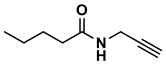

Synthesis according to the **general procedure 2**. The product was obtained without further purification as an orange powder (80 mg, 35%). **^{1}H NMR** (400 MHz, $CDCl_3$) δ**:** 5.61 (br, 1H), 4.05 (dd, J = 5.2 and 2.6 Hz, 2H), 2.22 (d, J = 2.5 Hz, 1H), 2.22 – 2.17 (m, 2H), 1.68 – 1.58 (m, 2H), 1.35 (d, J = 7.6 Hz, 2H), 0.92 (s, 3H). **HRMS (ESI) m/z**: $[M+H]^+$ calcd. 140.1069 found $[M+H]^+$: 140.1069. Characterization data in agreement with literature.[4]

N-2-Propyn-1-ylmethanesulfonamide (**Al-3**)

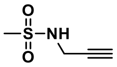

Synthesis according to the **general procedure 2**. The product was obtained without further purification as a beige powder (400 mg, 56%). **^{1}H NMR** (400 MHz, $CDCl_3$) δ**:** 4.59 (br, 1H), 3.98 (dd, J = 6.2 and 2.5 Hz, 2H), 3.09 (s, 3H), 2.38 (t, J = 2.5 Hz, 1H). **HRMS (ESI) m/z**: $[M+H]^+$ calcd. 134.0989 found $[M+H]^+$: Not visible. Characterization data in agreement with literature.[5]

N-(prop-2-yn-1-yl)propane-2-sulfonamide (**Al-4**)

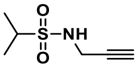

Synthesis according to the **general procedure 2**. The crude mixture was purified by flash chromatography using a 95:5 DCM:MeOH mixture. The product was obtained as an orange powder (56 mg, 22%). **^{1}H NMR** (400 MHz, $CDCl_3$) δ**:** 4.41 (br, 1H), 3.96 (dd, J = 6.1 and 2.5 Hz, 2H), 3.30 (hept, J = 6.8 Hz, 1H), 2.34 (t, J = 2.5 Hz, 1H), 1.41 (d, J = 6.8 Hz, 6H). **HRMS (ESI) m/z**: $[M+H]^+$ calcd. 162.0582 found $[M+H]^+$: 162.0583. Characterization data in agreement with literature.[4]

3-cyclohexyl-*N*-(prop-2-yn-1-yl)propanamide (**Al-10**)

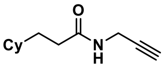

Synthesis according to the **general procedure 2**. The crude mixture was purified by recrystallisation using a DCM:Hex mixture. The product was obtained as a yellowish powder (251 mg, 79%). **^{1}H NMR** (400 MHz, $CDCl_3$) δ**:** 5.57 (br, 1H), 4.05 (dd, J = 5.2 and 2.6 Hz, 2H), 2.26 – 2.14 (m, 3H), 1.74 – 1.63 (m, 5H), 1.56 – 1.50 (m, 2H), 1.27 – 1.11 (m, 4H), 0.95 – 0.84 (m, 2H). **HRMS (ESI) m/z**: $[M+H]^+$ calcd. 194.1539 found $[M+H]^+$: 194.1539. Characterization data in agreement with literature.[4]

N-(prop-2-yn-1-yl)cyclohexanecarboxamide (**Al-11**)

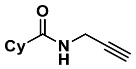

Synthesis according to the **general procedure 2**. The product was obtained without further purification as white crystals (130 mg, 48%). **^{1}H NMR** (400 MHz, $CDCl_3$) δ**:** 5.65 (br, 1H), 4.04 (dd, J = 5.2 and 2.6 Hz, 2H), 2.22 (t, J = 2.6 Hz, 1H), 2.14-2.06 (m, 1H), 1.91 – 1.76 (m, 4H), 1.70 – 1.62 (m, 1H), 1.46-1.41 (m, 2H), 1.30 – 1.12 (m, 3H). **HRMS (ESI) m/z**: $[M+H]^+$ calcd. 166.1226 found $[M+H]^+$: 166.1226. Characterization data in agreement with literature.[4]

N-(prop-2-yn-1-yl)cyclohexanesulfonamide (**Al-12**)

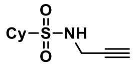

Synthesis according to the **general procedure 2**. The crude mixture was purified by flash chromatography using a 75:25 EtOPet:EtOAc mixture. The product was obtained as a yellow oil (75 mg, 73%). **^{1}H NMR** (400 MHz, $CDCl_3$) δ**:** 4.33 (br, 1H), 3.95 (dd, J = 6.2 and 2.5 Hz, 2H), 3.01 (tt, J = 12.1 and 3.4 Hz, 1H), 2.34 (t, J = 2.5 Hz, 1H), 2.25-2.18 (m, 2H), 1.91 (dt, J = 13.2 and 3.3 Hz, 2H), 1.76-1.68 (m, 1H), 1.60 – 1.52 (m, 2H), 1.33 – 1.19 (m, 3H). **HRMS (ESI) m/z**: $[M+H]^+$ calcd. 202.0895 found $[M+H]^+$: 202.0895. Characterization data in agreement with literature.[4]

N-(prop-2-yn-1-yl)cyclopropanesulfonamide (**Al-13**)

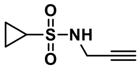

Synthesis according to the **general procedure 2**. The product was obtained without further purification as a yellowish powder (60 mg, 21%). **^{1}H NMR** (600 MHz, $CDCl_3$) δ**:** 4.58 (br, 1H), 3.96 (dd, J = 5.9 and 2.5 Hz, 2H), 2.55 (tt, J = 8.0 and 4.8 Hz, 1H), 2.35 (t, J = 2.5 Hz, 1H), 1.30 – 1.19 (m, 2H), 1.09 – 0.97 (m, 2H). **HRMS (ESI) m/z**: $[M+H]^+$ calcd. 160.0426 found $[M+H]^+$: 160.0427. Characterization data in agreement with literature.[4]

prop-2-yn-1-yl cyclohexanecarboxylate (**Al-14**)

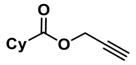

Synthesis according to the **general procedure 3**. The product was obtained without further purification as a yellow oil (109 mg, 37%). **^{1}H NMR** (600 MHz, $CDCl_3$) δ**:** 4.66 (d, J = 2.4 Hz, 2H), 2.45 (t, J = 2.5 Hz, 1H), 2.35 (tt, J = 11.3 and 3.6 Hz, 1H), 1.96 – 1.88 (m, 2H), 1.75 (dt, J = 12.8 and 3.6 Hz, 2H), 1.68 – 1.62 (m, 1H), 1.50-1.46 (m, 2H), 1.32 – 1.20 (m, 3H). **HRMS (ESI) m/z**: $[M+H]^+$ calcd. 167.1066 found $[M+H]^+$: 167.1069. Characterization data in agreement with literature.[6]

N-(prop-2-yn-1-yl)thiophene-2-carboxamide (**Al-19**)

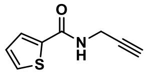

Synthesis according to the **general procedure 2**. The product was obtained without further purification as a white powder (110 mg, 40%). **^{1}H NMR** (400 MHz, $CDCl_3$) δ**:** 7.52 (dd, J = 4.4 and 1.2 Hz, 2H), 7.08 (dd, J =

5.0 and 3.7 Hz, 1H), 6.32 (s, 1H), 4.23 (dd, J = 5.3 and 2.6 Hz, 2H), 2.28 (t, J = 2.5 Hz, 1H). **HRMS (ESI) m/z**: $[M+H]^+$ calcd. 166.0320 found $[M+H]^+$: 166.0322. Characterization data in agreement with literature.[4]

N-(prop-2-yn-1-yl)furan-2-carboxamide (**Al-20**)

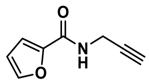

Synthesis according to the **general procedure 2**. The crude mixture was purified by recrystallisation using a DCM:Hex mixture. The product was obtained as a brownish powder (55 mg, 20%). **^{1}H NMR** (400 MHz, $CDCl_3$) δ**:** 7.45 (d, J = 1.8 Hz, 1H), 7.15 (d, J = 3.5 Hz, 1H), 6.50 (dd, J = 3.5 and 1.7 Hz, 1H), 4.22 (dd, J = 5.4 and 2.5 Hz, 2H), 2.27 (t, J = 2.5 Hz, 1H). **HRMS (ESI) m/z**: $[M+H]^+$ calcd. 150.0549 found $[M+H]^+$: 150.0550. Characterization data in agreement with literature.[4]

N-(prop-2-yn-1-yl)benzo[c][1,2,5]oxadiazole-4-sulfonamide (**Al-21**)

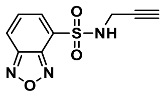

Synthesis according to the **general procedure 2**. The crude mixture was purified by flash chromatography using a 50:50 EtOPet:EtOAc mixture. The product was obtained as an orange powder (160 mg, 41%). **^{1}H NMR** (400 MHz, $CDCl_3$) δ**:** 8.13 – 8.06 (m, 2H), 7.57 (dd, J = 9.0 and 6.8 Hz, 1H), 5.40 (br, 1H), 3.99 (dd, J = 6.3 and 2.5 Hz, 2H), 1.79 (t, J = 2.5 Hz, 1H). **HRMS (ESI) m/z**: $[M+H]^+$ calcd. 238.0280 found $[M+H]^+$: 238.0280. Characterization data in agreement with literature.[4]

N-(prop-2-yn-1-yl)thiophene-2-sulfonamide (**Al-22**)

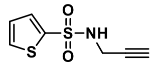

Synthesis according to the **general procedure 2**. The crude mixture was purified by flash chromatography using a 70:30 EtOPet:EtOAc mixture. The product was obtained as a white-pink powder (122 mg, 33%). **^{1}H NMR** (400 MHz, $CDCl_3$) δ**:** 7.68 – 7.60 (m, 2H), 7.11 (dd, J = 5.0 and 3.8 Hz, 1H), 4.66 (br, 1H), 3.92 (dd, J = 6.1 and 2.6 Hz, 2H), 2.15 (t, J = 2.5 Hz, 1H). **HRMS (ESI) m/z**: $[M+H]^+$ calcd. 201.9990 found $[M+H]^+$: 201.9990. Characterization data in agreement with literature.[4]

prop-2-yn-1-yl thiophene-2-carboxylate (**Al-23**)

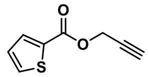

Synthesis according to the **general procedure 2**. The crude mixture was purified by flash chromatography using a 90:10 EtOPet:EtOAc mixture. The product was obtained as a yellow oil (373 mg, 66%). **^{1}H NMR** (400 MHz, $CDCl_3$) δ**:** 7.86 (dd, J = 3.8 and 1.3 Hz, 1H), 7.60 (dd, J = 5.0 and 1.3 Hz, 1H), 7.12 (dd, J = 5.0 and 3.8 Hz, 1H), 4.90 (d, J = 2.5 Hz, 2H), 2.52 (t, J = 2.5 Hz, 1H). **HRMS (ESI) m/z**: $[M+H]^+$ calcd. 167.0161 found $[M+H]^+$: 167.0165. Characterization data in agreement with literature.[7]

2-amino-*N*-(prop-2-yn-1-yl)acetamide (**Al-28**)

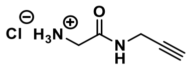

Synthesis according to the **general procedure 4, step 1 and 2**. The product was obtained as a white powder (55 mg, 30%). **^{1}H NMR** (400 MHz, MeOD) δ**:** 4.05 (d, J = 2.6 Hz, 2H), 3.67 (s, 2H), 2.66 (t, J = 2.6 Hz, 1H). **HRMS (ESI) m/z**: $[M\text{-}Cl]^+$ calcd. 113.0709 found $[M\text{-}Cl]^+$: 113.0712. Characterization data in agreement with literature.[8]

4-(prop-2-yn-1-yl)morpholine (**Al-30**)

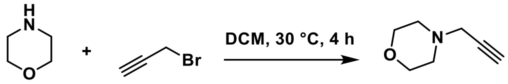

In a round-bottom flask morpholine (2.5 eq.) was added to a stirred solution of DCM (5 mL). Propargyl bromide (1.0 eq.) was added dropwise and the reaction stirred at 30 °C for 4 hours. Once disappearance of the starting materials was confirmed by TLC, the solution was cooled to rt and further diluted with DCM (5 mL). The organic layer (10 mL) was washed with: 1) H_2O (2x5 mL); 2) Brine (5 mL). Successively, the organic layer was dried over a $MgSO_4$ bed and evaporated under reduced pressure to afford an orange oil (60 mg, 25%). **^{1}H NMR** (400 MHz, $CDCl_3$) δ**:** 3.76 – 3.72 (m, 4H), 3.30 (d, J = 2.5 Hz, 2H), 2.58 (dd, J = 5.6 and 3.8 Hz, 4H), 2.27 (t, J = 2.4 Hz, 1H). **HRMS (ESI) m/z**: $[M+H]^+$ calcd. 126.0913 found $[M+H]^+$: 126.0913. Characterization data in agreement with literature.[9]

3-(prop-2-yn-1-yloxy)benzamide (**Al-32**)

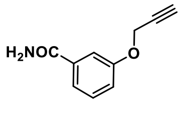

Synthesis according to the **general procedure 4, step 4**. The product was obtained without further purification as a yellowish powder (300 mg, 99%). **^{1}H NMR** (400 MHz, $CDCl_3$) δ**:** 7.49 – 7.45 (m, 1H), 7.42 – 7.35 (m, 2H), 7.15 (dt, J = 6.2 and 2.7 Hz, 1H), 5.89 (br, 2H), 4.75 (d, J = 2.4 Hz, 2H), 2.54 (t, J = 2.4 Hz, 1H). **HRMS (ESI) m/z**: $[M+H]^+$ calcd. 176.0705 found $[M+H]^+$: 176.0705. Characterization data in agreement with literature.[10]

4-oxo-4-(prop-2-yn-1-ylamino)butanoic acid (**Al-37**)

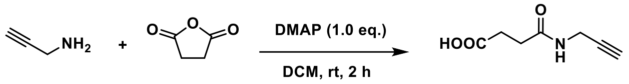

In a round bottom flask DMAP (1.0 eq.) was added to a mixture of propargyl amine (1.0 eq.) and succinic anhydride (1.2 eq.) in DCM (5.0 mL). The reaction was stirred at rt for 2 h. Successively, the organic layer was washed with a solution of 5% Na_2CO_3 (5 mL). The aqueous layer was acidified with a HCl 1 M and extract with ethyl acetate (3x10 mL). The combined organic layers were dried over a $MgSO_4$ bed, filtered and evaporate under reduced pressure. The residue was washed with Et_2O to afford a yellowish powder (30 mg, 25%). **^{1}H NMR** (400 MHz, MeOD) δ**:** 3.95 (d, J = 2.5 Hz, 2H), 2.63 – 2.54 (m, 3H), 2.48 (t, J = 6.8 Hz, 2H). **HRMS (ESI) m/z**: $[M\text{-}H]^-$ calcd. 154.0510 found $[M\text{-}H]^-$: 154.0500. Characterization data in agreement with literature.[11]

tert-butyl (*S*)-but-3-yn-2-ylcarbamate (**Al-38**)

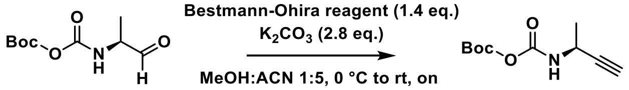

In a 3-neck round bottom flask, Bestmann-Ohira reagent (1.4 eq.) was added to a stirring solution of ACN (15 mL) at 0 °C. Next, MeOH (5 mL) and potassium carbonate (2.8 eq.) were added, followed by addition of BOC-Ala-CO (1.0 eq.). The reaction was allowed to warm up and stirred at rt overnight. Successively, EtOAc (20 mL) was added and the aqueous phase extracted twice (2x20 mL). The combined organic layers were washed with brine and dried over a $MgSO_4$ bed, filtered and evaporate under reduced pressure. The crude mixture was purified by flash chromatography using a 90:10 EtOPet:EtOAc mixture. The product was obtained as a yellow powder (273 mg, 92%). **^{1}H NMR** (400 MHz, $CDCl_3$) δ: 4.48 (br, 1H), 2.25 (d, J = 2.2 Hz, 1H), 1.45 (s, 9H), 1.40 (d, J = 6.9 Hz, 3H). **HRMS (ESI) m/z**: [M+H-*t*Bu]$^+$ calcd. 114.0549 found [M+H-*t*Bu]$^+$: 114.0555. Characterization data in agreement with literature.[12]

(prop-2-yn-1-ylsulfonyl)benzene (**Al-39**)

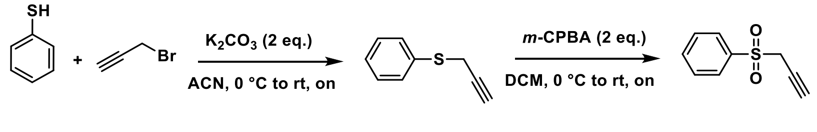

Step 1: In a 2-neck round bottom flask flushed with Ar, thiophenol (1.0 eq.) and potassium carbonate (2.0 eq.) were added to a stirring solution of ACN (10 mL) at 0 °C. Next, propargyl bromide (1.5 eq.) was added dropwise. The reaction was allowed to warm up and stirred at rt overnight. Successively, EtOAc (10 mL) was added. The resulting solid filtrated on a celite plug and washed with EtOAC (2x10 mL). The filtrate was evaporated under reduced pressure and purified by flash chromatography using a 95:5 EtOPet:EtOAc mixture. The resulting product (771 mg, 97%), phenyl(prop-2-yn-1-yl)sulfane was used for the next step.

Step 2: Phenyl(prop-2-yn-1-yl)sulfane (300 mg, 1.0 eq.) was added to a round bottom flask containing a stirring solution of DCM (10 mL) at 0 °C. *meta*-chloroperoxybenzoic acid (*m*-CPBA, 2.0 eq.) was added portion-wise. The reaction was allowed to warm up and stirred at rt overnight. The organic phase was washed three times with a solution of 5% Na_2CO_3 (3x5 mL). The organic layer was dried over a $MgSO_4$ bed, filtered and evaporate under reduced pressure. The product was obtained as a yellowish powder (353 mg, 97%). **^{1}H NMR** (400 MHz, $CDCl_3$) δ: 8.01 – 7.98 (m, 2H), 7.74 – 7.67 (m, 1H), 7.62 – 7.58 (m, 2H), 3.97 (d, J = 2.7 Hz, 2H), 2.37 (d, J = 5.4 Hz, 1H). **HRMS (ESI) m/z**: [M+H]$^+$ calcd. 181.0317 found [M+H]$^+$: 181.0319. Characterization data in agreement with literature.[13]

tert-butyl (*S*)-(1-phenylbut-3-yn-2-yl)carbamate (**Al-40**)

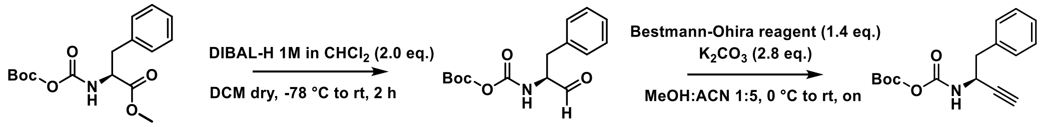

Step 1: In a 3-neck round bottom flask flushed with Ar cooled down to -78 °C, a solution of Boc-(*S*)-phenylalanine methyl ester (1.0 eq.) in anhydrous DCM is added. Diisobutylaluminium hydride (DIBAL-H, 2.0 eq.) is slowly added dropwise, and the reaction is allowed to stir at -78 °C for 75 mins. The mixture is treated at -78 °C with anhydrous MeOH (0.5 mL) to remove excess of DIBAL-H. After 10 mins, the reaction is allowed to warm up at room temperature and stirred for another 15 mins. At this stage, DCM (25 mL), H_2O (15 mL) and HCl 0.5 M (2.5 mL) are added to the mixture. The resulting gelatine is centrifuged resulting in an organic phase, an aqueous phase and a white solid. The aqueous phase is extracted with DCM (30 mL) and the organic phase combined with the one obtained from the centrifugation. The combined organic layers were washed with H_2O (20 mL), brine (10 mL), over a $MgSO_4$ bed, filtered and

evaporate under reduced pressure. The crude mixture was purified by flash chromatography (EtOPet:EtOAc 8:2) to afford a white solid (1.5 g, 84%).

Step 2: In a 3-neck round bottom flask, Bestmann-Ohira reagent (1.4 eq.) was added to a stirring solution of ACN (15 mL) at 0 °C. Next, MeOH (5 mL) and potassium carbonate (2.8 eq.) were added, followed by addition of *tert*-butyl (*S*)-(1-oxo-3-phenylpropan-2-yl)carbamate (1.0 eq.). The reaction was allowed to warm up and stirred at rt overnight. Successively, EtOAc (20 mL) was added and the aqueous phase extracted twice (2x20 mL). The combined organic layers were washed with brine and dried over a $MgSO_4$ bed, filtered and evaporate under reduced pressure. The product was obtained as a white solid (395 mg, 98%). ^{1}**H NMR** (400 MHz, $CDCl_3$) δ: 7.35 – 7.25 (m, 5H), 4.70-4.65 (m, 2H), 3.01 – 2.88 (m, 2H), 2.28 (t, J = 1.1 Hz, 1H), 1.43 (s, 9H). **HRMS (ESI) m/z**: $[M-C_4H_8]^+$ calcd. 190.0862 found $[M-C_4H_8]^+$: 190.0865. Characterization data in agreement with literature.[14]

3-(prop-2-yn-1-yloxy)benzoic acid (**Al-41**)

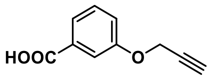

Synthesis according to the **general procedure 4, step 3.** The product was obtained as a white powder (500 mg, 92%). ^{1}**H NMR** (400 MHz, $CDCl_3$) δ: 7.77 (dd, J = 7.7 and 1.5 Hz, 1H), 7.71 (dd, J = 2.7 and 1.5 Hz, 1H), 7.42 (dd, J = 8.1 and 7.6 Hz, 1H), 7.26 – 7.21 (m, 1H), 4.76 (d, J = 2.4 Hz, 2H), 2.55 (t, J = 2.4 Hz, 1H). **HRMS (ESI) m/z**: $[M-H]^-$ calcd. 175.0401 found $[M-H]^-$: 175.0393. Characterization data in agreement with literature.[15]

2-ethynyl-5-(methylthio)benzoic acid (**Al-42**)

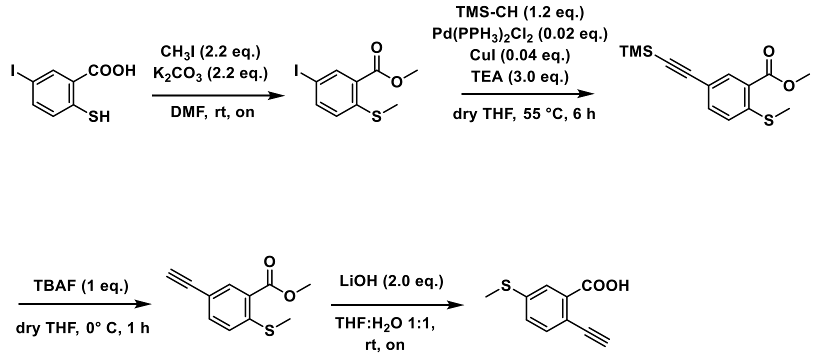

Step 1: 5-iodo-thiosalicylic acid was obtained following previously published procedures.[16] To a stirred suspension of 5-iodo-thiosalicylic acid (1.0 eq.) and K_2CO_3 (4.0 eq.) in anhydrous DMF (15 ml), methyl iodide (MeI, 3.0 eq.) was added under Ar and the reaction mixture stirred at rt overnight. Then, the excess of MeI was quenched with 5% aq. K_2CO_3 solution. After quenching, a yellow solid was formed into the reaction flask, so it was filtered and further purified through flash chromatography using a EtOPet:EtOAc 95:5 to 90:10 mixture to afford a white solid (411 mg, 82%).

Step 2: Methyl 5-iodo-2-(methylthio)benzoate (240 mg, 1.0 eq.), $Pd(PPh_3)_2Cl_2$ (0.02 eq.), and CuI (0.04 eq.) were introduced in a one-necked round-bottom flask, which was evacuated and flushed with argon three times. Dry THF (8 mL) and anhydrous TEA (3.0 eq.) were added via syringe and stirring was continued for 5 min at 55 °C. An orange reaction mixture was formed, which turned yellow upon addition of ethynyltrimethylsilane (1.2 eq.). Stirring was continued 5.5 h at 55 °C, when TLC confirmed the completion

of reaction. The mixture was then cooled to rt, quenched with sat. NH_4Cl and extracted with a 1:1 mixture of EtOAc/Et_2O. The organic layer was washed with brine, and dried with $MgSO_4$ and filtered. The mixture was evaporated under reduced pressure to afford methyl 2-(methylthio)-5-((trimethylsilyl)ethynyl)benzoate as an orange residue. The product was used in the next step without further purification.

Step 3: Methyl 2-(methylthio)-5-((trimethylsilyl)ethynyl)benzoate was dissolved in anhydrous THF, and the mixture cooled to 0 °C. Tetrabutylammonium fluoride (TBAF, 1 M in THF, 1.0 eq.) was added dropwise and the mixture stirred 1 h at 0 °C. Water (5 mL) and EtOAc (5 mL) were added, the two layers separated, and the organic layer was dried with $MgSO_4$ and filtered. Concentration in vacuo produced a black solid which was quickly purified through a short silica pad. The resulting product, methyl 5-ethynyl-2-(methylthio)benzoate, was isolated as an orange solid and used in the following step without further purification.

Step 4: Methyl 5-ethynyl-2-(methylthio)benzoate was dissolved in a 1:1 THF:H_2O (10 mL) mixture and LiOH (2.0 eq.) was added and the mixture stirred overnight. Successively, the mixture was acidified with conc. HCl (pH = 1) and extracted twice with EtOAc. The organic layer was then washed with brine, dried with $MgSO_4$, filtered and evaporated under reduced pressure. The crude product was purified through flash chromatography using a EOPet:Et_2O 40:60 mixture to afford a green solid (108 mg, 72%). **^{1}H NMR** (600 MHz, $CDCl_3$) δ: 8.25 (d, J = 1.9 Hz, 1H), 7.60 (dd, J = 8.4 and 1.9 Hz, 1H), 7.24 (s, 1H), 3.11 (s, 1H), 2.48 (s, 3H). **HRMS (ESI) m/z**: $[M-H]^-$ calcd. 191.0173 found $[M-H]^-$: 191.0165.

4-ethynylphthalic acid (**Al-45**)

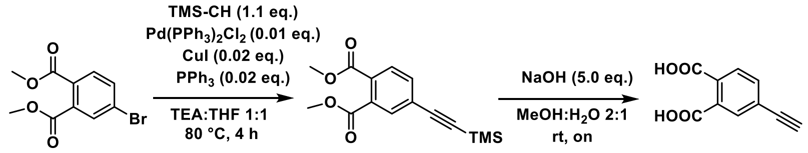

Step 1: $Pd(PPh_3)_2Cl_2$ (0.01 eq.), CuI (0.02 eq.), and triphenyl phosphine (PPh_3, 0.02 eq.) were added to a three necked round-bottomed flask, which was then evacuated and flushed with argon three times. A 1:1 mixture of THF and TEA (80 mL), both anhydrous and degassed, was introduced into the flask, then dimethyl 4-bromophthalate (1.0 eq.) was added as a solution in 5 mL of dry THF. The reaction temperature was raised to 80 °C and ethynyltrimethylsilane (1.2 eq.) was added dropwise. The stirring was continued at 80 °C for 4 hours when TLC showed complete consumption of the starting material. The reaction mixture was then cooled to rt and the solvents removed under reduced pressure. The crude was dissolved in EtOAC, and the organic layer washed with: 1) H_2O; 2) Brine. The organic layer was dried over a $MgSO_4$ bed and concentrated under reduced pressure. The resulting solid was purified by flash chromatography using a mixture EOPet:EtOAc 90:10 to give 2.26 g (79 %) of dimethyl 4-((trimethylsilyl)ethynyl)phthalate as a yellow solid.

Step 2: Dimethyl 4-((trimethylsilyl)ethynyl)phthalate was dissolved in a 2:1 mixture of MeOH/H_2O, then NaOH (5.0 eq.) was added and the mixture allowed to stir at rt overnight. Then, the pH of the solution was carefully lowered to 1 using conc. HCl and the product was extracted twice with EtOAc. The organic phase was dried ($MgSO_4$) and concentrated under reduced pressure to afford an orange solid (1.16 g, 99 %). **^{1}H NMR** (600 MHz, MeOD) δ: 7.77 (d, J = 1.7 Hz, 1H), 7.73 (d, J = 8.0 Hz, 1H), 7.66 – 7.64 (m, 1H), 3.74 (s, 1H). **HRMS (ESI) m/z**: $[M-H]^-$ calcd. 189.0194 found $[M-H]^-$: 189.0186.

2-phenyl-*N*-(prop-2-yn-1-yl)acetamide (**Al-46**)

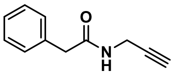

Synthesis according to the **general procedure 2**. The crude mixture was purified by recrystallisation using a DCM:Hex mixture. The product was obtained as a white powder (642 mg, 82%). **^{1}H NMR** (600 MHz, $CDCl_3$) δ**:** 7.37 (t, J = 7.0 Hz, 2H), 7.32 – 7.29 (m, 1H), 7.27 – 7.25 (m, 2H), 5.57 (br, 1H), 4.01 (dd, J = 5.3 and 2.6 Hz, 2H), 3.60 (s, 2H), 2.18 (t, J = 2.6 Hz, 1H). **HRMS (ESI) m/z**: $[M+H]^+$ calcd. 174.0913 found $[M+H]^+$: 174.0913. Characterization data in agreement with literature.[17]

1-phenyl-3-(prop-2-yn-1-yl)urea (**Al-47**)

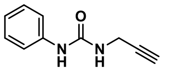

Synthesis according to the **general procedure 2** starting from phenylisocyanate (1.05 eq.). The crude mixture was purified by trituration in Et_2O. The product was obtained as a cream solid (648 mg, 82%). **^{1}H NMR** (600 MHz, $CDCl_3$) δ**:** 7.35 – 7.28 (m, 4H), 7.13 (tt, J = 7.4 and 1.4 Hz, 1H), 6.46 (br, 1H), 4.06 (d, J = 2.5 Hz, 2H), 2.24 (d, J = 5.1 Hz, 1H). **HRMS (ESI) m/z**: $[M+H]^+$ calcd. 175.0865 found $[M+H]^+$: 175.0865. Characterization data in agreement with literature.[18]

1-phenyl-*N*-(prop-2-yn-1-yl)methanesulfonamide (**Al-49**)

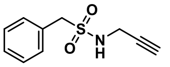

Synthesis according to the **general procedure 2.** The crude mixture was purified by flash chromatography using a mixture EOPet:EtOAc 70:30. The product was obtained as a yellow powder (125 mg, 76%). **^{1}H NMR** (600 MHz, $CDCl_3$) δ**:** 7.49 – 7.46 (m, 2H), 7.39 (dd, J = 5.0 and 2.0 Hz, 3H), 4.40-4.35 (m, 3H), 3.88 (dd, J = 6.0 and 2.5 Hz, 2H), 2.42 (t, J = 2.5 Hz, 1H). **HRMS (ESI) m/z**: $[M+H]^+$ calcd. 210.0582 found $[M+H]^+$: 210.0577. Characterization data in agreement with literature.[19]

4-methyl-*N*-(prop-2-yn-1-yl)benzamide (**Al-55**)

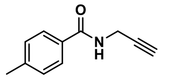

Synthesis according to the **general procedure 2.** The crude mixture was purified by trituration in Et_2O. The product was obtained as a white powder (174 mg, 50%). **^{1}H NMR** (600 MHz, $CDCl_3$) δ**:** 7.68 (dt, J = 8.2 and 1.9 Hz, 2H), 7.25 – 7.22 (m, 2H), 6.28 (br, 1H), 4.25 (dt, J = 4.3 and 2.0 Hz, 2H), 2.39 (s, 3H), 2.27 (q, J = 2.2 Hz, 1H). **HRMS (ESI) m/z**: $[M+H]^+$ calcd. 174.0913 found $[M+H]^+$: 174.0912. Characterization data in agreement with literature.[4]

N-(3-ethynylphenyl)acetamide (**Al-56**)

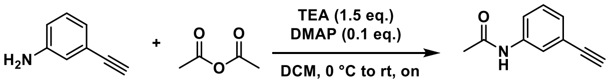

In a round-bottom flask to a solution of propargyl amine (1.0 eq.) in DCM (5 mL) at 0 °C were added sequentially acetic anhydride (1.2 eq.), TEA (1.5 eq.) and DMAP (0.1 eq.). The reaction was stirred at 0 °C for 15 mins, then overnight at rt. Once disappearance of the starting materials was confirmed by TLC, the

solution was further diluted with DCM (5 mL). The organic layer (10 mL) was washed with: 1) 5% $NaHCO_3$ (5 mL); 2) HCl 1 M (5 mL); 3) Brine (5 mL). Successively, the organic layer was dried over a $MgSO_4$ bed and evaporated under reduced pressure. The crude mixture was purified by flash chromatography using a mixture EtOPet:EtOAc 60:40. The product was obtained as a yellowish powder (372 mg, 78%). **^{1}H NMR** (400 MHz, $CDCl_3$) δ**:** 7.61 (t, J = 1.8 Hz, 1H), 7.54 (dt, J = 8.0 and 1.8 Hz, 1H), 7.28 (d, J = 7.7 Hz, 1H), 7.24 (dd, J = 4.1 and 2.5 Hz, 1H), 7.23 – 7.17 (m, 1H), 3.06 (s, 1H), 2.18 (s, 3H). **HRMS (ESI) m/z**: $[M+H]^+$ calcd. 160.0756 found $[M+H]^+$: 160.0755. Characterization data in agreement with literature.[20]

methyl 3-((*N*-(prop-2-yn-1-yl)sulfamoyl)methyl)benzoate (**Al-57**)

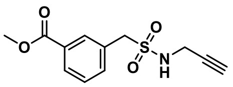

Synthesis according to the **general procedure 2.** The crude mixture was purified by flash chromatography using a mixture EOPet:EtOAc 70:30. The product was obtained as a pale yellow solid (218 mg, 71%). **^{1}H NMR** (600 MHz, $CDCl_3$) δ**:** 8.16 (d, J = 1.8 Hz, 1H), 8.06 (dt, J = 7.8 and 1.5 Hz, 1H), 7.69 (dt, J = 7.6 and 1.5 Hz, 1H), 7.49 (t, J = 7.7 Hz, 1H), 4.42 (s, 2H), 3.95-3.84 (m, J = 6.1 Hz, 5H), 2.46 (q, J = 2.1 Hz, 1H). **HRMS (ESI) m/z**: $[M+H]^+$ calcd. 268.0637 found $[M+H]^+$: 268.0639.

4-cyano-*N*-(prop-2-yn-1-yl)benzenesulfonamide (**Al-58**)

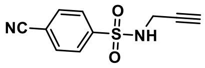

Synthesis according to the **general procedure 2.** The crude mixture was purified by flash chromatography using a mixture EOPet:EtOAc 70:30. The product was obtained as a pale yellow solid (100 mg, 48%). **^{1}H NMR** (600 MHz, $CDCl_3$) δ**:** 8.04 – 8.01 (m, 2H), 7.84 – 7.81 (m, 2H), 4.72 (t, J = 5.9 Hz, 1H), 3.94 (dd, J = 6.1 and 2.5 Hz, 2H), 2.09 (t, J = 2.5 Hz, 1H). **HRMS (ESI) m/z**: $[M-H]^-$ calcd. 219.0234 found $[M-H]^-$: 219.0230. Characterization data in agreement with literature.[21]

1-nitro-3-(prop-2-yn-1-yloxy)benzene (**Al-59**)

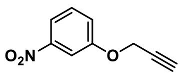

Synthesis according to the **general procedure 5, step 2,** using 3-nitrophenol (1.0 eq.) and propargyl bromide (1.5 eq.) as starting materials**.** The crude mixture was purified by flash chromatography using a mixture EOPet:EtOAc 80:20. The product was obtained as a white solid (253 mg, 92%). **^{1}H NMR** (600 MHz, $CDCl_3$) δ**:** 7.88 (dd, J = 8.2 and 2.1 Hz, 1H), 7.84 (t, J = 2.3 Hz, 1H), 7.46 (t, J = 8.2 Hz, 1H), 7.31 (dd, J = 8.3 and 2.5 Hz, 1H), 4.79 (d, J = 2.4 Hz, 2H), 2.58 (t, J = 2.4 Hz, 1H). **HRMS (ESI) m/z**: $[M+H]^+$ calcd. 178.0498 found $[M+H]^+$: 178.0498. Characterization data in agreement with literature.[13]

4-methyl-*N*-(prop-2-yn-1-yl)benzenesulfonamide (**Al-60**)

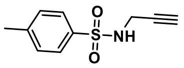

Synthesis according to the **general procedure 2.** The crude mixture was purified by flash chromatography using a mixture EOPet:EtOAc 70:30. The product was obtained as a pale yellow solid (1.81 g, 87%). **^{1}H NMR** (600 MHz, $CDCl_3$) δ**:** 7.80 – 7.75 (m, 2H), 7.35 – 7.30 (m, 2H), 4.55 (br, 1H), 3.83 (dd, J = 6.0 and 2.5 Hz, 2H),

2.44 (s, 3H), 2.11 (t, J = 2.5 Hz, 1H). **HRMS (ESI) m/z**: $[M+H]^+$ calcd. 210.0582 found $[M+H]^+$: 210.0579. Characterization data in agreement with literature.[22]

4-fluoro-*N*-(prop-2-yn-1-yl)benzamide (**Al-64**)

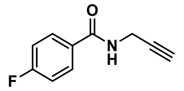

Synthesis according to the **general procedure 2.** The crude mixture was purified by recrystallisation using a DCM:Hex mixture. The product was obtained as white crystals (195 mg, 67%). **^{1}H NMR** (400 MHz, $CDCl_3$) δ**:** 7.83 – 7.77 (m, 2H), 7.16 – 7.09 (m, 2H), 6.18 (br, 1H), 4.25 (dd, J = 5.2 and 2.6 Hz, 2H), 2.29 (t, J = 2.6 Hz, 1H). **HRMS (ESI) m/z**: $[M+H]^+$ calcd. 178.0662 found $[M+H]^+$: 178.0660. Characterization data in agreement with literature.[4]

3-bromo-*N*-(prop-2-yn-1-yl)benzamide (**Al-65**)

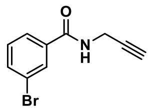

Synthesis according to the **general procedure 2.** The crude mixture was purified by recrystallisation using a DCM:Hex mixture. The product was obtained as yellow crystals (130 mg, 56%). **^{1}H NMR** (400 MHz, $CDCl_3$) δ**:** 7.93 (t, J = 1.9 Hz, 1H), 7.74 – 7.61 (m, 2H), 7.31 (t, J = 7.9 Hz, 1H), 6.38 (br, 1H), 4.24 (dd, J = 5.2 and 2.6 Hz, 2H), 2.29 (t, J = 2.5 Hz, 1H). **HRMS (ESI) m/z**: $[M-H]^-$ calcd. 235.9717 found $[M-H]^-$: 235.9717. Characterization data in agreement with literature.[23]

1-chloro-*N*-(prop-2-yn-1-yl)methanesulfonamide (**Al-66**)

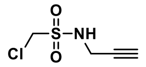

Synthesis according to the **general procedure 2.** The crude mixture was purified by recrystallisation using a DCM:Hex mixture. The product was obtained as orange oil (142 mg, 66%). **^{1}H NMR** (600 MHz, $CDCl_3$) δ**:** 4.96 (br, 1H), 4.63 (s, 2H), 4.03 (dd, J = 6.3 and 2.5 Hz, 2H), 2.42 (t, J = 2.5 Hz, 1H). **HRMS (ESI) m/z**: $[M-H]^-$ calcd. 165.9736 found $[M-H]^-$: 165.9733.

3-bromo-*N*-(prop-2-yn-1-yl)benzenesulfonamide (**Al-67**)

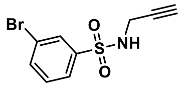

Synthesis according to the **general procedure 2.** The crude mixture was purified by recrystallisation using a DCM:Hex mixture. The product was obtained as a white solid (278 mg, 56%). **^{1}H NMR** (400 MHz, $CDCl_3$) δ**:** 8.05 (t, J = 1.9 Hz, 1H), 7.83 (dd, J = 7.9 and 1.8 Hz, 1H), 7.72 (dd, J = 8.0 and 2.0 Hz, 1H), 7.41 (t, J = 7.9 Hz, 1H), 4.68 (br, 1H), 3.90 (dd, J = 6.1 and 2.5 Hz, 2H), 2.13 (t, J = 2.5 Hz, 1H). **HRMS (ESI) m/z**: $[M-H]^-$ calcd. 271.9387 found $[M-H]^-$: 271.9390.

2,2,2-trifluoro-*N*-(3-(prop-2-yn-1-yloxy)phenyl)acetamide (**Al-68**)

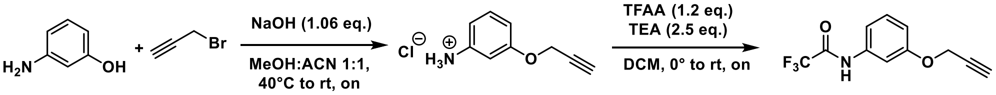

Step 1: In a round-bottom flask to a solution of 3-amino phenol (1.0 eq.) was added to a solution containing NaOH (1.065 eq.) and MeOH (5 mL). The solution was warmed up at 50 °C for 15 mins. Successively, the mixture was evaporated under reduced pressure to remove MeOH. The crude was taken with EtOH (5 mL) and evaporated under reduced pressure. This operation was repeated four times. Next, ACN (5 mL) was added followed by propargyl bromide (1.2 eq.) dropwise. The reaction was stirred at rt overnight. Once disappearance of the starting materials was confirmed by TLC, the solution was evaporated under reduced pressure. To the resulting brown solid MeOH (3 mL) was added followed by a solution of HCl conc. in MeOH (10 mL). The mixture was stirred 10 minutes at rt. The mixture was evaporated under reduced pressure and the resulting brownish solid was recrystallysed with EtOAc (6 mL) to afford 3-(prop-2-yn-1-yloxy)benzenaminium chloride as a white solid (508 mg, 38%). **^{1}H NMR** (400 MHz, *d*-DMSO) δ**:** 7.44 – 7.32 (m, 1H), 6.99 – 6.88 (m, 3H), 4.83 (d, J = 2.4 Hz, 2H), 3.63 (t, J = 2.4 Hz, 1H). **HRMS (ESI) m/z**: calcd. $[M+H]^+$: 148.0757, found. $[M+H]^+$: 148.0760.

Step 2: In a round-bottom flask to a solution of 3-(prop-2-yn-1-yloxy)benzenaminium chloride (1.0 eq.) was added to a solution containing DCM (5 mL) at 0° C. TEA (2.5 eq.) and trifluoro acetic anhydride (TFAA, 1.2 eq.) were added sequentially dropwise. The solution was allowed to stir at rt overnight. Once disappearance of the starting materials was confirmed by TLC, the solution was further diluted with DCM (5 mL). The organic layer (10 mL) was washed with: 1) HCl 1 M (5 mL); 2) 5% $NaHCO_3$ (5 mL); 3) Brine (5 mL). Successively, the organic layer was dried over a $MgSO_4$ bed and evaporated under reduced pressure. The crude mixture was purified by flash chromatography using a mixture EtOPet:EtOAc 80:20. The product was obtained as a white solid (35 mg, 27%). **^{1}H NMR** (400 MHz, $CDCl_3$) δ**:** 7.82 (br, 1H), 7.36 – 7.28 (m, 2H), 7.12 (m, 1H), 6.87 (m, 1H), 4.71 (d, J = 2.4 Hz, 2H), 2.54 (t, J = 2.4 Hz, 1H). **HRMS (ESI) m/z**: calcd. $[M+H]^+$: 244.0579, found $[M+H]^+$: 244.0578.

2,2,2-trifluoro-*N*-(prop-2-yn-1-yl)acetamide (**Al-73**)

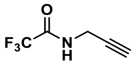

Synthesis according to the **general procedure 2.** The crude mixture was purified by recrystallisation using a DCM:Hex mixture. The product was obtained as a brown oil (100 mg, 76%). **^{1}H NMR** (400 MHz, $CDCl_3$) δ**:** 6.54 (br, 1H), 4.16 (dd, J = 5.4 and 2.6 Hz, 2H), 2.34 (t, J = 2.6 Hz, 1H). **HRMS (ESI) m/z**: calcd. $[M+H]^+$: 152.0317 found $[M+H]^+$: 152.0321. Characterization data in agreement with literature.[24]

tert-butyl prop-2-yn-1-ylcarbamate (**Al-74**)

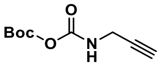

Synthesis according to the **general procedure 2.** The crude mixture was purified by flash chromatography using a mixture EOPet:EtOAc 80:20. The product was obtained as yellow crystals (274 mg, 98%). **^{1}H NMR** (400 MHz, $CDCl_3$) δ**:** 4.69 (br, 1H), 3.92 (dd, J = 5.5 and 2.6 Hz, 2H), 2.22 (t, J = 2.5 Hz, 1H), 1.45 (s, 9H). **HRMS (ESI) m/z**: $[M+H-C_4H_9]^+$ calcd. 100.0392 found $[M+H-C_4H_9]^+$: 100.0398. Characterization data in agreement with literature.[25]

phenyl(prop-2-yn-1-yl)sulfane (**Al-76**)

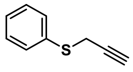

Synthesis according to the procedure reported for **Al-39 (step 1)**. The crude mixture was purified by flash chromatography using a 95:5 EtOPet:EtOAc mixture. The product was obtained as an orange oil (771 mg, 97%). **^{1}H NMR** (600 MHz, $CDCl_3$) δ**:** 7.47 – 7.45 (m, 2H), 7.33 (dd, J = 8.4 and 6.9 Hz, 2H), 7.26 – 7.23 (m, 1H), 3.61 (d, J = 2.6 Hz, 2H), 2.24 (t, J = 2.6 Hz, 1H). **HRMS (ESI) m/z**: $[M+H]^+$ calcd. 149.0419 found $[M+H]^+$: 149.0416. Characterization data in agreement with literature.[26]

N-(3-(prop-2-yn-1-yloxy)phenyl)acetamide (**Al-77**)

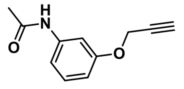

Synthesis according to the procedure reported for **Al-68 (step 1 and 2)** and using acetyl chloride as starting material. The crude mixture was purified by flash chromatography using a 50:50 EtOPet:EtOAc mixture. The product was obtained as a white solid (191 mg, 93%). **^{1}H NMR** (600 MHz, $CDCl_3$) δ**:** 7.32 (t, J = 2.4 Hz, 2H), 7.22 (t, J = 8.2 Hz, 1H), 7.05 – 6.99 (m, 1H), 6.73 (dd, J = 8.3 and 2.5 Hz, 1H), 4.68 (d, J = 2.4 Hz, 2H), 2.52 (t, J = 2.4 Hz, 1H), 2.17 (s, J = 2.0 Hz, 3H). **HRMS (ESI) m/z**: $[M+H]^+$ calcd. 190.0862 found $[M+H]^+$: 190.0861. Characterization data in agreement with literature.[27]

tert-butyl (3-(prop-2-yn-1-yloxy)phenyl)carbamate (**Al-81**)

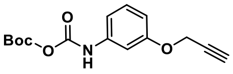

Synthesis according to the procedure reported for **Al-39 (step 1)** starting from *tert*-butyl (3-hydroxyphenyl)carbamate as starting material. The crude mixture was purified by flash chromatography using a 90:10 EtOPet:EtOAc mixture. The product was obtained as a white solid (358 mg, 89%). **^{1}H NMR** (400 MHz, $CDCl_3$) δ**:** 7.19 (t, J = 8.1 Hz, 1H), 7.11 (t, J = 2.3 Hz, 1H), 6.93 (dd, J = 8.0 and 2.1 Hz, 1H), 6.66 (dd, J = 8.3 and 2.5 Hz, 1H), 6.45 (br, 1H), 4.68 (d, J = 2.4 Hz, 2H), 2.51 (t, J = 2.4 Hz, 1H). **HRMS (ESI) m/z**: $[M+H-C_4H_9]^+$ calcd. 192.0654 found $[M+H-C_4H_9]^+$: 192.0655. Characterization data in agreement with literature.[28]

N-(prop-2-yn-1-yl)acetamide (**Al-82**)

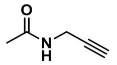

Synthesis according to the **general procedure 2.** The crude mixture was purified by recrystallisation using a DCM:Hexane mixture. The product was obtained as yellow crystals (253 mg, 64%). **^{1}H NMR** (600 MHz, $CDCl_3$) δ**:** 5.81 (br, 1H), 4.04 (dd, J = 5.2 and 2.6 Hz, 2H), 2.22 (t, J = 2.6 Hz, 1H), 2.00 (s, 3H). **HRMS (ESI) m/z**: Not detected due to low MW. Characterization data in agreement with literature.[25]

N-(3-ethynylphenyl)-2,2,2-trifluoroacetamide (**Al-83**)

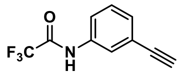

Synthesis according to the procedure reported for **Al-68 (step 2)** using 3-ethynyl aniline as starting material**.** The crude mixture was purified by flash chromatography using a 95:5 EtOPet:EtOAc mixture. The product was obtained as white powder (155 mg, 85%). **^{1}H NMR** (600 MHz, $CDCl_3$) δ**:** 7.83 (br, 1H), 7.71 – 7.68 (m, 1H), 7.60 – 7.57 (m, 1H), 7.38 – 7.35 (m, 2H), 3.12 (s, 1H). **HRMS (ESI) m/z**: $[M-H]^-$ calcd. 212.0329 found $[M-H]^-$: 212.0324.

N-(3-ethynylphenyl)methanesulfonamide (**Al-84**)

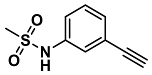

Synthesis according to the **general procedure 2.** The crude mixture was purified by flash chromatography using a 60:40 EtOPet:EtOAc mixture. The product was obtained as white powder (217 mg, 64%). **^{1}H NMR** (400 MHz, $CDCl_3$) δ**:** 7.60 (dt, J = 7.8 and 1.4 Hz, 1H), 7.48 (t, J = 1.9 Hz, 1H), 7.44 (t, J = 7.8 Hz, 1H), 7.35 (dd, J = 8.0 and 2.2 Hz, 1H), 3.41 (s, 3H), 3.16 (s, 1H). **HRMS (ESI) m/z**: $[M-H]^-$ calcd. 194.0282 found $[M-H]^-$: 194.0275. Characterization data in agreement with literature.[29]

2,2,2-trifluoro-*N*-(prop-2-yn-1-yl)ethane-1-sulfonamide (**Al-85**)

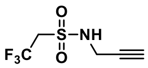

Synthesis according to the **general procedure 2.** The crude mixture was purified by recrystallisation using a DCM:Hexane mixture. The product was obtained as an orange solid (260 mg, 89%). **^{1}H NMR** (600 MHz, $CDCl_3$) δ**:** 4.93 (br, 1H), 4.04 (dd, J = 6.2 and 2.5 Hz, 2H), 3.98 (q, J = 8.9 Hz, 2H), 2.44 (t, J = 2.5 Hz, 1H). **HRMS (ESI) m/z**: $[M-H]^-$ calcd. 199.9999 found $[M-H]^-$: 199.9993. Characterization data in agreement with literature.[5]

methyl 4-(prop-2-yn-1-ylcarbamoyl)benzoate (**Al-88**)

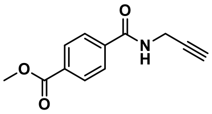

Synthesis according to the **general procedure 2** starting from methyl 4-(chlorocarbonyl)benzoate. The crude mixture was purified by recrystallisation using a DCM:Hexane mixture. The product was obtained as a yellow solid (176 mg, 74%). **^{1}H NMR** (400 MHz, $CDCl_3$) δ**:** 8.13 – 8.08 (m, 2H), 7.87 – 7.82 (m, 2H), 6.34 (br, 1H), 4.27 (dd, J = 5.2 and 2.6 Hz, 2H), 3.95 (s, 3H), 2.30 (t, J = 2.6 Hz, 1H). **HRMS (ESI) m/z**: $[M-H]^-$ calcd. 216.0667 found $[M-H]^-$: 216.0661. Characterization data in agreement with literature.[30]

General procedure and characterization data for protected and deprotected triazoles

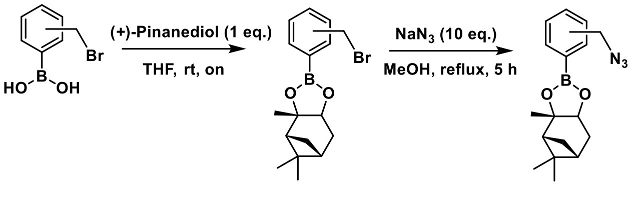

15 16

General procedure 6: **Step-1**: In a round-bottom flask *o*-, *m*- or *p*-(bromomethyl)phenylboronic acid (1.0 eq.) was added to a stirred solution of tetrahydrofuran (THF, 10 mL). (+)-Pinanediol (1.0 eq.) was added portion-wise and the mixture stirred at rt overnight. Then, THF was evaporated under reduced pressure and Et_2O (10 mL) added and stripped away to remove any excess of pinanediol and afford the protected boronic acid.

Step-2: Same procedure as **General procedure 1**, **method B**.

1,4-Regioisomers

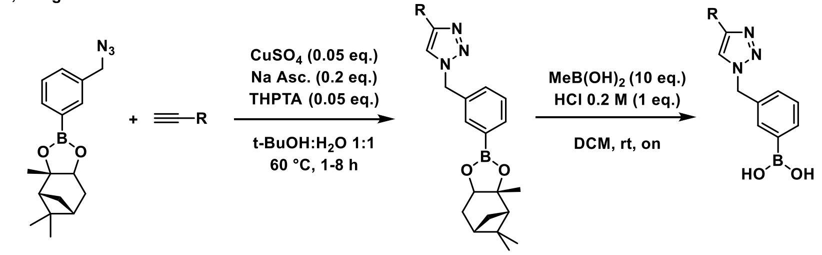

16

1,5-Regioisomer

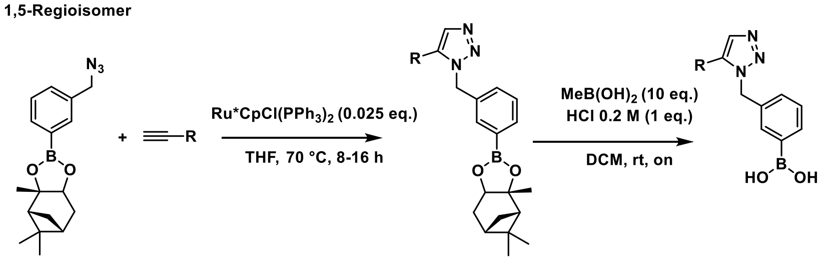

16

General procedure 7: **Step 1** (CuAAC): In a Schlenk tube compound **16** (1.0 eq.) and the alkyne (1.1 eq.) were added to a stirred solution of *tert*-butanol (1 mL). A 0.05 M solution in water of $CuSO_4$ (0.05 eq.) was added, followed by sodium ascorbate (0.2 eq.) and THPTA (0.05 eq.) and water to reach 1 mL. The reaction was stirred and warmed up to 60 °C under argon until disappearance of the starting materials. After completion, EtOAc (5 mL) was added to the reaction mixture and the aqueous phase extracted. The aqueous phase was extracted 2 more times with EtOAc (2x5 mL). The combined organic phases were reunited, washed with brine (7 mL), dried over a $MgSO_4$ bed and evaporated under reduced pressure to afford the crude product.

Step 2 (**Deprotection**): In a 10 mL round-bottom flask, the pinanediol esters (1.0 eq.) from step 1 were dissolved in acetone (2 mL) and in a 0.2 N solution of aqueous HCl (1.0 eq). Methyl boronic acid (10.0 eq.) was then added and the reaction stirred at room temperature overnight. After checking the disappearance

of the pinanediol esters by TLC, the crude mixture was evaporated under reduced pressure. Dichloromethane was added to the crude and evaporated under reduced pressure to strip away the methyl boronic acid left. This operation was repeated several times until removal of methyl boronic acid to afford the desired compounds.

General procedure 8: Step 1 (RuAAC): In a Schlenk tube compound **16** (1.0 eq.) and the alkyne (1.1 eq.) were added to a stirred solution THF (1 mL). The catalyst pentamethylcyclopentadienylbis (triphenylphosphine)ruthenium(II) chloride ($Cp^*RuCl(PPh_3)_2$, 0.025 eq.) was added and the reaction warmed up to 70 °C under argon until disappearance of the starting materials. After completion, EtOAc (5 mL) was added to the reaction mixture and the aqueous phase extracted. The aqueous phase was extracted 2 more times with EtOAc (2x5 mL). The combined organic phases were reunited, washed with brine (7 mL), dried over a $MgSO_4$ bed and evaporated under reduced pressure to afford the crude product.

Step 2 (**Deprotection**): Same as Step 1 in the general procedure 6.

(3a*S*,4*R*,6*R*)-2-(3-(bromomethyl)phenyl)-3*a*,5,5-trimethylhexahydro-4,6-methanobenzo [*d*][1,3,2] dioxaborole (**15**)

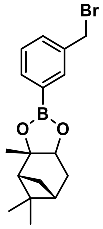

Synthesis according to the **general procedure 6: step 1**. The crude mixture was used without further purification to afford product **15**, a yellowish solid (3.18 g, 98% yield). **15** was used as intermediate for the synthesis of product **16**. **^{1}H NMR** (400 MHz, $CDCl_3$) δ**:** 7.83 (t, J = 1.6 Hz, 1H), 7.75 (dt, J = 7.4 and 1.3 Hz, 1H), 7.50 (dt, J = 7.7 and 1.6 Hz, 1H), 7.37 (t, J = 7.5 Hz, 1H), 4.51 (s, 2H), 4.46 (dd, J = 8.8 and 1.9 Hz, 1H), 2.45-2.40 (m, 1H), 2.28 – 2.21 (m, 1H), 2.16 (dd, J = 6.1 and 4.9 Hz, 1H), 2.01 – 1.94 (m, 2H), 1.32 (s, 3H), 1.22 – 1.19 (m, 1H), 0.90 (s, 3H).

(3a*S*,4*R*,6*R*)-2-(3-(azidomethyl)phenyl)-3*a*,5,5-trimethylhexahydro-4,6-methanobenzo[*d*][1,3,2] dioxaborole (**16**)

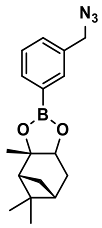

Synthesis according to the **general procedure 6: step 2**. Purified by flash chromatography EtOPet:EtOAc= 95:5 to afford transparent crystals (2.00 g, 82% yield). **^{1}H NMR** (400 MHz, $CDCl_3$) δ: 7.79 (m, 1H), 7.77 – 7.72 (m, 1H), 7.47 – 7.37 (m, 2H), 4.47 (dd, J = 8.8 and 1.9 Hz, 1H), 4.36 (s, 2H), 2.43-2.40 (m, 1H), 2.28–2.20 (m, 1H), 2.19–2.13 (m, 1H), 2.01–1.92 (m, 2H), 1.49 (s, 3H), 1.32 (s, 3H), 1.21 (d, J = 10.9 Hz, 1H), 0.90 (s, 3H). **^{13}C NMR** (101 MHz, $CDCl_3$) δ: 134.88, 134.77, 131.12, 128.44, 86.61, 78.51, 54.94, 51.55, 39.68, 38.37, 35.67, 28.85,

27.25, 26.65, 24.20. **^{11}B NMR** (128 MHz, $CDCl_3$) δ: 30.11. **HRMS (ESI) m/z**: [M-Pinanendiol+HCOO]$^-$ calcd. for 222.0686 found [M-Pinanendiol+HCOO]$^-$: 222.0687.

3-((1-(3-((3a*S*,4*R*,6*R*)-3a,5,5-trimethylhexahydro-4,6-methanobenzo[*d*][1,3,2]dioxaborol-2-yl)benzyl)-1*H*-1,2,3-triazol-4-yl)methoxy)benzamide (**5_P**)

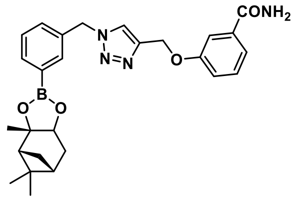

Synthesis according to the **general procedure 7, step 1**. The crude mixture was used without further purification to afford red powder (73 mg, 93% yield). **^{1}H NMR** (600 MHz, $CDCl_3$) δ**:** 7.81 (dt, J = 7.3 and 1.3 Hz, 1H), 7.79 (d, J = 1.9 Hz, 1H), 7.55 (s, 1H), 7.47 (t, J = 2.0 Hz, 1H), 7.41 – 7.32 (m, 4H), 7.12 (d, J = 6 Hz, 1H), 6.13 (s, 1H), 5.53 (s, 2H), 5.21 (s, 2H), 4.46 (dd, J = 8.8 and 1.9 Hz, 1H), 2.45-2.40 (m, 1H), 2.27 – 2.21 (m, 1H), 2.16 – 2.14 (m, 1H), 1.99 – 1.93 (m, 2H), 1.48 (s, 3H), 1.32 (s, 3H), 1.25 (t, J = 3.5 Hz, 1H), 1.19 (d, J = 11.0 Hz, 1H), 0.89 (s, 3H). **^{13}C NMR** (151 MHz, $CDCl_3$) δ: 169.06, 158.50, 135.51, 134.81, 133.75, 131.23, 129.94, 128.87, 120.14, 118.99, 113.80, 86.75, 78.57, 77.16, 62.20, 54.54, 51.51, 39.65, 38.37, 35.61, 28.83, 27.23, 26.65, 24.19. **^{11}B NMR** (193 MHz, $CDCl_3$) δ: 29.96. **HRMS (ESI) m/z**: [M+H]$^+$ calcd. for 487.2510 found [M+H]$^+$: 487.2518.

(3-((4-((3-carbamoylphenoxy)methyl)-1*H*-1,2,3-triazol-1-yl)methyl)phenyl)boronic acid (**5**)

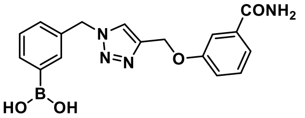

Synthesis according to the **general procedure 7, step 2**. The crude mixture was used without further purification to afford a dark yellow powder (40 mg, 81% yield). **^{1}H NMR** (600 MHz, MeOD) δ**:** 8.14 (s, 1H), 7.67 (s, 2H), 7.52 – 7.45 (m, 2H), 7.39 – 7.34 (m, 3H), 7.18 (dd, J = 8.2 and 2.5 Hz, 1H), 5.63 (s, 2H), 5.22 (s, 2H). **^{13}C NMR** (151 MHz, MeOD) δ: 159.68, 135.43, 135.03, 134.56, 130.78, 129.32, 125.83, 121.60, 119.70, 114.94, 62.21, 55.55. **^{11}B NMR** (193 MHz, $CDCl_3$) δ: 28.33. **HRMS (ESI) m/z**: [M+H]$^+$ calcd. for 353.1415 found [M+H]$^+$: 353.1414.

4-((phenylsulfonyl)methyl)-1-(3-((3a*S*,4*R*,6*R*)-3a,5,5-trimethylhexahydro-4,6-methanobenzo[*d*][1,3,2] dioxaborol-2-yl)benzyl)-1*H*-1,2,3-triazole (**6_P**)

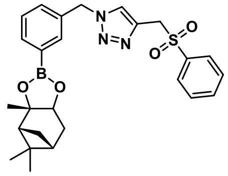

Synthesis according to the **general procedure 7, step 1**. The crude mixture was used without further purification to afford red powder (66 mg, 83% yield). **^{1}H NMR** (600 MHz, $CDCl_3$) δ**:** 7.84 (d, J = 7.3 Hz, 1H), 7.75 (s, 1H), 7.64 – 7.57 (m, 4H), 7.43 (dt, J = 16.9 and 7.6 Hz, 3H), 7.32 (dt, J = 7.8 and 1.7 Hz, 1H), 5.51 (s, 2H), 4.51 (s, 2H), 4.47 (dd, J = 8.8 and 1.9 Hz, 1H), 2.45-2.40 (m, 1H), 2.28-2.22 (m, 1H), 2.16 (t, J = 5.5 Hz, 1H), 2.02 – 1.93 (m, 2H), 1.50 (s, 3H), 1.32 (s, 3H), 1.20 (d, J = 10.9 Hz, 1H), 0.89 (s, 3H). **^{13}C NMR** (151 MHz,

CDCl3) δ: 137.76, 136.44, 135.59, 134.60, 134.14, 133.73, 131.03, 129.29, 128.84, 128.58, 124.38, 86.80, 78.60, 77.16, 54.56, 54.16, 51.52, 39.65, 38.37, 35.62, 28.86, 27.23, 26.68, 24.19. **^{11}B NMR** (193 MHz, CDCl3) δ: 30.36. **HRMS (ESI) m/z**: $[M+H]^+$ calcd. for 492.2122 found $[M+H]^+$: 492.2130.

(3-((4-((phenylsulfonyl)methyl)-1*H*-1,2,3-triazol-1-yl)methyl)phenyl)boronic acid (**6**)

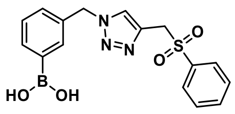

Synthesis according to the **general procedure 7, step 2**. The crude mixture was used without further purification to afford white powder (32 mg, 72% yield). **^{1}H NMR** (600 MHz, MeOD) δ: 7.66 (s, 1H), 7.60 – 7.34 (m, 5H), 7.34 – 7.20 (m, 3H), 7.16 (dt, J = 7.7 and 1.7 Hz, 1H), 5.41 (s, 2H), 4.48 (s, 2H).**^{13}C NMR** (151 MHz, MeOD) δ: 137.61, 135.18, 134.71, 134.14, 130.37, 130.28, 129.55, 129.35, 126.70, 55.10, 54.13. **^{11}B NMR** (193 MHz, CDCl3) δ: 28.43. **HRMS (ESI) m/z**: $[M+H]^+$ calcd. for 358.1027 found $[M+H]^+$: 358.1028.

methyl 3-((*N*-((1-(3-((3a*S*,4*R*,6*R*)-3a,5,5-trimethylhexahydro-4,6-methanobenzo[*d*][1,3,2]dioxaborol-2-yl)benzyl)-1*H*-1,2,3-triazol-4-yl)methyl)sulfamoyl)methyl)benzoate (**7_P**)

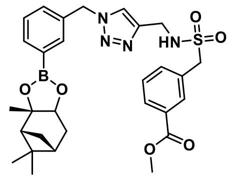

Synthesis according to the **general procedure 7, step 1**. The crude mixture was used without further purification to afford transparent crystals (85 mg, 88% yield). **^{1}H NMR** (600 MHz, CDCl3) δ**:** 8.02 (d, J = 7.6 Hz, 1H), 7.98 (s, 1H), 7.80 (d, J = 7.0 Hz, 1H), 7.76 (s, 1H), 7.55 (d, J = 7.4 Hz, 1H), 7.45-7.36 (m, 4H), 5.51 (s, 2H), 4.90 (s, 1H), 4.44 (dd, J = 8.8 and 1.8 Hz, 1H), 4.26 (d, J = 12.7 Hz, 3H), 3.92 (s, 3H), 2.45 – 2.38 (m, 1H), 2.25-2.19 (m, 1H), 2.13 (t, J = 5.4 Hz, 1H), 1.97 – 1.93 (m, 2H), 1.47 (s, 3H), 1.31 (s, 3H), 1.16 (d, J = 11.0 Hz, 1H), 0.88 (s, 3H). **^{13}C NMR** (151 MHz, CDCl3) δ: 166.56, 135.62, 135.32, 134.76, 133.58, 131.94, 131.19, 130.96, 130.06, 129.69, 129.12, 128.89, 86.75, 78.56, 77.16, 59.44, 54.73, 52.49, 51.50, 39.64, 39.00, 38.36, 35.59, 28.82, 27.22, 26.65, 24.18. **^{11}B NMR** (193 MHz, CDCl3) δ: 30.11. **HRMS (ESI) m/z**: $[M+H]^+$ calcd. for 579.2442 found $[M+H]^+$: 579.2442.

(3-((4-((((3-(methoxycarbonyl)phenyl)methyl)sulfonamido)methyl)-1*H*-1,2,3-triazol-1-yl)methyl)
phenyl)boronic acid (**7**)

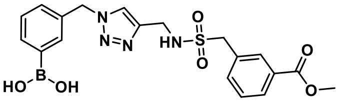

Synthesis according to the **general procedure 7, step 2**. The crude mixture was used without further purification to afford yellow powder (49.5 mg, 70% yield). **^{1}H NMR** (600 MHz, MeOD) δ**:** 7.99 (dd, J = 6.8 and 1.5 Hz, 2H), 7.92 (s, 1H), 7.72 – 7.62 (m, 2H), 7.55 (dt, J = 7.7 and 1.5 Hz, 1H), 7.44 – 7.34 (m, 3H), 5.61 (s, 2H), 4.36 (s, 2H), 4.27 (s, 2H), 3.91 (s, 3H). **^{13}C NMR** (151 MHz, MeOD) δ: 168.06, 136.63, 135.60, 134.98, 134.57, 132.95, 131.97, 131.68, 130.84, 130.39, 129.81, 129.29, 124.96, 59.20, 55.44, 52.74, 38.90. **^{11}B NMR** (193 MHz, CDCl3) δ: 28.47. **HRMS (ESI) m/z**: $[M+H]^+$ calcd. For 445.1347 found $[M+H]^+$: 445.1347.

methyl 3-((*N*-((1-(3-((3a*S*,4*R*,6*R*)-3a,5,5-trimethylhexahydro-4,6-methanobenzo[d][1,3,2]dioxaborol-2-yl)benzyl)-1*H*-1,2,3-triazol-5-yl)methyl)sulfamoyl)methyl)benzoate (**7a_P**)

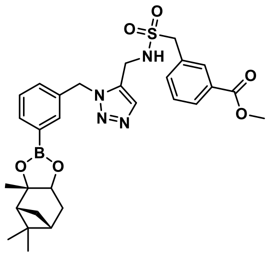

Synthesis according to the **general procedure 8, step 1**. The crude mixture was purified using flash chromatography DCM:EtOAc 1:1 to afford a beige solid (36 mg, 41% yield). **^{1}H NMR** (400 MHz, $CDCl_3$) δ**:** 8.03 (dt, J = 7.0 and 1.8 Hz, 1H), 7.93 (s, 1H), 7.77 (d, J = 7.4 Hz, 1H), 7.67 (s, 1H), 7.60 (s, 1H), 7.47 – 7.41 (m, 2H), 7.34 (t, J = 7.5 Hz, 1H), 7.20 (d, J = 7.8 Hz, 1H), 5.53 (s, 2H), 4.59 (t, J = 6.0 Hz, 1H), 4.42 (dd, J = 8.7 and 1.9 Hz, 1H), 4.15 (s, 2H), 4.06 (d, J = 6.0 Hz, 2H), 3.92 (s, 3H), 2.44 – 2.31 (m, 1H), 2.20 (m, 1H), 2.12 (m, 1H), 2.00 – 1.88 (m, 2H), 1.46 (s, 3H), 1.30 (s, 3H), 1.15 (d, J = 10.9 Hz, 1H), 0.87 (s, 3H). **^{13}C NMR** (101 MHz, $CDCl_3$) δ: 166.29, 135.22, 134.99, 134.48, 133.69, 133.60, 132.63, 131.55, 130.94, 130.18, 130.08, 129.13, 129.05, 128.74, 86.63, 78.42, 59.50, 52.43, 52.13, 51.35, 39.49, 38.21, 35.90, 35.42, 28.67, 27.08, 26.50, 24.04. **^{11}B NMR** (193 MHz, $CDCl_3$) δ: 31.31. **HRMS (ESI) m/z**: $[M+H]^+$ calcd. for 579.2442 found $[M+H]^+$: 579.2427.

(3-((5-((((3-(methoxycarbonyl)phenyl)methyl)sulfonamido)methyl)-1*H*-1,2,3-triazol-1-yl)methyl)phenyl) boronic acid (**7a**)

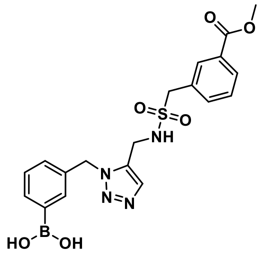

Synthesis according to the **general procedure 8, step 2**. The crude mixture was used without further purification to afford beige solid (9 mg, 45% yield). **^{1}H NMR** (600 MHz, MeOD) δ: 8.04 – 7.98 (m, 2H), 7.79 (s, 1H), 7.61 (d, J = 7.6 Hz, 2H), 7.48 (t, J = 7.7 Hz, 2H), 7.36 (t, J = 7.5 Hz, 1H), 7.24 (d, J = 7.7 Hz, 1H), 5.62 (s, 2H), 4.39 (s, 2H), 4.16 (s, 2H), 3.92 (s, 3H). **^{13}C NMR** (151 MHz, MeOD) δ: 167.96, 136.57, 134.98, 133.77, 132.88, 131.84, 131.72, 130.63, 130.02, 129.98, 129.40, 59.15, 53.24, 52.77, 36.66. **^{11}B NMR** (193 MHz, $CDCl_3$) δ: 28.48. **HRMS (ESI) m/z**: $[M+H]^+$ calcd. for 445.1347 found $[M+H]^+$: 445.1335.

N-((1-(3-((3a*S*,4*R*,6*R*)-3a,5,5-trimethylhexahydro-4,6-methanobenzo[*d*][1,3,2]dioxaborol-2-yl)benzyl)-1*H*-1,2,3-triazol-4-yl)methyl)pentanamide (**8_P**)

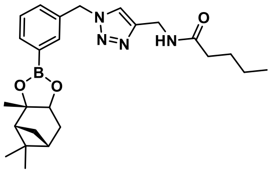

Synthesis according to the **general procedure 7, step 1**. The crude mixture was used without further purification to afford transparent crystals (72 mg, 99% yield). **^{1}H NMR** (600 MHz, $CDCl_3$) δ**:** 7.81 (dt, J = 7.2

and 1.3 Hz, 1H), 7.77 (d, J = 2.0 Hz, 1H), 7.48 (s, 1H), 7.42 – 7.33 (m, 2H), 5.50 (s, 2H), 4.51 – 4.43 (m, 3H), 2.43-2.40 (m, 1H), 2.28 – 2.16 (m, 1H), 2.19 – 2.14 (m, 3H), 1.58 (dq, J = 9.0 and 7.5 Hz, 2H), 1.36 – 1.23 (m, 6H), 1.19 (d, J = 11.0 Hz, 1H), 0.89 (d, J = 7.2 Hz, 6H). **^{13}C NMR** (151 MHz, $CDCl_3$) δ: 173.36, 135.58, 134.84, 133.54, 131.21, 128.85, 86.75, 78.58, 54.70, 51.51, 39.65, 38.36, 36.39, 35.61, 34.76, 28.83, 27.74, 27.23, 26.66, 24.18, 22.49, 13.90. **^{11}B NMR** (193 MHz, $CDCl_3$) δ: 29.46. **HRMS (ESI) m/z**: $[M+H]^+$ calcd. for 451.2874 found $[M+H]^+$: 451.2863.

(3-((4-(pentanamidomethyl)-1*H*-1,2,3-triazol-1-yl)methyl)phenyl)boronic acid (**8**)

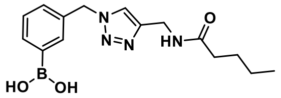

Synthesis according to the **general procedure 7, step 2**. The crude mixture was used without further purification to afford white powder (25 mg, 60% yield). **^{1}H NMR** (600 MHz, MeOD) δ**:** 7.93 (s, 1H), 7.67 (s, 2H), 7.36 (d, J = 7.5 Hz, 2H), 5.60 (s, 2H), 4.42 (s, 2H), 2.20 (q, J = 8.1 Hz, 2H), 1.55 (p, J = 8.4 Hz, 2H), 1.30 (dt, J = 14.7 and 8.3 Hz, 2H), 0.96 – 0.85 (m, 3H). **^{13}C NMR** (151 MHz, MeOD) δ: 134.65, 134.27, 130.42, 129.30, 124.17, 55.04, 36.61, 35.56, 29.01, 23.31, 14.07. **^{11}B NMR** (193 MHz, MeOD) δ: 28.64. **HRMS (ESI) m/z:** $[M-H]^+$ calcd. for 317.1779 found $[M-H]^+$: 317.1776.

3-methyl-1-(1-(3-((3a*S*,4*R*,6*R*)-3a,5,5-trimethylhexahydro-4,6-methanobenzo[*d*][1,3,2]dioxaborol-2-yl)benzyl)-1*H*-1,2,3-triazol-4-yl)butan-1-ol (**9_P**)

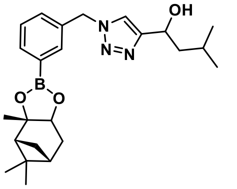

Synthesis according to the **general procedure 7, step 1**. The crude mixture was used without further purification to afford transparent crystals (63 mg, 94% yield). **^{1}H NMR** (600 MHz, $CDCl_3$) δ**:** 7.81 (dt, J = 7.4 and 1.3 Hz, 1H), 7.77 (s, 1H), 7.49 – 7.30 (m, 3H), 5.51 (s, 2H), 4.96 (s, 1H), 4.46 (dd, J = 8.8 and 1.9 Hz, 1H), 2.50 – 2.38 (m, 1H), 2.28 – 2.21 (m, 1H), 2.19 – 2.13 (m, 2H), 2.00 – 1.93 (m, 2H), 1.85-1.75 (m, 2H), 1.70-1.62 (m, 1H), 1.49 (s, 3H), 1.32 (s, 3H), 1.19 (d, J = 11.0 Hz, 1H), 0.96 – 0.94 (m, 6H), 0.89 (s, 3H). **^{13}C NMR** (151 MHz, $CDCl_3$) δ: 135.70, 134.87, 133.29, 131.28, 128.94, 86.79, 78.60, 51.52, 39.65, 38.38, 35.61, 28.84, 27.23, 26.66, 24.64, 24.19, 23.39, 21.98. **^{11}B NMR** (193 MHz, $CDCl_3$) δ: 30.08. **HRMS (ESI) m/z**: $[M+H]^+$ calcd. for 424.2765 found $[M+H]^+$: 424.2766.

(3-((4-(1-hydroxy-3-methylbutyl)-1*H*-1,2,3-triazol-1-yl)methyl)phenyl)boronic acid (**9**)

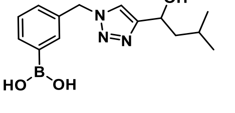

Synthesis according to the **general procedure 7, step 2**. The crude mixture was used without further purification to afford yellow powder (28 mg, 71% yield). **^{1}H NMR** (400 MHz, DMSO) δ**:** 7.93 (s, 1H), 7.75 (dt, J = 7.9 and 2.9 Hz, 2H), 7.35 (d, J = 4.5 Hz, 2H), 5.54 (s, 2H), 4.69 (dd, J = 8.7 and 5.2 Hz, 1H), 1.71 (p, J = 6.6 Hz, 1H), 1.64 – 1.49 (m, 2H), 0.93 – 0.85 (m, 6H). **^{13}C NMR** (101 MHz, DMSO) δ: 152.53, 135.07, 133.91,

133.77, 129.75, 127.75, 121.52, 63.71, 52.98, 46.51, 23.97, 23.14, 21.97. **^{11}B NMR** (193 MHz, $CDCl_3$) δ: 28.44. **HRMS (ESI) m/z**: [M+H]$^+$ calcd. for 290.1670 found [M+H]$^+$: 290.1661.

N-((1-(3-((3a*S*,4*R*,6*R*)-3a,5,5-trimethylhexahydro-4,6-methanobenzo[*d*][1,3,2]dioxaborol-2-yl)benzyl)-1*H*-1,2,3-triazol-4-yl)methyl)cyclohexanesulfonamide (**10_P**)

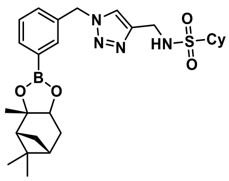

Synthesis according to the **general procedure 7, step 1**. The crude mixture was used without further purification to afford transparent crystals (73 mg, 91% yield). **^{1}H NMR** (600 MHz, $CDCl_3$) δ**:** 7.81 (dt, J = 7.1 and 1.4 Hz, 1H), 7.78 (d, J = 1.8 Hz, 1H), 7.48 (s, 1H), 7.41 – 7.35 (m, 2H), 5.51 (s, 2H), 4.56 (t, J = 5.8 Hz, 1H), 4.46 (dd, J = 8.8 and 1.9 Hz, 1H), 4.38 (d, J = 6.0 Hz, 2H), 2.77 (tt, J = 12.1 and 3.4 Hz, 1H), 2.45-2.40 (m, 1H), 2.26-2.21 (m, 1H), 2.17 – 2.09 (m, 3H), 1.99-1.93 (m, 2H), 1.84 (d, J = 10.4 Hz, 2H), 1.66 (t, J = 4.4 Hz, 1H), 1.49 (s, 4H), 1.32 (s, 3H), 1.25 (s, 1H), 1.21 – 1.14 (m, 4H), 0.89 (s, 3H). **^{13}C NMR** (151 MHz, $CDCl_3$) δ: 145.07, 135.56, 134.79, 133.77, 131.17, 128.87, 122.15, 86.76, 78.58, 61.98, 54.53, 51.52, 39.65, 38.99, 38.37, 35.62, 29.85, 28.84, 27.24, 26.67, 26.46, 25.23, 25.20, 24.19. **^{11}B NMR** (193 MHz, $CDCl_3$) δ: 30.31. **HRMS (ESI) m/z**: [M+H]$^+$ calcd. for 513.2701 found [M+H]$^+$: 513.2698.

(3-((4-(cyclohexanesulfonamidomethyl)-*1H*-1,2,3-triazol-1-yl)methyl)phenyl)boronic acid (**10**)

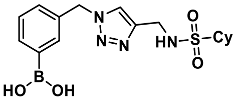

Synthesis according to the **general procedure 7, step 2**. The crude mixture was used without further purification to afford yellow powder (24 mg, 48% yield). **^{1}H NMR** (600 MHz, MeOD) δ: 7.92 (s, 1H), 7.84 – 7.71 (m, 1H), 7.62 (d, J = 10.3 Hz, 1H), 7.44 – 7.32 (m, 2H), 5.60 (d, J = 9.8 Hz, 2H), 4.33 (s, 2H), 2.77 (dt, J = 24.6 and 12.2 Hz, 1H), 2.02 (d, J = 12.3 Hz, 2H), 1.78 (s, 2H), 1.68 – 1.59 (m, 1H), 1.39 (q, J = 13.0 Hz, 2H), 1.15 (dd, J = 17.5 and 9.7 Hz, 3H). **^{13}C NMR** (101 MHz, MeOD) δ: 135.98, 135.14, 134.84, 134.70, 134.36, 131.13, 130.50, 129.26, 124.51, 62.15, 55.18, 38.83, 27.38, 26.29, 26.06. **^{11}B NMR** (128 MHz, MeOD) δ: 28.53. **HRMS (ESI) m/z**: [M+H]$^+$ calcd. for 379.1605 found [M+H]$^+$: 379.1598.

N-((1-(3-((3a*S*,4*R*,6*R*)-3a,5,5-trimethylhexahydro-4,6-methanobenzo[*d*][1,3,2]dioxaborol-2-yl)benzyl)-1*H*-1,2,3-triazol-5-yl)methyl)cyclohexanesulfonamide (**10a_P**)

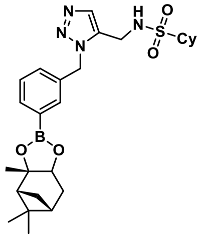

Synthesis according to the **general procedure 8**. The crude mixture was purified using flash chromatography DCM:EtOAc 1:1 (Rf= 0.36) to afford yellowish oil (34 mg, 40% yield). **^{1}H NMR** (600 MHz, $CDCl_3$) δ**:** 7.78 (dt, J = 7.2 and 1.3 Hz, 1H), 7.73 – 7.66 (m, 2H), 7.36 (t, J = 7.5 Hz, 1H), 7.26 – 7.23 (m, 1H), 5.63

(s, 2H), 4.45-4.42 (m, 2H), 4.25 (d, J = 6.1 Hz, 2H), 2.68-2.63 (m, 1H), 2.45-2.40 (m, 1H), 2.24 – 2.20 (m, 1H), 2.16 – 2.12 (m, 1H), 2.03 – 1.97 (m, 2H), 1.97 – 1.90 (m, 2H), 1.83 (dt, J = 13.1 and 3.2 Hz, 2H), 1.68 (s, 2H), 1.47 (s, 3H), 1.36 (qd, J = 12.3, 3.4 Hz, 2H), 1.31 (s, 3H), 1.23 – 1.15 (m, 4H), 0.88 (s, 3H). **^{13}C NMR** (151 MHz, $CDCl_3$) δ: 135.31, 134.47, 133.95, 133.70, 130.19, 128.88, 86.74, 78.55, 62.37, 52.28, 51.49, 39.63, 38.35, 35.91, 35.57, 28.81, 27.22, 26.63, 26.39, 25.14, 25.11, 24.17. **^{11}B NMR** (193 MHz, $CDCl_3$) δ: 30.34. **HRMS (ESI) m/z**: $[M+H]^+$ calcd. for 513.2701 found $[M+H]^+$: 513.2704.

(3-((5-(cyclohexanesulfonamidomethyl)-1*H*-1,2,3-triazol-1-yl)methyl)phenyl)boronic acid (**10a**)

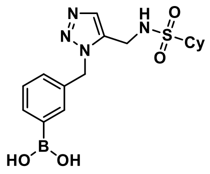

Synthesis according to the **general procedure 8, step 2**. The crude mixture was used without further purification to afford yellow-brown powder (19 mg, 76% yield). **^{1}H NMR** (600 MHz, MeOD) δ**:** 7.86 (s, 1H), 7.64 (d, J = 27.4 Hz, 2H), 7.39 – 7.30 (m, 2H), 5.72 (s, 2H), 4.33 (s, 2H), 2.86 (t, J = 12.0 Hz, 1H), 2.08 – 2.04 (m, 2H), 1.85 (dt, J = 13.3 and 3.3 Hz, 2H), 1.69 (dt, J = 13.1 and 3.6 Hz, 1H), 1.41 (q, J = 12.5 and 3.5 Hz, 2H), 1.29 (q, J = 12.9 and 3.5 Hz, 2H), 1.20 (t, J = 12.8 Hz, 1H). **^{13}C NMR** (151 MHz, MeOD) δ: 135.07, 134.89, 134.00, 130.32, 129.33, 62.18, 53.27, 36.52, 27.46, 26.28, 26.07. **^{11}B NMR** (193 MHz, MeOD) δ: 28.54. **HRMS (ESI) m/z**: $[M+H]^+$ calcd. for 379.1605 found $[M+H]^+$: 379.1599.

N,*N*-dimethyl-4-(1-(3-((3a*S*,4*R*,6*R*)-3a,5,5-trimethylhexahydro-4,6-methanobenzo[*d*][1,3,2]dioxaborol-2-yl)benzyl)-1*H*-1,2,3-triazol-4-yl)aniline (**11_P**)

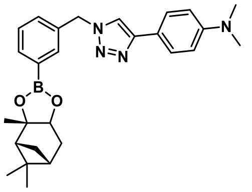

Synthesis according to the **general procedure 7, step 1**. The crude mixture was used without further purification to afford white solid (37 mg, 50% yield). **^{1}H NMR** (600 MHz, $CDCl_3$) δ**:** 7.84 – 7.78 (m, 2H), 7.67 (d, J = 8.4 Hz, 2H), 7.52 (s, 1H), 7.39 (dd, J = 4.9 and 1.6 Hz, 2H), 6.77 (s, 2H), 5.55 (s, 2H), 4.46 (dd, J = 8.8 and 1.9 Hz, 1H), 2.98 (s, 6H), 2.46 – 2.37 (m, 1H), 2.28 – 2.21 (m, 1H), 2.16 (dd, J = 6.1 and 5.0 Hz, 1H), 2.00 – 1.93 (m, 2H), 1.49 (s, 3H), 1.32 (s, 3H), 1.20 (d, J = 11.0 Hz, 1H), 0.89 (s, 3H). **^{13}C NMR** (151 MHz, $CDCl_3$) δ: 135.30, 134.67, 134.33, 131.13, 128.79, 126.84, 118.23, 86.72, 78.56, 54.34, 51.52, 39.66, 38.37, 35.62, 31.08, 28.84, 27.24, 26.66, 24.19. **^{11}B NMR** (193 MHz, $CDCl_3$) δ: 30.37. **HRMS (ESI) m/z**: $[M+H]^+$ calcd. for 457.2769 found $[M+H]^+$: 457.2762.

(3-((4-(4-(dimethylamino)phenyl)-1*H*-1,2,3-triazol-1-yl)methyl)phenyl)boronic acid (**11**)

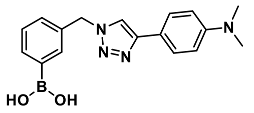

Synthesis according to the **general procedure 7, step 2**. The crude mixture was used without further purification to afford a red powder (53 mg, 85% yield). **^{1}H NMR** (600 MHz, MeOD) δ**:** 8.44 (s, 1H), 8.02 (d,

J = 7.8 Hz, 2H), 7.80-7.57 (m, 4H), 7.44-7.33 (m, 2H), 5.66 (s, 2H), 3.31 (d, J = 6.4 Hz, 9H, MeOH signal included). **^{13}C NMR** (151 MHz, MeOD) δ: δ 147.27, 143.87, 135.63, 135.04, 134.59, 133.07, 129.28, 128.55, 123.24, 121.93, 55.41, 47.04. **^{11}B NMR** (193 MHz, MeOD) δ: 28.26. **HRMS (ESI) m/z**: [M+H]$^+$ calcd. for 323.1673 found [M+H]$^+$: 323.1675.

methyl 3-(((1-(3-((3a*S*,4*R*,6*R*)-3a,5,5-trimethylhexahydro-4,6-methanobenzo[*d*][1,3,2]dioxaborol-2-yl) benzyl)-1*H*-1,2,3-triazol-4-yl)methyl)carbamoyl)benzoate (**12_P**)

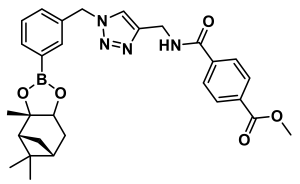

Synthesis according to the **general procedure 7, step 1**. The crude mixture was used without further purification to afford transparent crystals (80 mg, 94% yield). **^{1}H NMR** (600 MHz, $CDCl_3$) δ**:** 8.08 (d, J = 8.1 Hz, 2H), 7.84 (dd, J = 8.3 and 1.9 Hz, 2H), 7.81 (dt, J = 7.0 and 1.4 Hz, 1H), 7.77 (s, 1H), 7.41 – 7.36 (m, 2H), 5.51 (s, 2H), 4.81 – 4.61 (m, 2H), 4.45 (dd, J = 8.8 and 1.8 Hz, 1H), 3.93 (s, 3H), 2.45-2.40 (m, 1H), 2.25 – 2.21 (m, 1H), 2.14 (dd, J = 6.0 and 4.8 Hz, 1H), 1.98 – 1.93 (m, 2H), 1.48 (s, 3H), 1.31 (s, 3H), 1.26 (t, J = 7.1 Hz, 3H), 1.18 (d, J = 11.0 Hz, 1H), 0.89 (s, 3H). **^{13}C NMR** (151 MHz, $CDCl_3$) δ: 166.61, 137.91, 135.65, 134.87, 133.43, 133.03, 131.28, 129.99, 128.89, 127.30, 86.77, 78.58, 77.16, 52.59, 52.53, 51.51, 39.64, 38.36, 35.61, 28.83, 27.23, 26.66, 24.18. **^{11}B NMR** (193 MHz, $CDCl_3$) δ: 30.31. **HRMS (ESI) m/z**: [M+H]$^+$ calcd. for 529.2616 found [M+H]$^+$: 529.2612.

(3-((4-((3-(methoxycarbonyl)benzamido)methyl)-1*H*-1,2,3-triazol-1-yl)methyl)phenyl)boronic acid (**12**)

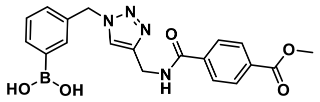

Synthesis according to the **general procedure 7, step 2**. The crude mixture was used without further purification to afford white powder (36 mg, 61% yield). **^{1}H NMR** (600 MHz, MeOD) δ**:** 8.11 – 8.07 (m, 2H), 7.94 – 7.89 (m, 3H), 7.74-7.58 (m, 2H), 7.42 – 7.30 (m, 2H), 5.60 (s, 2H), 4.64 (s, 2H), 3.94 (s, 3H). **^{13}C NMR** (151 MHz, MeOD) δ: 169.09, 167.67, 146.57, 139.47, 135.98, 135.10, 134.73, 134.63, 134.23, 134.07, 131.06, 130.61, 130.41, 129.29, 128.59, 124.40, 55.06, 52.87, 36.24. **^{11}B NMR** (193 MHz, MeOD) δ: 28.37. **HRMS (ESI) m/z**: [M+H]$^+$ calcd. For 395.1520 found [M+H]$^+$: 395.1514.

3-cyclohexyl-*N*-((1-(3-((3a*S*,4*R*,6*R*)-3a,5,5-trimethylhexahydro-4,6-methanobenzo[*d*][1,3,2]dioxaborol-2-yl)benzyl)-1*H*-1,2,3-triazol-4-yl)methyl)propanamide (**13_P**)

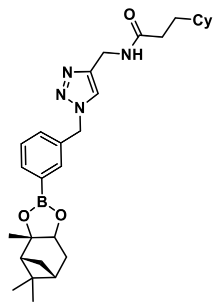

Synthesis according to the **general procedure 7, step 1**. The crude mixture was used without further purification to afford yellow oil (81 mg, 87% yield). **^{1}H NMR** (600 MHz, $CDCl_3$) δ**:** 7.81 (dt, J = 7.2 and 1.3 Hz, 1H), 7.77 (s, 1H), 7.43 (s, 1H), 7.41 – 7.33 (m, 2H), 6.12 (s, 1H), 5.49 (s, 2H), 4.57 – 4.33 (m, 3H), 2.45-2.40 (m, 1H), 2.24 (m, 1H), 2.21 – 2.13 (m, 3H), 2.01 – 1.93 (m, 2H), 1.66 (d, J = 9.6 Hz, 5H), 1.49 (s, 5H), 1.32 (s, 3H), 1.22 – 1.10 (m, 5H), 0.89 (s, 3H), 0.86 (dt, J = 12.1 and 6.6 Hz, 2H).**^{13}C NMR** (151 MHz, $CDCl_3$) δ: 173.57, 135.49, 134.81, 133.78, 131.20, 128.82, 86.74, 78.57, 77.37, 77.16, 76.95, 54.49, 51.52, 39.65, 38.37, 37.47, 35.62, 35.00, 34.22, 33.16, 33.09, 28.84, 27.24, 26.66, 26.35, 24.19. **^{11}B NMR** (193 MHz, $CDCl_3$) δ: 30.06. **HRMS (ESI) m/z**: $[M+H]^+$ calcd. for 505.3344 found $[M+H]^+$: 505.3346.

(3-((4-((3-cyclohexylpropanamido)methyl)-1*H*-1,2,3-triazol-1-yl)methyl)phenyl)boronic acid (**13**)

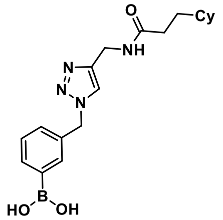

Synthesis according to the **general procedure 7, step 2**. The crude mixture was used without further purification to afford a yellow powder (33 mg, 56% yield). **^{1}H NMR** (600 MHz, MeOD) δ**:** 7.94 (s, 1H), 7.66 (s, 2H), 7.42 – 7.35 (m, 2H), 5.62 (s, 2H), 4.43 (s, 2H), 2.24 – 2.18 (m, 2H), 1.73 – 1.63 (m, 5H), 1.47 (dt, J = 9.2 and 6.7 Hz, 2H), 1.19 (dt, J = 15.0 and 9.8 Hz, 4H), 0.92 – 0.85 (m, 2H).**^{13}C NMR** (151 MHz, MeOD) δ: 176.67, 135.03, 134.62, 130.81, 129.33, 124.91, 55.63, 38.62, 35.25, 34.42, 34.30, 34.15, 27.63, 27.34. **^{11}B NMR** (193 MHz, MeOD) δ: 28.31. **HRMS (ESI) m/z**: $[M+H]^+$ calcd. for 371.2248 found $[M+H]^+$: 371.2246.

In situ click chemistry data

Multicomponent in situ click chemistry KPC-2

Table S1. *In situ* click chemistry data using KPC-2 as scaffold for catalysis. AC= Amplification Coefficient. In green the compounds selected from the screening.

Triazole	Mass Detected	rt 1,4-	rt 1,5-	Average PA No Protein	Average PA BSA	Average PA KPC-2	Regioselectivity (1,4:1,5)	AC
**Bor3_1**	303.1618	6.07	6.16	1.29E+05	7.22E+04	8.60E+04	50:50	0.67
**Bor3_2**	317.1779	6.90	7.10	2.91E+04	2.39E+04	7.47E+04	100:0	2.57
**Bor3_3**	311.0975	5.10	5.22	9.20E+03	1.44E+04	2.52E+04	16:84	1.75
**Bor3_4**	339.1290	6.09	5.73	3.26E+04	5.29E+04	1.12E+05	80:20	2.12
**Bor3_5**	276.1147	6.07	6.13	2.36E+04	2.76E+04	3.03E+04	100:0	1.10
**Bor3_6**	274.1719	8.48	8.60	N.F.	N.F.	N.F.	N.A.	N.A.
**Bor3_7**	290.1668	7.43	7.51	3.75E+03	1.31E+04	1.99E+04	100:0	1.52
**Bor3_8**	276.1512	6.14	6.22	N.F.	N.F.	N.F.	N.A.	N.A.
**Bor3_9**	260.1564	7.99	8.05	N.F.	N.F.	N.F.	N.A.	N.A.
**Bor3_10**	371.2245	8.66	8.51	N.F.	N.F.	N.F.	N.A.	N.A.
**Bor3_11**	343.1935	7.57	7.65	6.65E+04	1.03E+05	1.11E+05	90:10	1.08
**Bor3_12**	379.1605	7.37	7.45	2.36E+04	2.74E+04	4.46E+04	100:0	1.63
**Bor3_13**	337.1134	5.78	5.89	1.12E+04	1.98E+04	3.25E+04	100:0	1.64
**Bor3_14**	344.1774	8.51	8.51	N.F.	N.F.	N.F.	N.A.	N.A.
**Bor3_15**	274.1719	8.30	8.44	N.F.	N.F.	N.F.	N.A.	N.A.
**Bor3_16**	272.1562	8.07	7.95	N.F.	N.F.	N.F.	N.A.	N.A.
**Bor3_17**	286.1717	8.54	8.38	N.F.	N.F.	N.F.	N.A.	N.A.
**Bor3_18**	302.1667	7.27	7.66	N.F.	N.F.	N.F.	N.A.	N.A.
**Bor3_19**	343.1024	6.76	6.50	N.F.	N.F.	N.F.	N.A.	N.A.
**Bor3_20**	327.1258	6.25	5.92	1.17E+04	4.14E+03	1.61E+04	75:25	1.38
**Bor3_21**	415.0985	6.76	6.99	4.56E+04	1.53E+05	1.15E+05	75:25	0.75
**Bor3_22**	379.0694	6.59	6.75	7.12E+04	1.26E+05	1.40E+05	44:55	1.11
**Bor3_23**	344.0864	7.74	7.56	1.48E+04	5.63E+04	6.42E+04	51:49	1.14
**Bor3_24**	281.1198	5.37	5.54	N.F.	N.F.	N.F.	N.A.	N.A.
**Bor3_25**	282.1153	6.64	6.71	7.05E+04	8.38E+04	1.58E+05	100:0	1.88
**Bor3_26**	286.0812	7.60	7.58	N.F.	N.F.	N.F.	N.A.	N.A.
**Bor3_27**	331.1358	7.74	7.64	N.F.	N.F.	N.F.	N.A.	N.A.
**Bor3_28**	290.1424	3.75	3.21	2.66E+03	8.44E+03	1.01E+04	100:0	1.19
**Bor3_29**	291.1261	3.94	3.84	N.F.	N.F.	N.F.	N.A.	N.A.
**Bor3_30**	303.1623	4.03	3.80	5.02E+03	7.61E+03	6.95E+03	100:0	0.91
**Bor3_31**	323.1675	6.74	6.84	3.91E+04	1.21E+04	1.53E+04	100:0	0.39
**Bor3_32**	353.1417	6.74	6.78	2.00E+04	6.78E+04	2.67E+05	50:50	3.94
**Bor3_33**	301.183	5.40	5.73	N.F.	N.F.	N.F.	N.A.	N.A.
**Bor3_34**	247.1362	3.94	3.84	N.F.	N.F.	N.F.	N.A.	N.A.
**Bor3_35**	261.1519	3.78	3.84	5.82E+03	6.25E+03	1.62E+04	100:0	2.58
**Bor3_36**	345.2458	6.46	6.52	8.92E+04	1.05E+05	1.53E+05	100:0	1.45
**Bor3_37**	333.1355	5.15	4.90	N.F.	N.F.	N.F.	N.A.	N.A.
**Bor3_38**	347.1882	7.65	7.76	N.F.	N.F.	N.F.	N.A.	N.A.
**Bor3_39**	350.1018	6.58	6.66	2.05E+04	5.42E+03	7.39E+04	100:0	3.60
**Bor3_40**	423.2193	8.81	9.01	1.88E+04	1.97E+04	5.41E+04	100:0	2.75
**Bor3_41**	352.1108 (-)	7.53	7.26	1.27E+07	3.04E+06	3.42E+06	10:90	0.27
**Bor3_42**	370.102	7.80	7.52	N.F.	N.F.	N.F.	N.A.	N.A.
**Bor3_43**	248.083	5.12	5.32	N.F.	N.F.	N.F.	N.A.	N.A.
**Bor3_44**	324.1142	7.59	7.30	7.17E+04	7.58E+04	2.16E+05	100:0	2.85
**Bor3_45**	366.0909	6.85	6.48	N.F.	N.F.	N.F.	N.A.	N.A.
**Bor3_46**	351.1618	7.06	7.20	3.88E+04	1.79E+04	3.62E+04	80:20	0.93

**Bor3_47**	352.1574	7.23	7.10	N.F.	N.F.	N.F.	N.A.	N.A.
**Bor3_48**	322.1725	9.27	9.38	N.F.	N.F.	N.F.	N.A.	N.A.
**Bor3_49**	387.1291	7.40	7.48	1.80E+05	1.85E+05	4.27E+05	50:50	2.31
**Bor3_50**	310.1355	7.92	8.01	N.F.	N.F.	N.F.	N.A.	N.A.
**Bor3_51**	330.1402	8.71	9.03	N.F.	N.F.	N.F.	N.A.	N.A.
**Bor3_52**	280.125	7.90	8.12	N.F.	N.F.	N.F.	N.A.	N.A.
**Bor3_53**	356.1354	9.32	9.07	2.50E+07	2.91E+07	2.70E+07	100:0	0.93
**Bor3_54**	303.1055 (-)	7.75	7.91	N.F.	N.F.	N.F.	N.A.	N.A.
**Bor3_55**	351.1625	7.60	7.41	6.97E+03	2.15E+04	1.93E+04	50:50	0.90
**Bor3_56**	337.1465	7.28	7.17	N.F.	N.F.	N.F.	N.A.	N.A.
**Bor3_57**	445.1349	7.38	7.31	3.29E+04	3.61E+04	2.08E+05	50:50	5.77
**Bor3_58**	410.1102 (-)	6.66	6.87	N.F.	N.F.	N.F.	N.A.	N.A.
**Bor3_59**	355.1208	7.98	7.86	6.04E+04	3.59E+05	1.94E+05	50:50	0.54
**Bor3_60**	387.1294	7.38	7.46	1.26E+05	2.24E+05	3.44E+05	50:50	1.53
**Bor3_61**	295.136	5.65	6.18	N.F.	N.F.	N.F.	N.A.	N.A.
**Bor3_62**	322.1725	9.17	9.27	N.F.	N.F.	N.F.	N.A.	N.A.
**Bor3_63**	296.1198	7.22	7.48	N.F.	N.F.	N.F.	N.A.	N.A.
**Bor3_64**	355.1371	7.29	7.06	N.F.	N.F.	N.F.	N.A.	N.A.
**Bor3_65**	415.0756	8.02	7.73	N.F.	N.F.	N.F.	N.A.	N.A.
**Bor3_66**	345.0589	5.66	5.52	2.47E+04	2.85E+04	4.85E+04	100:0	1.70
**Bor3_67**	451.0247	7.80	7.90	2.35E+04	1.26E+05	2.32E+05	90:10	1.84
**Bor3_68**	421.1297	8.03	8.13	1.57E+04	6.98E+04	2.64E+04	50:50	0.38
**Bor3_69**	298.116	8.14	8.04	N.F.	N.F.	N.F.	N.A.	N.A.
**Bor3_70**	314.086	8.66	8.75	N.F.	N.F.	N.F.	N.A.	N.A.
**Bor3_71**	364.1075	8.88	8.71	N.F.	N.F.	N.F.	N.A.	N.A.
**Bor3_72**	358.0358	8.50	8.38	N.F.	N.F.	N.F.	N.A.	N.A.
**Bor3_73**	329.1023	6.26	6.33	N.F.	N.F.	N.F.	N.A.	N.A.
**Bor3_74**	333.1724	7.48	7.34	1.56E+04	1.16E+04	1.91E+04	100:0	1.22
**Bor3_75**	312.0819	4.99	4.84	N.F.	N.F.	N.F.	N.A.	N.A.
**Bor3_76**	326.1127	8.17	8.44	1.44E+04	1.68E+04	7.34E+03	50:50	0.51
**Bor3_77**	367.1572	7.16	7.24	4.35E+04	5.45E+04	6.14E+04	50:50	1.13
**Bor3_78**	248.1198	5.08	5.21	N.F.	N.F.	N.F.	N.A.	N.A.
**Bor3_79**	290.1667	6.73	6.82	3.61E+03	5.75E+03	5.99E+03	50:50	1.04
**Bor3_80**	290.1303	6.12	6.21	N.F.	N.F.	N.F.	N.A.	N.A.
**Bor3_81**	425.1987	8.64	8.49	3.48E+04	9.81E+05	4.75E+05	80:20	0.48
**Bor3_82**	275.1307	5.10	4.83	N.F.	N.F.	N.F.	N.A.	N.A.
**Bor3_83**	391.118	8.16	8.36	N.F.	N.F.	N.F.	N.A.	N.A.
**Bor3_84**	373.1132	7.13	6.92	N.F.	N.F.	N.F.	N.A.	N.A.
**Bor3_85**	379.0847	6.26	6.30	2.25E+04	2.83E+04	3.98E+04	80:20	1.41
**Bor3_86**	234.1046	4.76	4.86	N.F.	N.F.	N.F.	N.A.	N.A.
**Bor3_87**	325.1099	8.07	7.95	N.F.	N.F.	N.F.	N.A.	N.A.
**Bor3_88**	395.1521	7.43	7.24	1.36E+04	7.73E+03	4.04E+04	45:55	2.97
**Bor3_89**	398.203	10.40	10.51	N.F.	N.F.	N.F.	N.A.	N.A.
**Bor3_90**	336.1872	9.47	9.29	N.F.	N.F.	N.F.	N.A.	N.A.

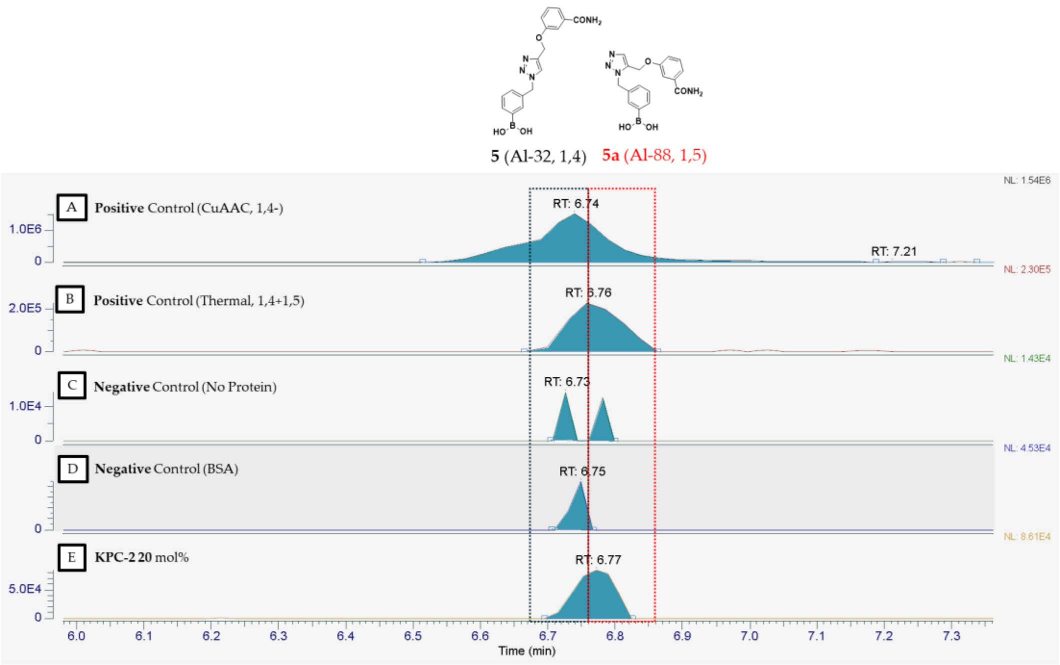

Figure S3. *In situ* click chemistry outcome from the reaction between warhead **3** and **Al-32.** HRMS (ESI) detected m/z: $[M+H]^+$: 353.1417**.** A) CuAAC (Positive control); B) Thermal reaction (Positive control) C) Reaction without catalyst; D) Reaction with BSA as catalyst; E) Reaction with KPC-2 as catalyst.

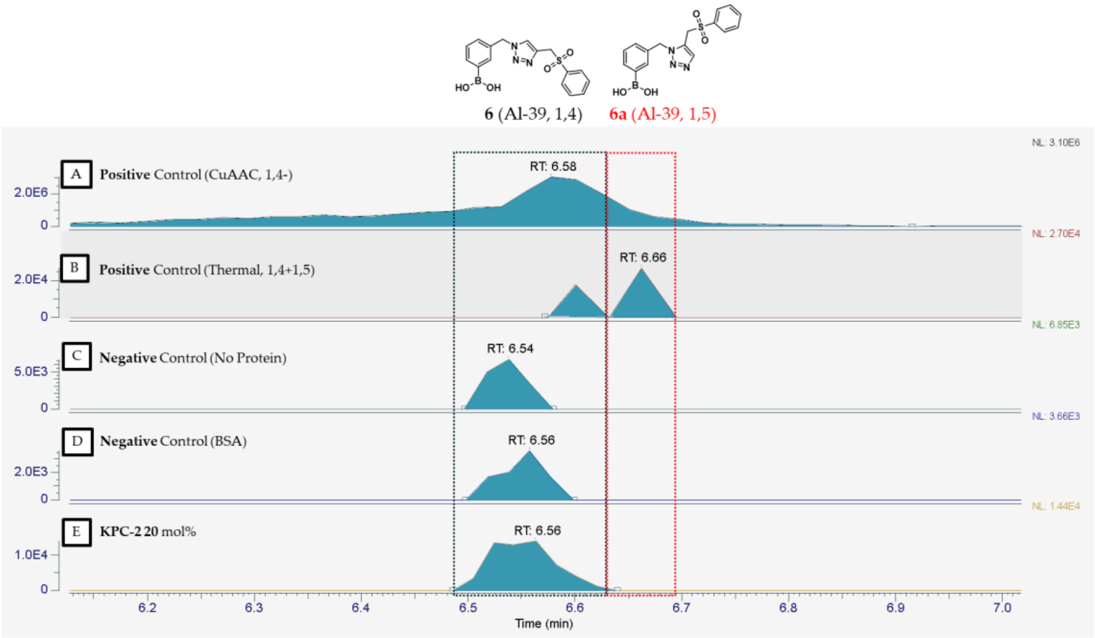

Figure S4. *In situ* click chemistry outcome from the reaction between warhead **3** and **Al-39.** HRMS (ESI) detected m/z: $[M+H]^+$: 358.1018**.** A) CuAAC (Positive control); B) Thermal reaction (Positive control) C) Reaction without catalyst; D) Reaction with BSA as catalyst; E) Reaction with KPC-2 as catalyst.

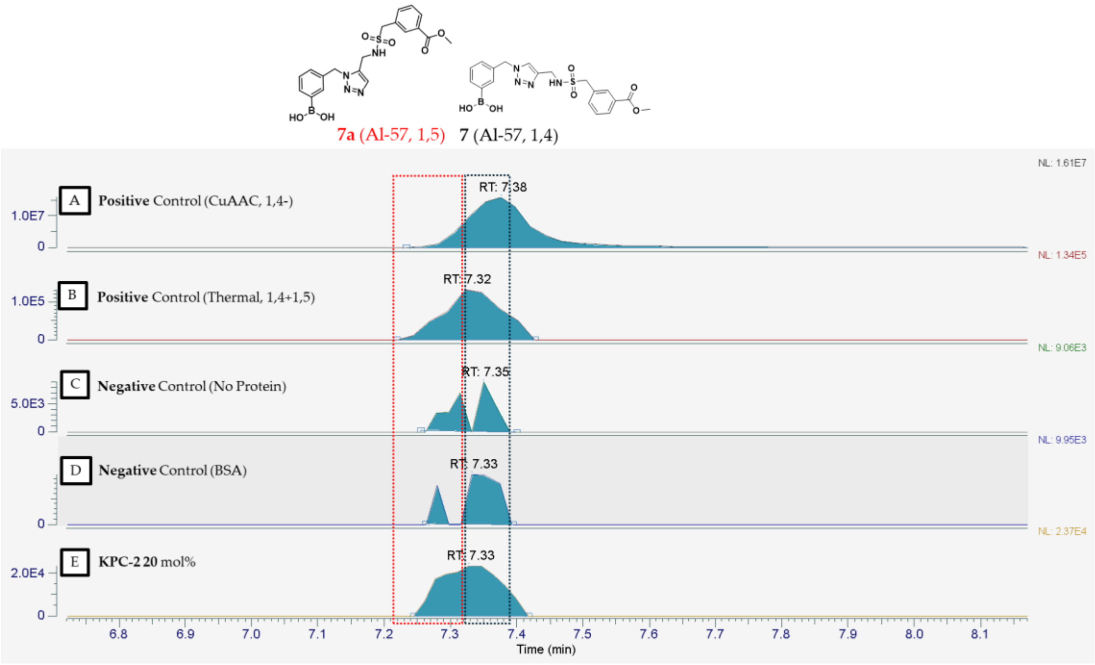

Figure S5. *In situ* click chemistry outcome from the reaction between warhead **3** and **Al-57**. HRMS (ESI) detected m/z: $[M+H]^+$: 445.1349**.** A) CuAAC (Positive control); B) Thermal reaction (Positive control) C) Reaction without catalyst; D) Reaction with BSA as catalyst; E) Reaction with KPC-2 as catalyst.

Optimisation on AmpC

To determine the appropriate catalyst loading for *in situ* click chemistry reactions, a binary experiment between warhead **3** and alkyne **Al-12** using different catalyst loadings of AmpC was performed.

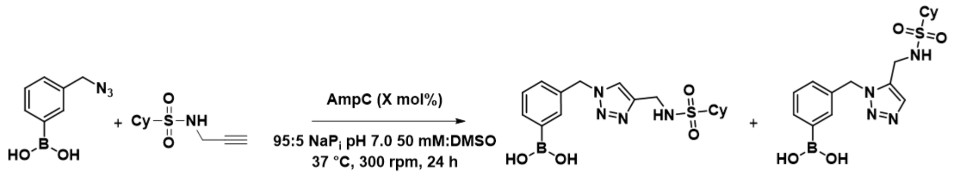

3 **Al-12**

Table S2. Catalyst loading determination using AmpC as scaffold for catalysis. AC= Amplification Coefficient. In green the conditions selected.

Entry*	Catalyst	Catalyst Loading (mol%)	Regioselectivity (**10** vs **10a**)	AC **10/10a**
1	No catalyst	-	50:50	-
2	BSA	20	60:40	-
3	AmpC	1	92:8	2-3
4		5	42:58	2-3
5		10	30:70	3-4
6		20	21:79	5-6
7		100	13:87	59-60

*Reactions were run in 0.2 mL microtubes with a total volume of 100 μL. Conditions: **3** (1 eq.), **Al-12** (5 eq.), 95:5 sodium phosphate 50 mM pH 7.0: DMSO; 300 rpm; 37 °C; 24 h.

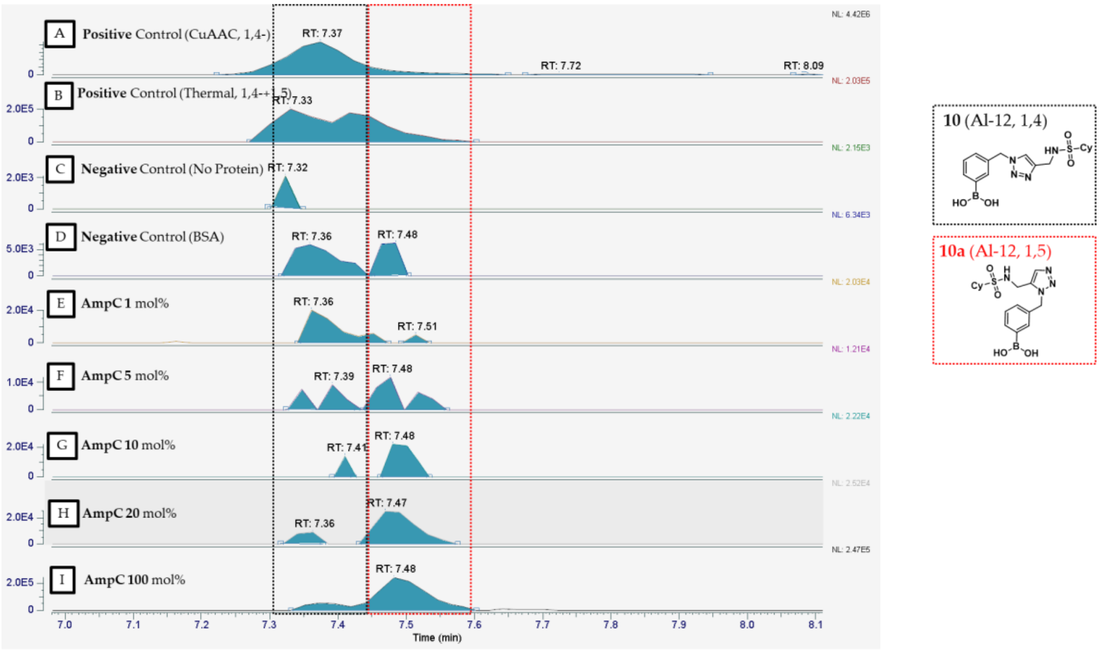

Figure S6. Binary *in situ* click chemistry results between warhead **3** and **Al-12**. HRMS (ESI) detected m/z: [M+H]+: 379.1605. A) CuAAC (Positive control); B) Thermal reaction (Positive control) C) Reaction without catalyst; D) Reaction with 20 mol% BSA as catalyst; E) Reaction with 1 mol% AmpC as catalyst; F) Reaction with 5 mol% AmpC as catalyst; G) Reaction with 10 mol% AmpC as catalyst; H) Reaction with 20 mol% AmpC as catalyst; I) Reaction with 100 mol% AmpC as catalyst.

Control experiment in presence of ***SM23***

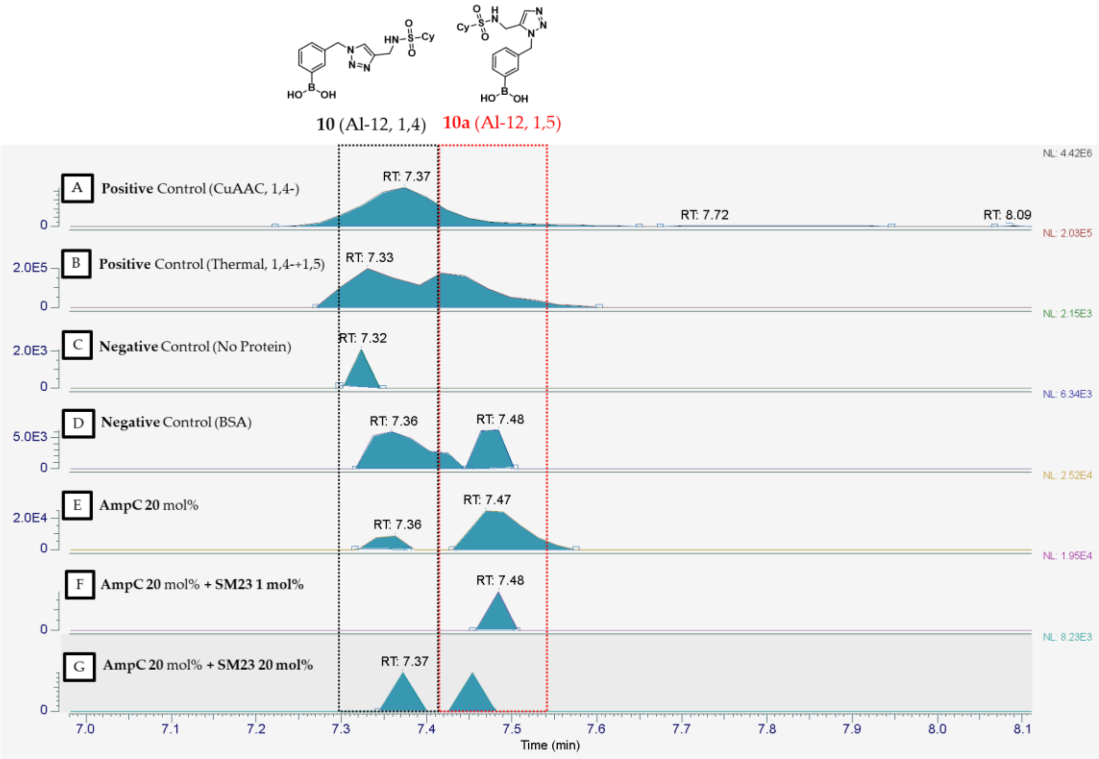

Figure S7. Binary *in situ* click chemistry results between warhead **3** and **Al-12** in presence of known BLIs **SM23**. HRMS (ESI) detected m/z: $[M+H]^+$: 379.1605. A) CuAAC (Positive control); B) Thermal reaction (Positive control) C) Reaction without catalyst; D) Reaction with BSA as catalyst; E) Reaction with 20 mol% AmpC as catalyst; F) Reaction with 20 mol% AmpC as catalyst and 1 mol% **SM23**; G) Reaction with 20 mol% AmpC as catalyst and 20 mol% **SM23**.

Multicomponent in situ click chemistry AmpC

Table S3. *In situ* click chemistry data using AmpC as scaffold for catalysis. AC= Amplification Coefficient. In green the compounds selected from the screening.

Triazole	Mass Detected	rt 1,4-	rt 1,5-	Average PA No Protein	Average PA BSA	Average PA AmpC	Regioselectivity (1,4:1,5)	AC
**Bor3_1**	303.1618	6.07	6.16	3.23E+04	4.01E+04	4.72E+04	58:43	1.18
**Bor3_2**	317.1779	6.90	7.10	8.62E+03	1.97E+04	4.68E+04	95:5	2.37
**Bor3_3**	311.0975	5.10	5.22	8.67E+03	1.62E+04	2.33E+04	55:45	1.43
**Bor3_4**	339.1290	6.09	5.73	2.82E+04	5.24E+04	8.81E+04	100:0	1.68
**Bor3_5**	276.1147	6.07	6.13	1.01E+04	1.40E+04	1.88E+04	100:0	1.34
**Bor3_6**	274.1719	8.48	8.60	N.F.	N.F.	N.F.	N.A.	N.A.
**Bor3_7**	290.1668	7.43	7.51	3.68E+03	1.00E+04	2.21E+04	19:81	2.20
**Bor3_8**	276.1512	6.14	6.22	N.F.	N.F.	N.F.	N.A.	N.A.
**Bor3_9**	260.1564	7.99	8.05	N.F.	N.F.	N.F.	N.A.	N.A.
**Bor3_10**	371.2245	8.66	8.51	3.22E+04	3.50E+04	3.53E+04	55:45	1.00
**Bor3_11**	343.1935	7.57	7.65	5.46E+03	5.83E+03	8.53E+03	60:40	1.46
**Bor3_12**	379.1605	7.37	7.45	0.00E+00	1.43E+04	7.88E+04	21:79	5.50
**Bor3_13**	337.1134	5.78	5.89	2.44E+03	4.65E+03	1.32E+04	100:0	2.85
**Bor3_14**	344.1774	8.51	8.51	N.F.	N.F.	N.F.	N.A.	N.A.
**Bor3_15**	274.1719	8.30	8.44	N.F.	N.F.	N.F.	N.A.	N.A.
**Bor3_16**	272.1562	8.07	7.95	N.F.	N.F.	N.F.	N.A.	N.A.
**Bor3_17**	286.1717	8.54	8.38	N.F.	N.F.	N.F.	N.A.	N.A.
**Bor3_18**	302.1667	7.27	7.66	N.F.	N.F.	N.F.	N.A.	N.A.
**Bor3_19**	343.1024	6.76	6.50	1.83E+04	2.23E+04	2.76E+04	24:76	1.24
**Bor3_20**	327.1258	6.25	5.92	7.25E+03	3.32E+03	5.43E+03	40:60	0.74
**Bor3_21**	415.0985	6.76	6.99	2.37E+04	3.98E+04	7.80E+04	100:0	1.95
**Bor3_22**	379.0694	6.59	6.75	3.96E+04	7.09E+04	1.18E+05	25:75	1.66
**Bor3_23**	344.0864	7.74	7.56	1.32E+04	5.15E+04	3.18E+04	82:18	0.61
**Bor3_24**	281.1198	5.37	5.54	N.F.	N.F.	N.F.	N.A.	N.A.
**Bor3_25**	282.1153	6.64	6.71	5.70E+04	8.05E+04	8.56E+04	100:0	1.06
**Bor3_26**	286.0812	7.60	7.58	N.F.	N.F.	N.F.	N.A.	N.A.
**Bor3_27**	331.1358	7.74	7.64	2.84E+04	6.51E+04	7.15E+04	50:50	1.09
**Bor3_28**	290.1424	3.75	3.21	N.F.	N.F.	N.F.	N.A.	N.A.
**Bor3_29**	291.1261	3.94	3.84	N.F.	N.F.	N.F.	N.A.	N.A.
**Bor3_30**	303.1623	4.03	3.80	N.F.	N.F.	N.F.	N.A.	N.A.
**Bor3_31**	323.1675	6.74	6.84	N.F.	N.F.	N.F.	N.A.	N.A.
**Bor3_32**	353.1417	6.74	6.78	4.73E+04	4.49E+04	1.19E+05	100:0	2.5
**Bor3_33**	301.183	5.40	5.73	N.F.	N.F.	N.F.	N.A.	N.A.
**Bor3_34**	247.1362	3.94	3.84	N.F.	N.F.	N.F.	N.A.	N.A.
**Bor3_35**	261.1519	3.78	3.84	1.11E+04	6.56E+03	1.05E+04	0:100	0.94
**Bor3_36**	345.2458	6.46	6.52	5.50E+04	4.93E+04	5.63E+04	100:0	1.14
**Bor3_37**	333.1355	5.15	4.90	2.80E+03	5.16E+03	4.11E+03	0:100	0.8
**Bor3_38**	347.1882	7.65	7.76	1.81E+05	2.12E+05	1.69E+05	66:41	0.92
**Bor3_39**	350.1018	6.58	6.66	1.60E+04	9.54E+03	1.84E+04	100:0	1.14
**Bor3_40**	423.2193	8.81	9.01	4.92E+04	2.16E+04	1.89E+05	0:100	3.85

**Bor3_41**	352.1108 (-)	7.53	7.26	1.38E+06	1.59E+06	1.58E+06	5:95	0.99
**Bor3_42**	370.102	7.80	7.52	1.37E+03	1.37E+04	1.57E+04	2:98	1.15
**Bor3_43**	248.083	5.12	5.32	N.F.	N.F.	N.F.	N.A.	N.A.
**Bor3_44**	324.1142	7.59	7.30	1.31E+04	1.07E+04	9.15E+03	45:55	0.70
**Bor3_45**	366.0909	6.85	6.48	N.F.	N.F.	N.F.	N.A.	N.A.
**Bor3_46**	351.1618	7.06	7.20	2.65E+04	1.95E+04	1.07E+04	80:20	0.40
**Bor3_47**	352.1574	7.23	7.10	1.32E+04	1.11E+04	2.27E+04	24:76	1.72
**Bor3_48**	322.1725	9.27	9.38	N.F.	N.F.	N.F.	N.A.	N.A.
**Bor3_49**	387.1291	7.40	7.48	8.94E+04	1.17E+05	2.85E+05	21:79	2.44
**Bor3_50**	310.1355	7.92	8.01	7.33E+03	4.35E+04	3.24E+04	55:45	0.74
**Bor3_51**	330.1402	8.71	9.03	6.50E+03	1.12E+04	1.91E+04	36:64	1.70
**Bor3_52**	280.125	7.90	8.12	N.F.	N.F.	N.F.	N.A.	N.A.
**Bor3_53**	356.1354	9.32	9.07	N.F.	N.F.	N.F.	N.A.	N.A.
**Bor3_54**	303.1055 (-)	7.75	7.91	N.F.	N.F.	N.F.	N.A.	N.A.
**Bor3_55**	351.1625	7.60	7.41	N.F.	N.F.	N.F.	N.A.	N.A.
**Bor3_56**	337.1465	7.28	7.17	6.41E+03	2.14E+04	2.67E+04	21:78	1.25
**Bor3_57**	445.1349	7.38	7.31	2.91E+04	3.47E+04	1.70E+05	50:50	4.88
**Bor3_58**	410.1102 (-)	6.66	6.87	N.F.	N.F.	N.F.	N.A.	N.A.
**Bor3_59**	355.1208	7.98	7.86	3.57E+04	2.14E+05	1.85E+05	100:0	0.86
**Bor3_60**	387.1294	7.38	7.46	9.68E+04	1.55E+05	2.58E+05	23:77	1.66
**Bor3_61**	295.136	5.65	6.18	6.55E+03	9.33E+03	1.62E+04	40:60	1.74
**Bor3_62**	322.1725	9.17	9.27	N.F.	N.F.	N.F.	N.A.	N.A.
**Bor3_63**	296.1198	7.22	7.48	N.F.	N.F.	N.F.	N.A.	N.A.
**Bor3_64**	355.1371	7.29	7.06	N.F.	N.F.	N.F.	N.A.	N.A.
**Bor3_65**	415.0756	8.02	7.73	1.57E+04	1.88E+04	1.04E+04	20:80	0.55
**Bor3_66**	345.0589	5.66	5.52	1.18E+04	9.17E+03	N.F.	N.A.	N.A.
**Bor3_67**	451.0247	7.80	7.90	4.15E+03	4.93E+04	5.63E+04	10:90	1.14
**Bor3_68**	421.1297	8.03	8.13	1.62E+04	3.31E+04	7.41E+04	0:100	2.24
**Bor3_69**	298.116	8.14	8.04	N.F.	N.F.	N.F.	N.A.	N.A.
**Bor3_70**	314.086	8.66	8.75	N.F.	N.F.	N.F.	N.A.	N.A.
**Bor3_71**	364.1075	8.88	8.71	N.F.	N.F.	N.F.	N.A.	N.A.
**Bor3_72**	358.0358	8.50	8.38	N.F.	N.F.	N.F.	N.A.	N.A.
**Bor3_73**	329.1023	6.26	6.33	2.18E+04	N.F.	1.85E+04	100:0	0.85
**Bor3_74**	333.1724	7.48	7.34	N.F.	N.F.	N.F.	N.A.	N.A.
**Bor3_75**	312.0819	4.99	4.84	N.F.	N.F.	N.F.	N.A.	N.A.
**Bor3_76**	326.1127	8.17	8.44	N.F.	N.F.	N.F.	N.A.	N.A.
**Bor3_77**	367.1572	7.16	7.24	3.38E+04	N.F.	8.45E+04	0:100	2.50
**Bor3_78**	248.1198	5.08	5.21	N.F.	N.F.	N.F.	N.A.	N.A.
**Bor3_79**	290.1667	6.73	6.82	N.F.	N.F.	N.F.	N.A.	N.A.
**Bor3_80**	290.1303	6.12	6.21	N.F.	N.F.	N.F.	N.A.	N.A.
**Bor3_81**	425.1987	8.64	8.49	N.F.	N.F.	N.F.	N.A.	N.A.
**Bor3_82**	275.1307	5.10	4.83	1.44E+04	1.03E+04	1.44E+04	39:61	1.00
**Bor3_83**	391.118	8.16	8.36	N.F.	N.F.	N.F.	N.A.	N.A.
**Bor3_84**	373.1132	7.13	6.92	N.F.	N.F.	N.F.	N.A.	N.A.
**Bor3_85**	379.0847	6.26	6.30	1.59E+04	1.07E+04	3.61E+04	100:0	2.26
**Bor3_86**	234.1046	4.76	4.86	N.F.	N.F.	N.F.	N.A.	N.A.
**Bor3_87**	325.1099	8.07	7.95	N.F.	N.F.	N.F.	N.A.	N.A.
**Bor3_88**	395.1521	7.43	7.24	2.36E+03	3.35E+03	1.32E+04	0:100	3.94
**Bor3_89**	398.203	10.40	10.51	N.F.	N.F.	N.F.	N.A.	N.A.
**Bor3_90**	336.1872	9.47	9.29	N.F.	N.F.	N.F.	N.A.	N.A.

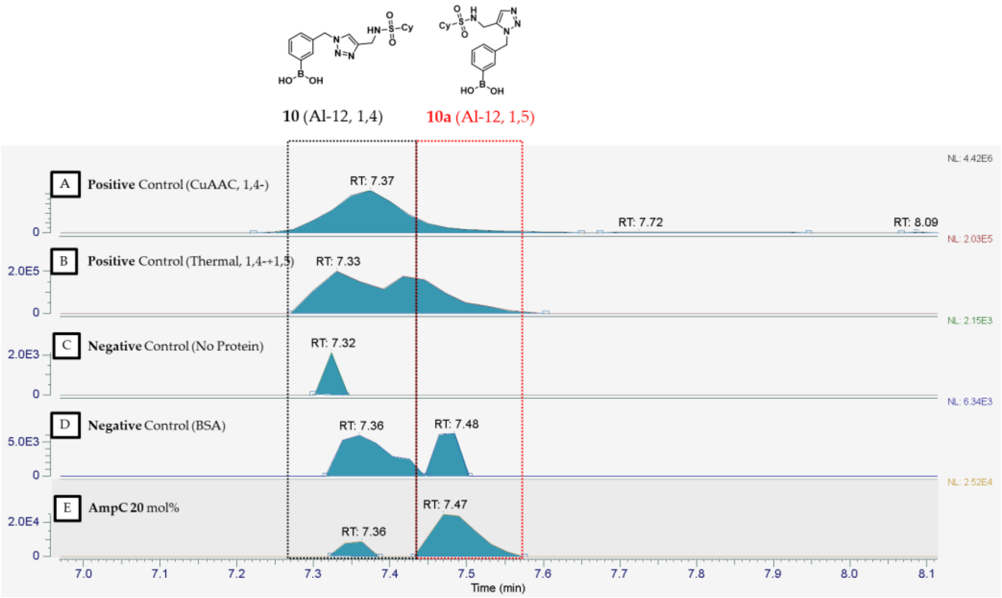

Figure S8. *In situ* click chemistry results between warhead **3** and **Al-12**. HRMS (ESI) detected m/z: $[M+H]^+$: 379.1605. A) CuAAC (Positive control); B) Thermal reaction (Positive control) C) Reaction without catalyst; D) Reaction with BSA as catalyst; E) Reaction with AmpC as catalyst.

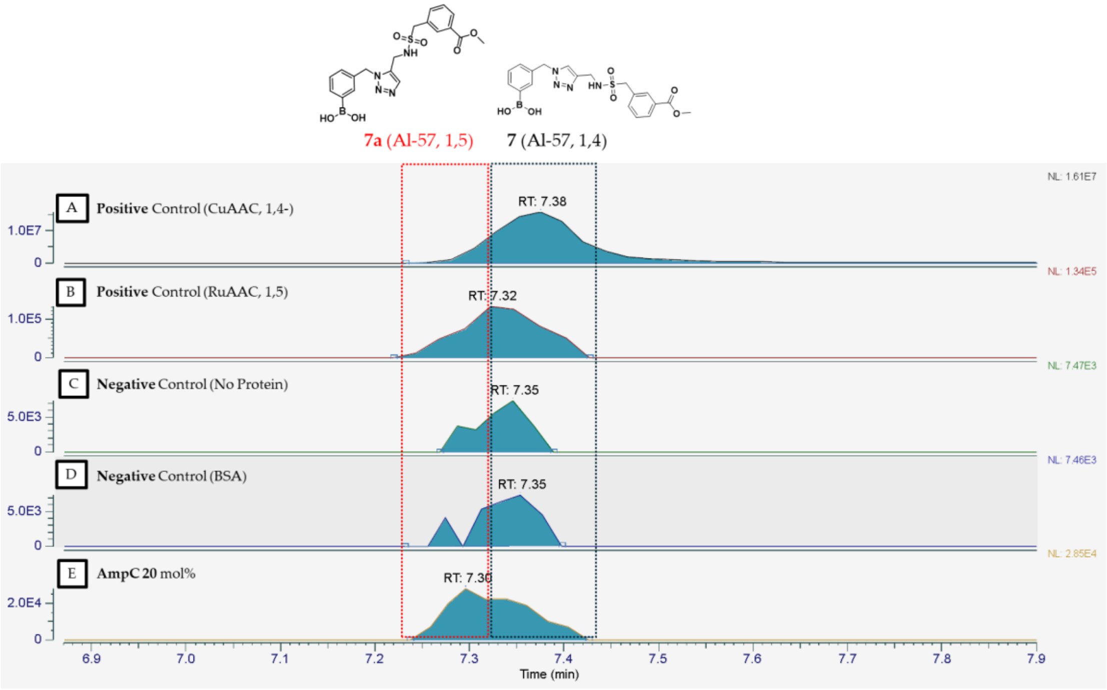

Figure S9. *In situ* click chemistry results between warhead **3** and **Al-57**. HRMS (ESI) detected m/z: $[M+H]^+$: 445.1349. A) CuAAC (Positive control); B) RuAAC (Positive control) C) Reaction without catalyst; D) Reaction with BSA as catalyst; E) Reaction with AmpC as catalyst.

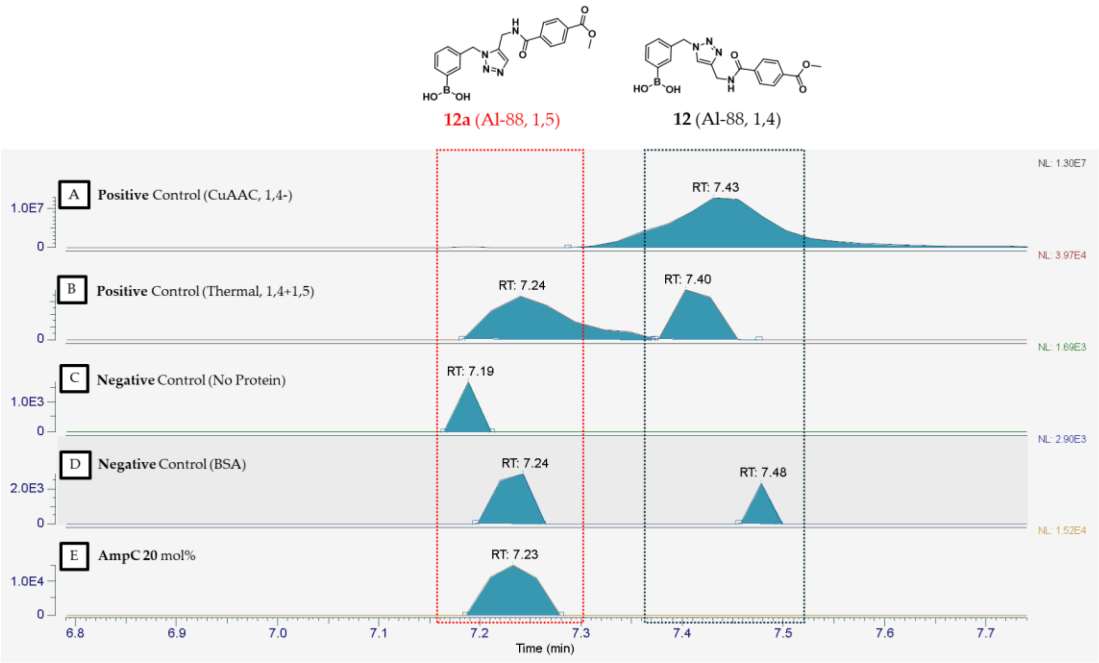

Figure S10. *In situ* click chemistry results between warhead **3** and **Al-88**. HRMS detected (ESI) m/z: $[M+H]^+$: 395.1521. A) CuAAC (Positive control); B) Thermal reaction (Positive control) C) Reaction without catalyst; D) Reaction with BSA as catalyst; E) Reaction with AmpC as catalyst.

DogSite Scorer analysis

Summary for β-lactamases binding pockets

Table S4. DogSiteScorer results for the pocket prediction of different β-lactamases (BL) in combination of boronic acids. Values coloured in red are not in compliance with values reported in previous KTGS examples. Green bold coloured values are in line with previously reported KTGS examples.

BL	*PDB*	*Ligand*	*Pocket*	*Volume (Å³)*	*Enclosure*	*Surface (Å²)*	*Depth*	*Surf / vol*	*H accept*	*H donor*	*Hydrop*	*Polar AA*	*Apolar AA*	*Simple Score*	*Dog Score*
**KTGS Pocket values**				800-1800	0.08-0.13	1000-2000	20-30	1.05-1.15	50-130	18-43	0.29-0.41	0.30-0.38	0.37-0.49	0.2-0.6	0.6-0.8
KPC-2 (A)	5EEC	S02030	P_1	336.90	0.15	412.14	11.03	1.22	30	12	0.47	0.58	0.16	0.21	0.45
	3RXX	3-PNBA	P_1	365.57	0.26	466.08	11.63	1.27	30	12	0.47	0.58	0.16	0.25	0.5
CTX-M-15 (A)	4XUZ	Vaborbactam	P_0	435.97	0.17	581.14	15.36	1.33	52	23	0.29	0.68	0.11	0.22	0.65
SHV-1 (A)	5EE8	S02030	P_2	274.75	0.17	303.74	15.52	1.10	31	10	0.31	0.41	0.32	0.08	0.62
NDM-1 (B)	6RFM	Taniborbactam	P_0	436.29	0.20	484.99	14.14	1.11	29	15	0.43	0.36	0.28	0.28	0.65
VIM-2 (B)	6SP7		P_1	720.19	0.12	894.8	16.11	1.24	41	19	0.50	0.37	0.34	0.52	0.78
AmpC (C)	3O88	CR167	P_0	1169.96	0.13	1479.98	29.42	1.26	89	32	0.34	0.38	0.39	0.62	0.81
	6YEN	Taniborbactam	P_0	934.08	0.09	1121.72	23.05	1.20	67	29	0.32	0.50	0.31	0.58	0.81
ADC-7 (C)	5WAE	CR167	P_2	735.49	0.11	735.47	23.82	0.99	57	26	0.22	0.48	0.40	0.42	0.85
MOX-1 (C)	5ZYB	S02030	P_0	642.05	0.10	642.73	17.18	1.00	48	24	0.26	0.51	0.34	0.37	0.78
OXA-24 (D)	8CUL	CR167	P_0	818.05	0.12	867.44	18.18	1.05	48	18	0.42	0.31	0.64	0.55	0.83
OXA-48 (D)	6V1O	Xeruborbactam	P_0	953.98	0.11	1160.56	22.27	1.21	69	32	0.36	0.34	0.51	0.61	0.82

Figures for Class A

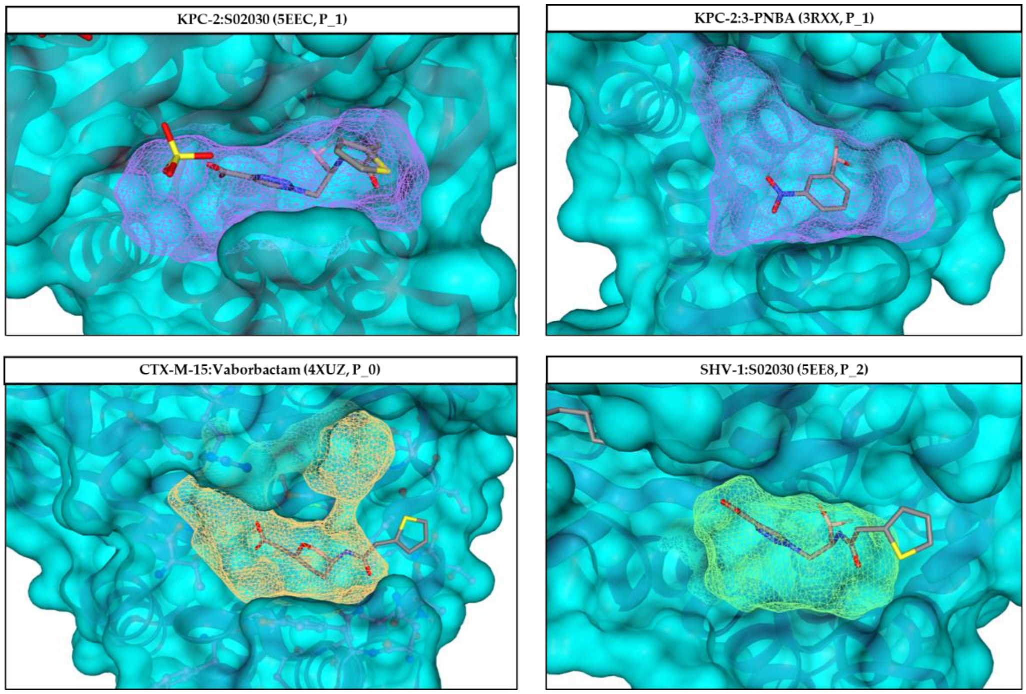

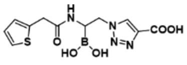

S02030

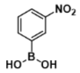

3-PNBA

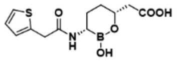

Vaborbactam

Figure S11. DoGSiteScorer analysis of the active sites of class A β-lactamases (KPC-2, CTX-M-15 and SHV-1) with bound ligands in the predicted subpockets (Coloured grids). Ligands are acyclic (**S02030**), phenyl (**3-PNBA**) and cyclic boronic acids (**Vaborbactam**).

Figures for Class B

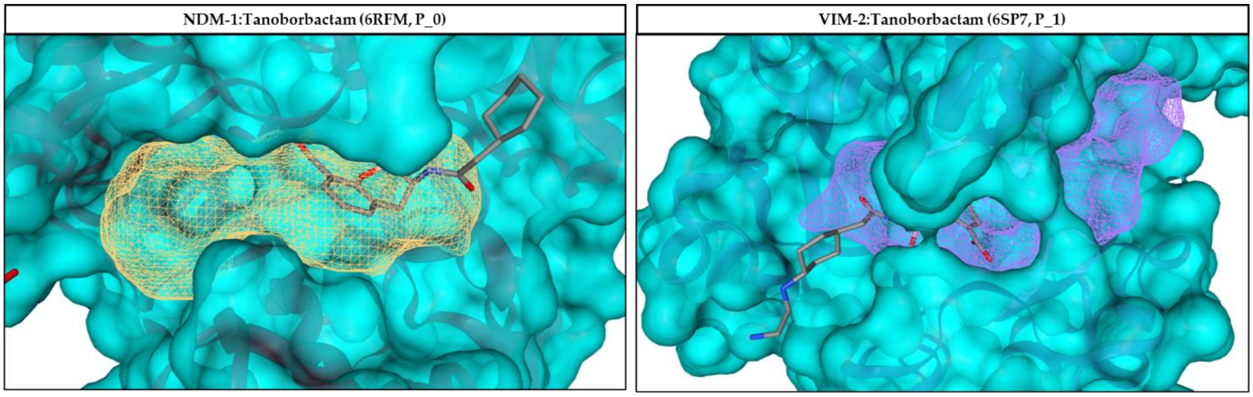

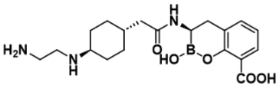

Taniborbactam

Figure S12. DoGSiteScorer analysis of the active sites of class B β-lactamases (NDM-1 and VIM-2) with bound ligands in the predicted subpockets (Coloured grids). Ligand is a cyclic boronic acid (**Taniborbactam**).

Figures for Class C

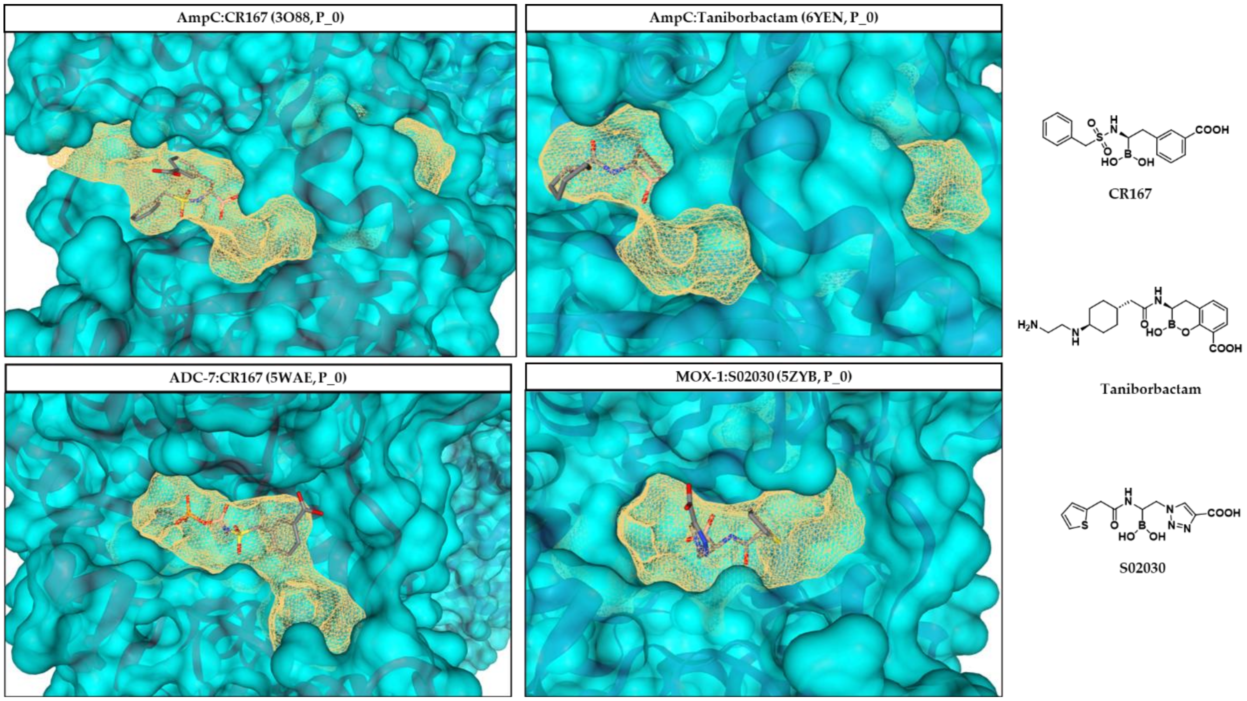

Figure S13. DoGSiteScorer analysis of the active sites of class C β-lactamases (AmpC, ADC-7 and MOX-1) with bound ligands in the predicted subpockets (Coloured grids). Ligands are acyclic (**S02030** and **CR167**) and cyclic boronic acids (**Taniborbactam**).

Figures for Class D

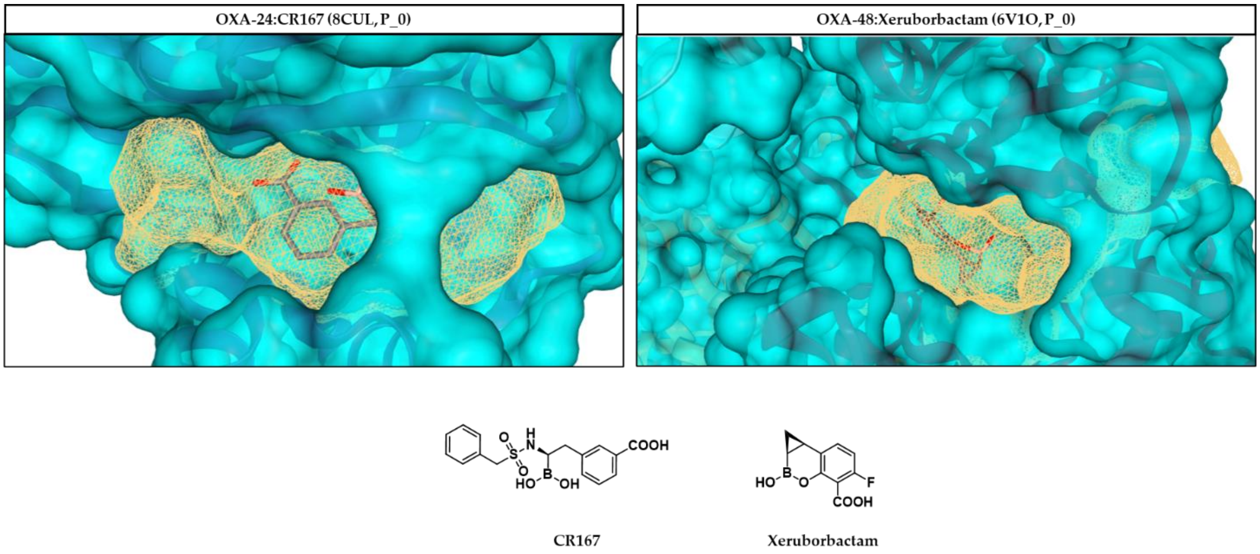

Figure S14. DoGSiteScorer analysis of the active sites of class D β-lactamases (OXA-24 and OXA-48) with bound ligands in the predicted subpockets (Coloured grids). Ligands are acyclic (**CR167**) and cyclic boronic acids (**Xeruborbactam**).

References

1. Caselli, E.; Romagnoli, C.; Vahabi, R.; Taracila, M.A.; Bonomo, R.A.; Prati, F. Click Chemistry in Lead Optimization of Boronic Acids as β-Lactamase Inhibitors. *Journal of Medicinal Chemistry* **2015**, *58*, 5445-5458, doi:10.1021/acs.jmedchem.5b00341.
2. Naumov, M.I.; Nuchev, A.V.; Sitnikov, N.S.; Malysheva, Y.B.; Shavyrin, A.S.; Beletskaya, I.P.; Gavryushin, A.E.; Combes, S.; Fedorov, A.Y. 2-(Azidomethyl)arylboronic Acids in the Synthesis of Coumarin-Type Compounds. *Synthesis* **2009**, *2009*, 1673-1682, doi:10.1055/s-0028-1088058.
3. Zhou, J.; Stapleton, P.; Haider, S.; Healy, J. Boronic acid inhibitors of the class A β-lactamase KPC-2. *Bioorganic & Medicinal Chemistry* **2018**, *26*, 2921-2927, doi:https://doi.org/10.1016/j.bmc.2018.04.055.
4. Deprez-Poulain, R.; Hennuyer, N.; Bosc, D.; Liang, W.G.; Enée, E.; Marechal, X.; Charton, J.; Totobenazara, J.; Berte, G.; Jahklal, J.; et al. Catalytic site inhibition of insulin-degrading enzyme by a small molecule induces glucose intolerance in mice. *Nature Communications* **2015**, *6*, 8250, doi:10.1038/ncomms9250.
5. Hirsch, A.K.H.; Lauw, S.; Gersbach, P.; Schweizer, W.B.; Rohdich, F.; Eisenreich, W.; Bacher, A.; Diederich, F. Nonphosphate Inhibitors of IspE Protein, a Kinase in the Non-Mevalonate Pathway for Isoprenoid Biosynthesis and a Potential Target for Antimalarial Therapy. *ChemMedChem* **2007**, *2*, 806-810, doi:https://doi.org/10.1002/cmdc.200700014.
6. Zhou, Y.; Wang, L.; Li, S.; Ma, S.; Walsh, P.J.; Bian, Q.; Li, F.; Wang, M.; Zhong, J. Enantioselective Addition of Alkynyl Esters and Ethers to Aldehydes Catalyzed by a Cyclopropyl Amino Alcohol Based Zinc Catalyst. *Synlett* **2020**, *31*, 60-64, doi:10.1055/s-0039-1690264.
7. Singh, G.; Rani, S. An expedient 'click' approach for the synthetic evaluation of ester-triazole-tethered organosilica conjugates. *Applied Organometallic Chemistry* **2018**, *32*, e4028, doi:https://doi.org/10.1002/aoc.4028.
8. Nikolayevskiy, H.; Robello, M.; Scerba, M.T.; Pasternak, E.H.; Saha, M.; Hartman, T.L.; Buchholz, C.A.; Buckheit, R.W.; Durell, S.R.; Appella, D.H. The structure-activity profile of mercaptobenzamides' anti-HIV activity suggests that thermodynamics of metabolism is more important than binding affinity to the target. *European Journal of Medicinal Chemistry* **2019**, *178*, 818-837, doi:https://doi.org/10.1016/j.ejmech.2019.06.020.
9. Clements, M.; Blackie, M.; de Kock, C.; Lawrence, N.; Smith, P.; Roex, T.l. Investigation into the Structures and Properties of Multicomponent Crystals Formed from a Series of 7-Chloroquinolines and Aromatic Acids. *Crystal Growth & Design* **2019**, *19*, 1540-1549, doi:10.1021/acs.cgd.8b01049.
10. Riva, B.; Griglio, A.; Serafini, M.; Cordero-Sanchez, C.; Aprile, S.; Di Paola, R.; Gugliandolo, E.; Alansary, D.; Biocotino, I.; Lim, D.; et al. Pyrtriazoles, a Novel Class of Store-Operated Calcium Entry Modulators: Discovery, Biological Profiling, and in Vivo Proof-of-Concept Efficacy in Acute Pancreatitis. *Journal of Medicinal Chemistry* **2018**, *61*, 9756-9783, doi:10.1021/acs.jmedchem.8b01512.
11. Jung, Y.-H.; Jain, S.; Gopinatth, V.; Phung, N.B.; Gao, Z.-G.; Jacobson, K.A. Structure activity relationship of 3-nitro-2-(trifluoromethyl)-2H-chromene derivatives as P2Y6 receptor antagonists. *Bioorganic & Medicinal Chemistry Letters* **2021**, *41*, 128008, doi:https://doi.org/10.1016/j.bmcl.2021.128008.
12. Wellhöfer, I.; Frydenvang, K.; Kotesova, S.; Christiansen, A.M.; Laursen, J.S.; Olsen, C.A. Functionalized Helical β-Peptoids. *The Journal of Organic Chemistry* **2019**, *84*, 3762-3779, doi:10.1021/acs.joc.9b00218.
13. Achard, T.; Lepronier, A.; Gimbert, Y.; Clavier, H.; Giordano, L.; Tenaglia, A.; Buono, G. A Regio- and Diastereoselective Platinum-Catalyzed Tandem [2+1]/[3+2] Cycloaddition Sequence. *Angewandte Chemie International Edition* **2011**, *50*, 3552-3556, doi:https://doi.org/10.1002/anie.201007992.
14. Ko, E.; Liu, J.; Perez, L.M.; Lu, G.; Schaefer, A.; Burgess, K. Universal Peptidomimetics. *Journal of the American Chemical Society* **2011**, *133*, 462-477, doi:10.1021/ja1071916.
15. Segal, M.; Avinery, R.; Buzhor, M.; Shaharabani, R.; Harnoy, A.J.; Tirosh, E.; Beck, R.; Amir, R.J. Molecular Precision and Enzymatic Degradation: From Readily to Undegradable Polymeric Micelles by Minor Structural Changes. *Journal of the American Chemical Society* **2017**, *139*, 803-810, doi:10.1021/jacs.6b10624.
16. Wang, J.L.; Deutsch, E.C.; Oya, S.; Kung, H.F. FlipADAM: a potential new SPECT imaging agent for the serotonin transporter. *Nuclear Medicine and Biology* **2010**, *37*, 577-586, doi:https://doi.org/10.1016/j.nucmedbio.2010.02.010.
17. Krasavin, M.; Lukin, A.; Vedekhina, T.; Manicheva, O.; Dogonadze, M.; Vinogradova, T.; Zabolotnykh, N.; Rogacheva, E.; Kraeva, L.; Yablonsky, P. Conjugation of a 5-nitrofuran-2-oyl moiety to aminoalkylimidazoles produces non-toxic nitrofurans that are efficacious in vitro and in vivo against multidrug-resistant Mycobacterium tuberculosis. *European Journal of Medicinal Chemistry* **2018**, *157*, 1115-1126, doi:https://doi.org/10.1016/j.ejmech.2018.08.068.
18. Casnati, A.; Perrone, A.; Mazzeo, P.P.; Bacchi, A.; Mancuso, R.; Gabriele, B.; Maggi, R.; Maestri, G.; Motti, E.; Stirling, A.; et al. Synthesis of Imidazolidin-2-ones and Imidazol-2-ones via Base-Catalyzed Intramolecular Hydroamidation of Propargylic Ureas under Ambient Conditions. *The Journal of Organic Chemistry* **2019**, *84*, 3477-3490, doi:10.1021/acs.joc.9b00064.
19. Nguyen, T.-A.; Roger, J.; Nasrallah, H.; Rampazzi, V.; Fournier, S.; Cattey, H.; Sosa Carrizo, E.D.; Fleurat-Lessard, P.; Devillers, C.H.; Pirio, N.; et al. Gold(I) Complexes Nuclearity in Constrained Ferrocenyl Diphosphines: Dramatic Effect in Gold-Catalyzed Enyne Cycloisomerization. *Chemistry – An Asian Journal* **2020**, *15*, 2879-2885, doi:https://doi.org/10.1002/asia.202000579.

20. Jordan, R.S.; Li, Y.L.; Lin, C.-W.; McCurdy, R.D.; Lin, J.B.; Brosmer, J.L.; Marsh, K.L.; Khan, S.I.; Houk, K.N.; Kaner, R.B.; et al. Synthesis of N = 8 Armchair Graphene Nanoribbons from Four Distinct Polydiacetylenes. *Journal of the American Chemical Society* **2017**, *139*, 15878-15890, doi:10.1021/jacs.7b08800.
21. Liu, Y.; Huang, Y.; Song, H.; Liu, Y.; Wang, Q. Regio- and Chemoselective N-1 Acylation of Indoles: Pd-Catalyzed Domino Cyclization to Afford 1,2-Fused Tricyclic Indole Scaffolds. *Chemistry – A European Journal* **2015**, *21*, 5337-5340, doi:https://doi.org/10.1002/chem.201406617.
22. Marcyk, P.T.; Cook, S.P. Iron-Catalyzed Hydroamination and Hydroetherification of Unactivated Alkenes. *Organic Letters* **2019**, *21*, 1547-1550, doi:10.1021/acs.orglett.9b00427.
23. Wilson, K.L.; Murray, J.; Jamieson, C.; Watson, A.J.B. Cyrene as a bio-based solvent for HATU mediated amide coupling. *Organic & Biomolecular Chemistry* **2018**, *16*, 2851-2854, doi:10.1039/C8OB00653A.
24. He, Z.; Chen, Y.; Wang, Y.; Wang, J.; Mo, J.; Fu, B.; Wang, Z.; Du, Y.; Zhou, X. A rapidly photo-activatable light-up fluorescent nucleoside and its application in DNA base variation sensing. *Chemical Communications* **2016**, *52*, 8545-8548, doi:10.1039/C6CC03098J.
25. Trabbic, C.J.; Zhang, F.; Walseth, T.F.; Slama, J.T. Nicotinic Acid Adenine Dinucleotide Phosphate Analogues Substituted on the Nicotinic Acid and Adenine Ribosides. Effects on ReceptorMediated Ca2+ Release. *Journal of Medicinal Chemistry* **2015**, *58*, 3593-3610, doi:10.1021/acs.jmedchem.5b00279.
26. Sousa, S.C.A.; Bernardo, J.R.; Wolff, M.; Machura, B.; Fernandes, A.C. Oxo-Rhenium(V) Complexes Containing Heterocyclic Ligands as Catalysts for the Reduction of Sulfoxides. *European Journal of Organic Chemistry* **2014**, *2014*, 1855-1859, doi:https://doi.org/10.1002/ejoc.201301057.
27. Joolakanti, H.B.; Kamepalli, R.; Miryala, J.; Battu, S. Synthesis, Docking, and Biological activities of novel Metacetamol embedded [1,2,3]-triazole derivatives. *Journal of Molecular Structure* **2021**, *1242*, 130786, doi:https://doi.org/10.1016/j.molstruc.2021.130786.
28. Siles, R.; Kawasaki, Y.; Ross, P.; Freire, E. Synthesis and biochemical evaluation of triazole/tetrazole-containing sulfonamides against thrombin and related serine proteases. *Bioorganic & Medicinal Chemistry Letters* **2011**, *21*, 5305-5309, doi:https://doi.org/10.1016/j.bmcl.2011.07.023.
29. Thirupataiah, B.; Mounika, G.; Reddy, G.S.; Kumar, J.S.; Hossain, K.A.; Medishetti, R.; Samarpita, S.; Rasool, M.; Mudgal, J.; Mathew, J.E.; et al. PdCl2-catalyzed synthesis of a new class of isocoumarin derivatives containing aminosulfonyl / aminocarboxamide moiety: First identification of a isocoumarin based PDE4 inhibitor. *European Journal of Medicinal Chemistry* **2021**, *221*, 113514, doi:https://doi.org/10.1016/j.ejmech.2021.113514.
30. Jiang, C.-s.; Wang, X.-m.; Zhang, S.-q.; Meng, L.-s.; Zhu, W.-h.; Xu, J.; Lu, S.-m. Discovery of 4-benzoylamino-N-(prop-2-yn-1-yl)benzamides as novel microRNA-21 inhibitors. *Bioorganic & Medicinal Chemistry* **2015**, *23*, 6510-6519, doi:https://doi.org/10.1016/j.bmc.2015.08.007.

NMR Spectra for Azides

*¹H NMR Spectrum of **1***

600 MHz, d_6-DMSO

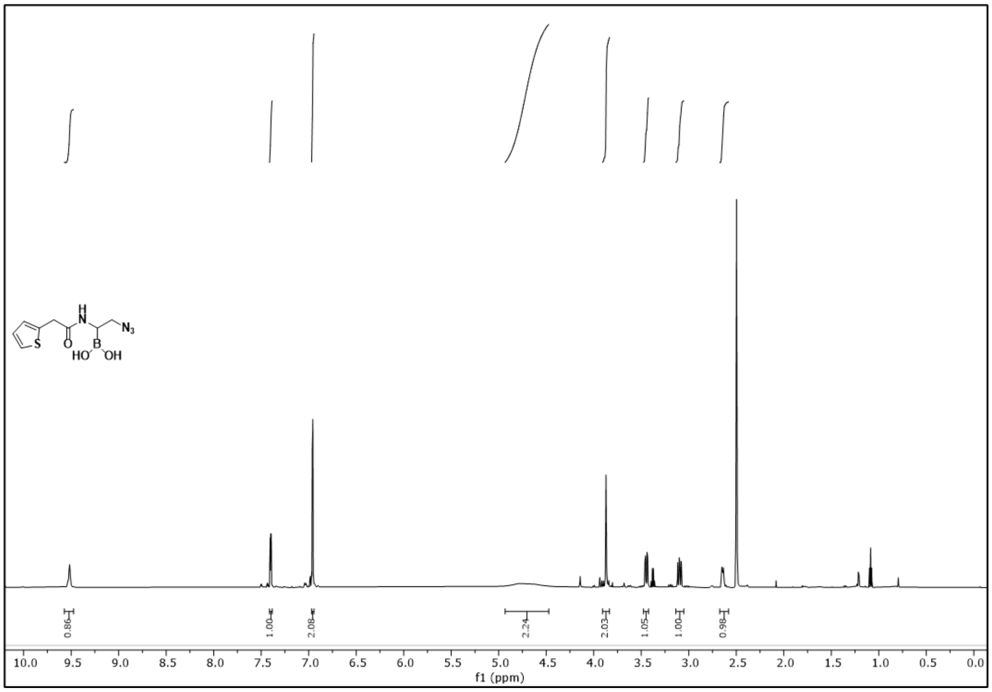

*¹³C NMR Spectrum of **1***

151 MHz, d_6-DMSO

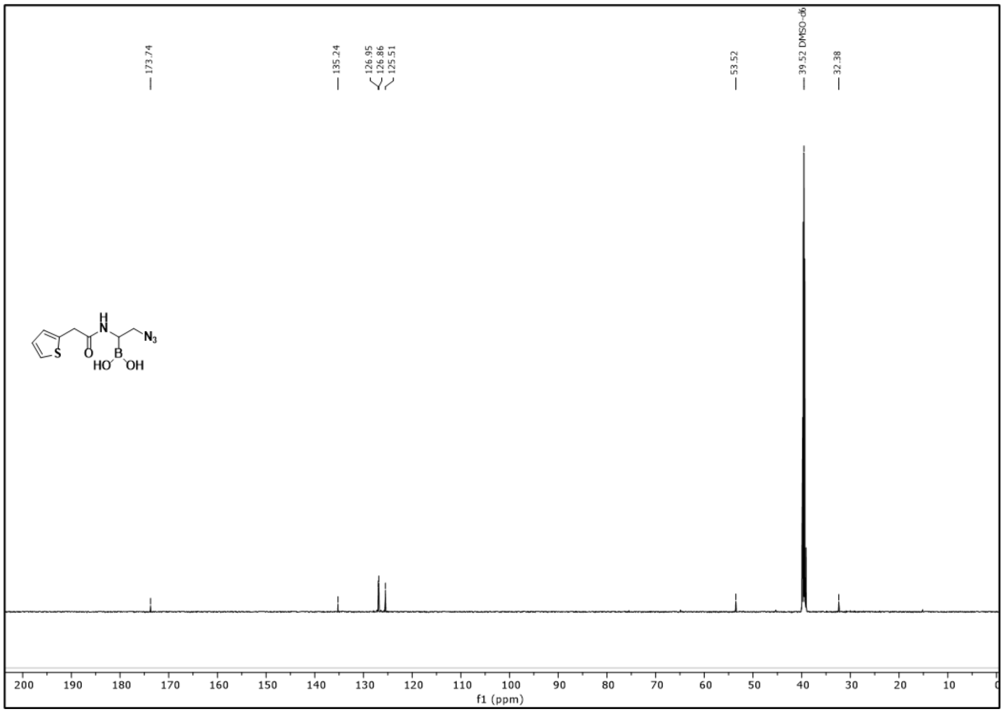

^{1}H *NMR Spectrum of* **2**

400 MHz, D_2O

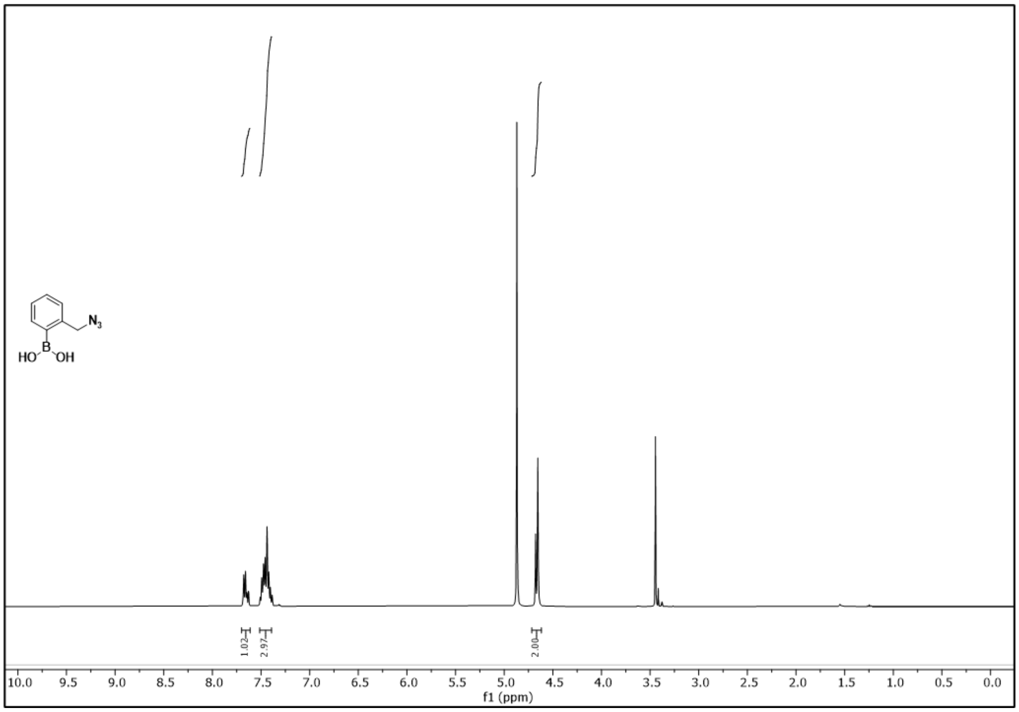

^{1}H *NMR Spectrum of* **3**

400 MHz, MeOD

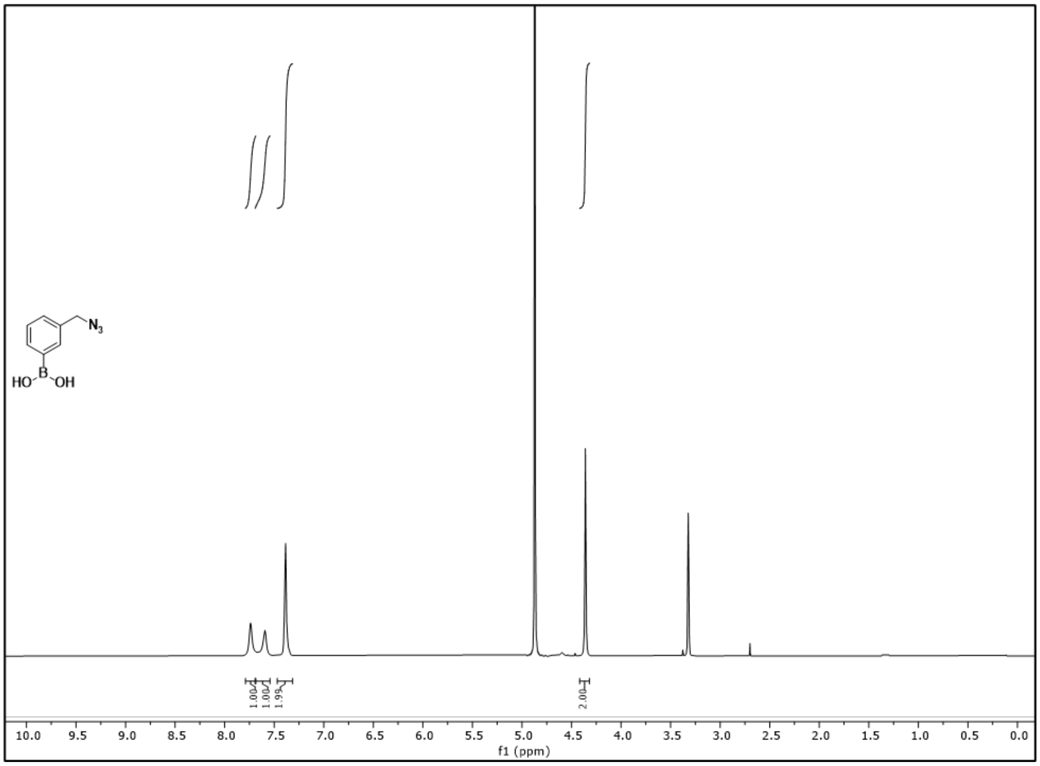

¹H NMR Spectrum of ***4***

400 MHz, D_2O

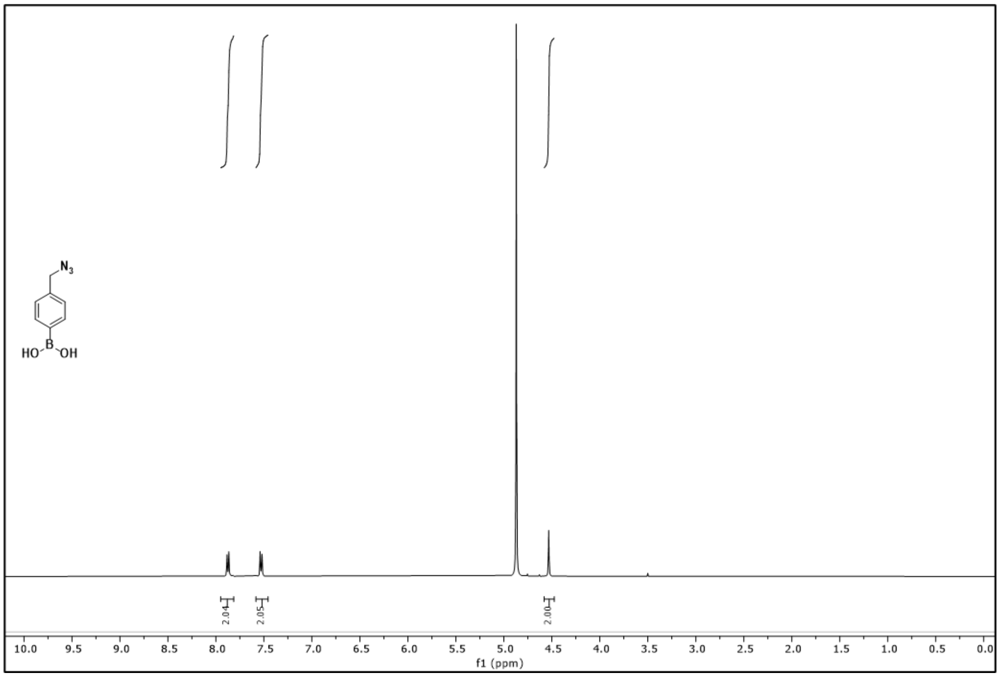

NMR Spectra for Alkynes

^{1}H NMR Spectrum of ***Al-1***

400 MHz, $CDCl_3$

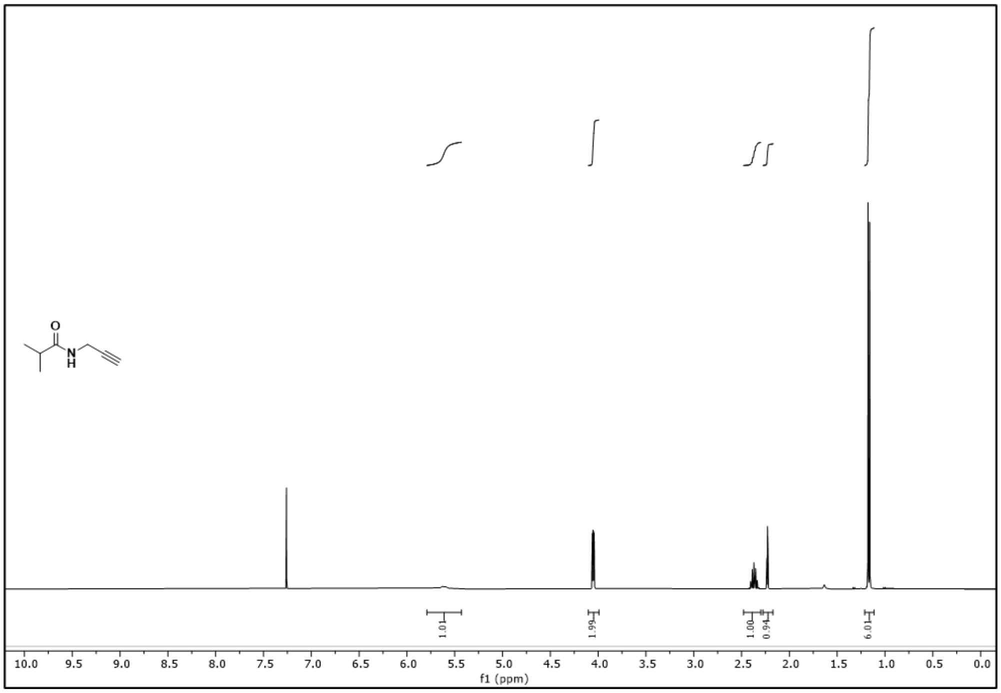

^{1}H NMR Spectrum of ***Al-2***

400 MHz, $CDCl_3$

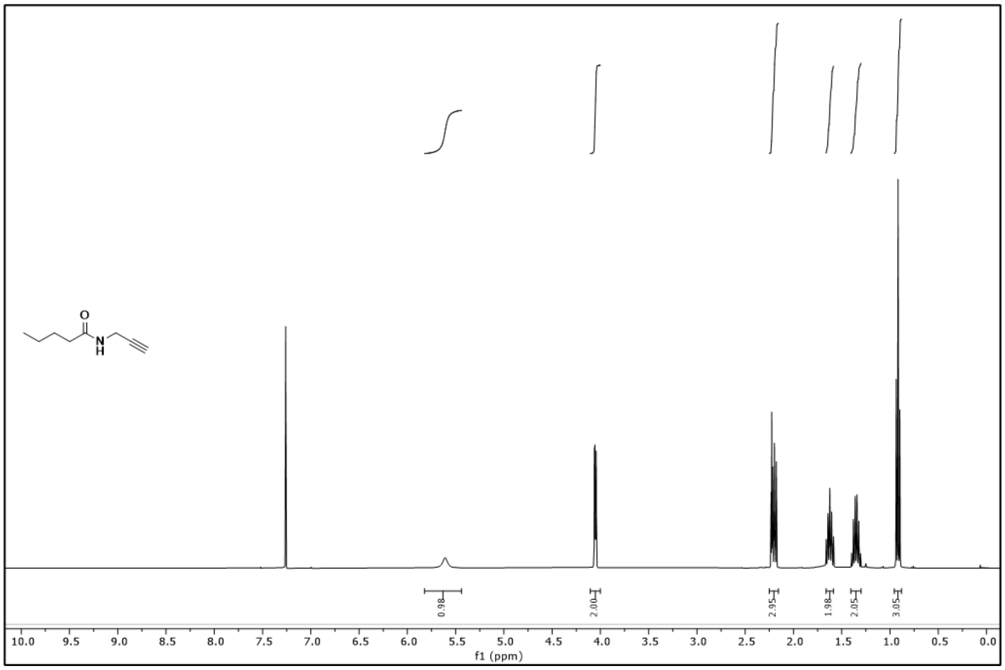

¹H NMR Spectrum of ***Al-3***

400 MHz, $CDCl_3$

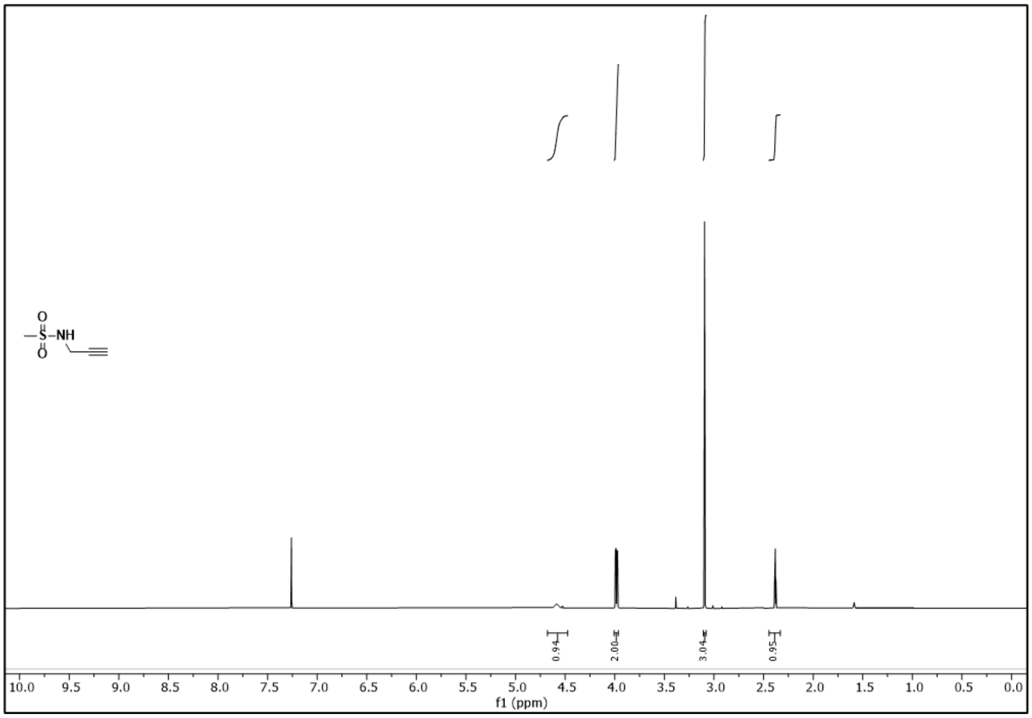

¹H NMR Spectrum of ***Al-4***

400 MHz, $CDCl_3$

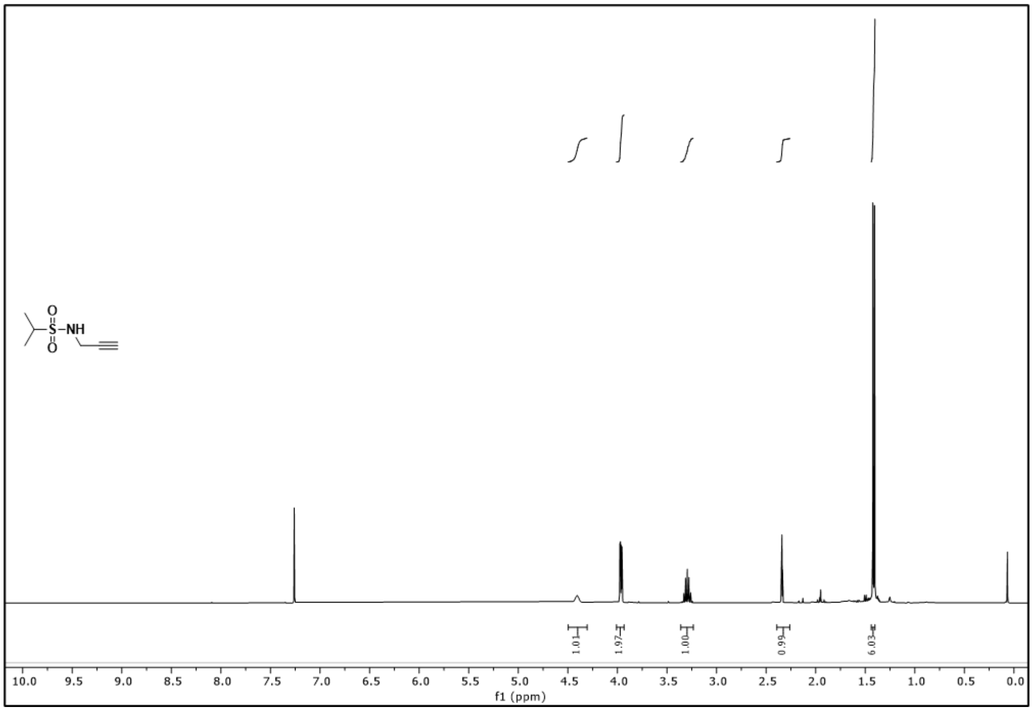

¹H NMR Spectrum of ***Al-10***

400 MHz, $CDCl_3$

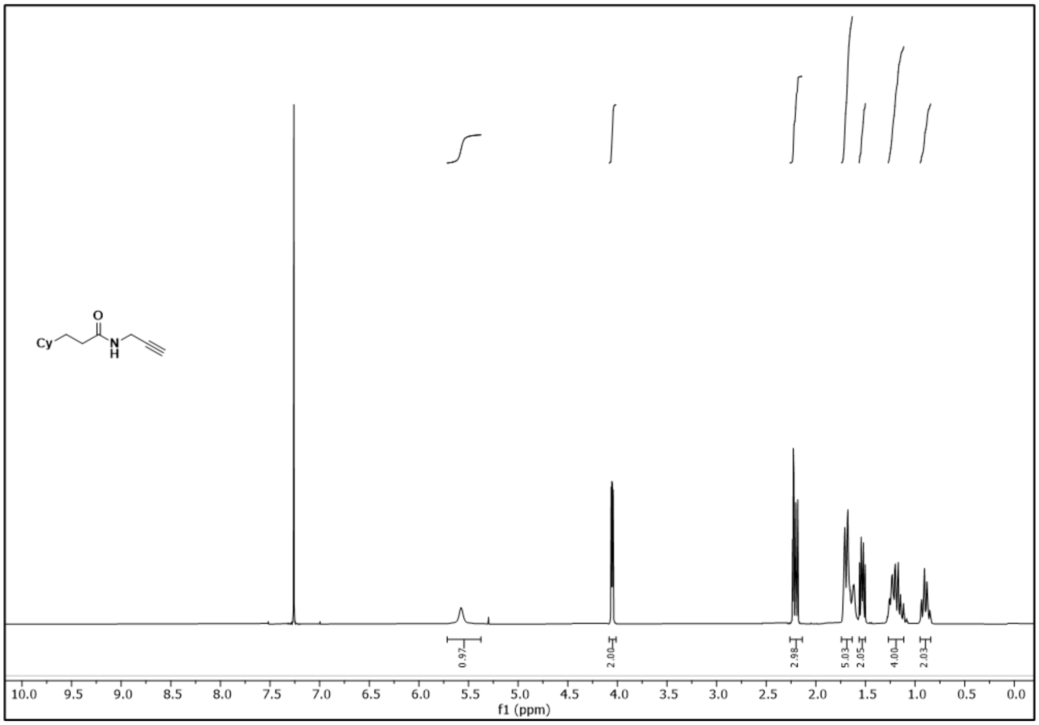

¹H NMR Spectrum of ***Al-11***

400 MHz, $CDCl_3$

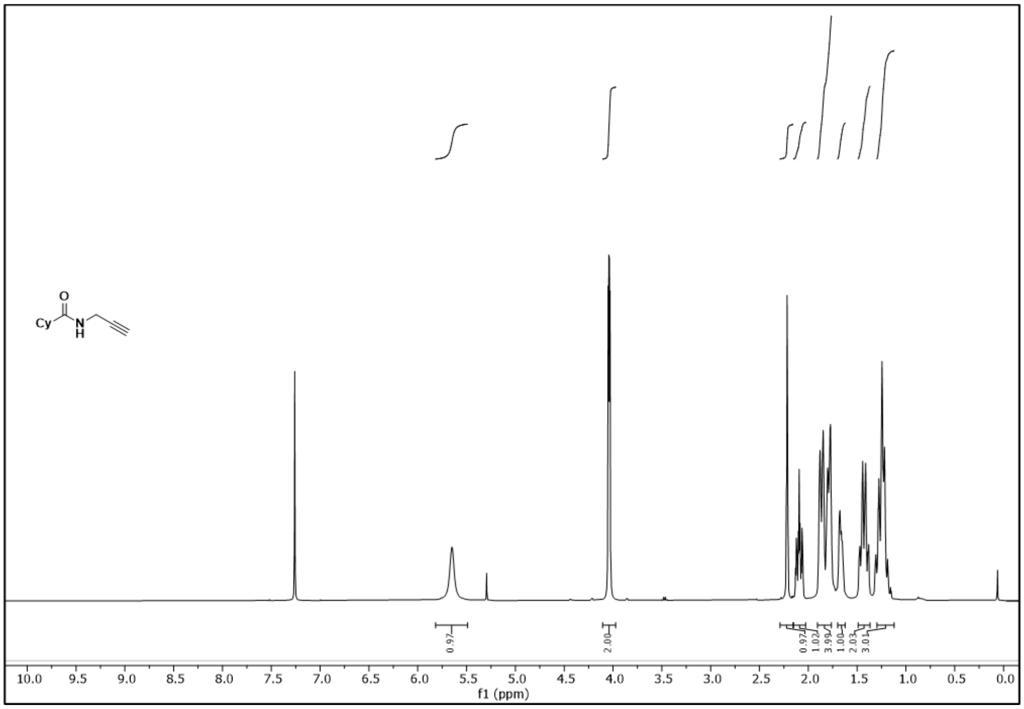

*^{1}H NMR Spectrum of **Al-12***

400 MHz, $CDCl_3$

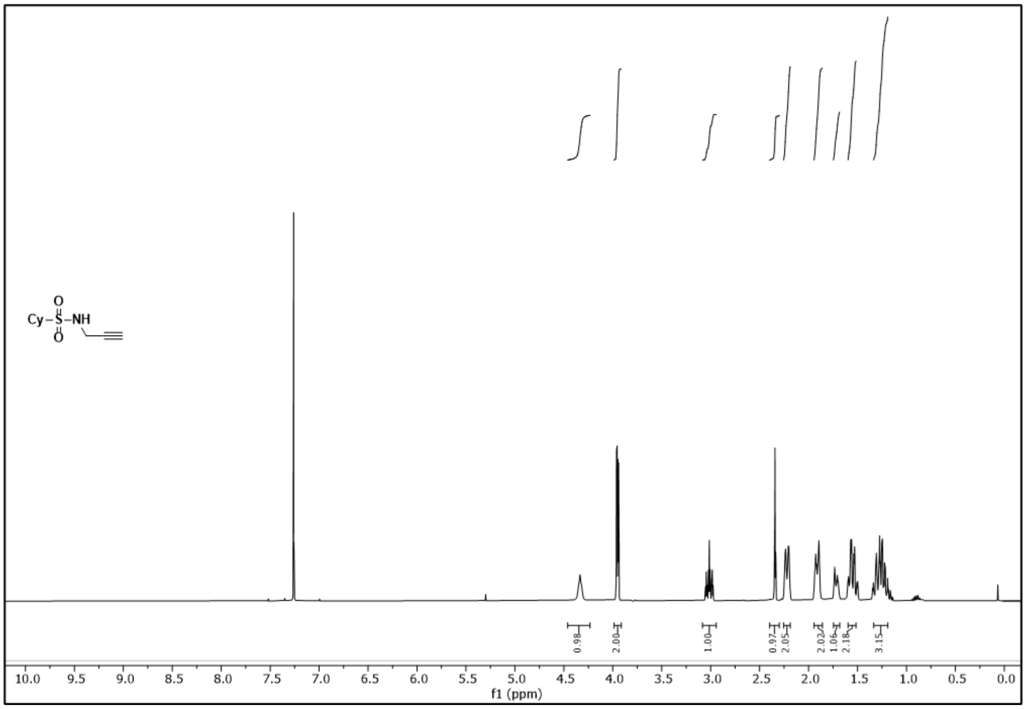

*^{1}H NMR Spectrum of **Al-13***

600 MHz, $CDCl_3$

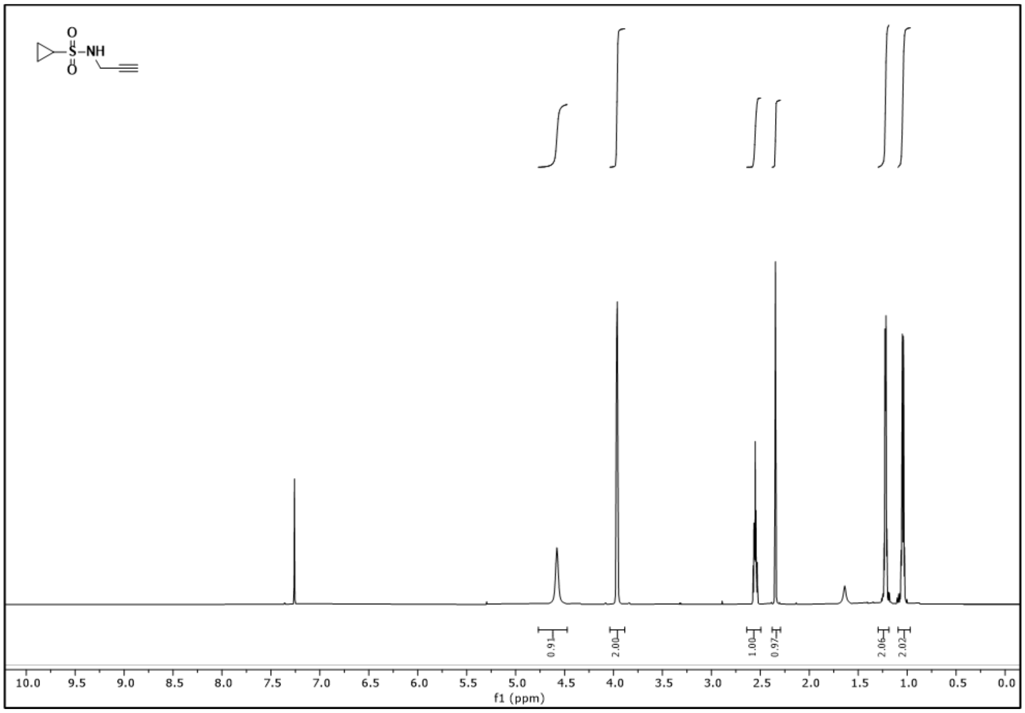

*^{1}H NMR Spectrum of **Al-14***

600 MHz, $CDCl_3$

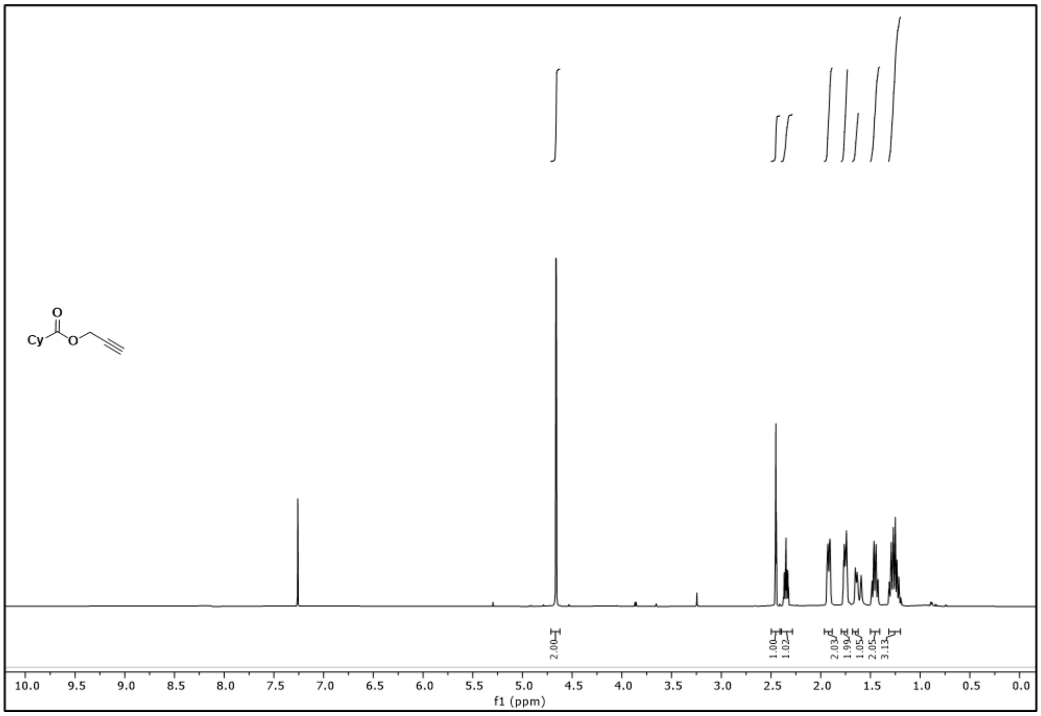

*^{1}H NMR Spectrum of **Al-19***

400 MHz, $CDCl_3$

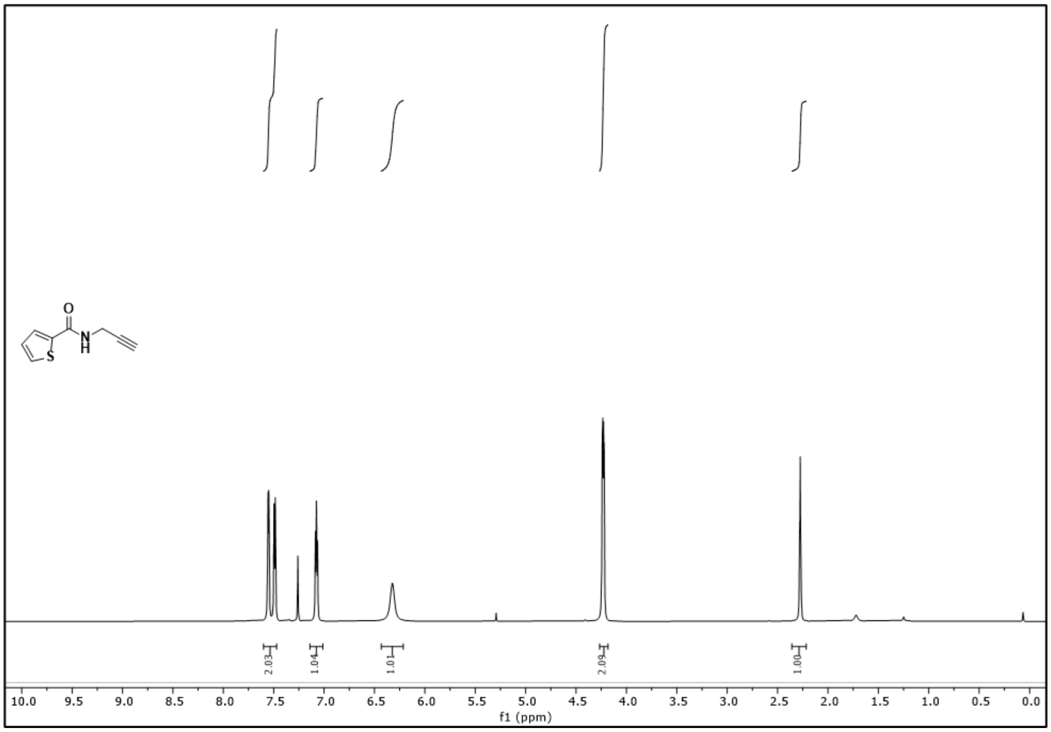

*¹H NMR Spectrum of **Al-20***

400 MHz, $CDCl_3$

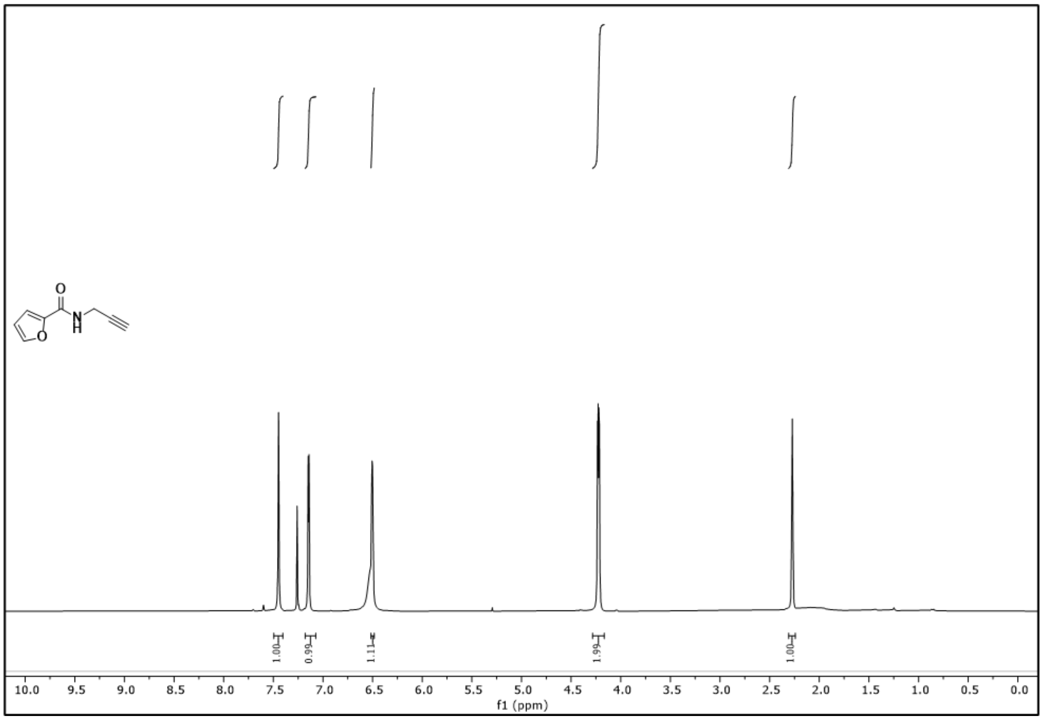

*¹H NMR Spectrum of **Al-21***

400 MHz, $CDCl_3$

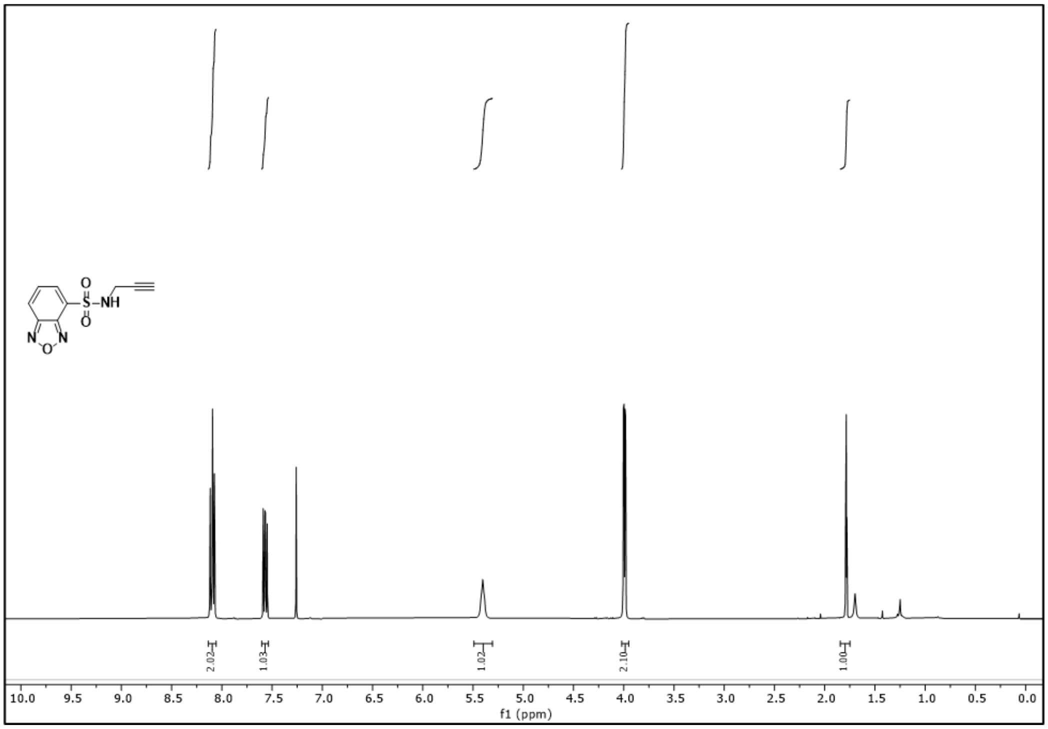

^{1}H NMR Spectrum of ***Al-22***

400 MHz, $CDCl_3$

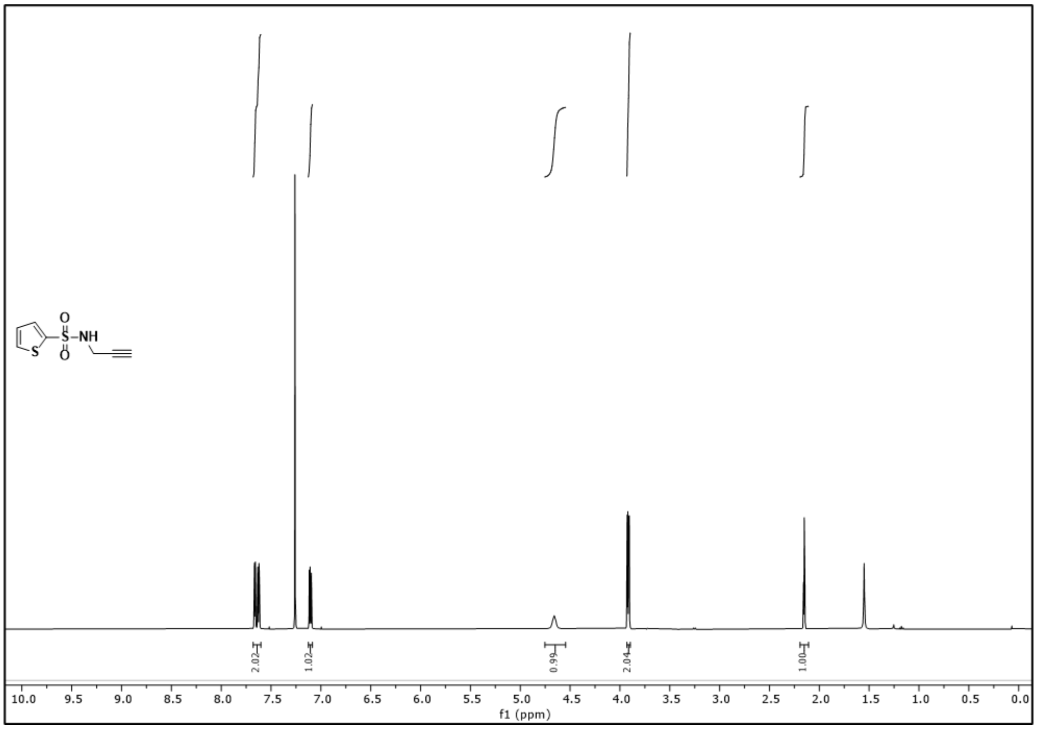

^{1}H NMR Spectrum of ***Al-23***

400 MHz, $CDCl_3$

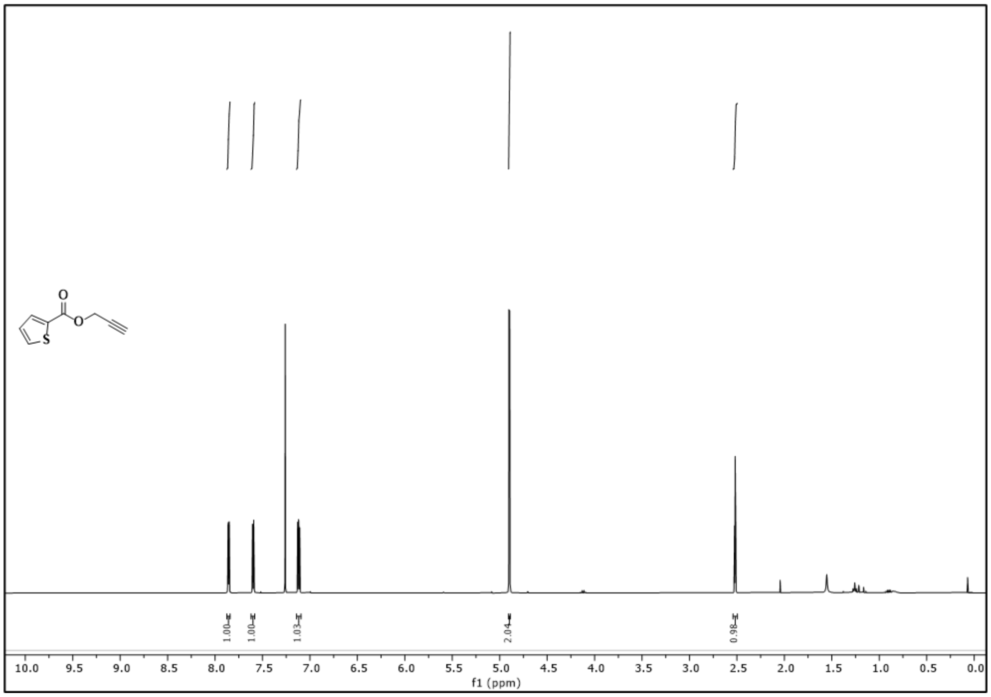

*^{1}H NMR Spectrum of **Al-28***

400 MHz, MeOD

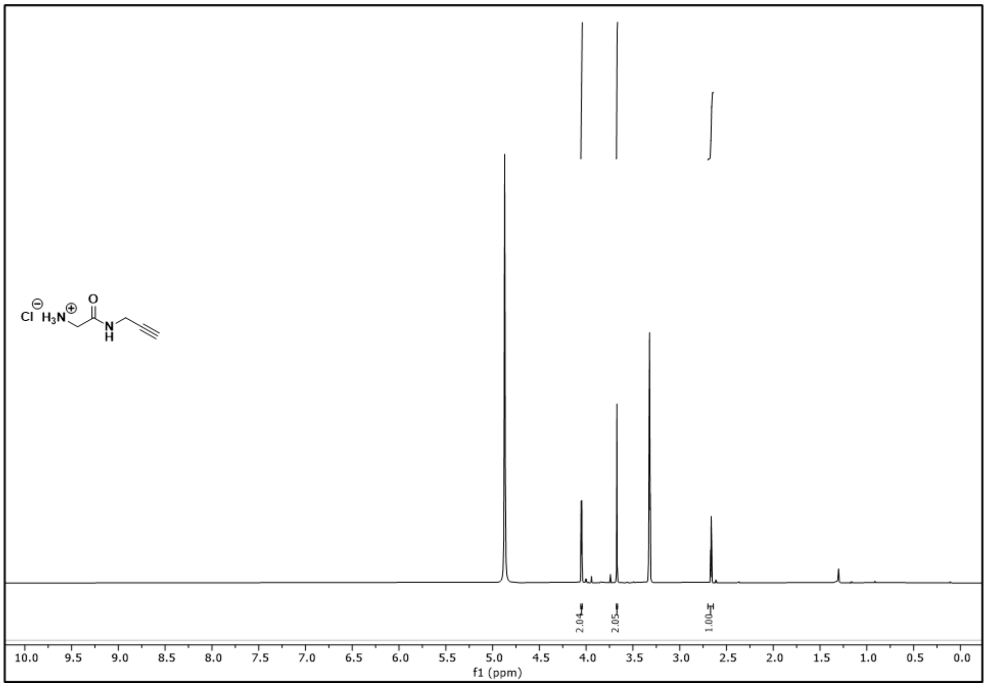

*^{1}H NMR Spectrum of **Al-30***

400 MHz, $CDCl_3$

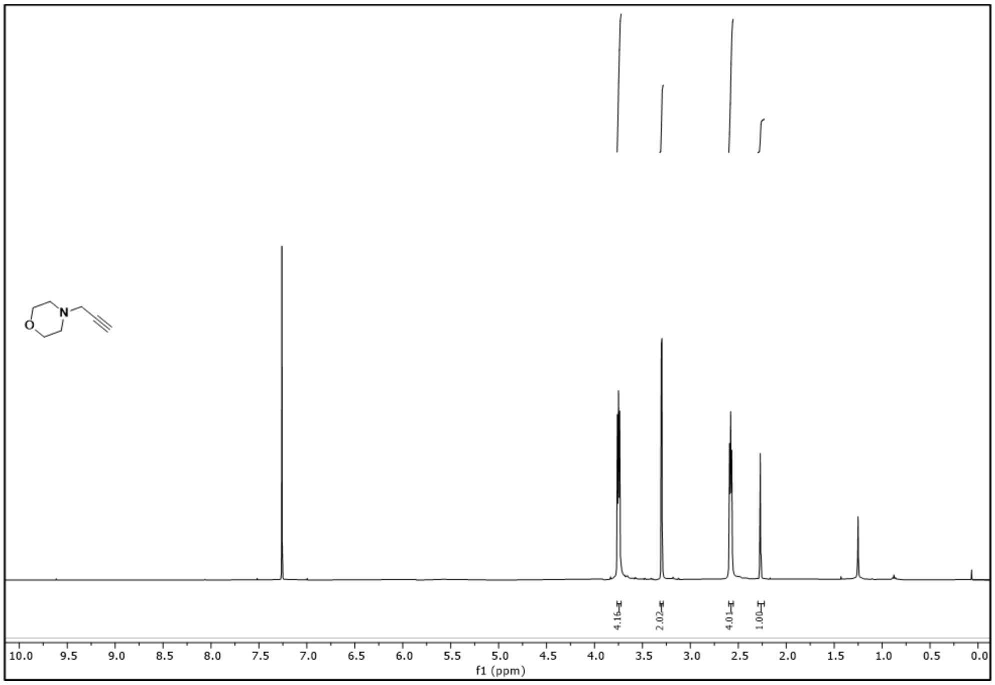

*¹H NMR Spectrum of **Al-32***

400 MHz, $CDCl_3$

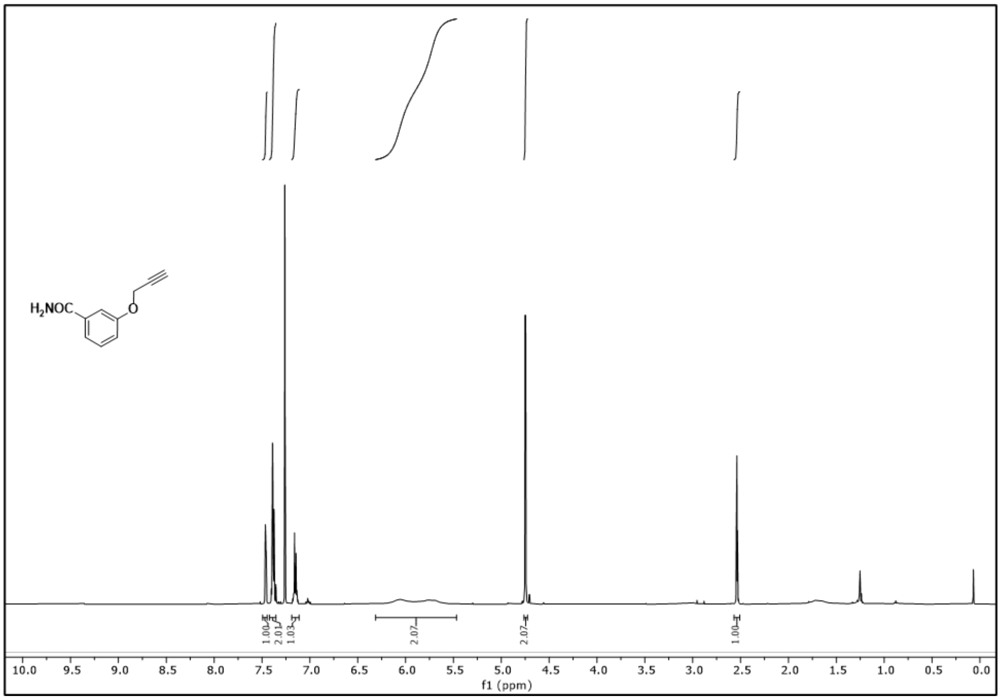

*¹H NMR Spectrum of **Al-37***

400 MHz, MeOD

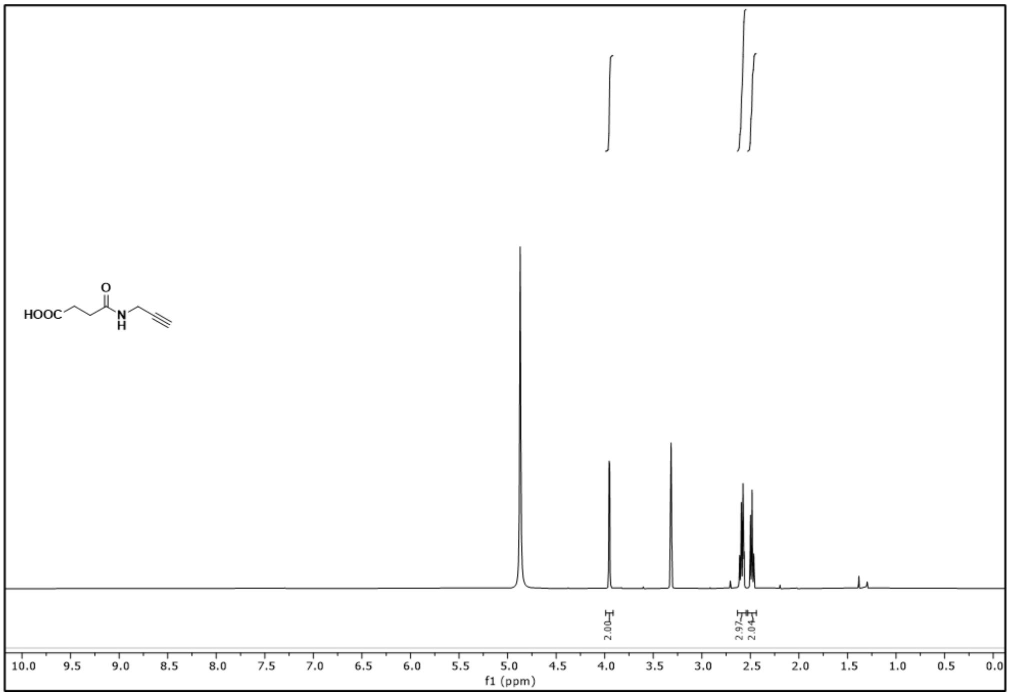

^{1}H NMR Spectrum of ***Al-38***

400 MHz, $CDCl_3$

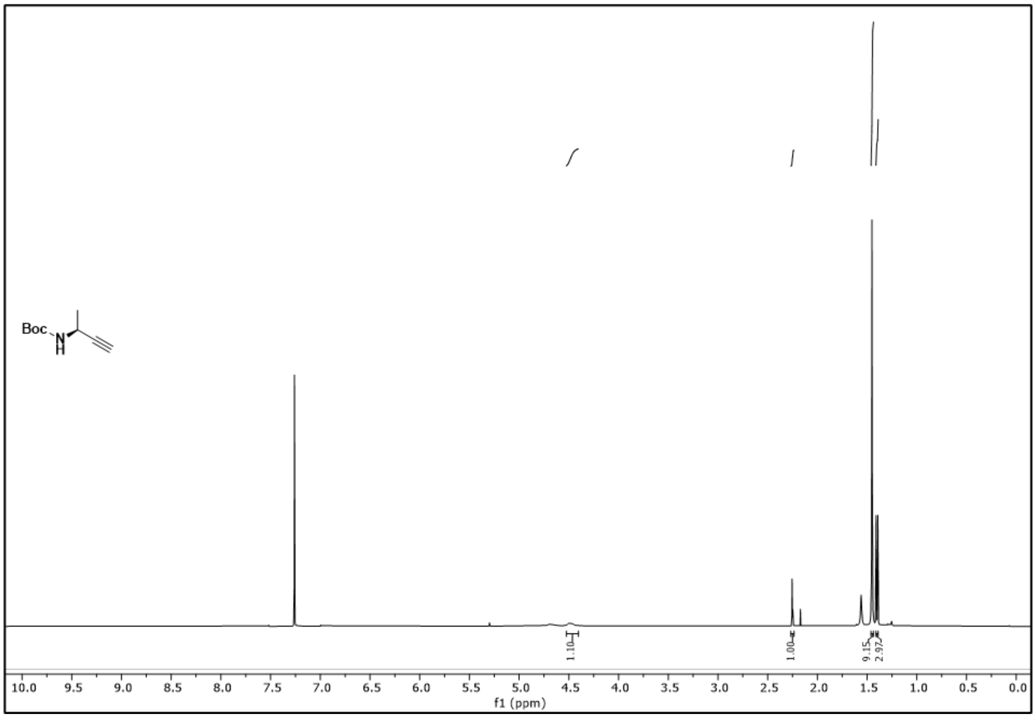

^{1}H NMR Spectrum of ***Al-39***

400 MHz, $CDCl_3$

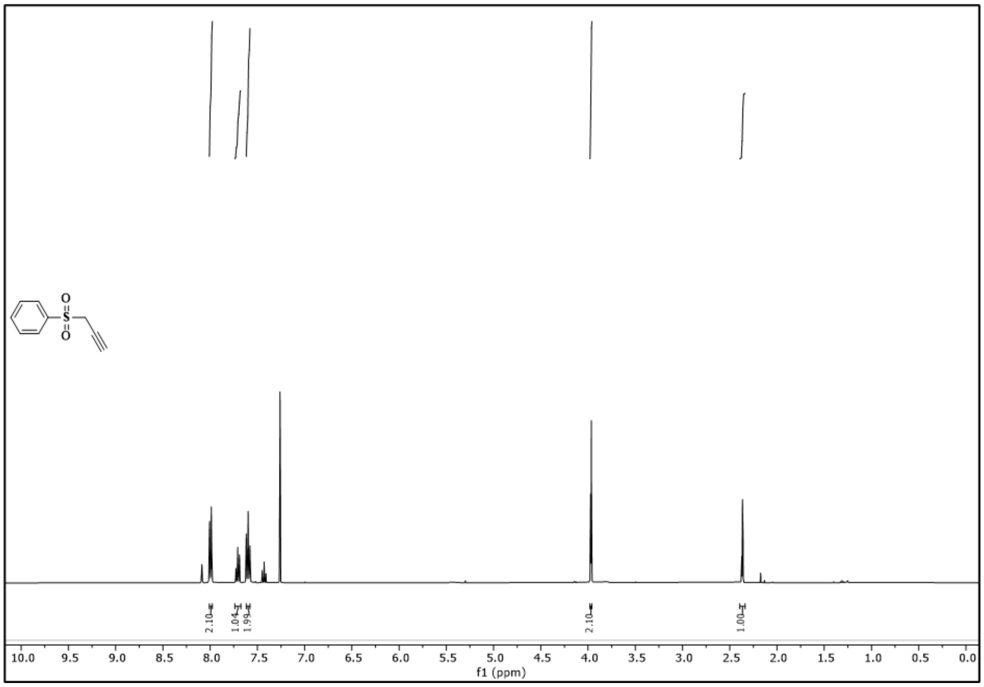

^{1}H NMR Spectrum of ***Al-40***

400 MHz, $CDCl_3$

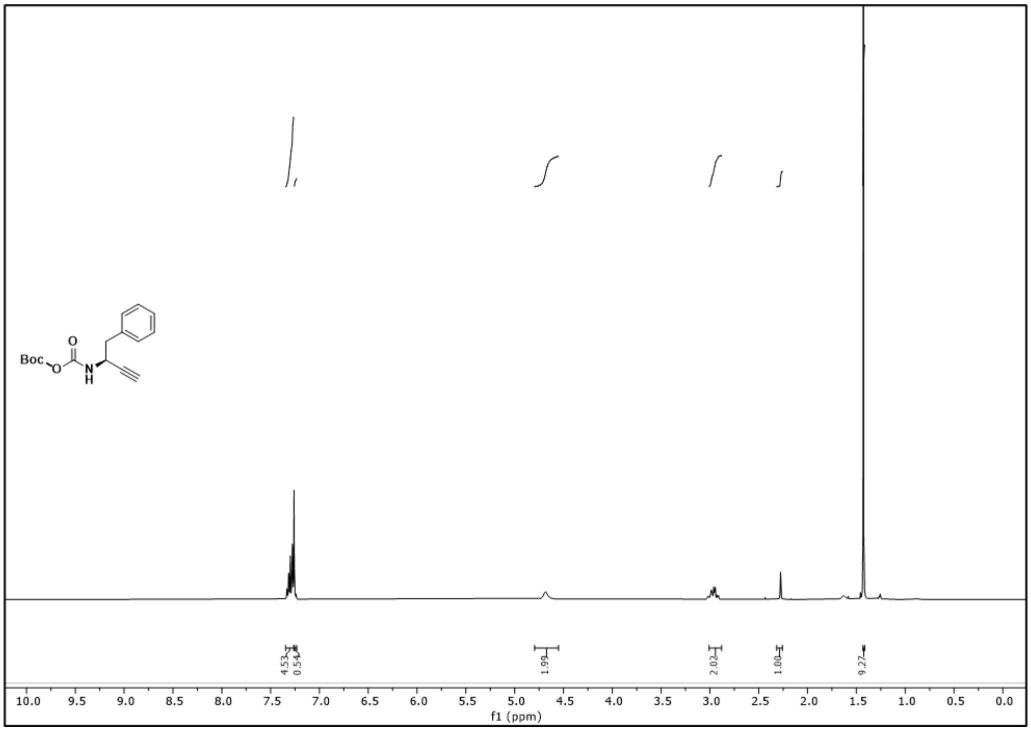

^{1}H NMR Spectrum of ***Al-41***

400 MHz, $CDCl_3$

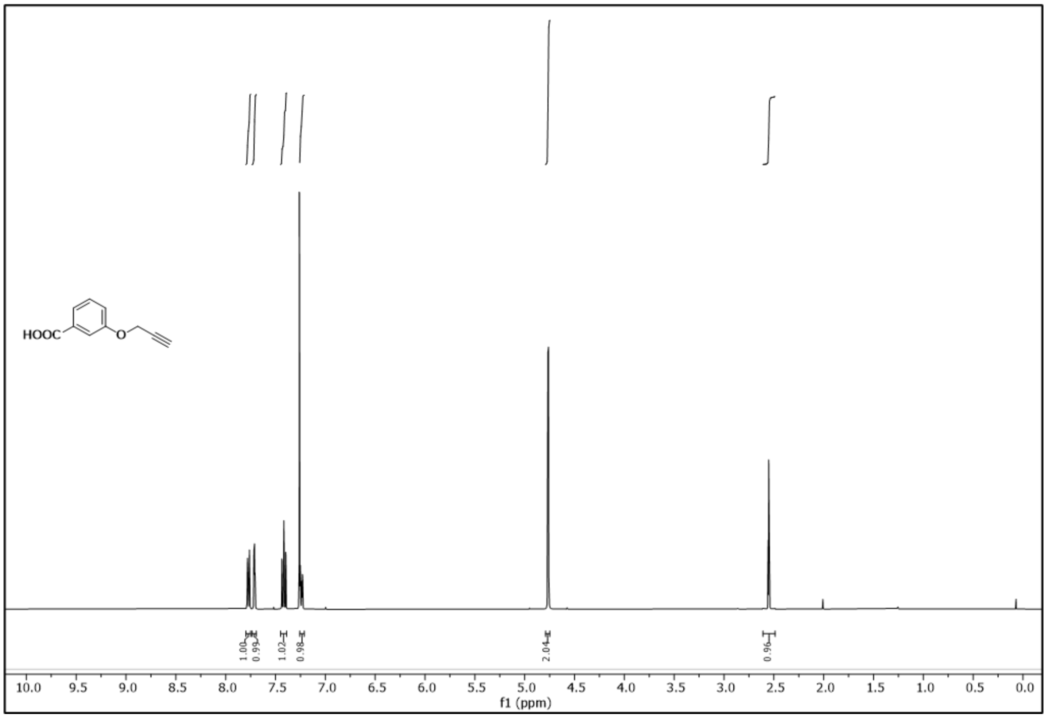

^{1}H NMR Spectrum of ***Al-42***

600 MHz, $CDCl_3$

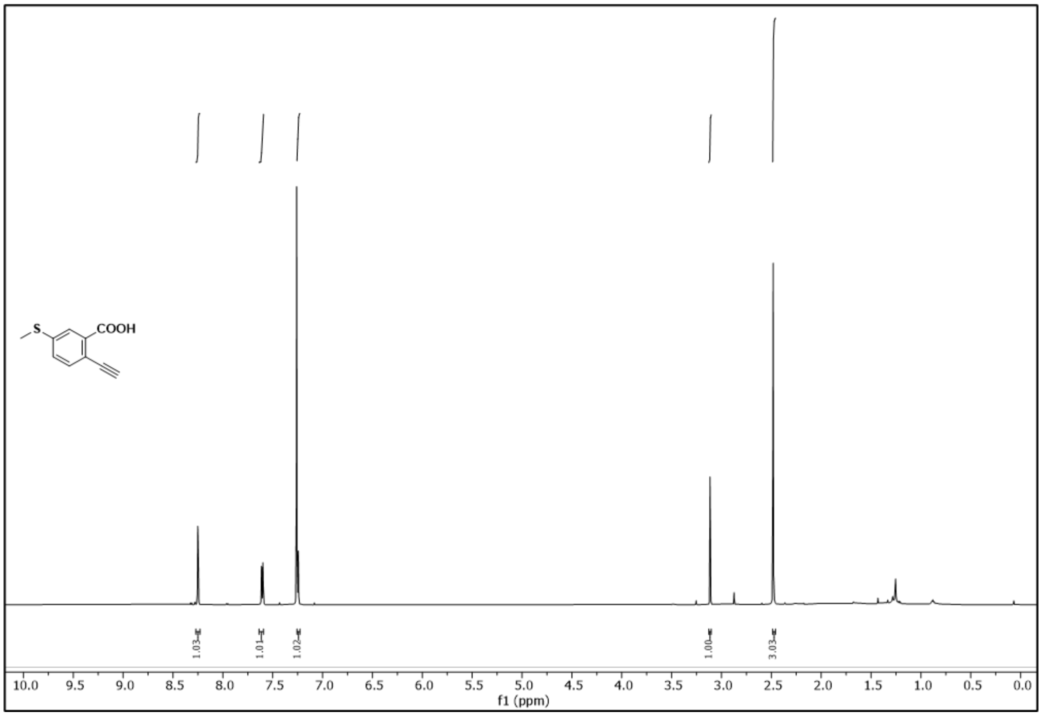

^{1}H NMR Spectrum of ***Al-45***

600 MHz, MeOD

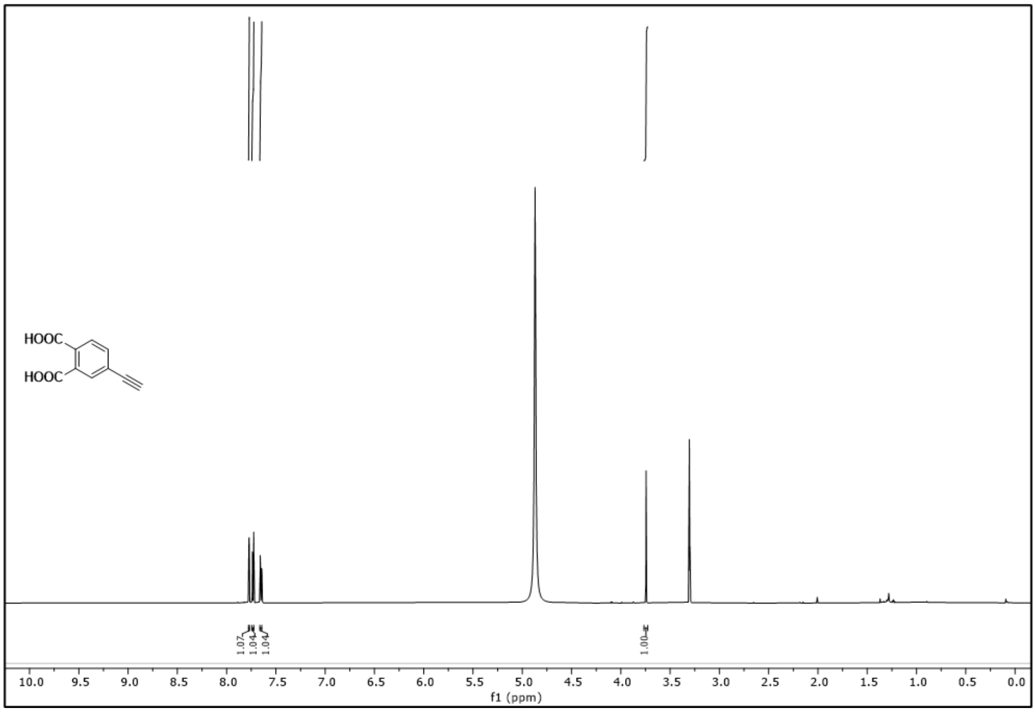

^{1}H NMR Spectrum of ***Al-46***

600 MHz, $CDCl_3$

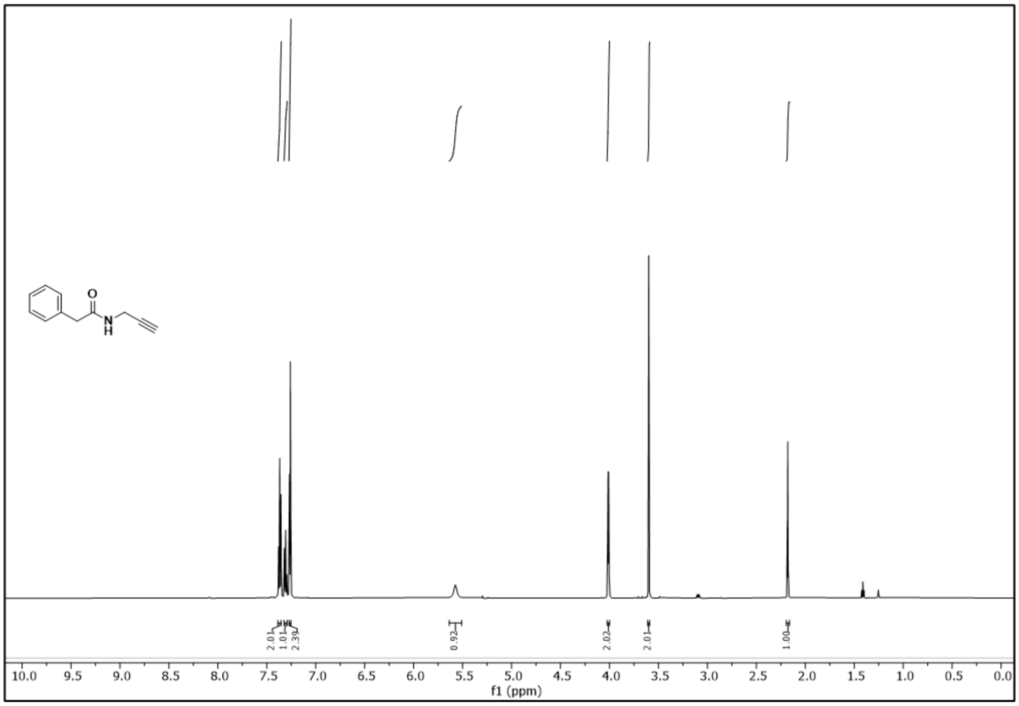

^{1}H NMR Spectrum of ***Al-47***

600 MHz, $CDCl_3$

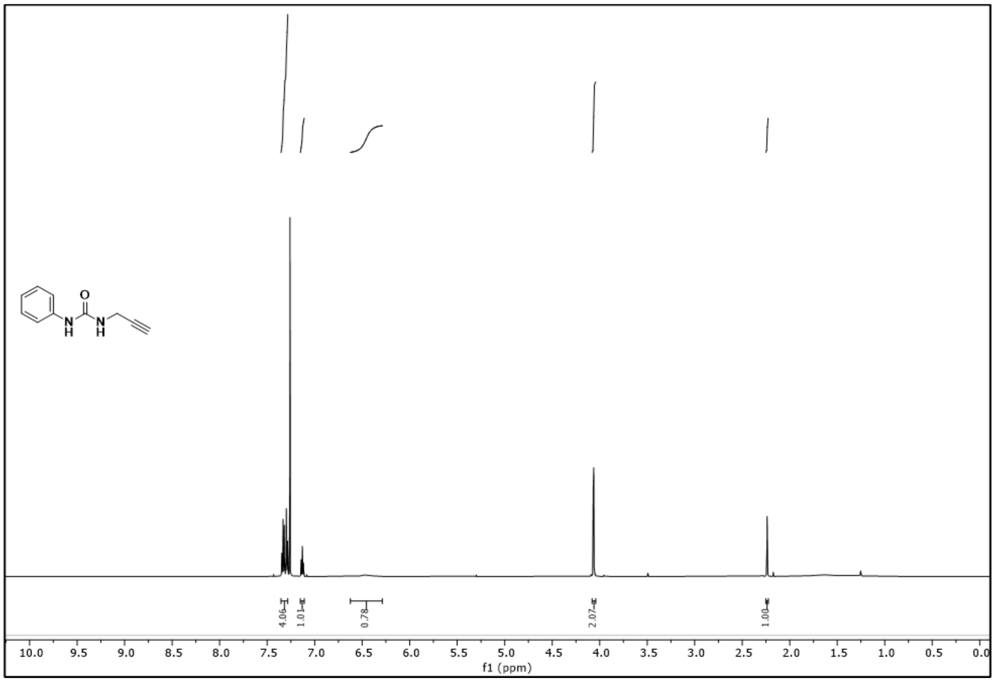

^{1}H NMR Spectrum of ***Al-49***

600 MHz, $CDCl_3$

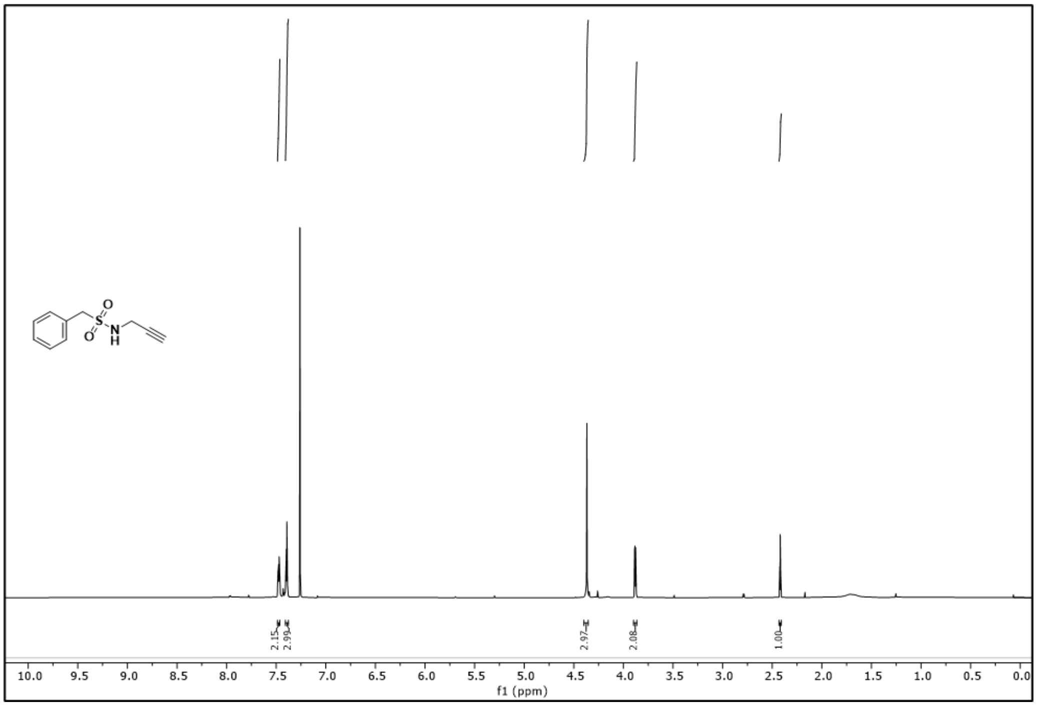

^{1}H NMR Spectrum of ***Al-55***

600 MHz, $CDCl_3$

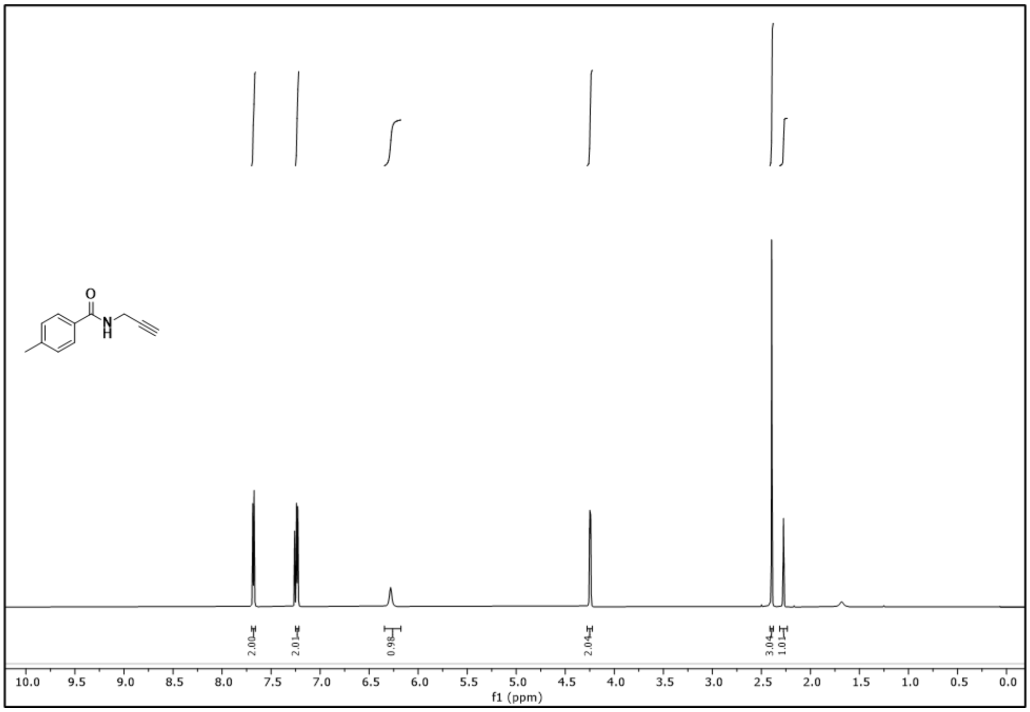

^{1}H NMR Spectrum of ***Al-56***

400 MHz, $CDCl_3$

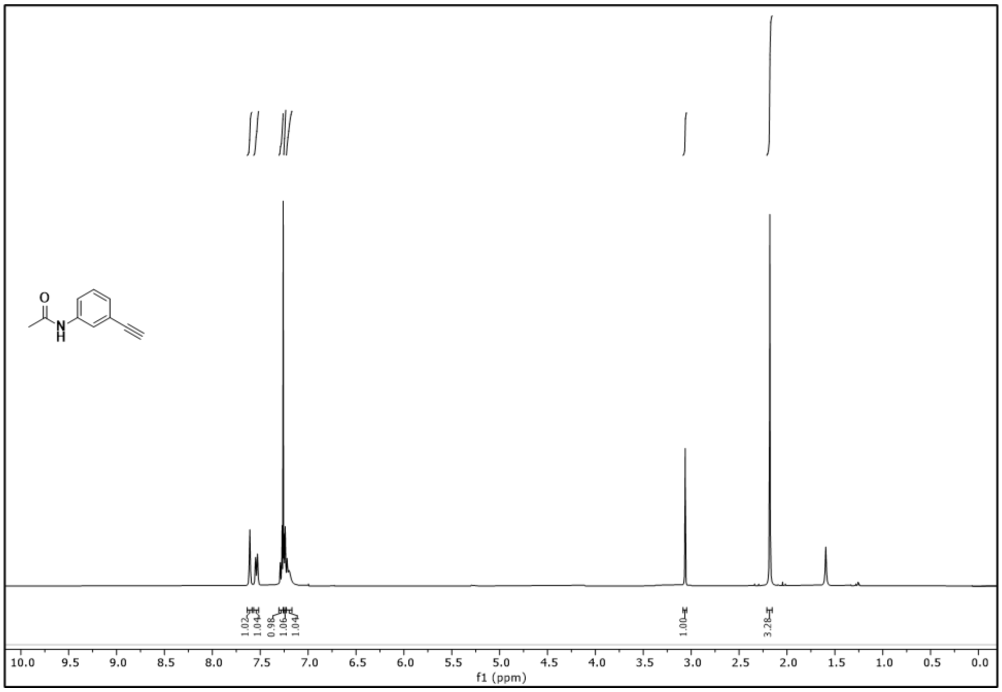

^{1}H NMR Spectrum of ***Al-57***

600 MHz, $CDCl_3$

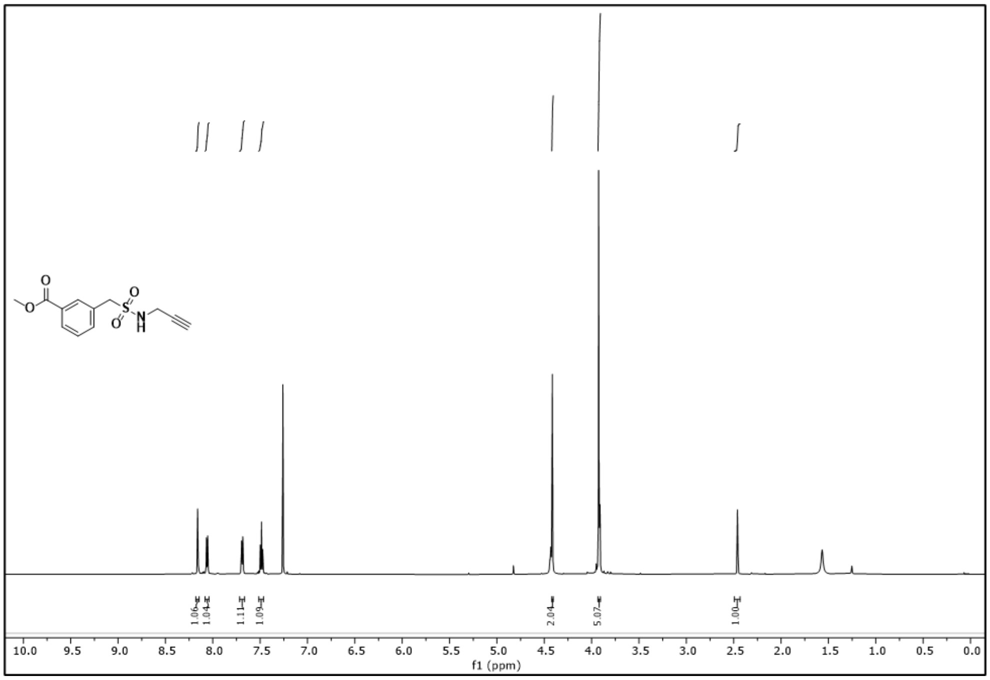

¹H NMR Spectrum of ***Al-58***

600 MHz, $CDCl_3$

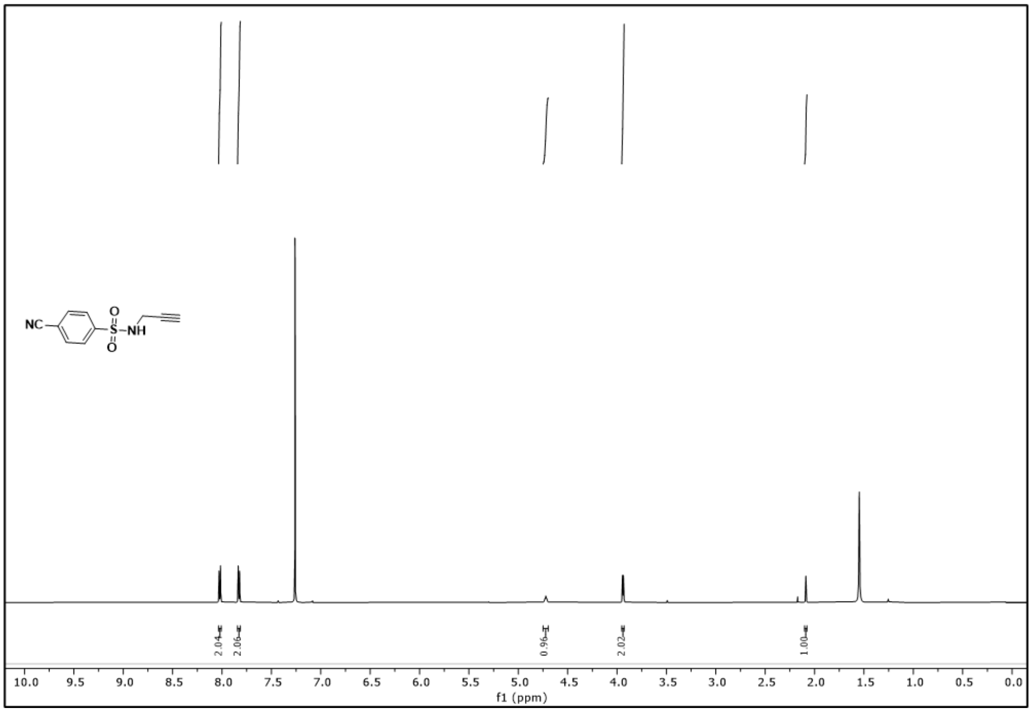

¹H NMR Spectrum of ***Al-59***

600 MHz, $CDCl_3$

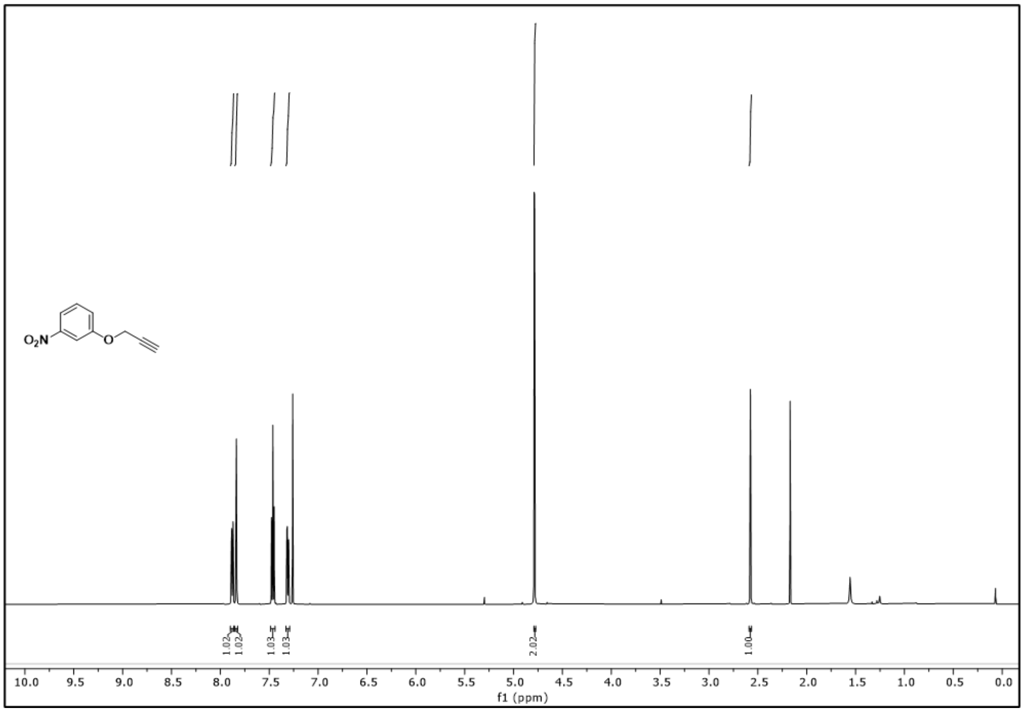

^{1}H NMR Spectrum of ***Al-60***

600 MHz, $CDCl_3$

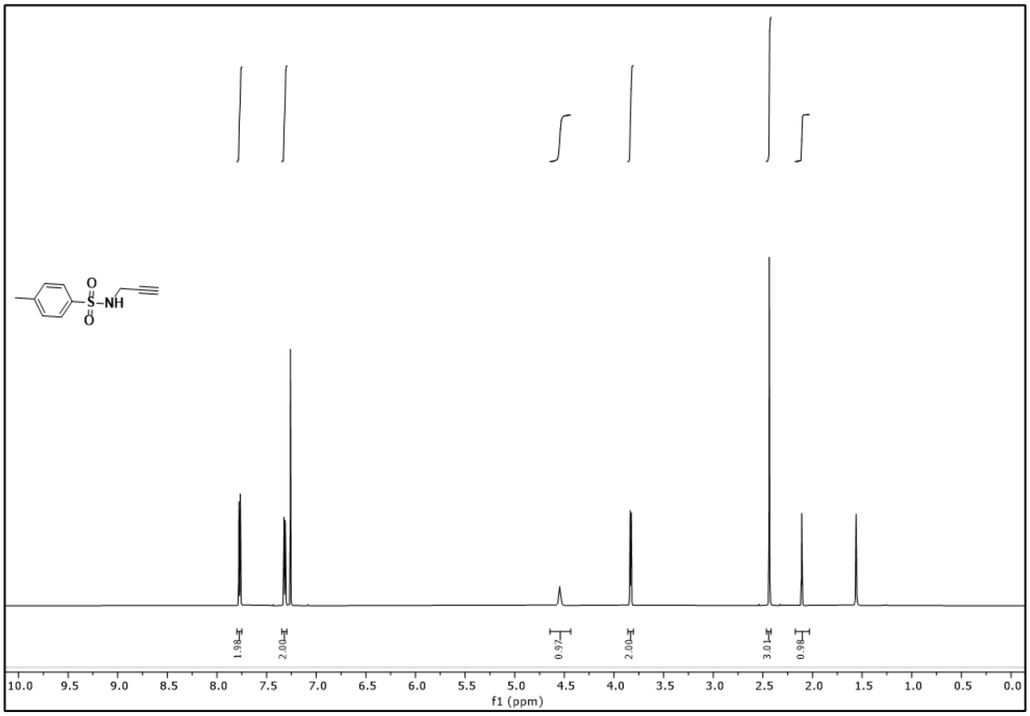

^{1}H NMR Spectrum of ***Al-64***

400 MHz, $CDCl_3$

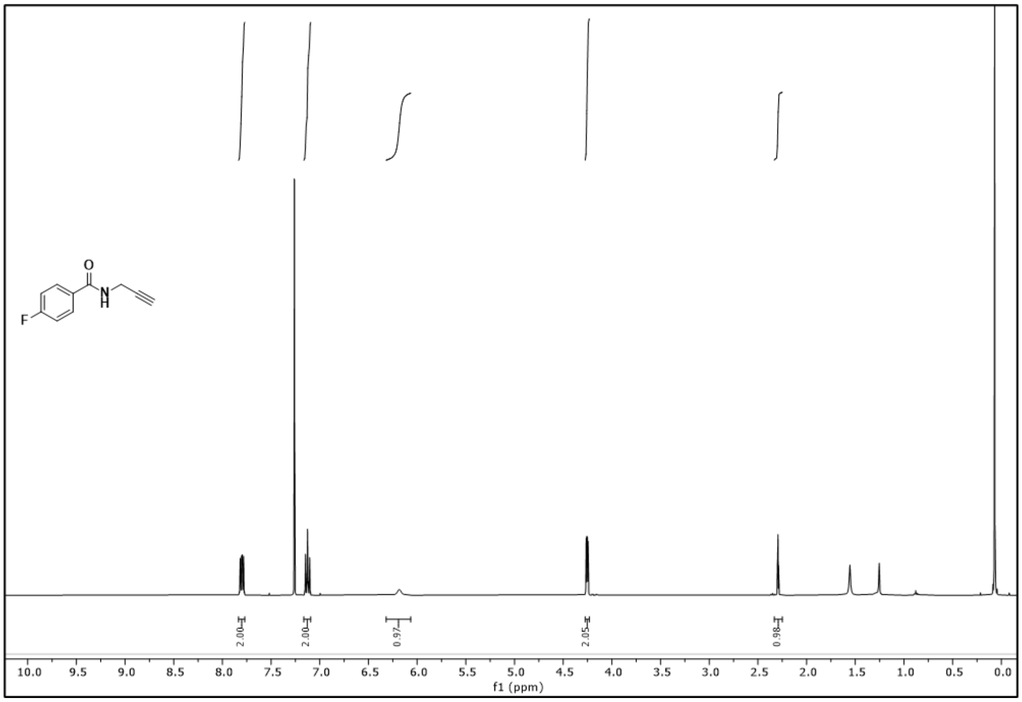

^{1}H NMR Spectrum of ***Al-65***

400 MHz, $CDCl_3$

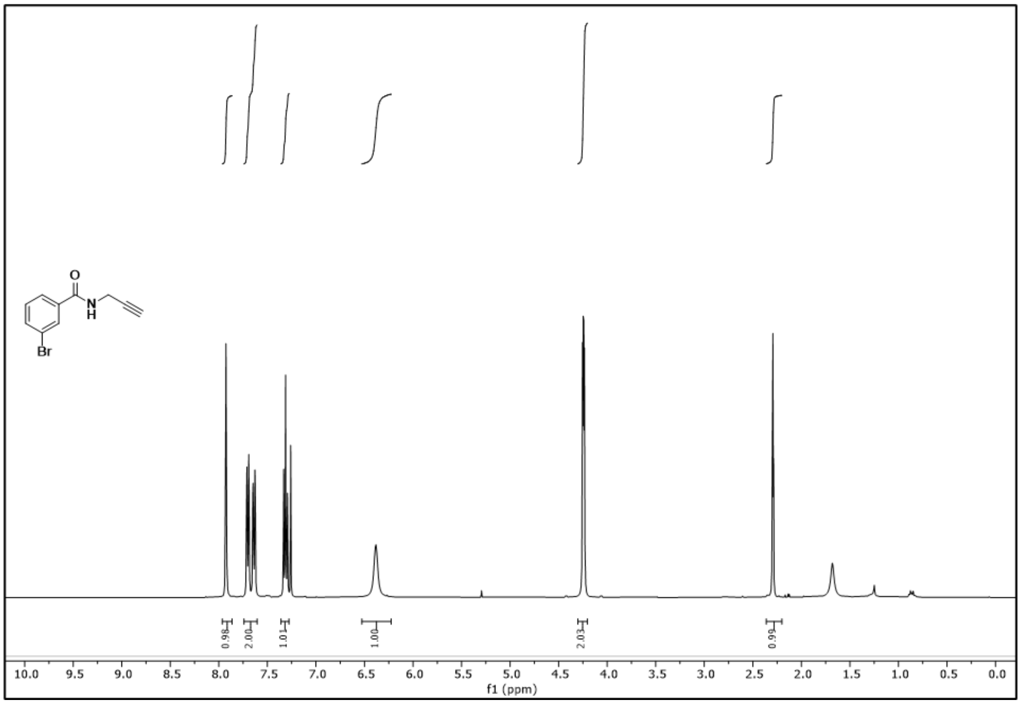

^{1}H NMR Spectrum of ***Al-66***

600 MHz, $CDCl_3$

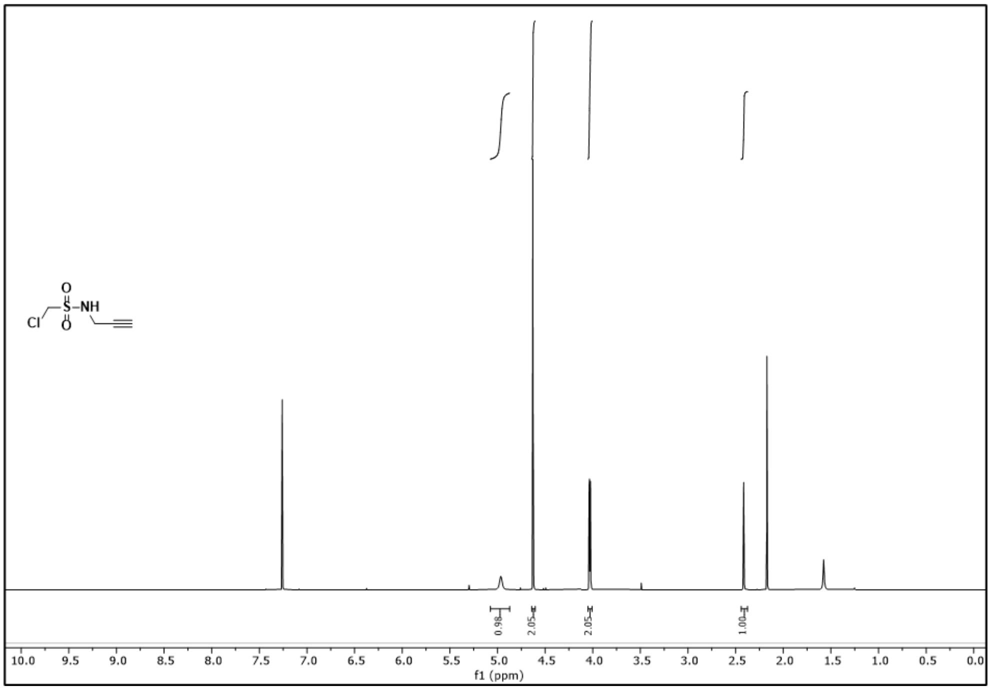

*¹H NMR Spectrum of **Al-67***

400 MHz, $CDCl_3$

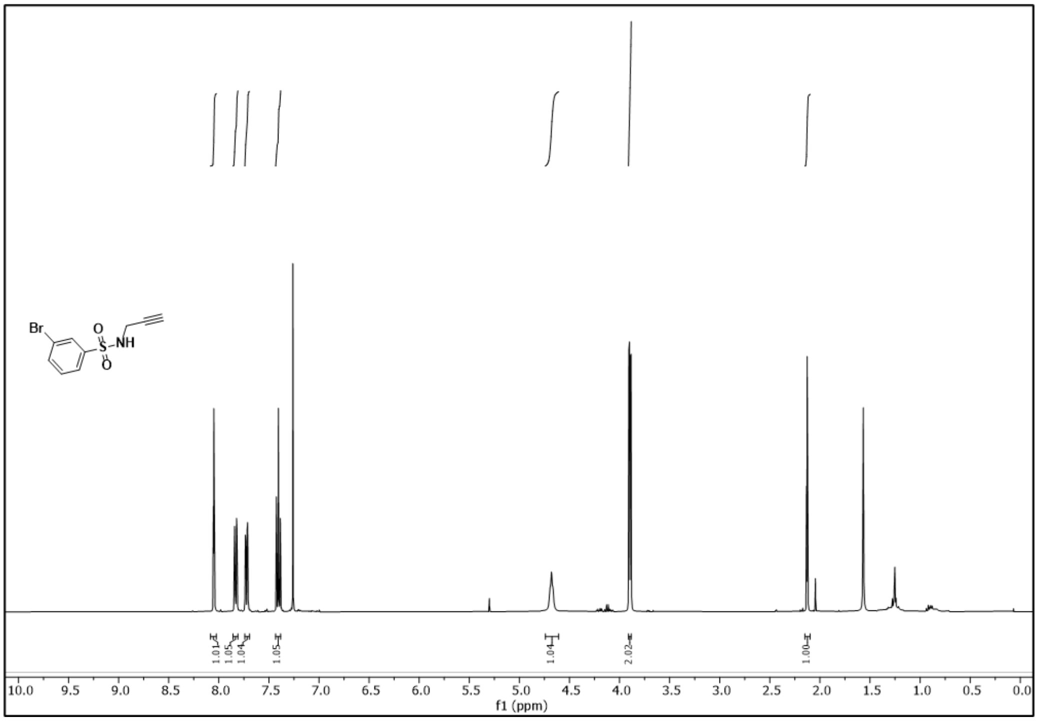

*¹H NMR Spectrum of **Al-68***

400 MHz, $CDCl_3$

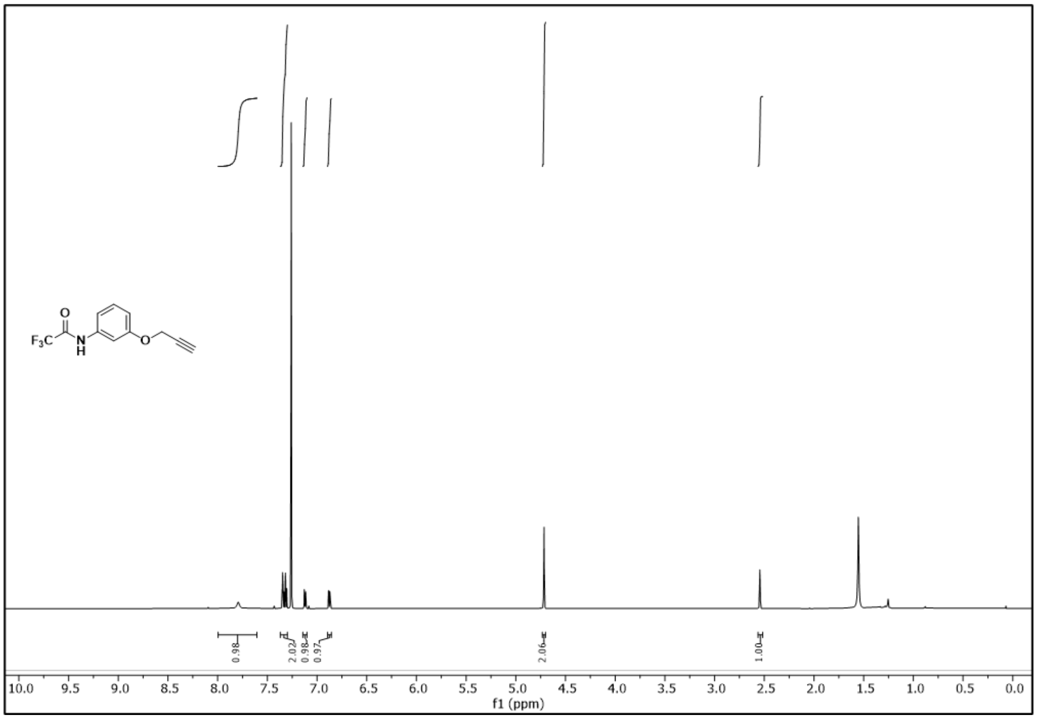

*¹H NMR Spectrum of **Al-73***

400 MHz, $CDCl_3$

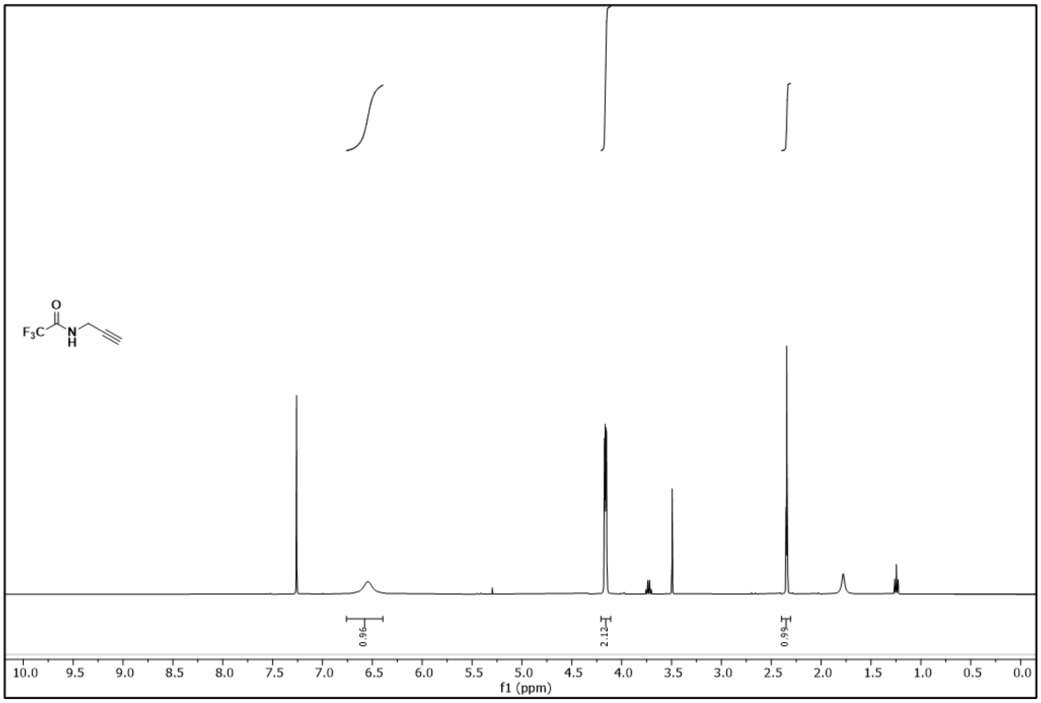

*¹H NMR Spectrum of **Al-74***

400 MHz, $CDCl_3$

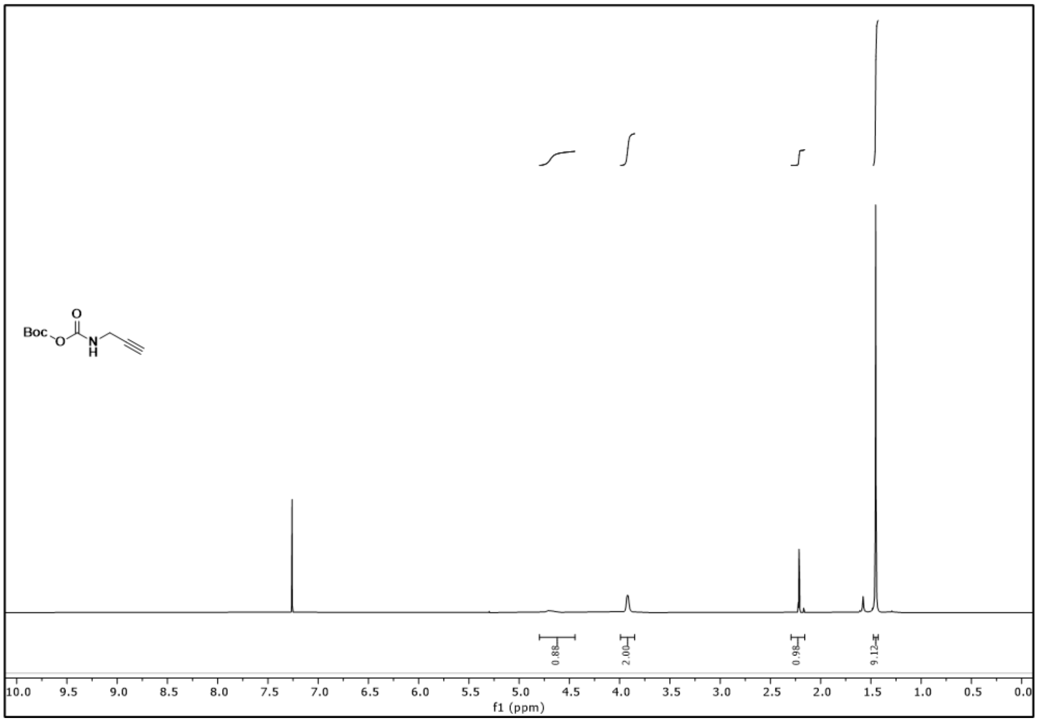

^{1}H NMR Spectrum of ***Al-76***

600 MHz, $CDCl_3$

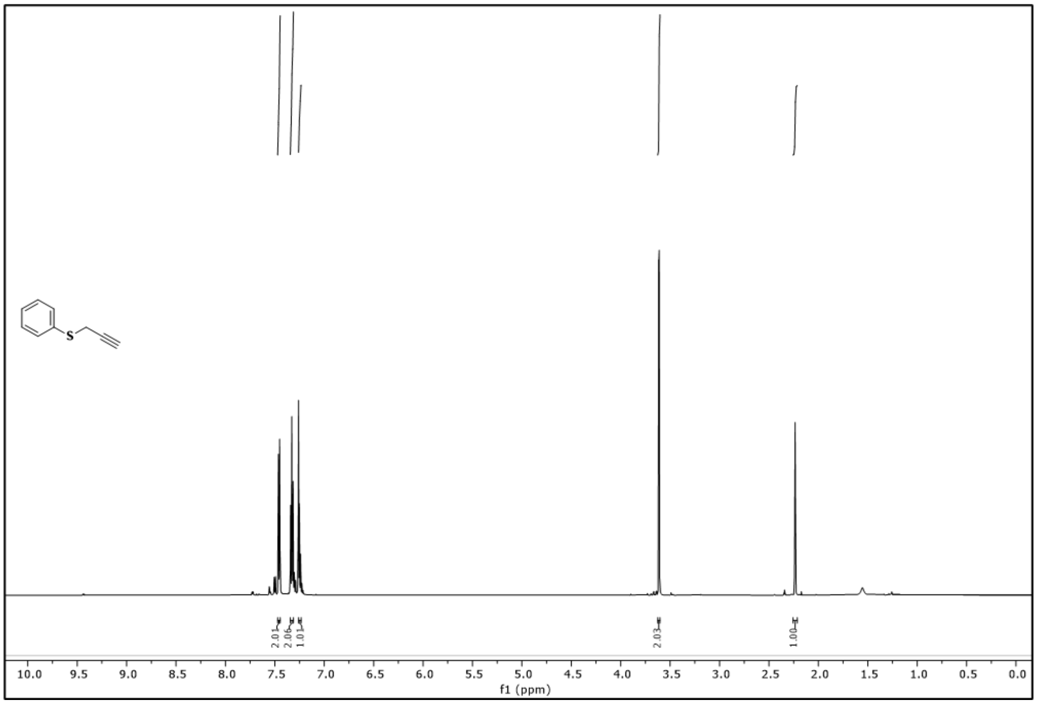

^{1}H NMR Spectrum of ***Al-77***

600 MHz, $CDCl_3$

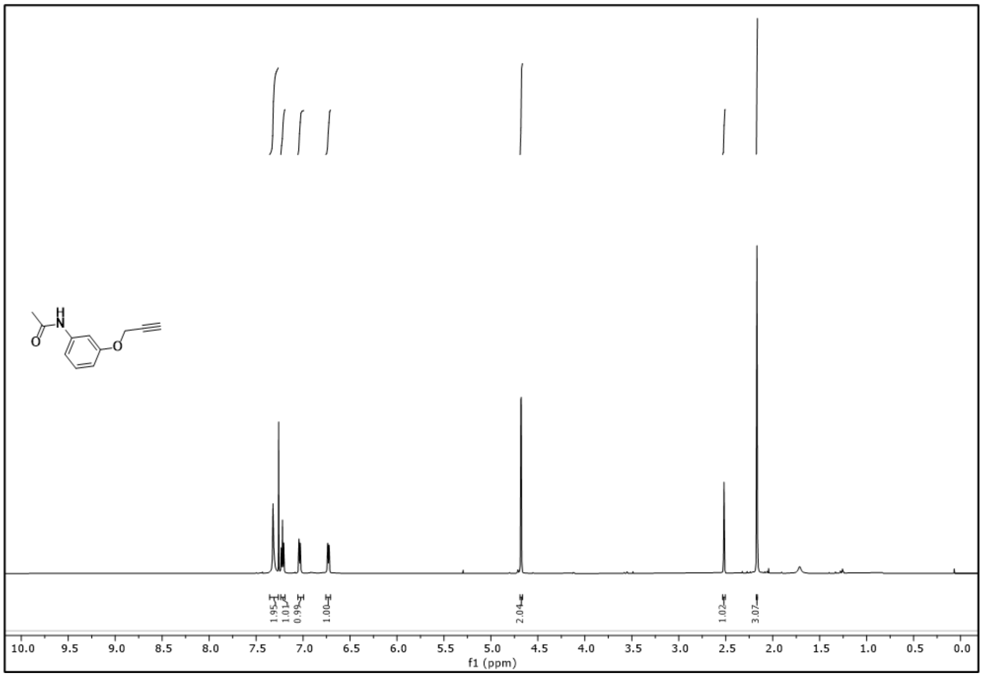

¹H NMR Spectrum of ***Al-81***

400 MHz, $CDCl_3$

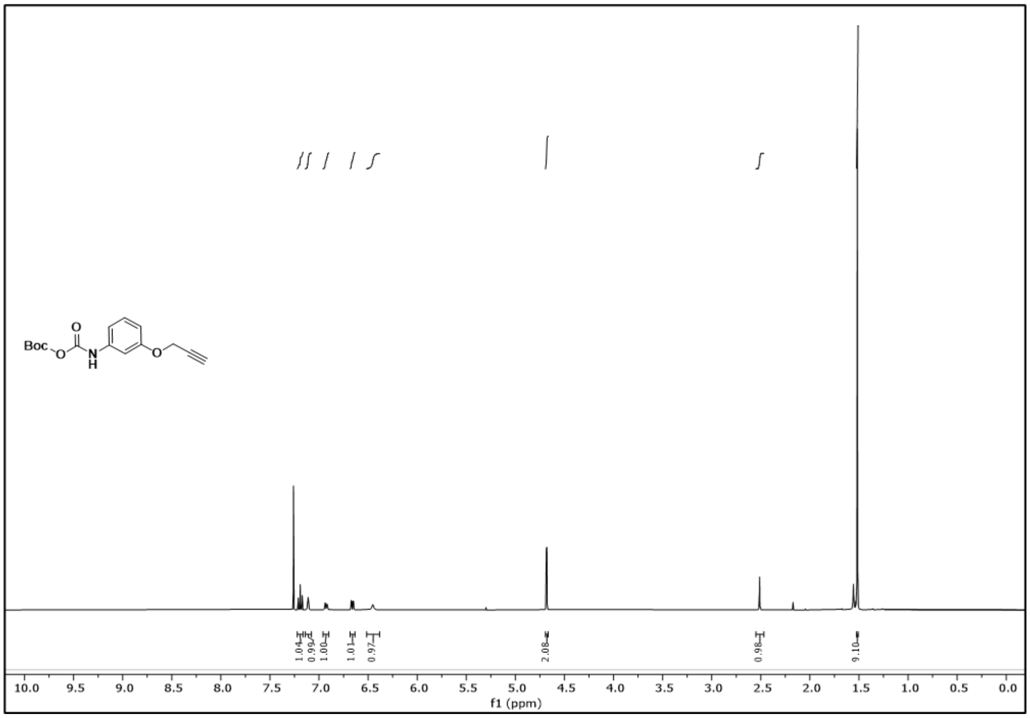

¹H NMR Spectrum of ***Al-82***

600 MHz, $CDCl_3$

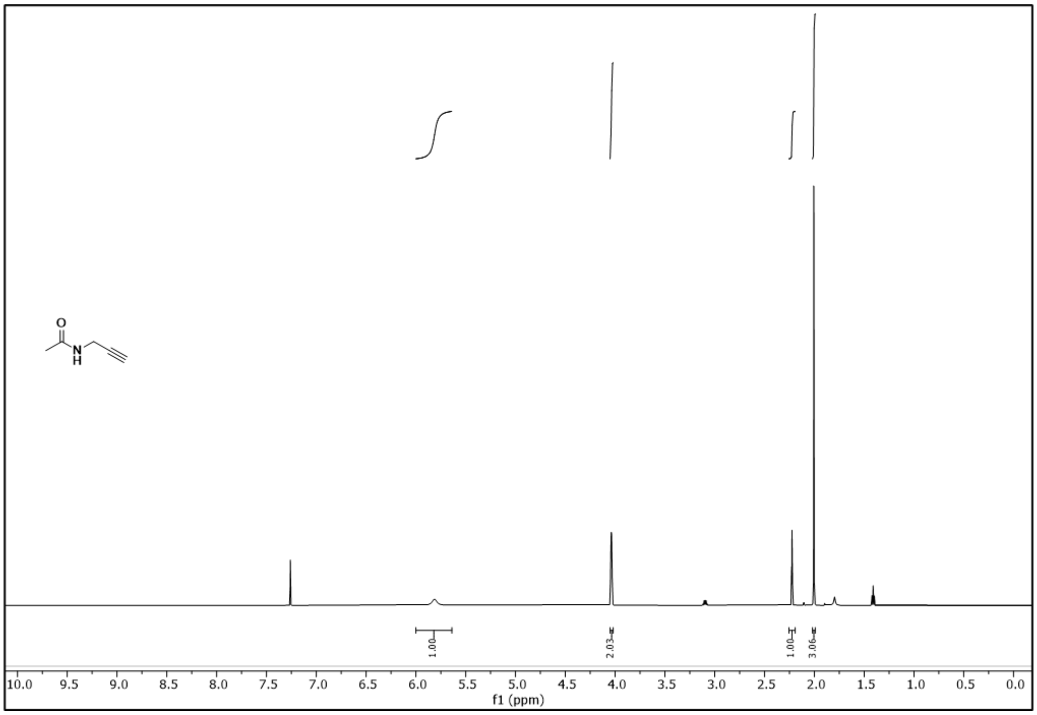

^{1}H NMR Spectrum of ***Al-83***

600 MHz, $CDCl_3$

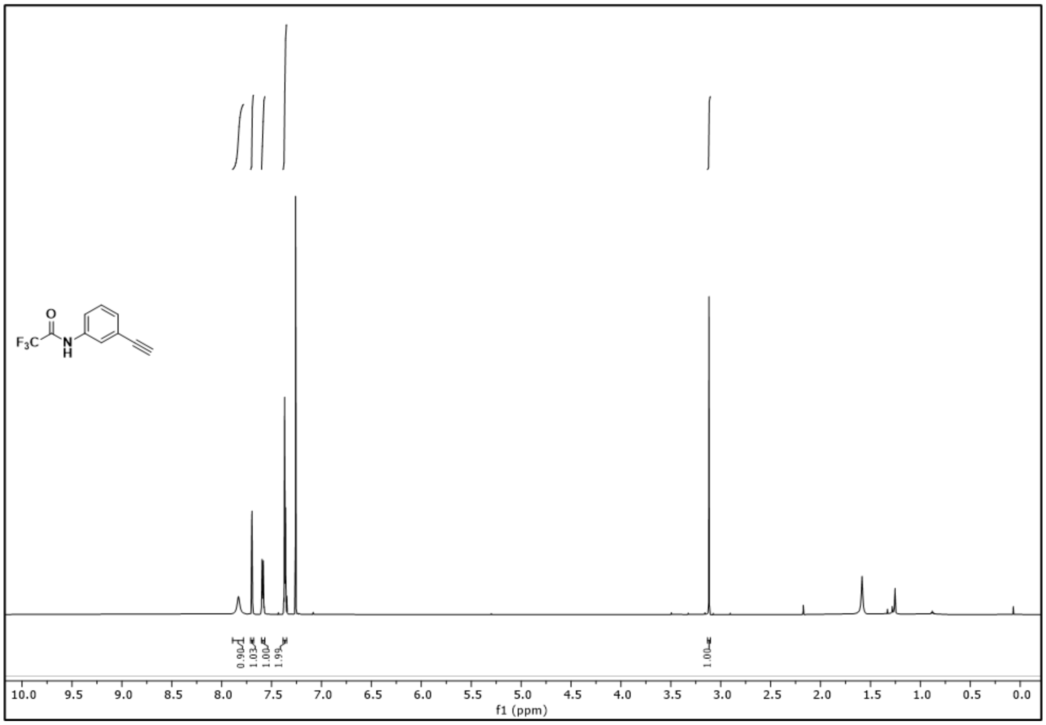

^{1}H NMR Spectrum of ***Al-84***

400 MHz, $CDCl_3$

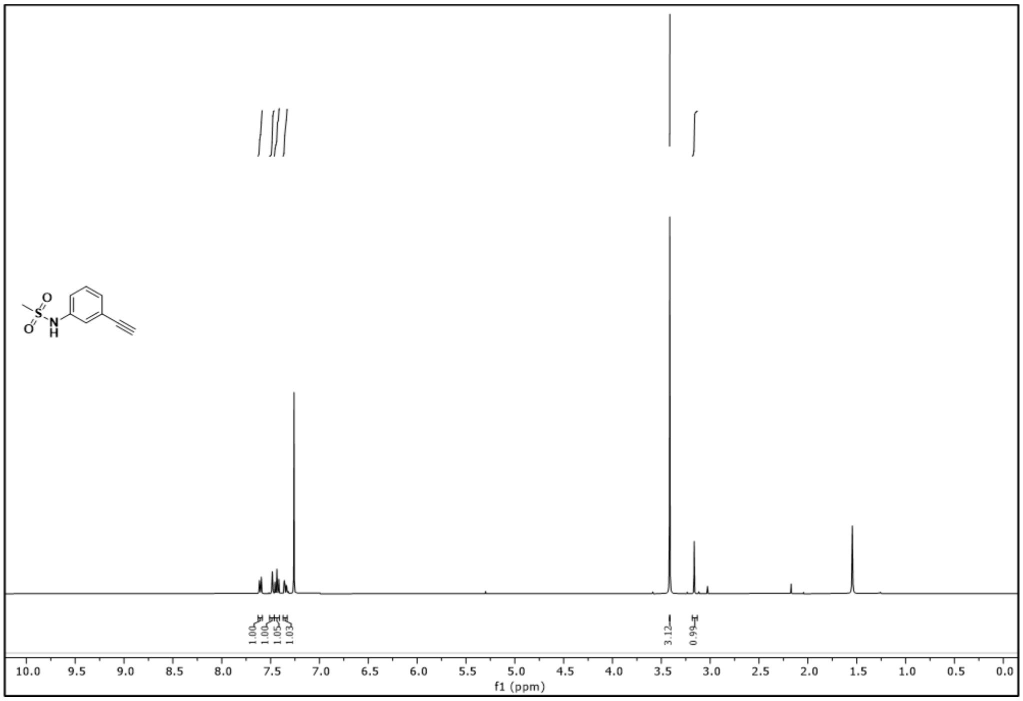

[1]*H NMR Spectrum of* ***Al-85***

600 MHz, $CDCl_3$

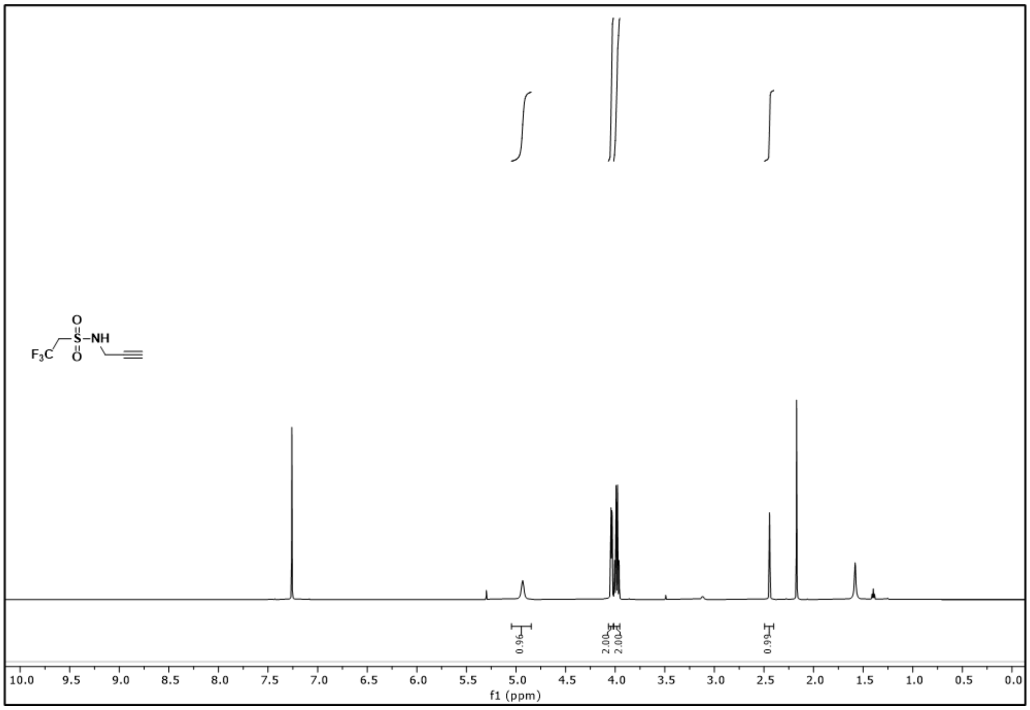

[1]*H NMR Spectrum of* ***Al-88***

400 MHz, $CDCl_3$

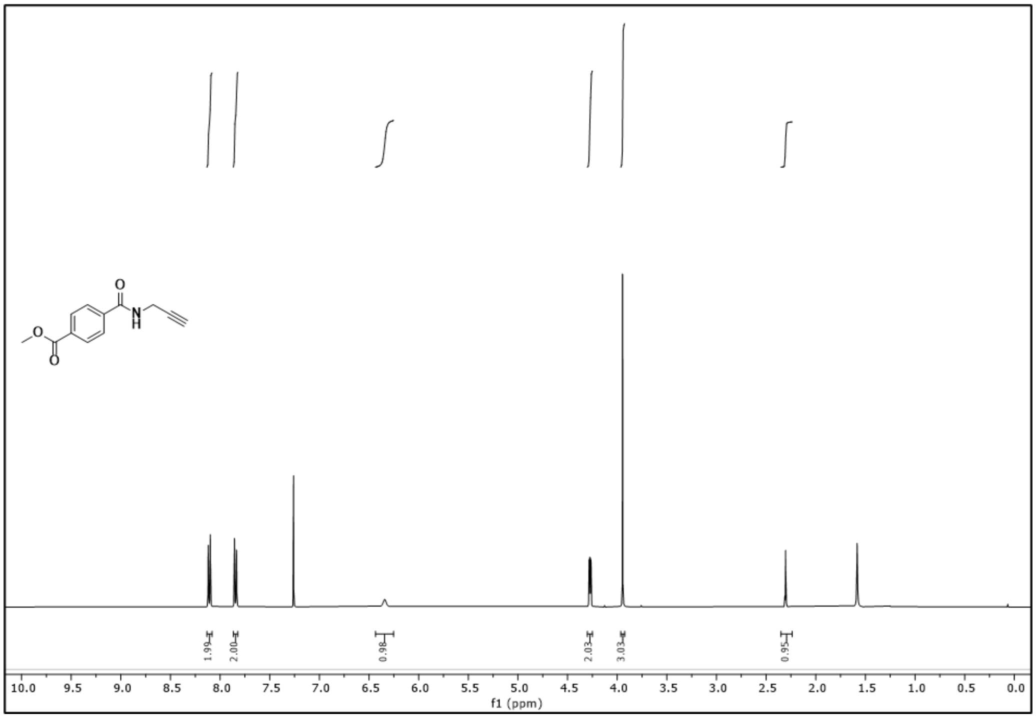

NMR Spectra for Triazoles and starting materials

^{1}H NMR Spectrum of ***15***

400 MHz, $CDCl_3$

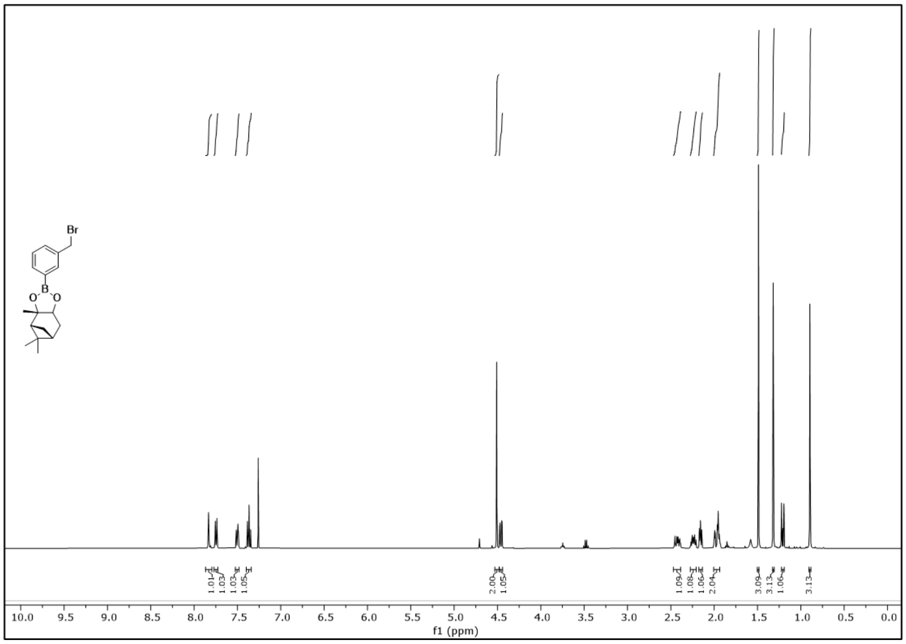

^{1}H NMR Spectrum of ***16***

400 MHz, $CDCl_3$

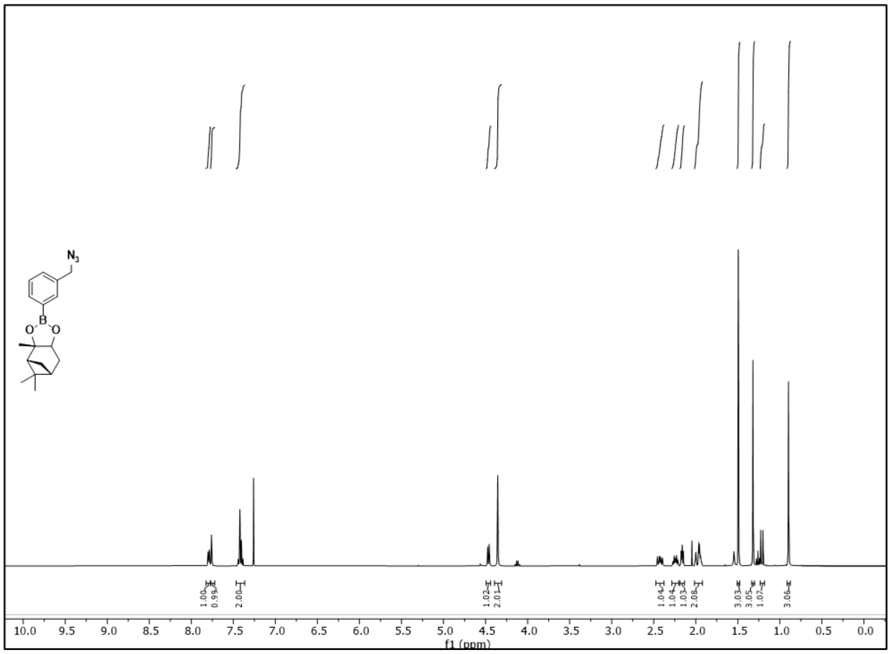

^{13}C NMR Spectrum of ***16***

400 MHz, $CDCl_3$

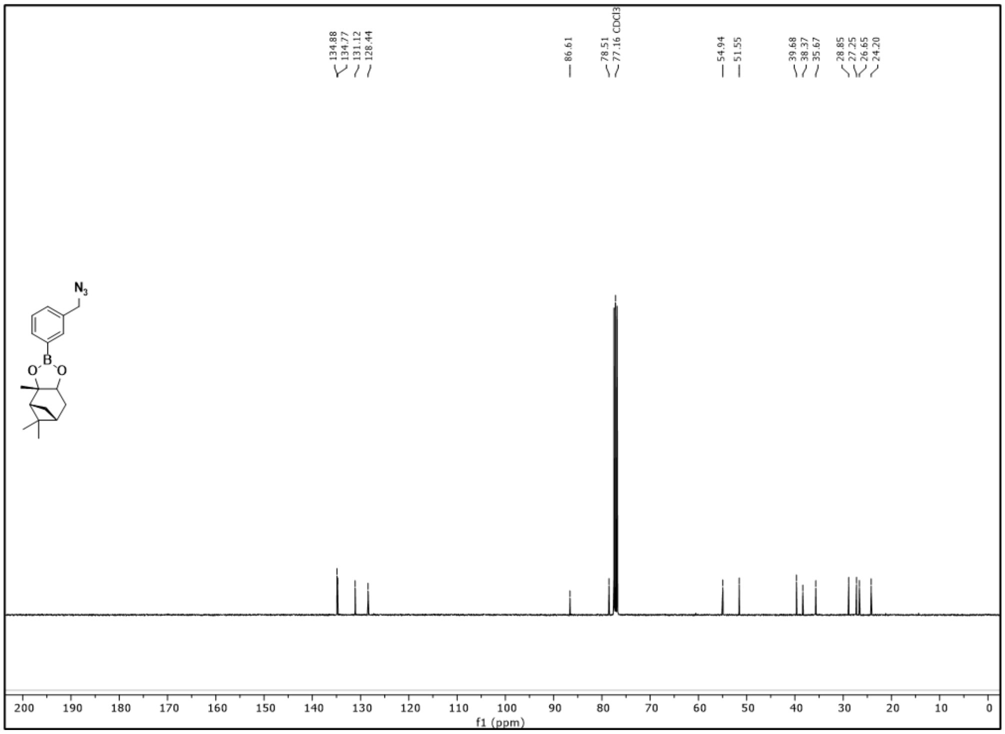

^{1}H NMR Spectrum of ***5_P***

600 MHz, $CDCl_3$

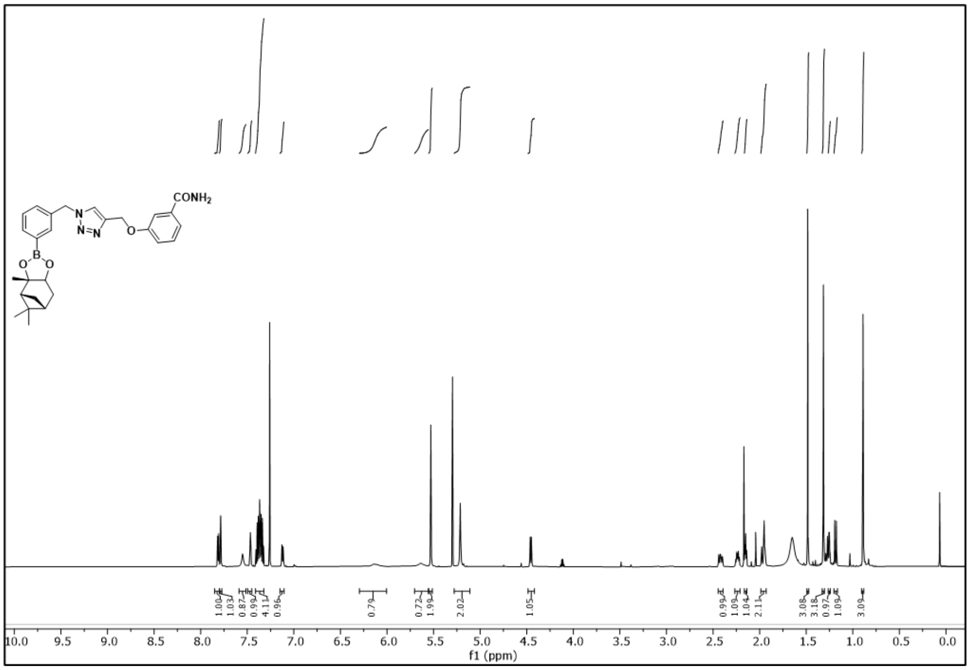

*^{13}C NMR Spectrum of **5_P***

151 MHz, $CDCl_3$

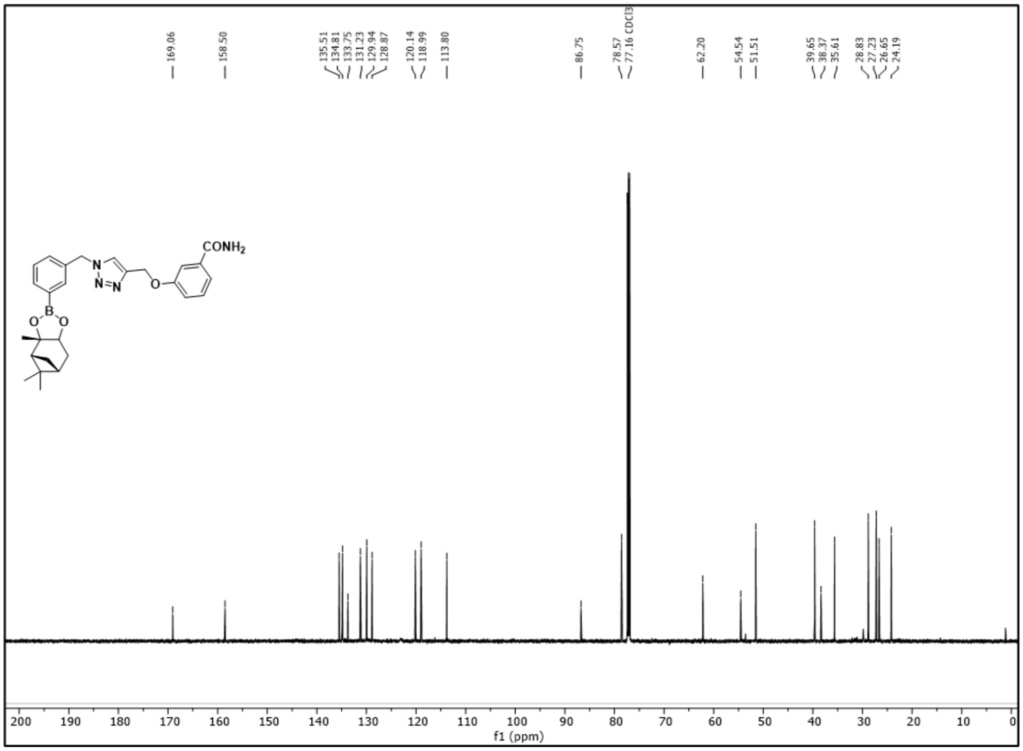

*^{1}H NMR Spectrum of **5***

600 MHz, MeOD

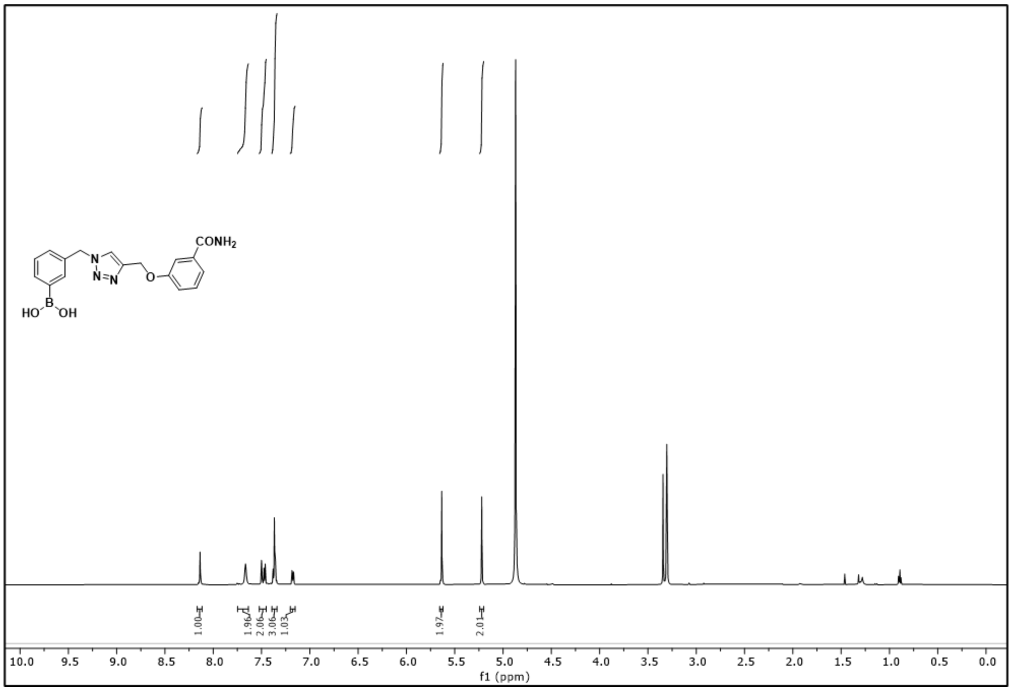

^{13}C NMR Spectrum of ***5***

151 MHz, MeOD

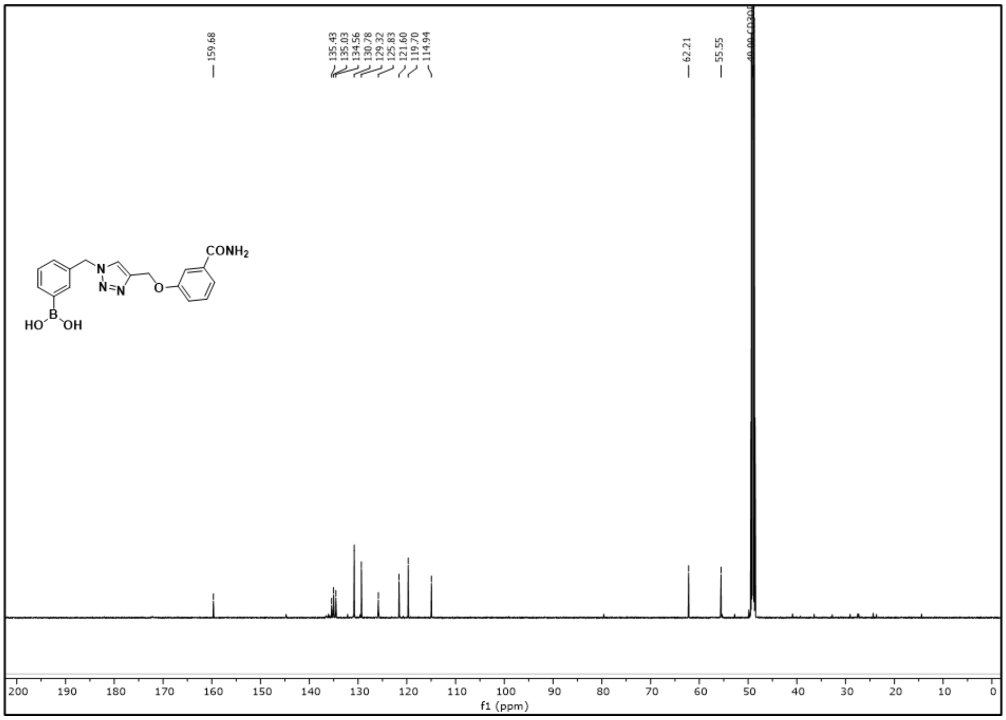

^{1}H NMR Spectrum of ***6_P***

600 MHz, $CDCl_3$

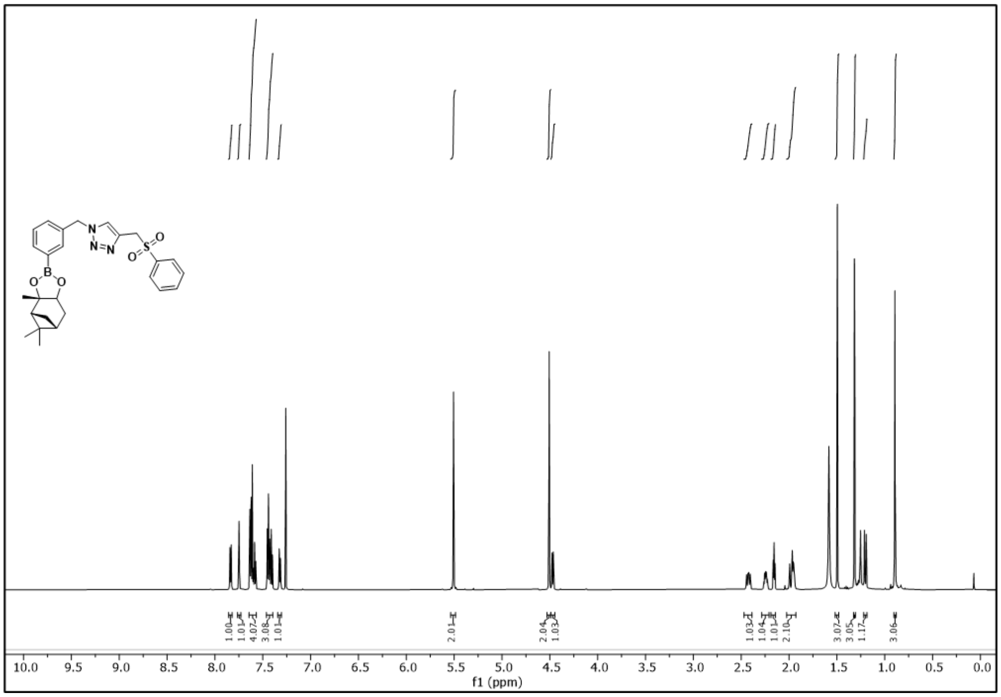

^{13}C NMR Spectrum of ***6_P***

151 MHz, $CDCl_3$

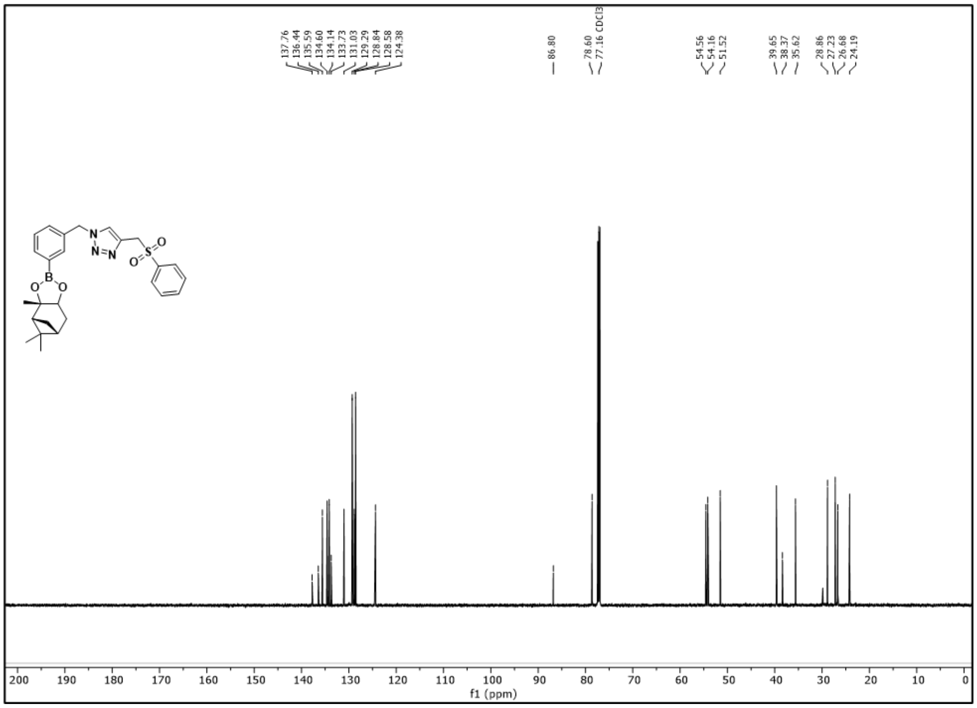

^{1}H NMR Spectrum of ***6***

600 MHz, MeOD

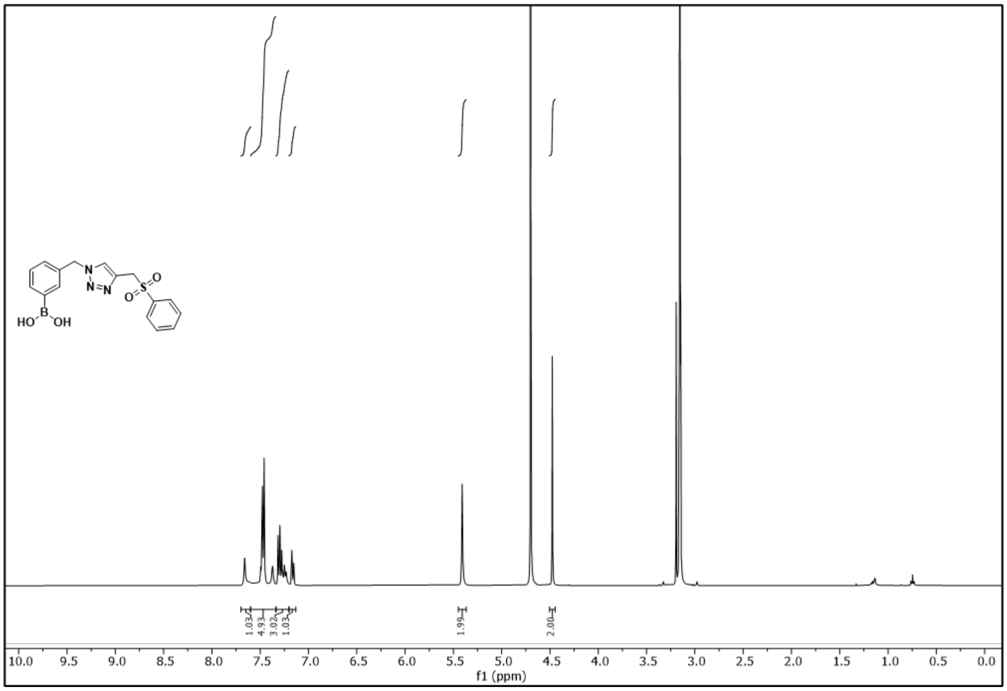

^{13}C NMR Spectrum of ***6***

151 MHz, MeOD

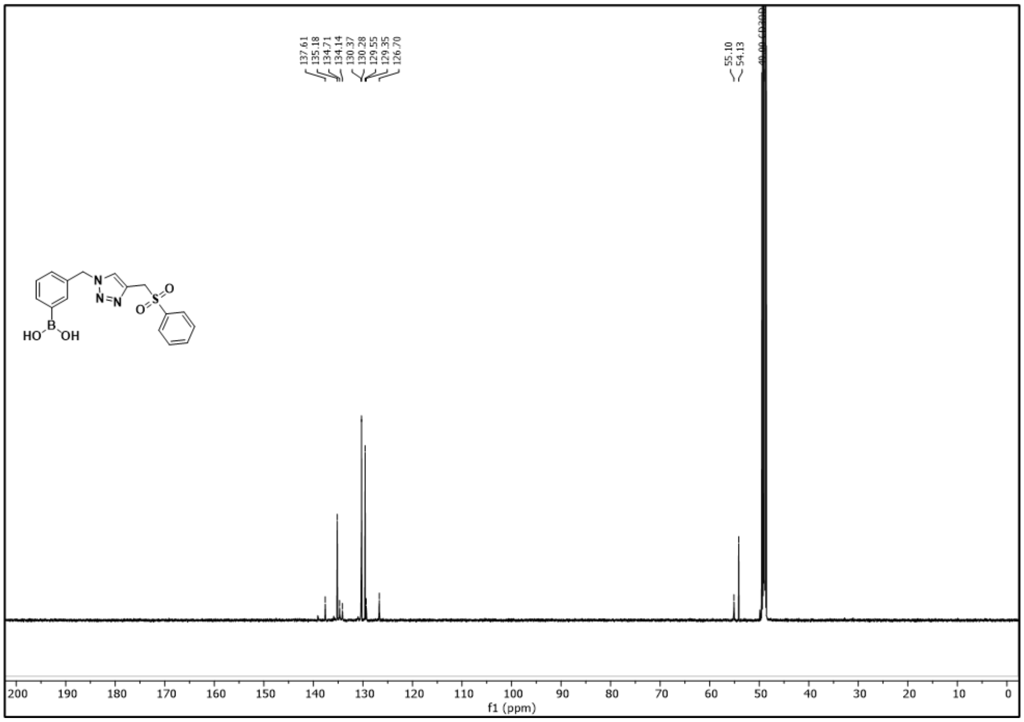

^{1}H NMR Spectrum of ***7_P***

600 MHz, $CDCl_3$

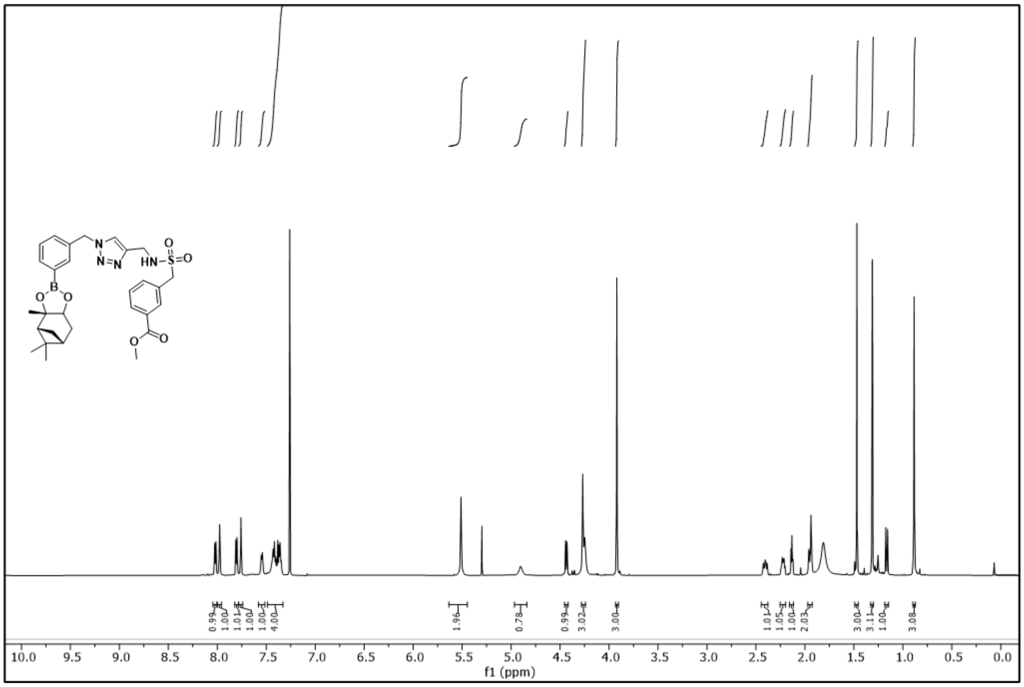

^{13}C NMR Spectrum of **7_P**

151 MHz, $CDCl_3$

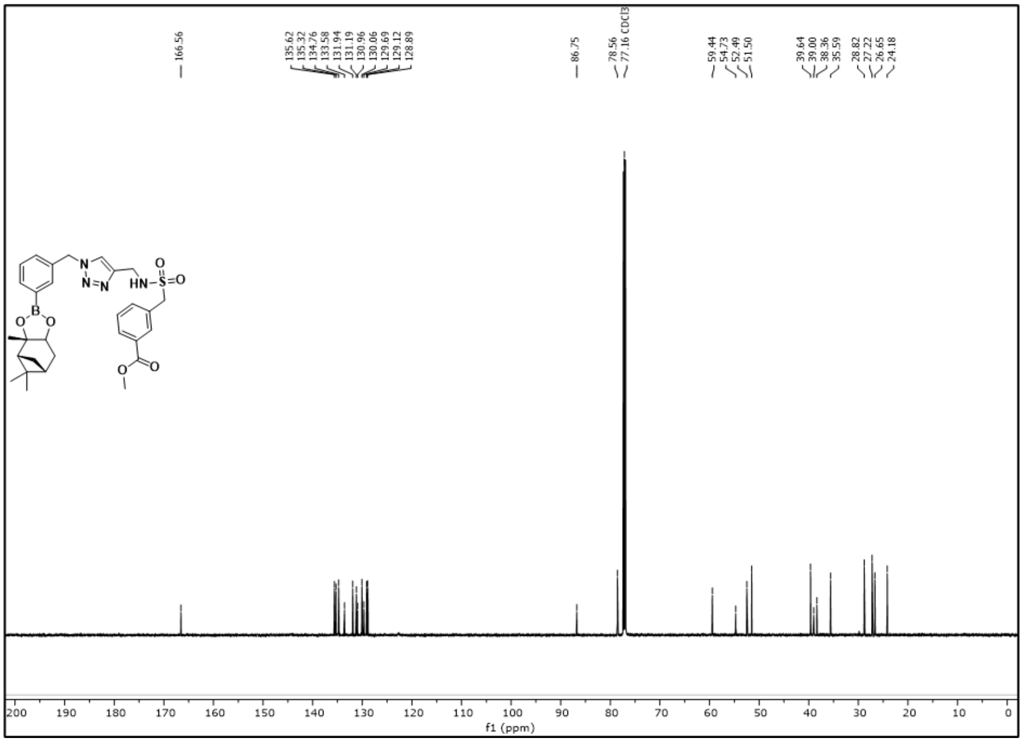

^{1}H NMR Spectrum of **7**

600 MHz, MeOD

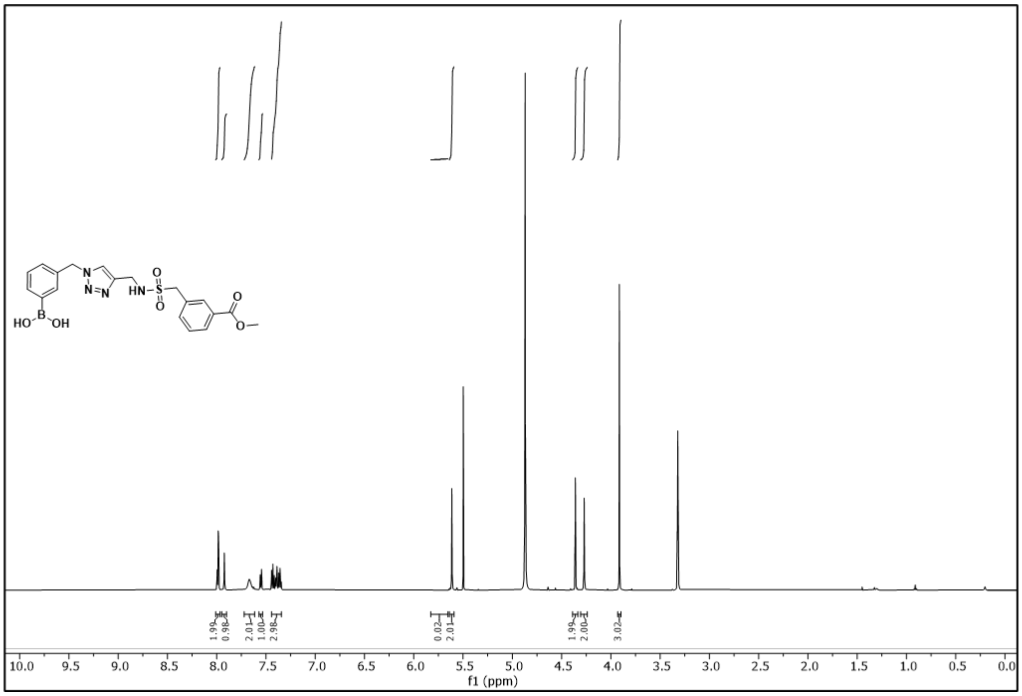

*^{13}C NMR Spectrum of **7***

151 MHz, MeOD

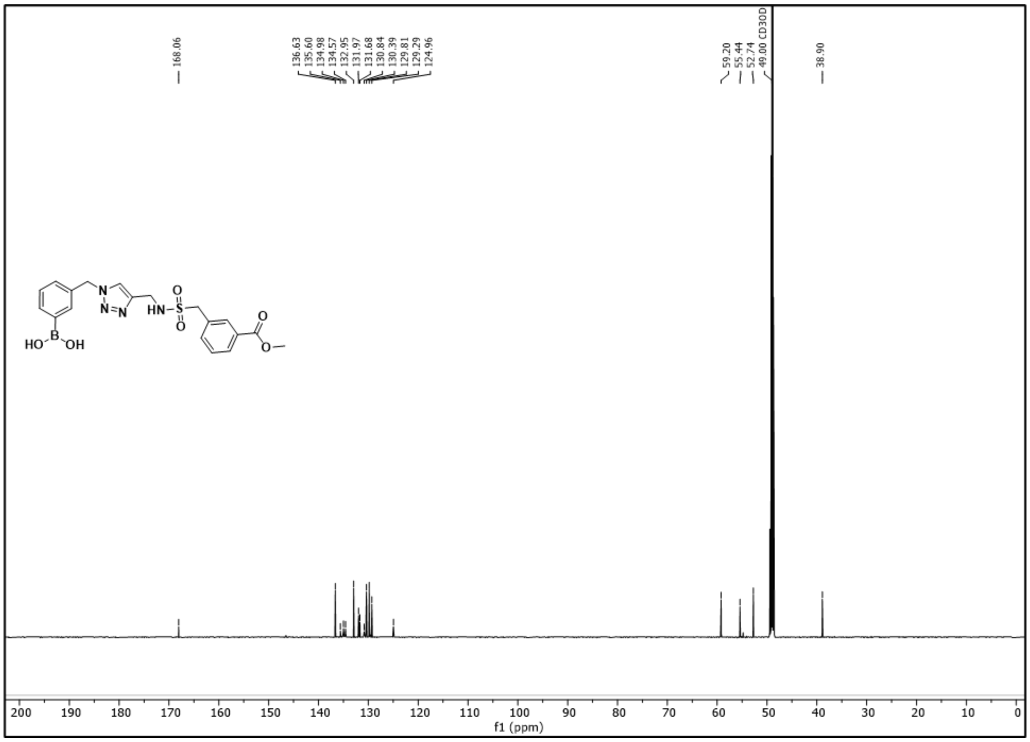

*^{1}H NMR Spectrum of **7a_P***

400 MHz, $CDCl_3$

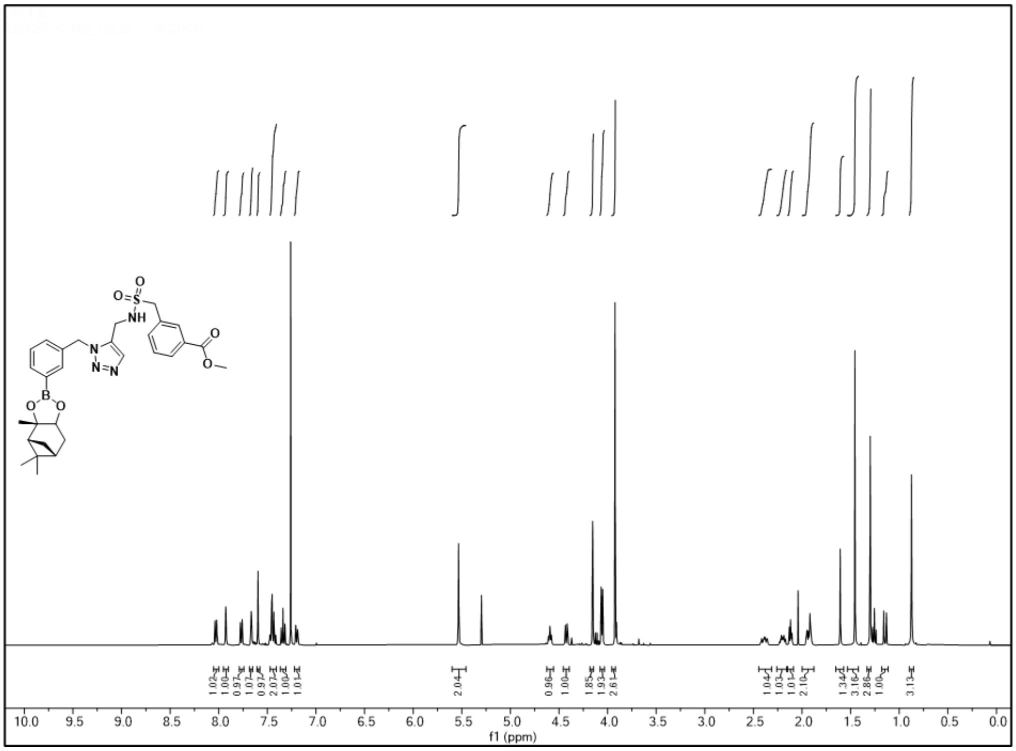

^{13}C NMR Spectrum of ***7a_P***

101 MHz, $CDCl_3$

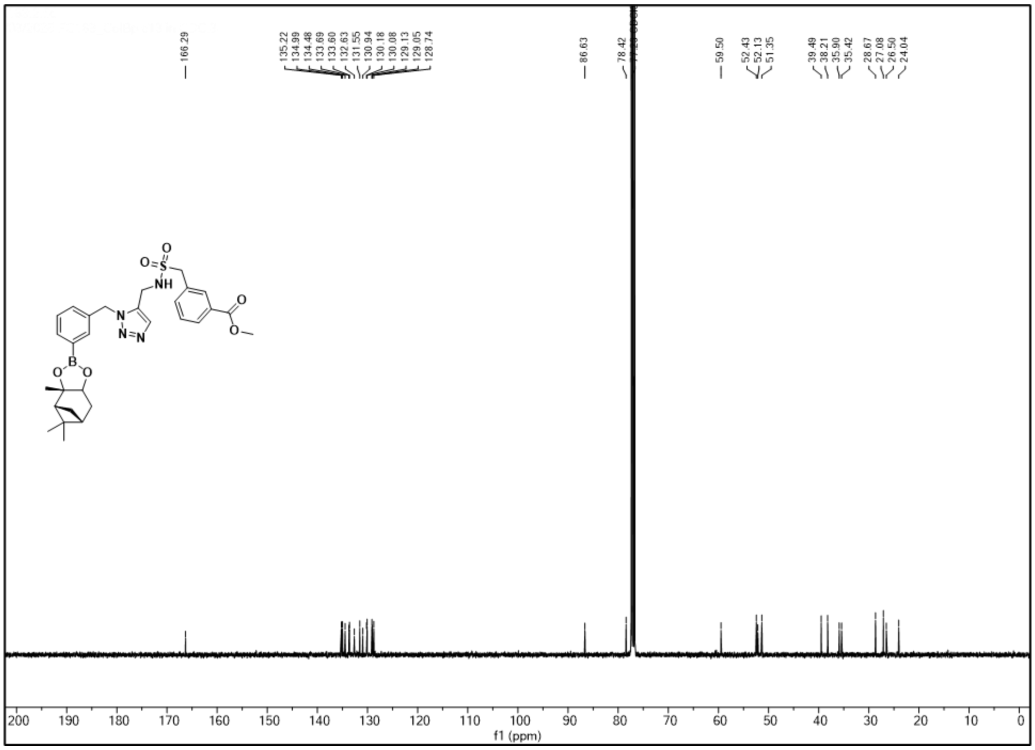

^{1}H NMR Spectrum of ***7a***

600 MHz, MeOD

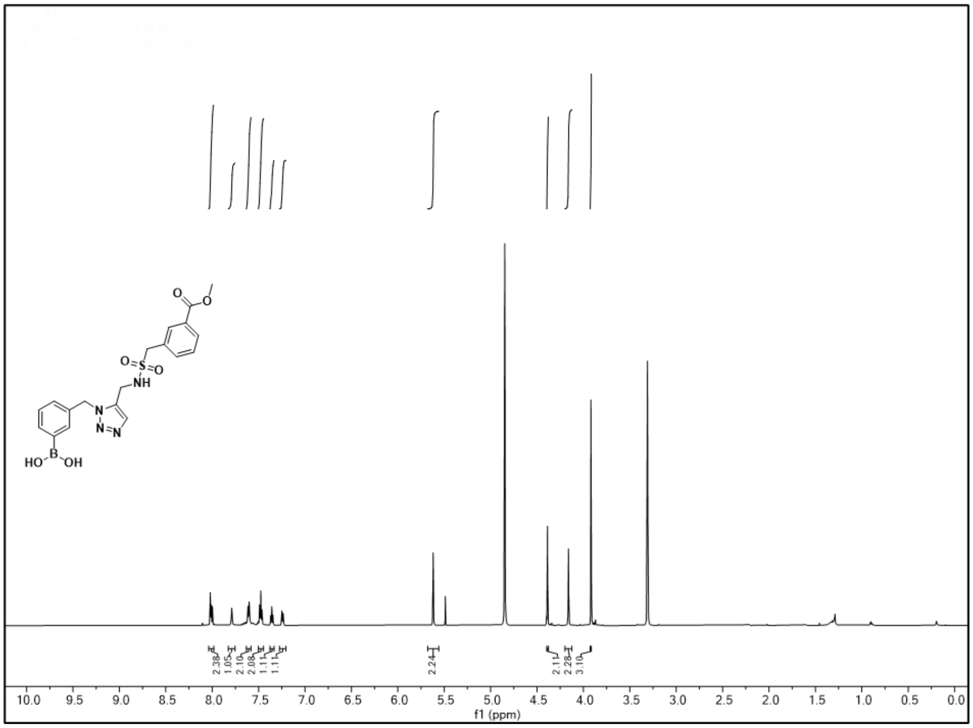

*^{13}C NMR Spectrum of **7a***

151 MHz, MeOD

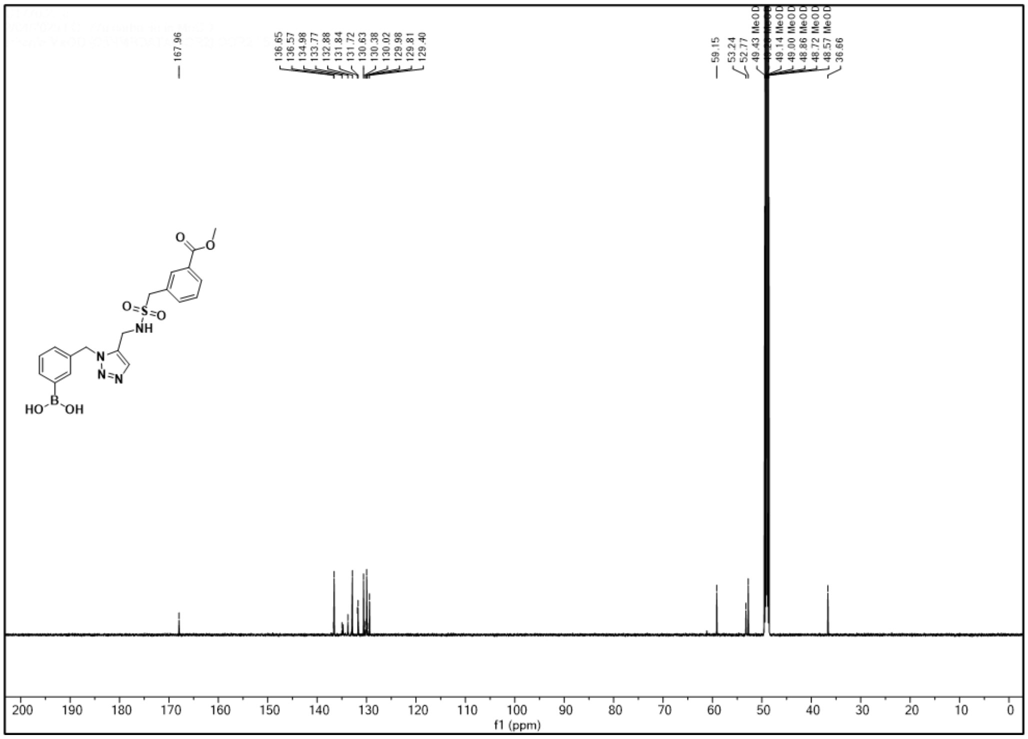

*^{1}H NMR Spectrum of **8_P***

600 MHz, $CDCl_3$

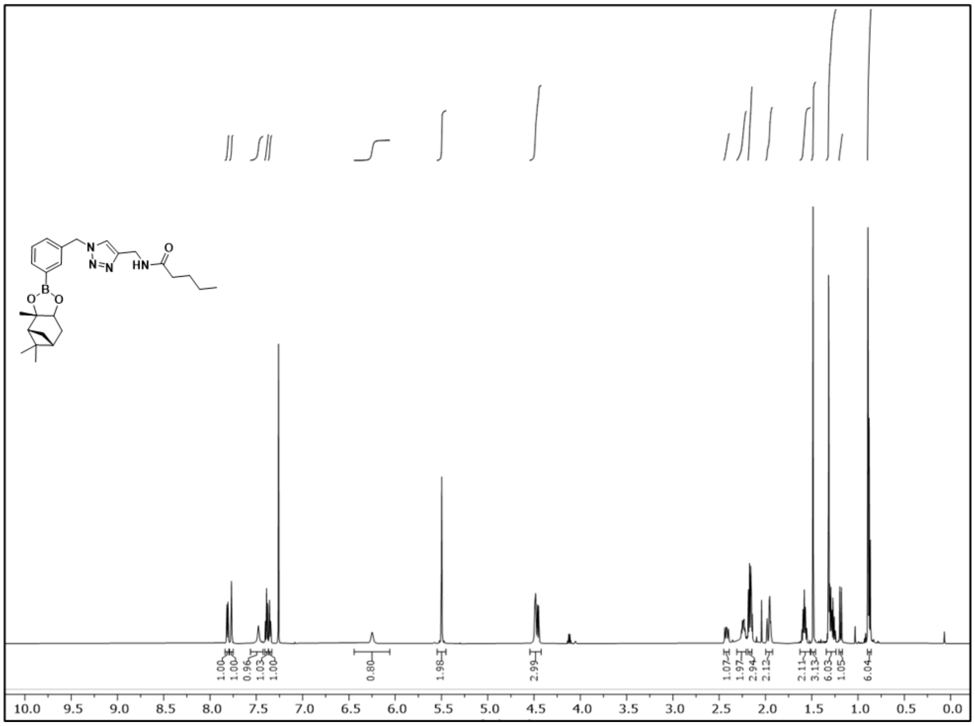

*^{13}C NMR Spectrum of **8_P***

151 MHz, $CDCl_3$

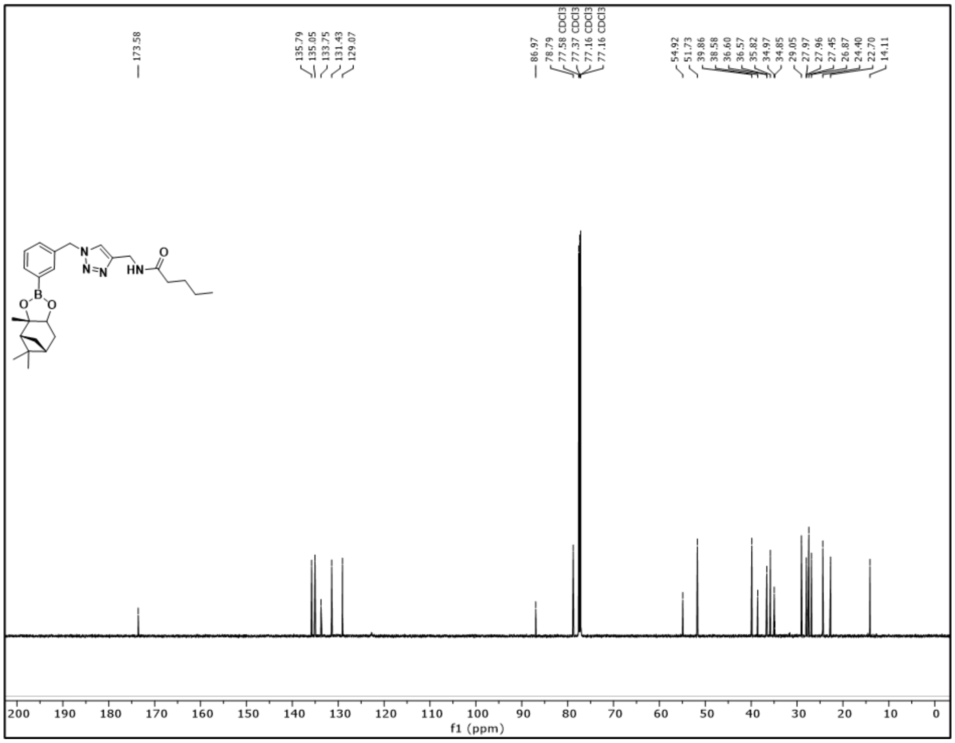

*^{1}H NMR Spectrum of **8***

600 MHz, MeOD

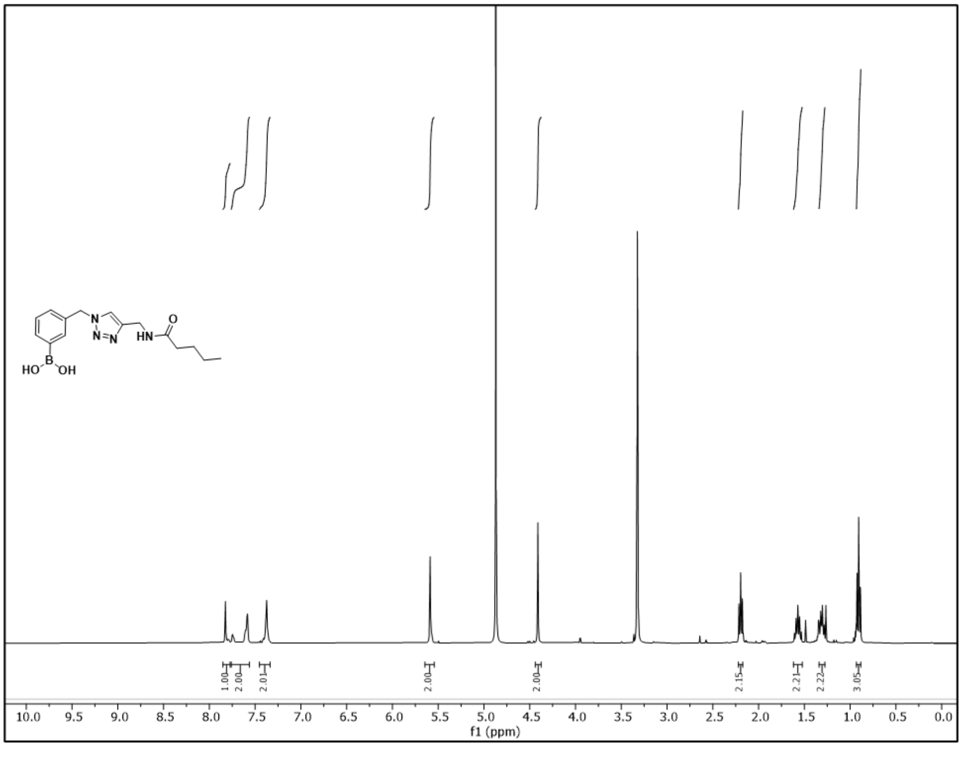

^{13}C NMR Spectrum of ***8***

151 MHz, MeOD

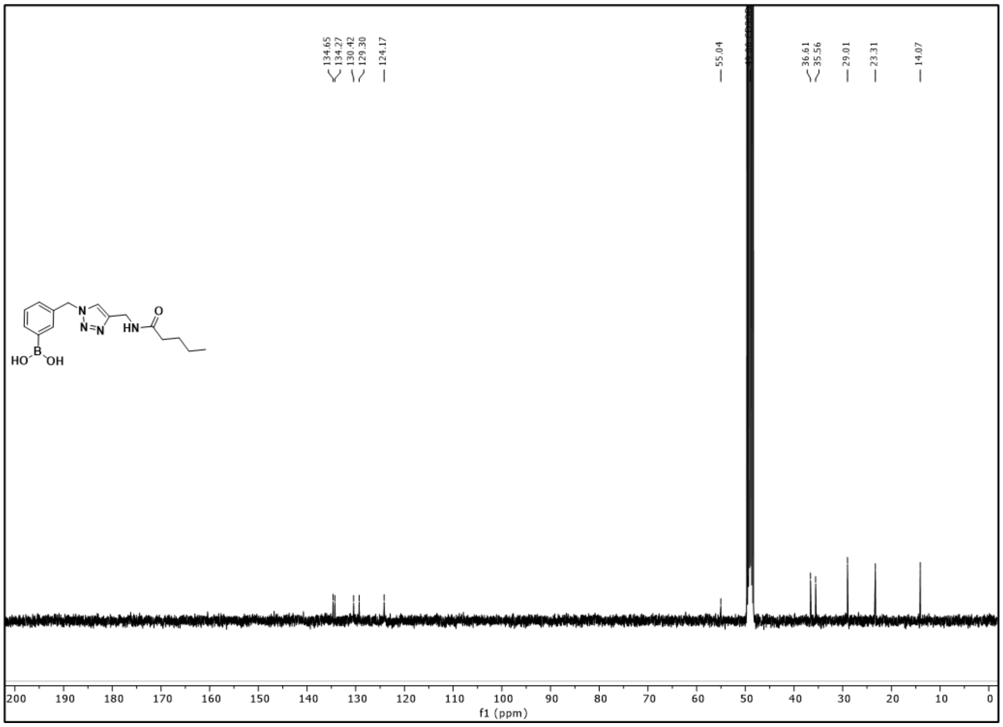

^{1}H NMR Spectrum of ***9_P***

600 MHz, $CDCl_3$

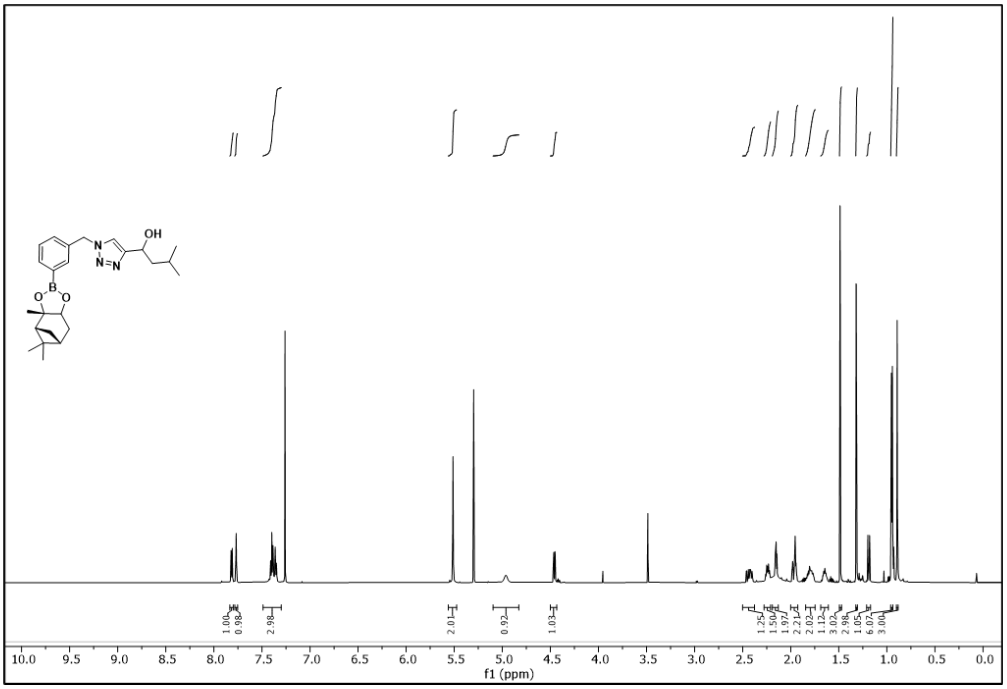

*^{13}C NMR Spectrum of **9_P***

151 MHz, $CDCl_3$

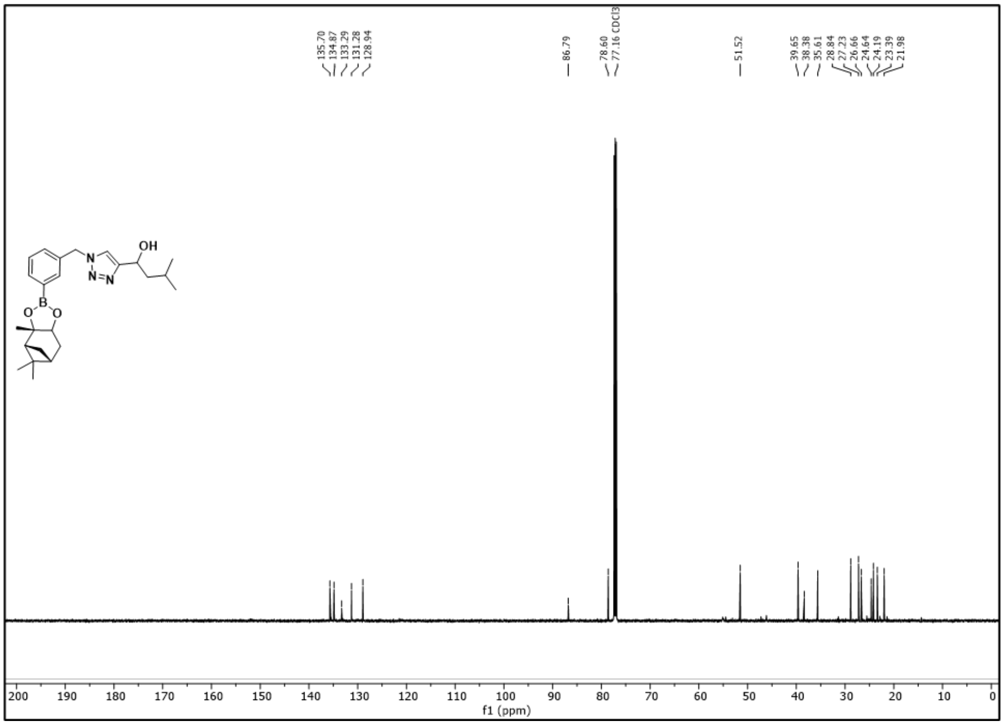

*^{1}H NMR Spectrum of **9***

400 MHz, *d6*-DMSO

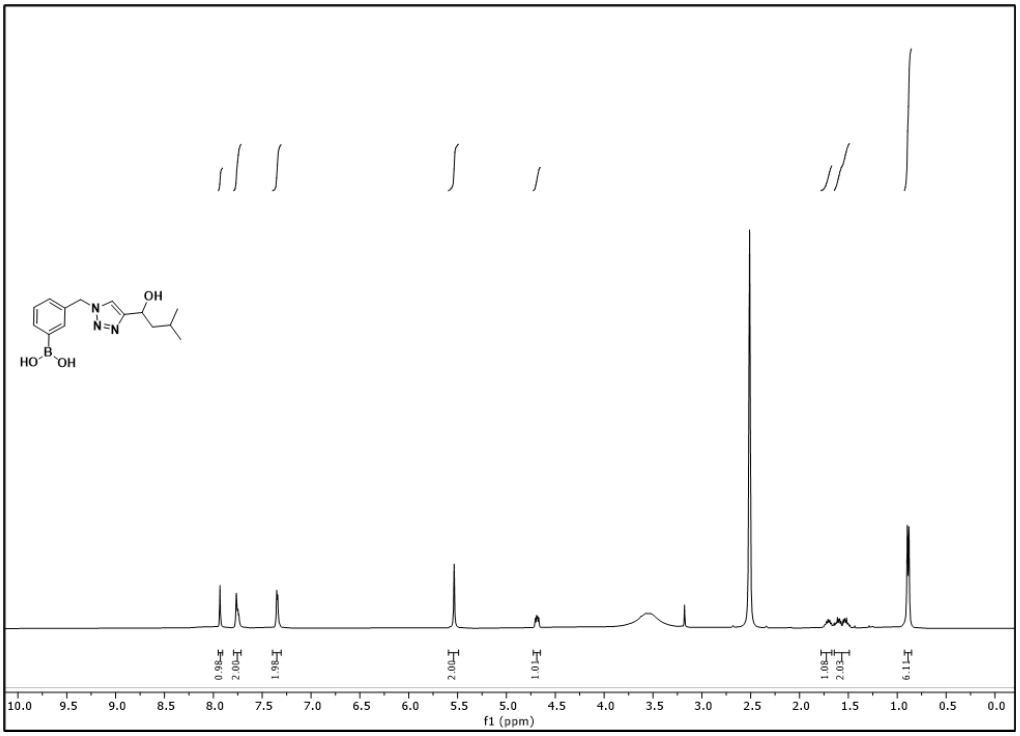

*^{13}C NMR Spectrum of **9***

101 MHz, *d6*-DMSO

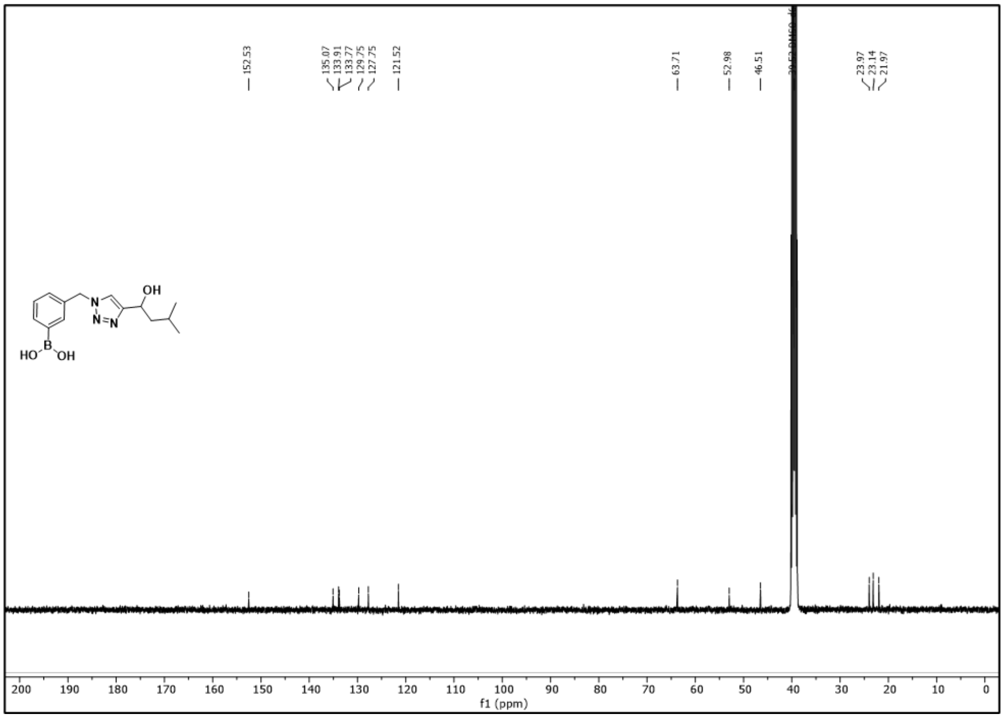

*^{1}H NMR Spectrum of **10_P***

600 MHz, $CDCl_3$

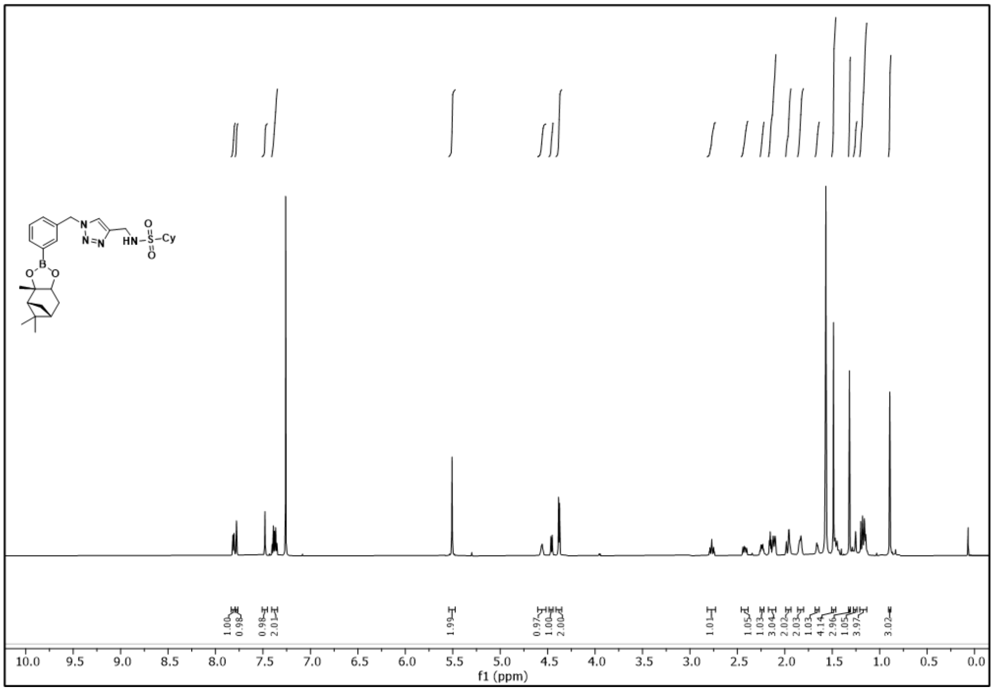

^{13}C *NMR Spectrum of* ***10_P***

151 MHz, $CDCl_3$

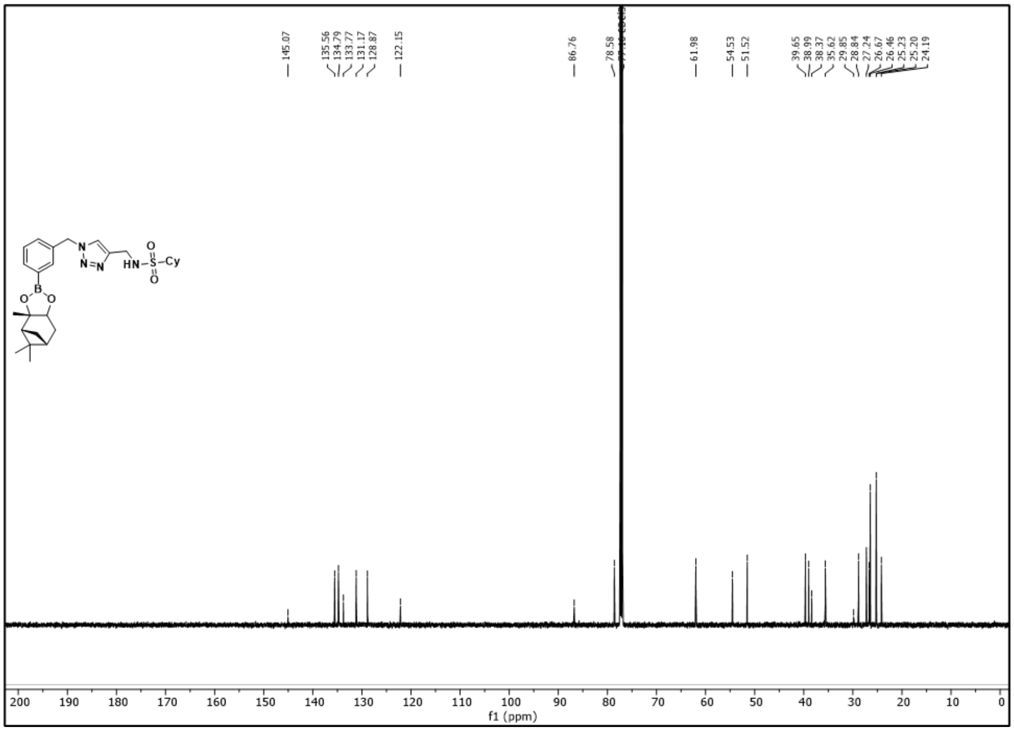

^{1}H *NMR Spectrum of* ***10***

600 MHz, MeOD

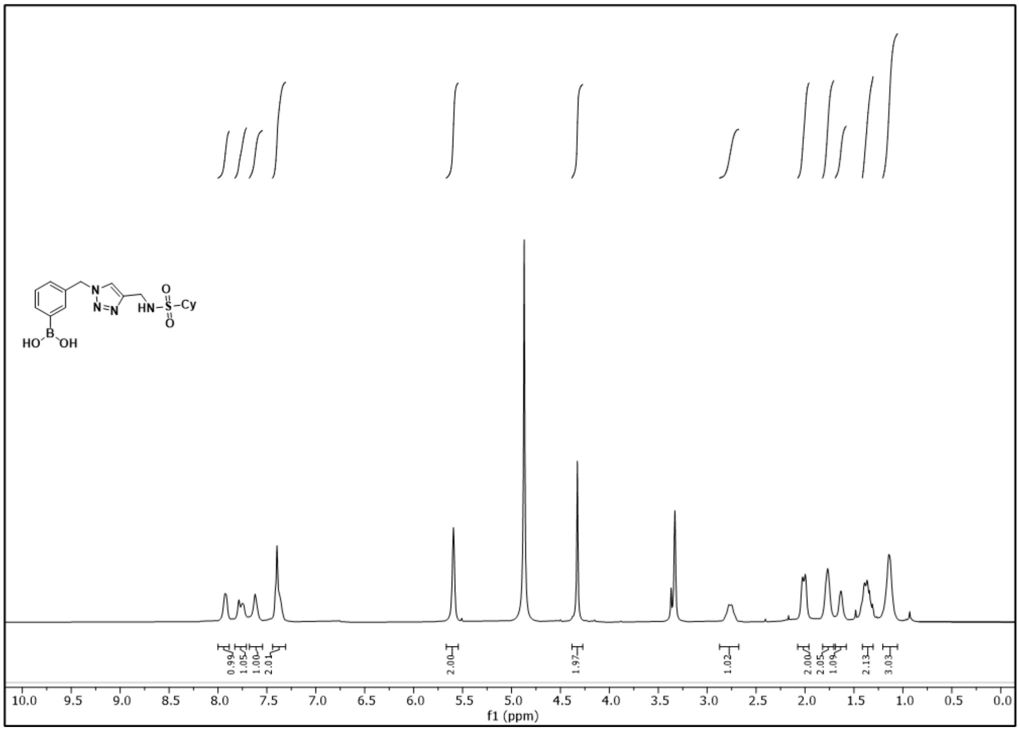

*^{13}C NMR Spectrum of **10***

151 MHz, MeOD

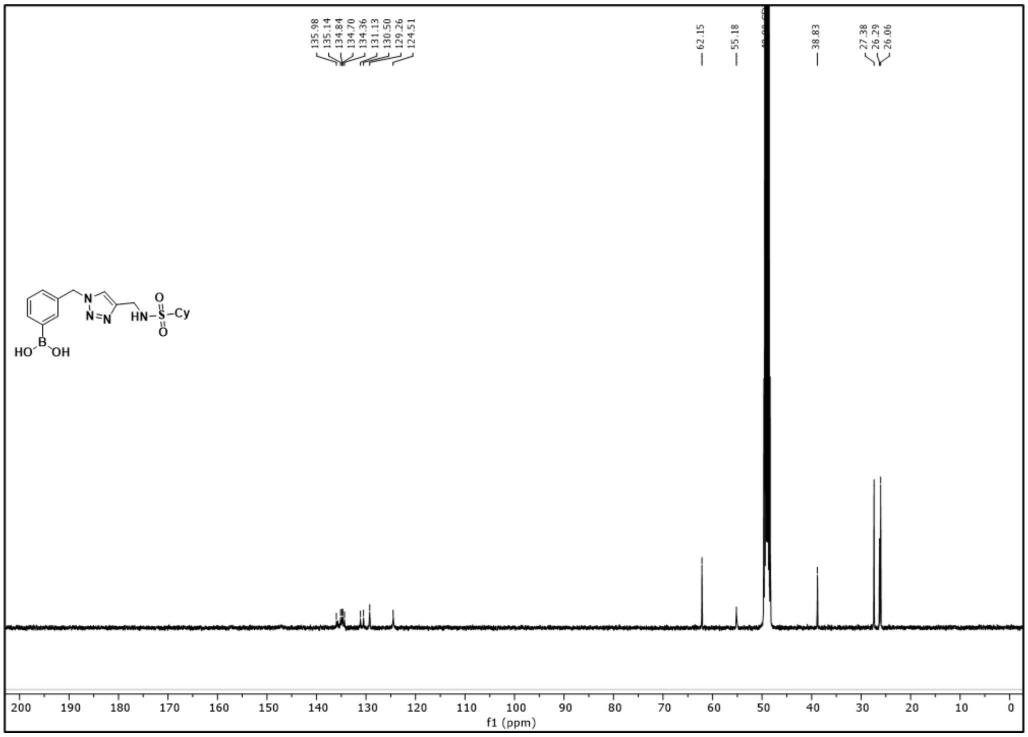

*^{1}H NMR Spectrum of **10a_P***

600 MHz, $CDCl_3$

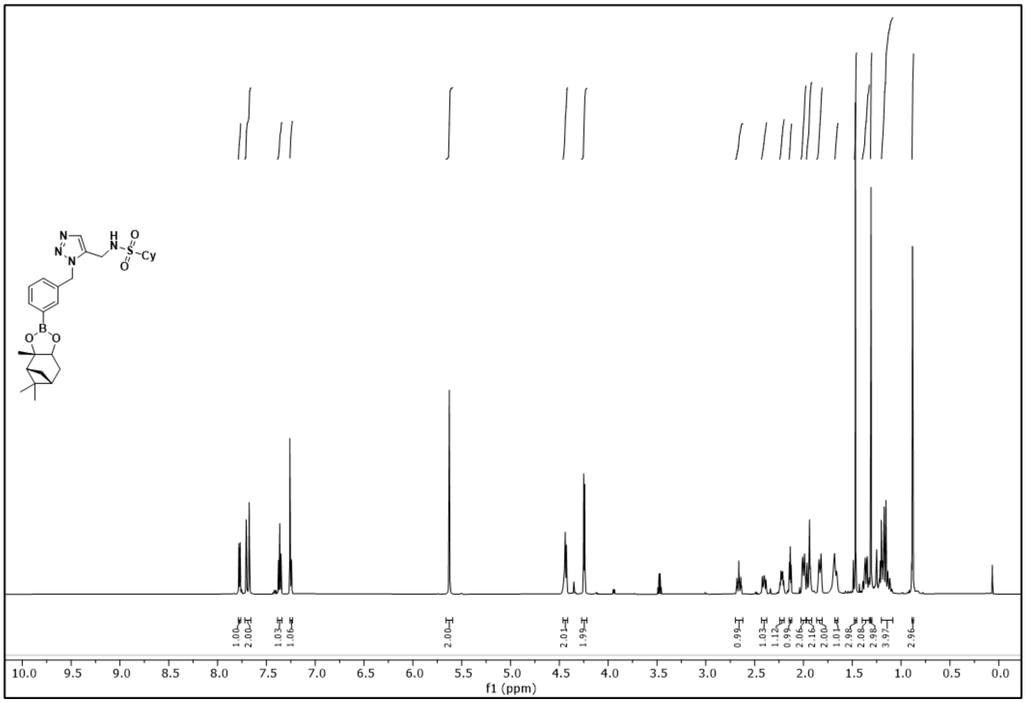

^{13}C NMR Spectrum of ***10a_P***

151 MHz, $CDCl_3$

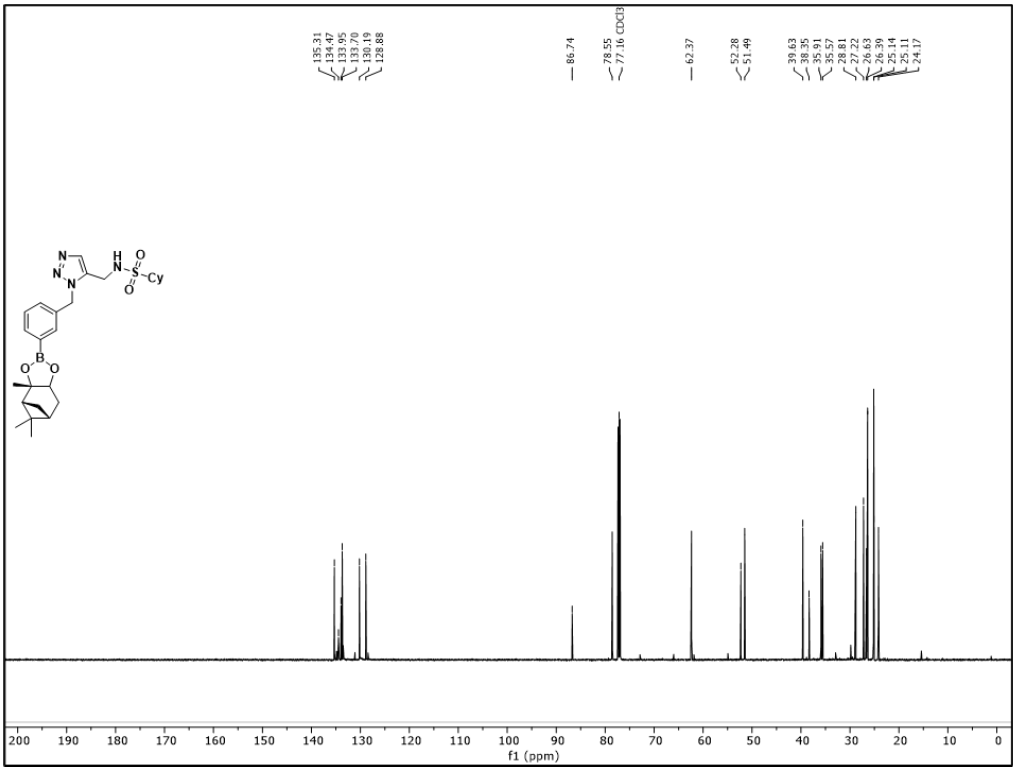

^{1}H NMR Spectrum of ***10a***

600 MHz, MeOD

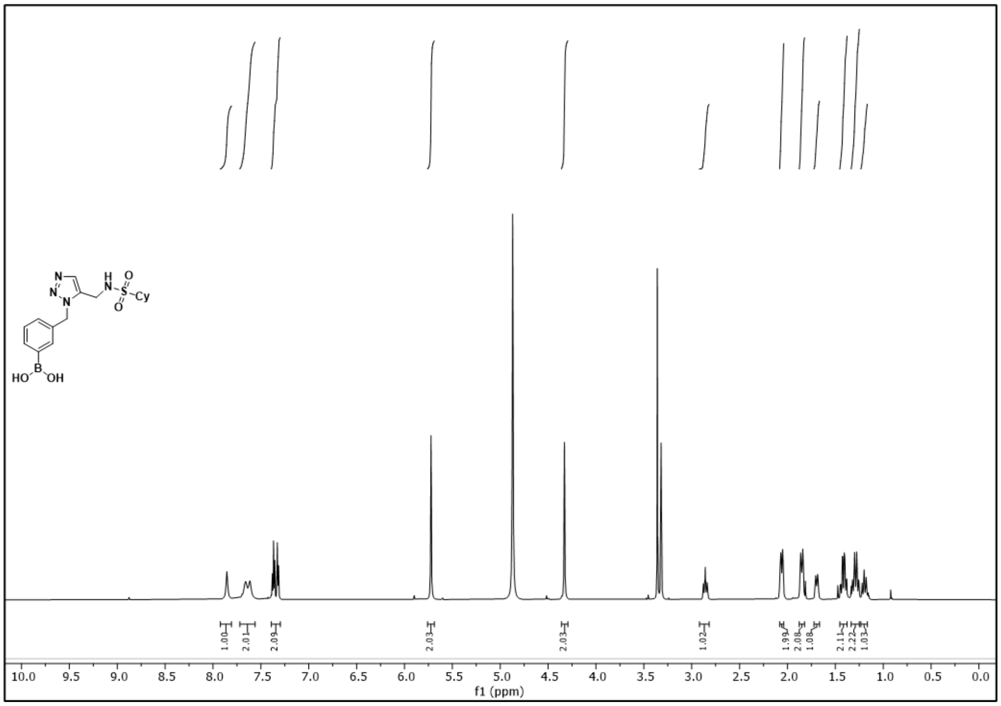

^{13}C NMR Spectrum of ***10a***

151 MHz, MeOD

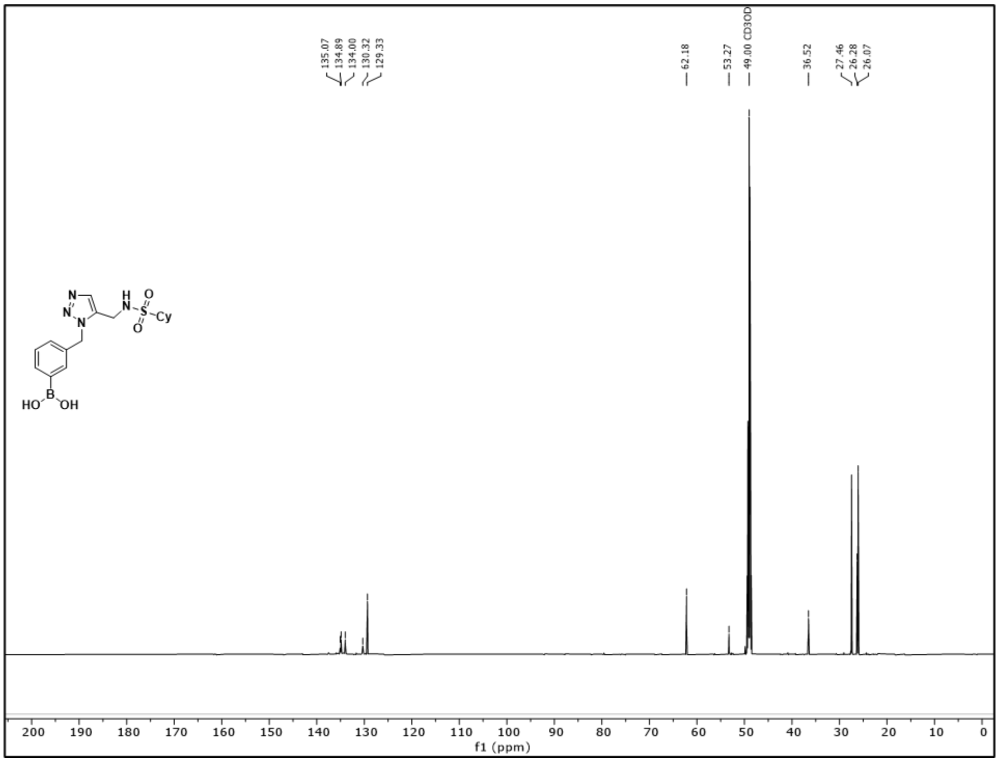

^{1}H NMR Spectrum of ***11_P***

600 MHz, $CDCl_3$

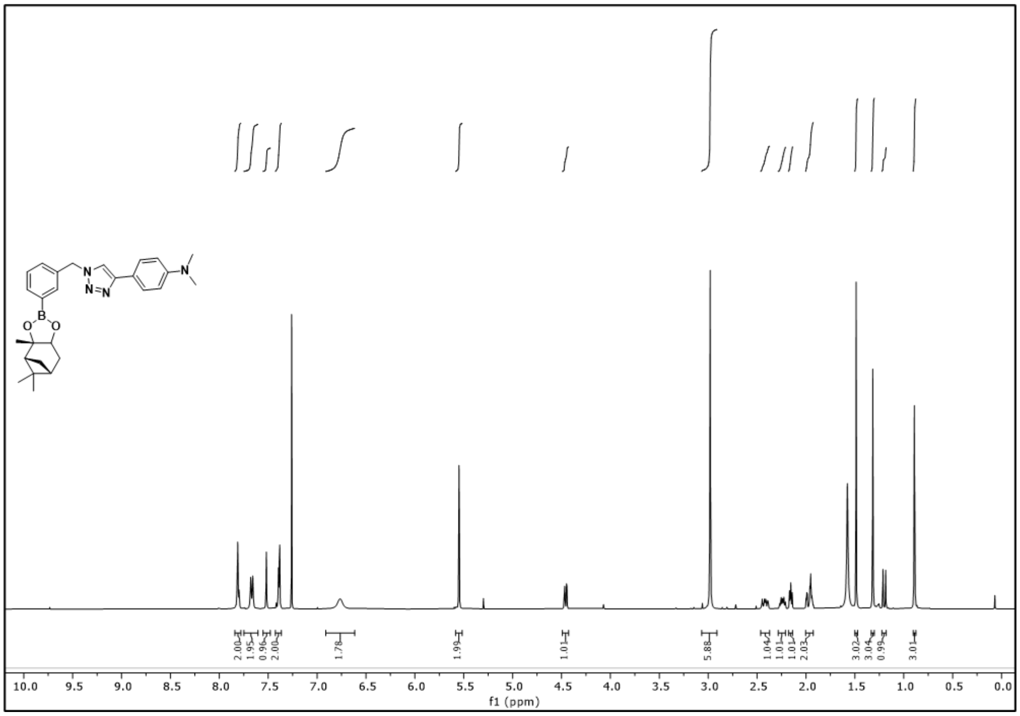

^{13}C NMR Spectrum of ***11_P***

151 MHz, $CDCl_3$

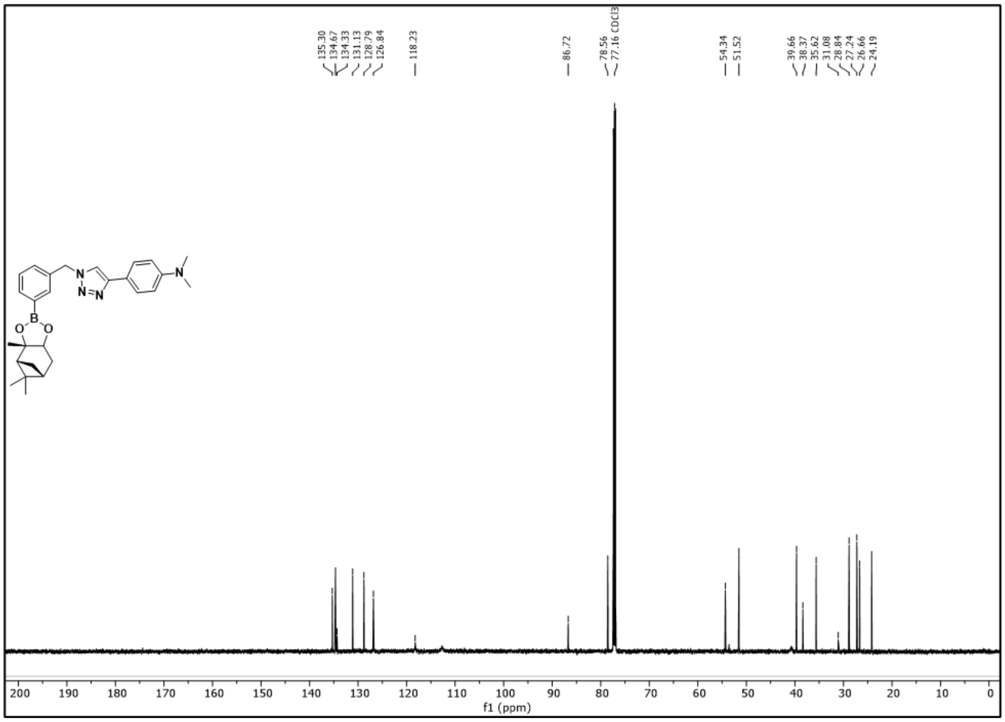

^{1}H NMR Spectrum of ***11***

600 MHz, MeOD

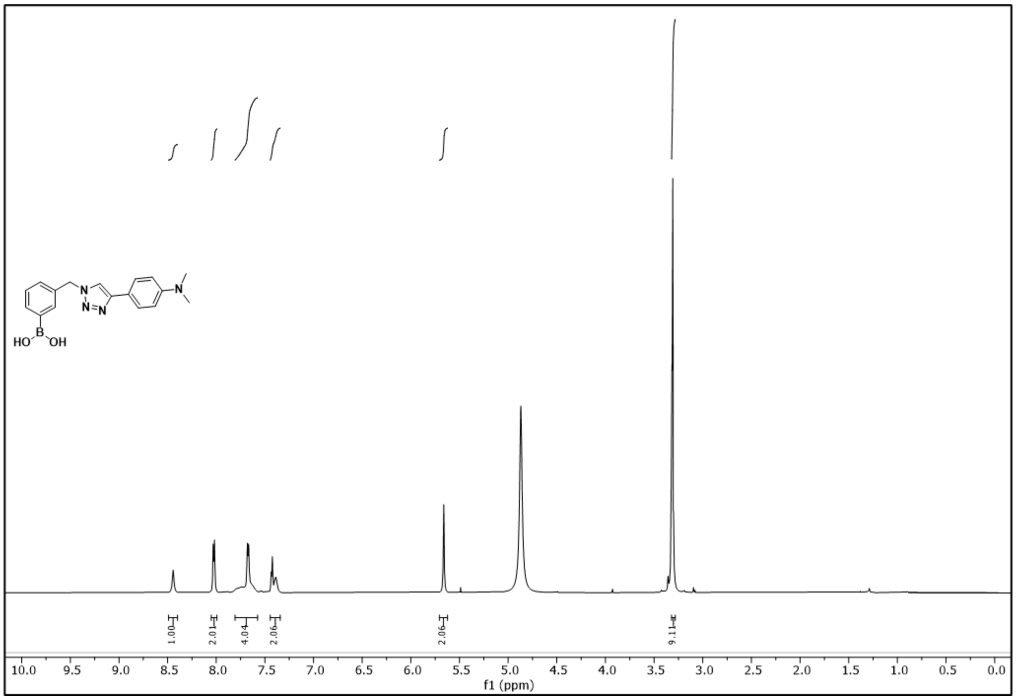

^{13}C NMR Spectrum of ***11***

151 MHz, MeOD

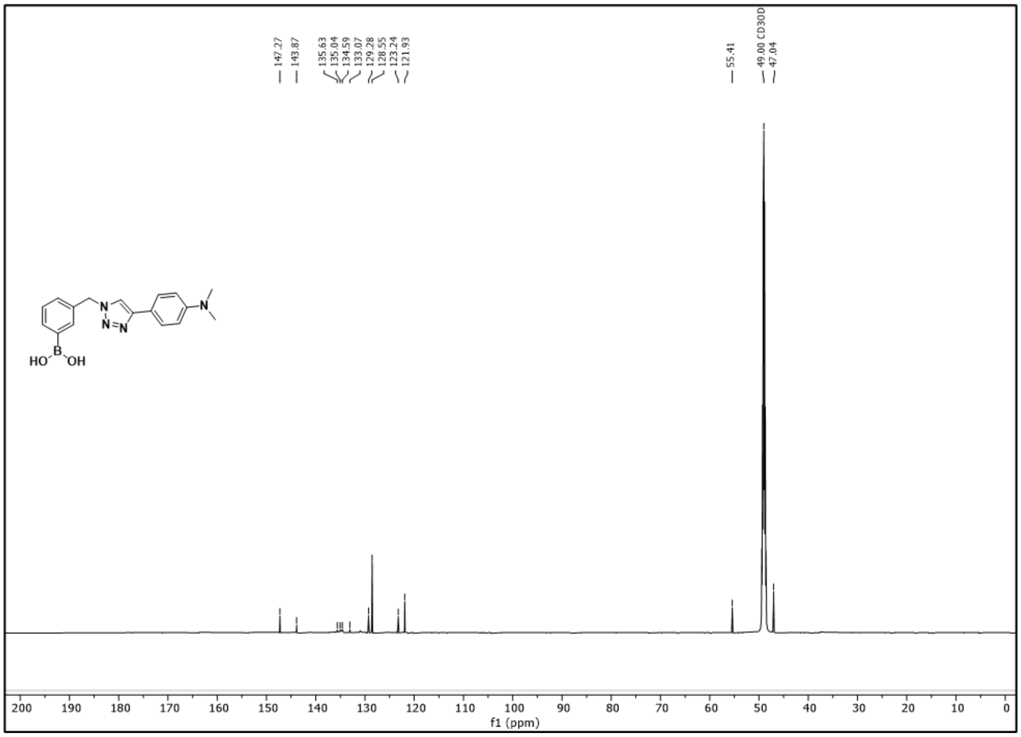

^{1}H NMR Spectrum of ***12_P***

600 MHz, $CDCl_3$

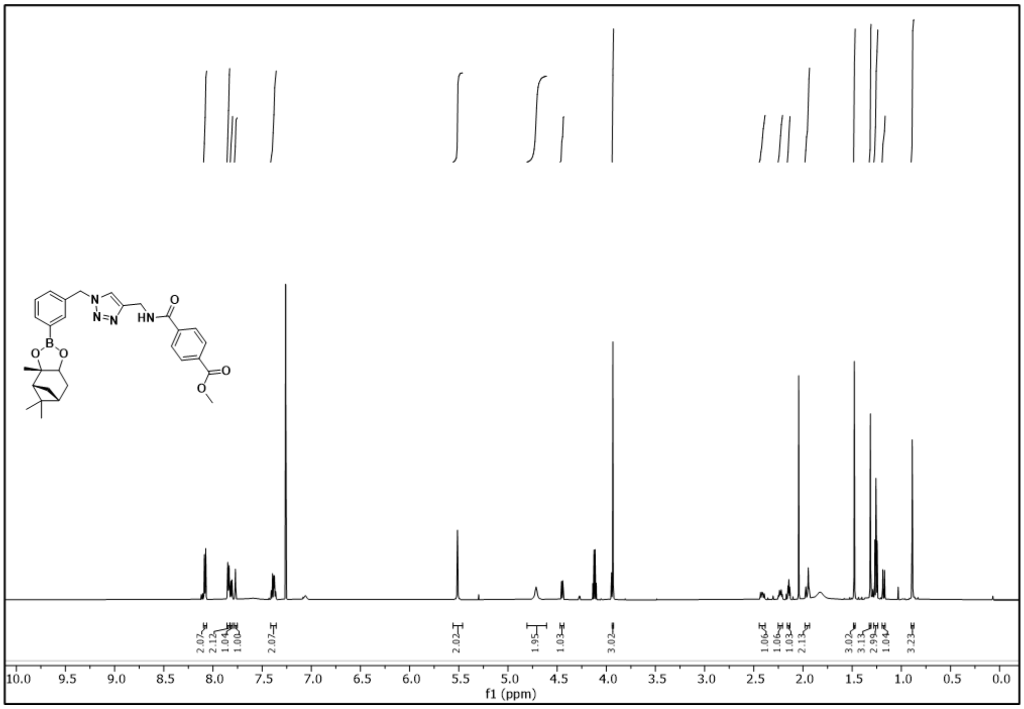

^{13}C NMR Spectrum of ***12_P***

151 MHz, $CDCl_3$

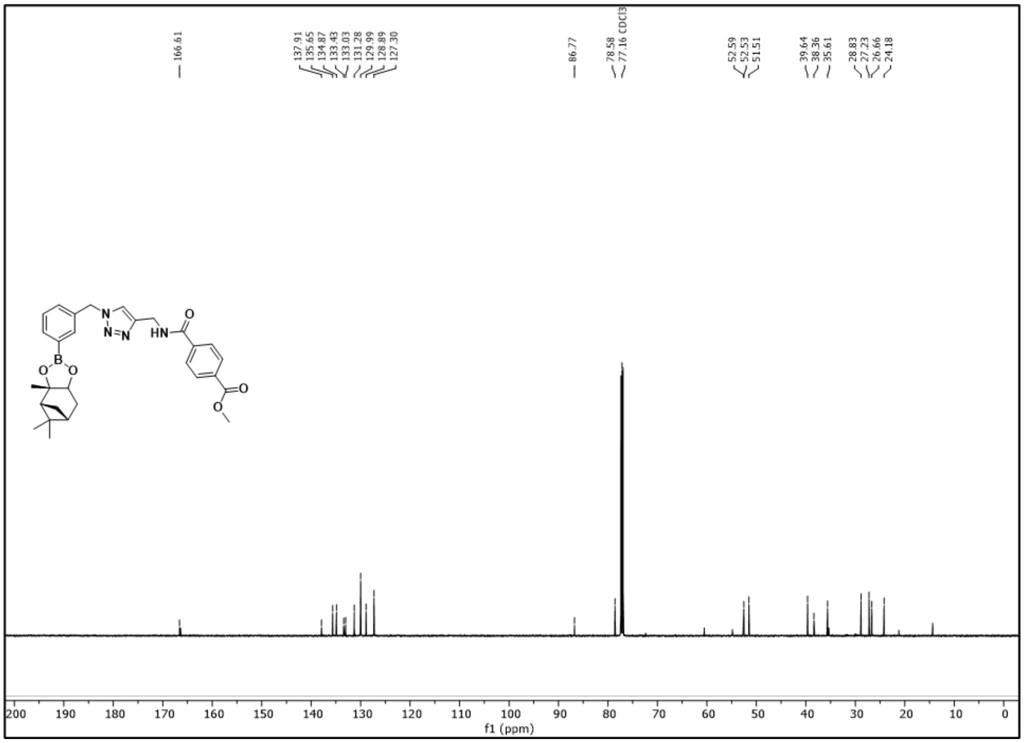

^{1}H NMR Spectrum of ***12***

600 MHz, MeOD

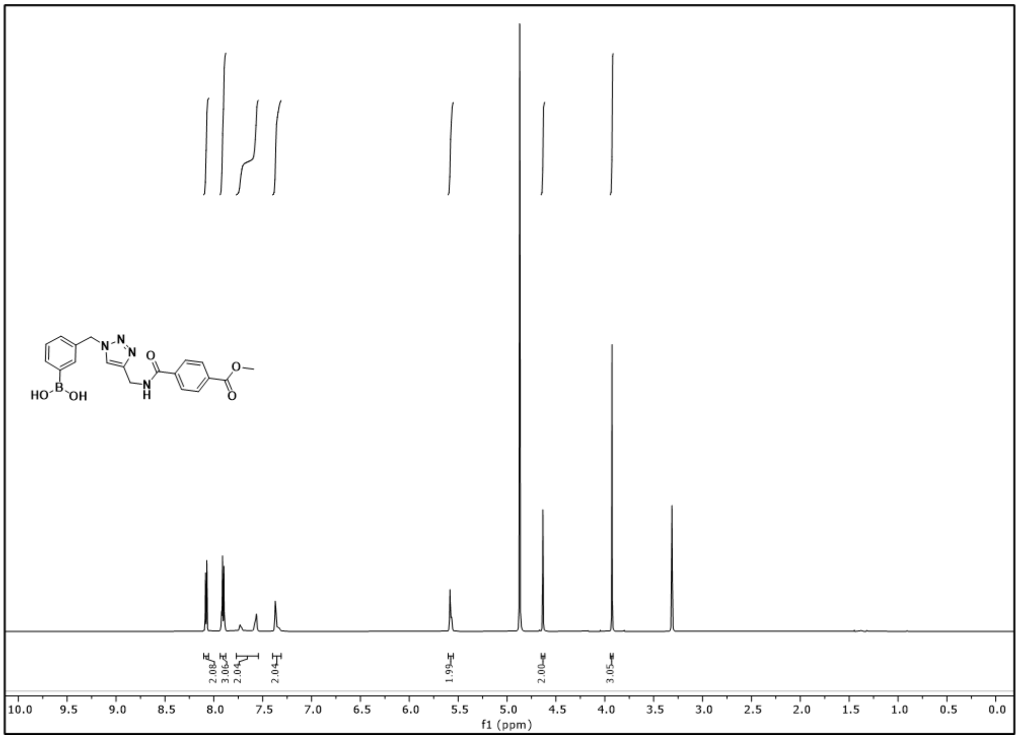

*^{13}C NMR Spectrum of **12***

151 MHz, MeOD

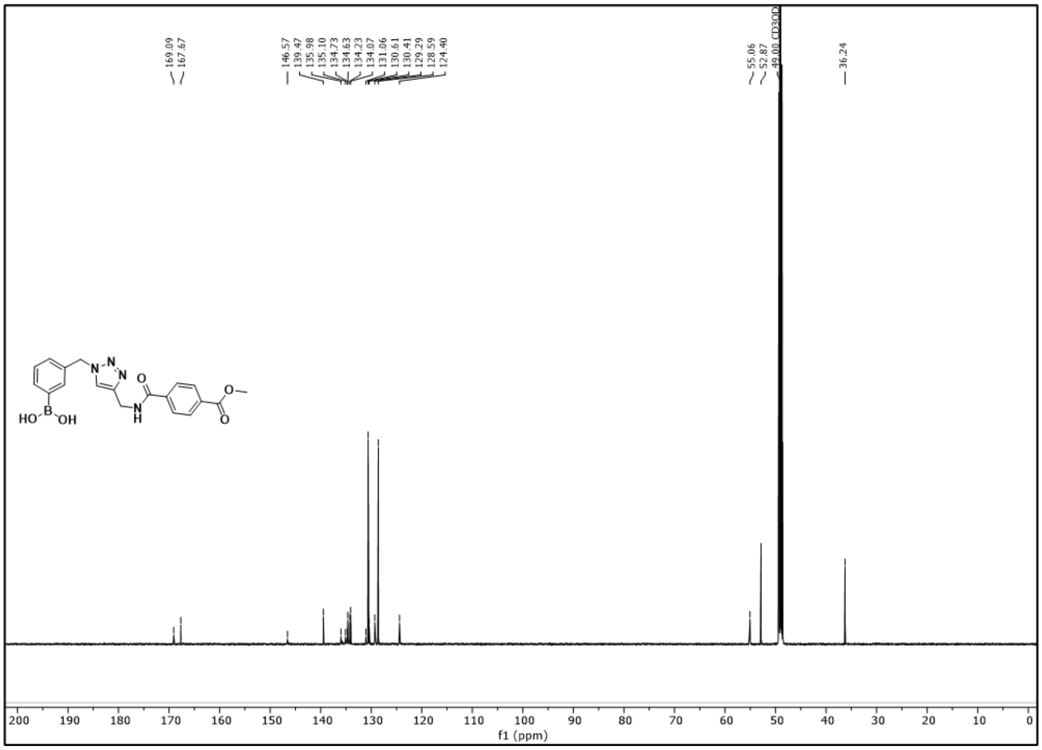

*^{1}H NMR Spectrum of **13_P***

600 MHz, $CDCl_3$

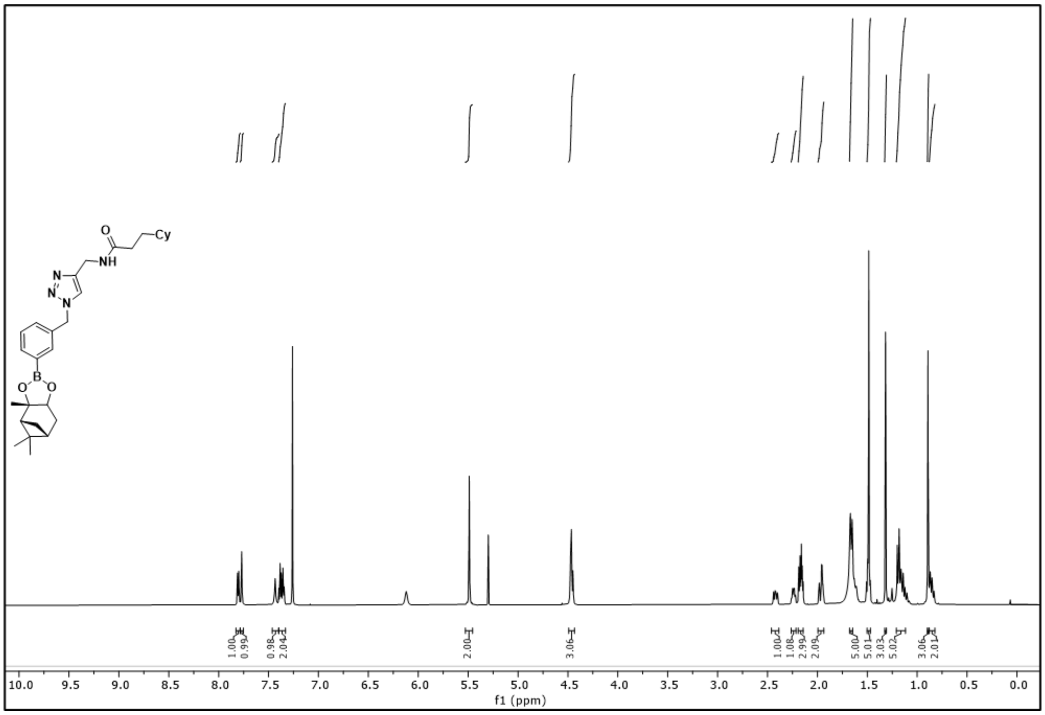

^{13}C NMR Spectrum of ***13_P***

151 MHz, $CDCl_3$

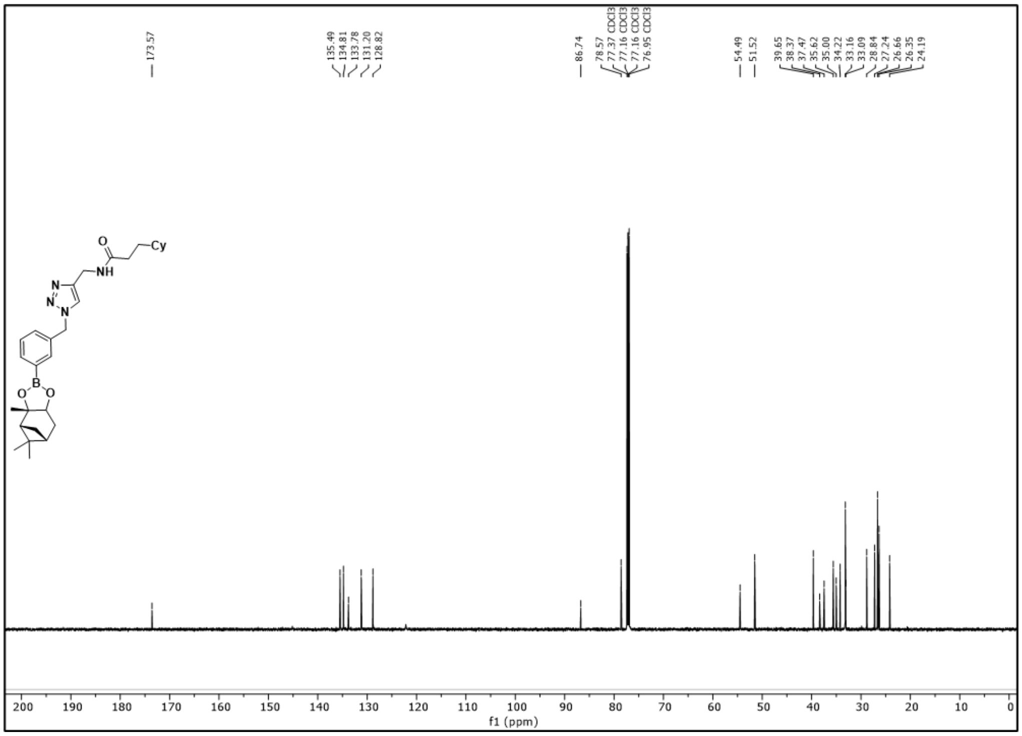

^{1}H NMR Spectrum of ***13***

600 MHz, MeOD

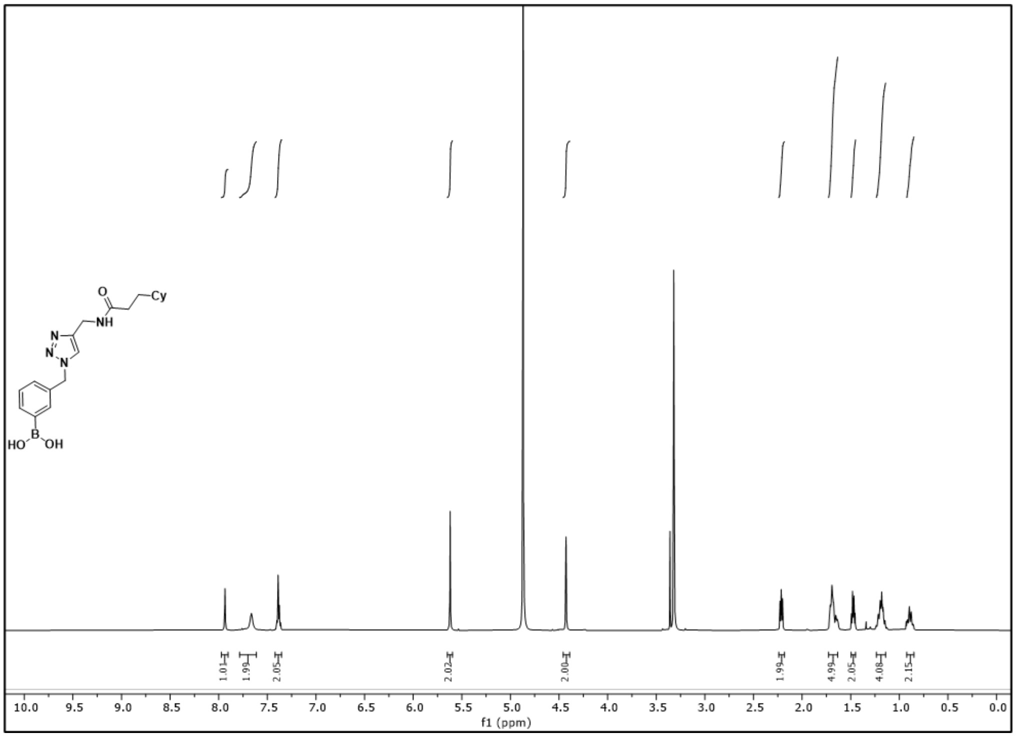

^{13}C NMR Spectrum of ***13***

151 MHz, MeOD

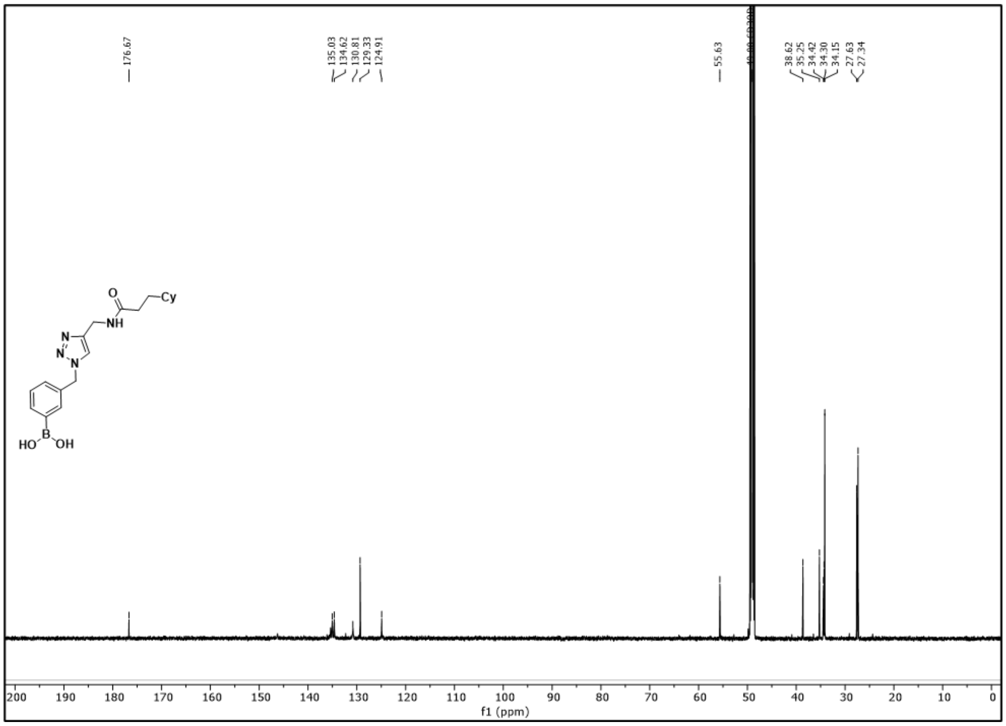

Supplement: Supplementary file 1 [file ijms-26-04182-s001.zip › ijms-3536564-supplementary.pdf]
